# Green, one-pot strategy for substitution of the 5-position of 1,10-phenanthrolines

**DOI:** 10.1039/d6ra04592h

**Published:** 2026-09-03

**Authors:** Jonathan P. Wheeler, Eli M. Espinoza, Kritika Sharma, Nicolas Durand, Felix N. Castellano

**Affiliations:** a Department of Chemistry, North Carolina State University Raleigh North Carolina 27695- 8204 USA fncastel@ncsu.edu

## Abstract

The preparation of symmetric 1,10-phenanthroline derivatives has been thoroughly explored, while substitution of the 5- or 6-positions on the phenanthroline backbone is less understood. Existing methods for forming these synthetic moieties (*e.g.*, 5-nitro-1,10-phenanthroline) utilize highly toxic reagents and harsh conditions such as high temperatures and pressures. Curiously, alkylation at different phenanthroline positions affects both product formation rates and the products themselves. As a result, 5-substituted phenanthroline derivatives are often either expensive or unavailable commercially. A previously discovered breakthrough involves the formation of 1*a*,9*b*-dihydrooxireno[2,3-f][1,10]phenanthroline (epoxyphen). This synthetically expedient intermediate permits facile formation of 5-substituted derivatives of 1,10-phenanthroline and 2,9-dimethyl-1,10-phenanthroline upon treatment with strongly nucleophilic salts and solvation in methanol/water mixtures. Substitution with synthetically advantageous functional groups, such as nitrite, azide, benzenethiolate, imidazolate, cyanide, or arylsulfinates enables subsequent reactions to form carboxylic acids, primary amines, and reactive intermediates for click chemistry. These reactions proceed *via* an S_N_2-like mechanism, in which ring-opening, driven by a loss of ring strain, precedes an elimination process that deprotonates the substituted phenanthroline carbon and eliminates the *in situ*-formed hydroxyl group. This mechanism results in moderate to good yields, facile purification, and high purity while replacing toxic reagents and energy-intensive conditions.

## Abstract 2

Aqueous solvent systems are used to prepare a range of 5-substituted-1,10-phenanthrolines using commercial bleach and sodium salt-based nucleophiles under mild conditions.

## Graphical abstract

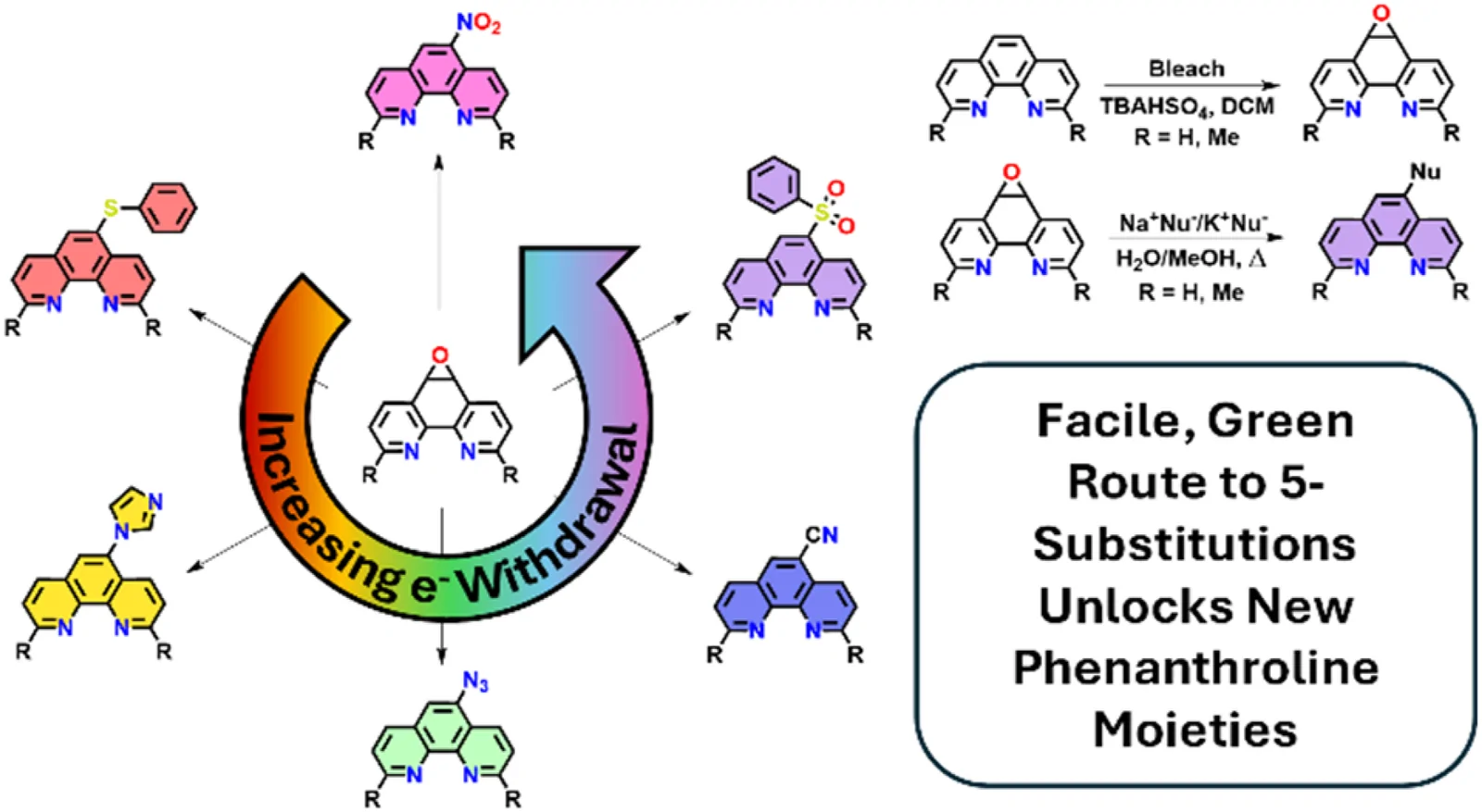

## Introduction

*N*-heterocyclic ligands are ubiquitous within inorganic and organometallic chemistry due to their ability to readily form coordination complexes with cationic transition metals.^1–7^ Amongst the most common *N*-heterocyclic ligands are diimine compounds such as 2,2′-bipyridine (bpy) or 1,10-phenanthroline (phen).^1,3,8,9^ Transition-metal complexes featuring bpy ligands are perhaps the best-studied molecules in the fields of photoredox catalysis, photophysics, and inorganic chemistry.^10–18^ They are synthetically facile and accessible while offering an array of desirable photophysical properties.^13,19–28^ While not as common as bpy, phen ligands are still ubiquitous and offer increased rigidity, reducing the impact of nonradiative relaxation pathways that are exceedingly abundant in cationic metal complexes with bpy.^29–32^ The empty π* orbitals of phen are low-lying enough in energy to interact with the d_*xy*_, d_*xz*_, and d_*yz*_ orbitals, which form the t_2g_ set in octahedral symmetry (*O*_h_), resulting in greater stability compared to complexes of bpy ligands. Phen ligands have been readily derivatized, yielding an extensive library of compounds in the published literature with interesting properties and unexplored, yet easily conceivable downstream products.^32–46^

Symmetric and unsymmetric functionalisations of the 2- and 9-positions of phen are very common and have been extensively studied within Cu(i) MLCT photophysics.^32,47–51^ Symmetric functionalisation of the 3-, 4-, 7-, and 8-positions is also well documented, although unsymmetric derivatisation at these positions is rarer.^4,32,45,46,52–55^ The symmetric substitution of the 5- and 6-positions is also known, but unsymmetric 5- and 6-position functionalisation is rare and relies upon a handful of reactions that employ a series of highly toxic chemicals and expensive reaction conditions (high temperature and pressure), making them broadly impractical for industrial utilisation.^32,56–60^ Moreover, these 5-substituted phen derivatives are often prohibitively expensive or unavailable in most instances. Development of greener, energy-conscious synthetic routes to these ligands would represent a significant accomplishment toward a greater understanding of these understudied phen derivatives, enabling access to a variety of valuable chemistries.

Shen and Sullivan demonstrated a method for readily producing epoxyphen from 1,10-phenanthroline and commercial bleach under pH-controlled conditions.^33^ The epoxyphen scaffold has since been most commonly used to access 5-carbonitrile-1,10-phenanthroline (5-CN-phen) by treating it with sodium cyanide in water.^61,62^ Downstream hydrolysis can be employed to form 1,10-phenanthroline-5-carboxylic acid (5-COOH-phen), an important functional group in surface anchoring chemistry and for accessing other specific functionalisations.^63^ Preparation of secondary amines and 5-methoxy-1,10-phenanthroline has also been attempted, but these are generally accessed *via* a 6-hydroxyl-substituted, dearomatized intermediate or through wholly separate synthetic strategies.^64,65^ Treatment with sodium hydride is sufficient to drive the elimination of the hydroxyl group, resulting in aromatisation.^33,64,66^ Other derivatives, such as 5-bromo-1,10-phenanthroline (5-Br-phen) and 1,10-phenanthroline-5-ol (5-HO-phen), have been reported; the preparation of 5-Br-phen requires heating oleum and Br_2_ to 150 °C in a pressure tube with phen.^67,68^ Meanwhile, the 5-HO-phen preparation is an acid-catalyzed method that uses sulfuric acid to facilitate the elimination reaction.^69,70^

Functionalisation of the 5-position of 2,9-dimethyl-1,10-phenanthroline (dmp) is quite rare within the greater phenanthroline synthetic literature, this rarity originates from increased synthetic challenges posed by dmp as compared to phen. This synthetic difficulty originates from a combination of increased steric hindrance created by the 2,9-dimethyl substitutions and the deactivation of the phenanthroline backbone by the electron-donating (+I) methyl groups resulting in sluggish reactions and deleterious side reactions.^31,32,71^ That aside, 5-bromo-2,9-dimethyl-1,10-phenanthroline (5-Br-dmp) and 5-nitro-2,9-dimethyl-1,10-phenanthroline (5-NO_2_-dmp) have been accessed through electrophilic substitution.^72–76^ 5-NO_2_-dmp can be reduced to form 5-amino-2,9-dimethyl-1,10-phenanthroline (5-NH_2_-dmp), which can be further modified for a variety of synthetically interesting applications.^76,77^

**Fig. 1:**
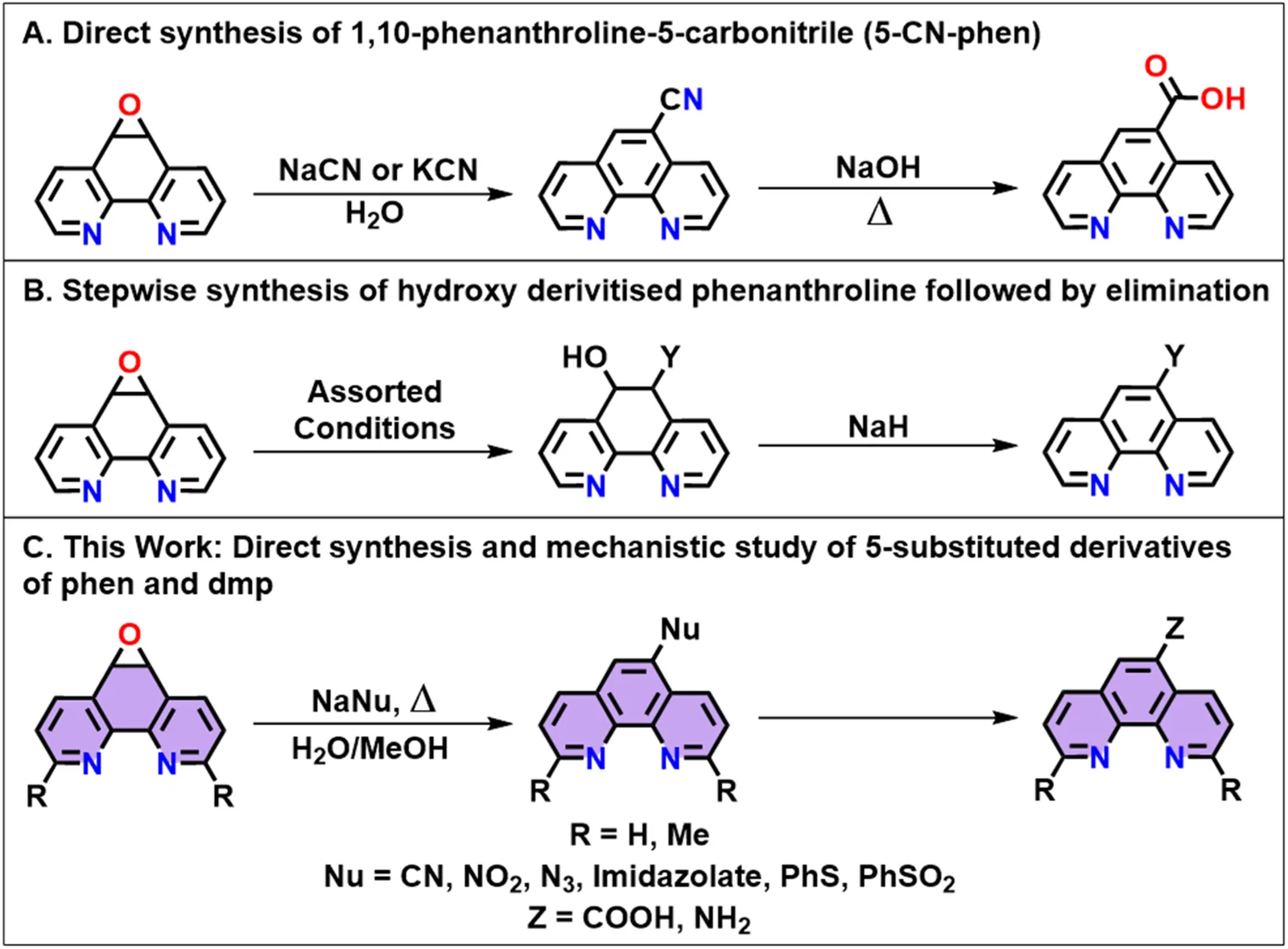
(A) Prior reports on the direct synthesis of 1,10-phenanthroline-5-carbonitrile from epoxyphen. (B) The preparation of 5-methoxy-1,10-phenanthroline and secondary amine derivatized phens by way of the 5-hydroxyl substituted, dearomatized intermediate. (C) This work presents the direct synthesis of CN^−^, NO_2_^−^, N_3_^−^, imidazolate, thiophenylate, and benzenesulfinate derivatized phen and dmp directly from their epoxy precursors. These ligands can be used in numerous downstream reactions.

In this work, we report the preparation of phen and dmp ligands functionalized with NO_2_^−^, N_3_^−^, and CN^−^ groups at the 5-position *via* the epoxyphen route (Fig. 1). The necessary conditions for efficient conversion of epoxyphen and epoxydmp to these derivatives are also investigated. The mechanism of this conversion, we propose, begins with ring opening of the epoxide *via* nucleophilic substitution, leading to the formation of the hydroxyl intermediate. Elimination of the hydroxyl group of the intermediate 5-hydroxyl-6-Nu-phen species re-aromatizes the phenanthroline backbone, yielding the final products. A greater understanding of the proposed mechanism was obtained both through the successful synthesis of the additional thiophenylate (PhS^−^), imidazolate (Im^−^), and benzenesulfinate (PhSO_2_^−^) derivatives and the failed attempts to produce derivatives featuring electron donating or sterically bulky moieties. These findings have allowed us to carefully map out the conditions under which this one-pot method is most advantageous. In the successful cases, phen derivatives were obtained in high purity by vacuum filtration, while the dmp derivatives required only a minimal organic workup procedure.

## Experimental

### General

All starting materials were purchased from commercial vendors (Sigma-Aldrich, VWR, Thermo Fisher, TCI, and Ambeed) and used immediately without further purification. Hypochlorite was obtained from commercial bleach. Several varieties of commercial bleach were used, but we observed the best yields for the preparation of epoxyphen from phen when LA's Awesome Bleach® was used; this bleach was obtained from a local dollar store. Reaction rates and product formation were monitored with TLC and NMR. Structural characterisation of the resultant derivatized phen and dmp ligands was accomplished with ^1^H NMR, ^13^C NMR, MALDI-TOF HR-MS, and ATR-IR spectroscopy. ^1^H and ^13^C NMR measurements were performed on a Bruker Avance 400 MHz spectrometer; all NMR spectra were processed in MestReNova 10.0. ESI HR-MS measurements were performed with on a ThermoFisher Exactive Plus interfaced with an Ultimate-3000 uHPLC system. All samples were dissolved in dichloromethane and diluted in acetone before a 20 µL aliquot was injected into the spectrometer and eluted with a 10% water, 0.1% formic acid, and 90% MeOH solvent system. All compounds were ionized by electrospray operating in positive mode. ATR-IR spectroscopy was collected with a Bruker ALPHA Platinum-ATR on solid-state samples.

### General preparation of epoxyphen and epoxydmp

Procedures reporting this process have been used for both phen and dmp.^33,78^ The desired phenanthroline ligand was dissolved in dichloromethane and treated with commercial bleach and TBAHSO_4_. The organic and aqueous layers were emulsified by vigorous stirring (>800 rpm) with a large magnetic stir bar (∼7 cm in length, 1 cm in width); the reaction pH was maintained between 8.2 and 8.6 for 2 to 8 hours. The epoxidized material was collected from the DCM following a straightforward workup, yielding both epoxyphen and epoxydmp in good yields. Exact parameters which we employed are available within the supporting information.

### General preparation of 5-Nu-phen derivatives

Multiple preparation methods were investigated for each phen derivative; these are provided in the SI. Epoxyphen was dissolved in deionized water and/or methanol. The corresponding sodium salt was added in 10-fold excess, and the reaction was heated to 70 °C overnight while stirring. The next day, a precipitate had formed. This material was collected, washed with water, and dried, affording pure 5-Nu-phen in moderate to good yields. Additional details are available in the SI.

### General preparation of 5-Nu-dmp derivatives

Epoxydmp was dissolved in methanol, while the corresponding sodium salt was dissolved in deionized water before being added to the methanolic mixture. The reaction was stirred overnight. The next day, the solvent was removed *in vacuo*. Purification by plug-column chromatography was generally sufficient to obtain the dmp derivative in moderate to good yields and with high purity. Additional details are available in the SI.

### Synthesis of downstream derivatives and byproducts

The synthesis of other interesting 5-functionalized derivatives of phen and dmp was carried out using known synthetic methods. The details for these procedures are provided in the SI.

## Results and discussion

**Scheme 1:**
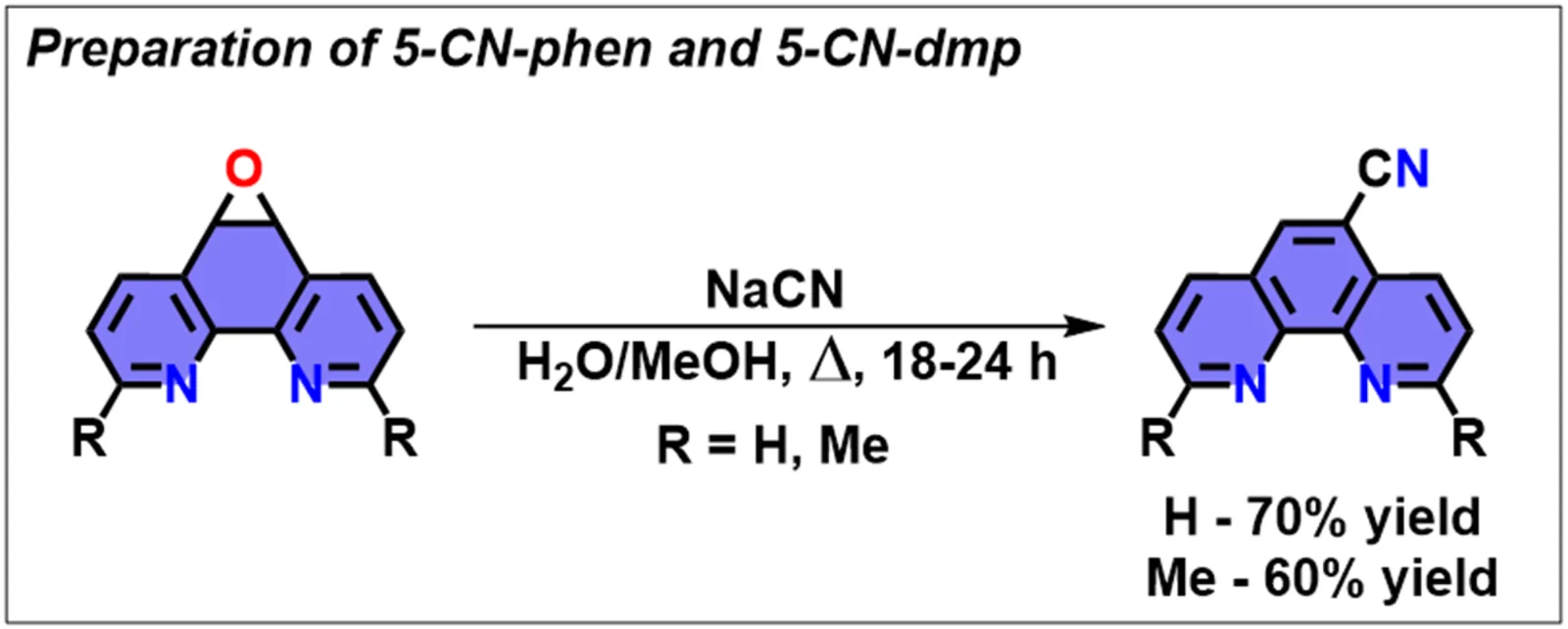
The conditions employed in the synthesis of carbonitrile derivatives of phen and dmp.

Phen has been extensively functionalized previously, including the 5- and 6-positions. Generally, the preparation of 5-substituted phen derivatives has used electrophilic substitution, but the development of the epoxyphen method provided an alternative route. Epoxyphen has become a key intermediate in the preparation of 5-CN-phen, 5-OH-phen, and 5-methoxy-1, 10-phenanthroline (5-MeO-phen). 5-CN-phen has been prepared directly by treating epoxyphen with sodium cyanide in deionized water (Scheme 1). The other derivatives have been synthesized utilizing a base-catalyzed mechanism. Epoxydmp has also been prepared; however, preparation of downstream derivatives, such as 5-CN-dmp, has not been described in an adjacent procedure similar to that for 5-CN-phen.

The preparation of epoxydmp is notably different from that of epoxyphen. Despite the structural similarity, the formation of epoxydmp is not kinetically favorable to the degree that epoxyphen formation is. Additionally, epoxydmp and dmp have nearly identical retention factors (*R*_f_), making it impractical to monitor reaction progress by thin-layer chromatography (TLC). ^1^H NMR spectroscopy was used instead to monitor the reaction to form epoxydmp. Interestingly, hypochlorite concentration affected the reaction rate in the epoxyphen reaction but less so in the formation of epoxydmp. Epoxyphen reactions generally were completed within 2 hours; higher concentrations of hypochlorite would further reduce the reaction time. In the case of epoxydmp, reactions always occurred on a timescale of 4–12 hours; the reaction time was invariant to hypochlorite concentration. After the epoxy reaction was complete, the organic layers were collected, and the volume was reduced. This high-concentration solution was poured into diethyl ether, forming a precipitate. The was filtered, the filtrate collected, washed with water, dried over sodium sulfate, and the solvent removed by rotary evaporation. Epoxydmp was obtained as a tan solid, which was not purified further before use.

Given the wealth of precedent literature on the preparation of 5-CN-phen from epoxyphen, we began our studies by replicating the preparation of 5-CN-phen and by developing a generalized procedure to prepare 5-CN-dmp from epoxydmp. The former was prepared in aqueous conditions and obtained in good yields. These conditions were manipulated in several ways, including altering the solvent, increasing the reaction scale, and raising the temperature. The addition of methanol to the solvent mixture had a minimal impact (1%) on the observed yield (70%). The reaction was also tolerant of a 10-fold increase in the reaction scale; the overall yield remained unchanged. Increasing the temperature from room temperature to 70 °C did yield a small increase in the reaction yield from 63% to 70% (Table 1).

**Table 1:** The variations of different reaction conditions for the preparation of 5-CN-phen^a^

Entry	Solvent	Temperature (°C)	NaCN (mmol)	Yield (%)
1	H_2_O	25	10	63
2	H_2_O	60	10	69
3	MeOH	60	10	70

a Full descriptions of these conditions are available within the SI.

These observations were subsequently applied to the corresponding reaction with epoxydmp; to our knowledge, a preparation of 5-CN-dmp from epoxydmp has not been described. This is ascribed to the limited solubility of epoxydmp in water. This issue was addressed by adding methanol to the solvent environment, as previous observations with epoxyphen showed no meaningful difference in reaction yield when methanol was used in place of water. Epoxydmp readily dissolved in methanol; sodium cyanide was dissolved in water and added to the mixture. The methanol-to-water ratio needed to be roughly 4 : 1, as too much water would precipitate epoxydmp from solution, whereas too little water would insufficiently solubilize the sodium cyanide. The reaction proceeded in much the same way as the analogous epoxyphen-to-5-CN-phen reaction. Once completed, the addition of a large excess of water precipitated 5-CN-dmp from solution; this precipitate was collected by vacuum filtration and found by ^1^H NMR to be pure 5-CN-dmp.

**Scheme 2:**
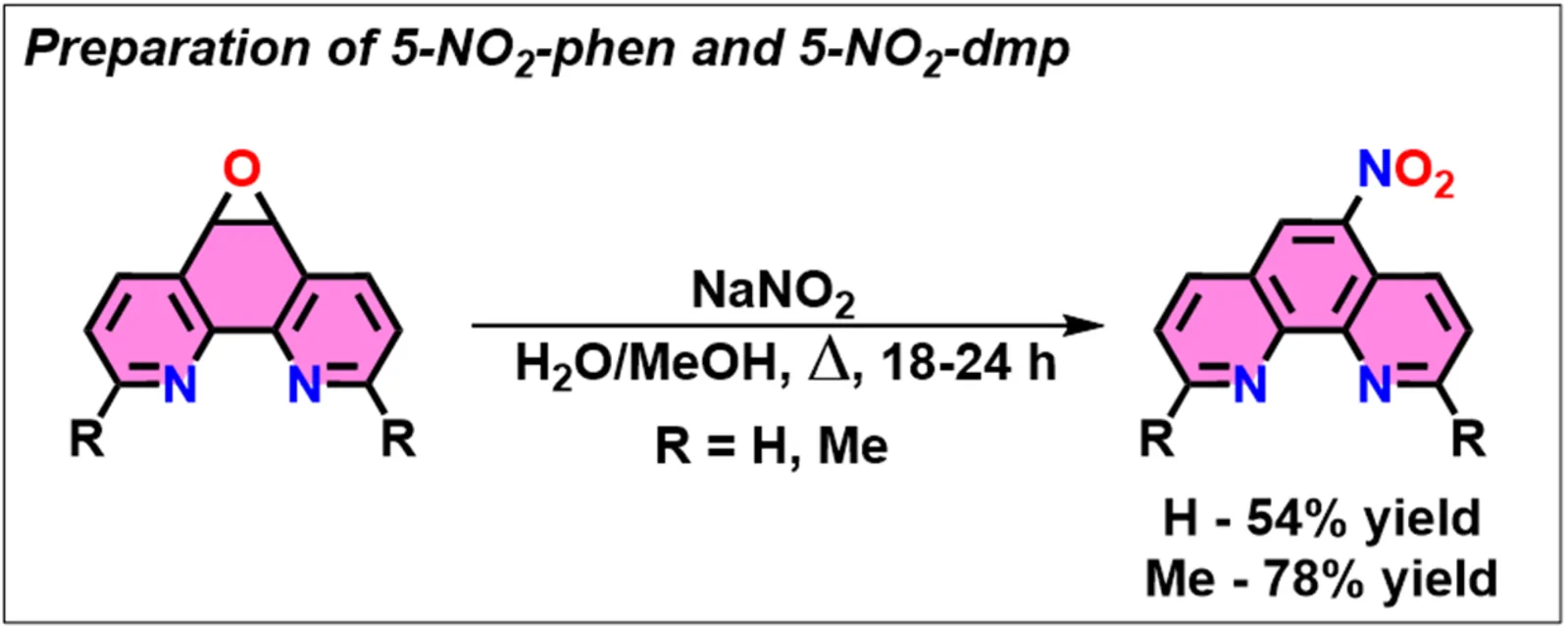
The conditions employed in the synthesis of nitro derivatives of phen and dmp.

We sought to gain a greater understanding of this reaction mechanism, which had been insufficiently described at the time of writing. The preparation of nitro derivatives represented an advantageous intermediate to access, as 5-NO_2_-phen is a key intermediate in the preparation of 5-amino-1,10-phenanthroline (5-NH_2_-phen). Prior reports on the formation of 5-NO_2_ derivatives on phen and dmp have utilized nitric acid and sulfuric acid heated to 120 °C to facilitate nitration.^79,80^ Meanwhile, we were able to maintain conditions which were largely unchanged from the preparation of 5-CN-phen and 5-CN-dmp from epoxyphen and epoxydmp. To emulate the procedure for 5-CN-phen, sodium nitrite was chosen as the nitration reagent (Scheme 2). Initial reactions were performed at room temperature on a 1 mmol scale using epoxyphen. Similarly to the 5-CN-phen reaction, a precipitate formed following an overnight reaction. However, the yield of 5-NO_2_-phen was lacking at only 16%. A substantial temperature dependence was observed as heating the reaction to 70 °C overnight increased the yield of 5-NO_2_-phen to 54% (Table 2). The reaction responded well to both the addition of methanol and an increase in the mole basis of the reaction (45% yield). Maintaining water in the solvent mixture was important – reactions in methanol alone yielded only 27%. Yields beyond 48–54% were difficult to attain; however, reactions were not heated beyond 70 °C, and the thermal dependence could be further exploited.

**Table 2:** The variations of different reaction conditions for the preparation of 5-NO_2_-phen^a^

Entry	Solvent	Temperature (°C)	NaNO_2_ (mmol)	Yield (%)
1	H_2_O	25	10	16
2	H_2_O	70	10	54
3	MeOH	60	10	27
4	MeOH : H_2_O (1 : 1.5 v/v)	70	51^b^	45
5	H_2_O	60	100^b^	48

a Full descriptions of these conditions are available within the SI.

b These reactions were scaled by a factor of 5.1 and 10, respectively.

The 5-NO_2_-dmp precursor was prepared using the same methanol/water mixtures as for 5-CN-dmp (Scheme 2). In this case, a 1 : 6 methanol-to-water mixture yielded the best results. This reaction was stirred and heated overnight, then a large excess of water was added to facilitate precipitation of the product. This was not necessarily instantaneous and would form after roughly one hour. The precipitate was isolated and identified as 5-NO_2_-dmp by ^1^H-NMR. Interestingly, the yield of the 5-NO_2_-dmp reaction was higher, at 78%, than that of the 5-NO_2_-phen reactions (Scheme 2). We suspect that yields of the dmp derivatives are primarily a function of the methanol/water solvent mixture rather than temperature as in the case of the phen derivatives.

**Scheme 3:**
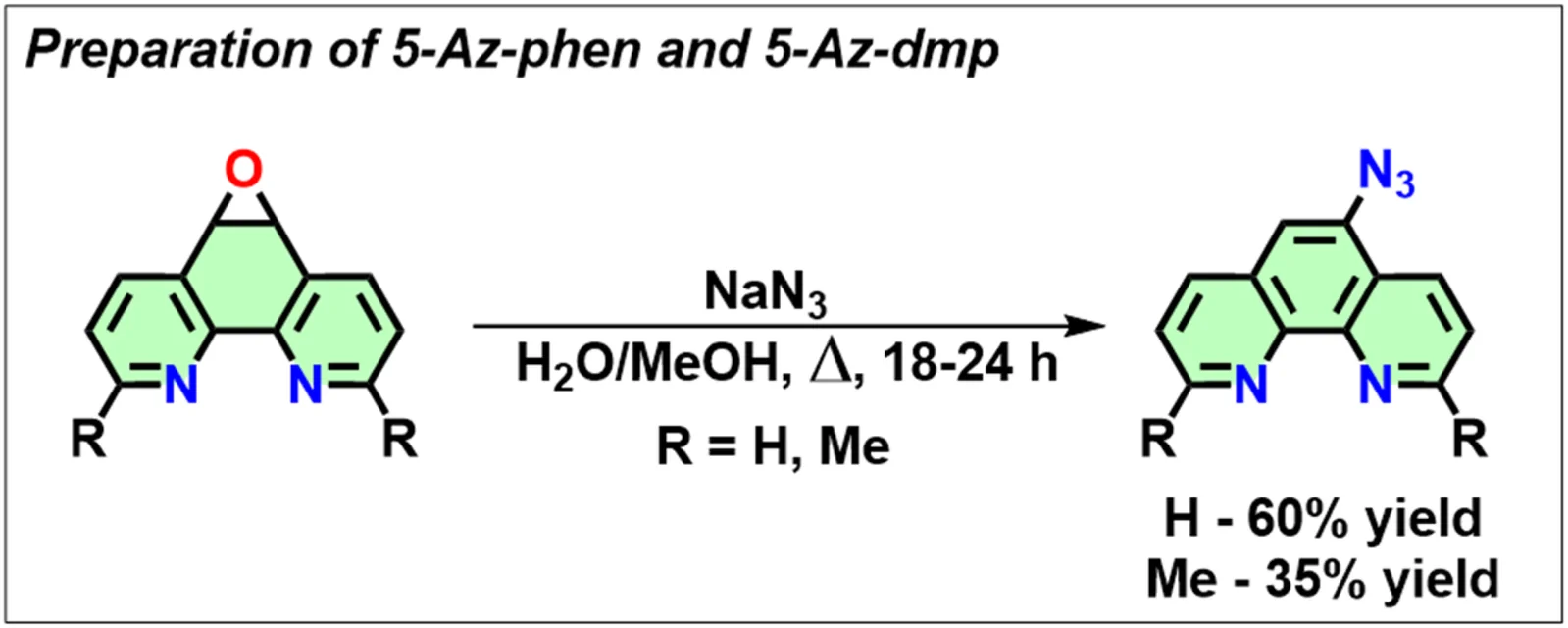
The reaction conditions employed in the synthesis of azido derivatives of phen and dmp.

The final primary functionalisation we considered was azides at the 5-position of phen and dmp (Scheme 3). 5-Azido-1,10-phenanthroline (5-Az-phen) has been prepared *via* a two-step route that yields 1,10-phenanthrolin-5-ol-6-azido-5,6-dihydro-5-acetate as the key intermediate. Treatment with DBU forms the final product, 5-Az-phen. 5-Az-phen has been prepared from epoxyphen by Kellett and coworkers, who reported a quantitative yield.^60,81^ It is, however, improbable that this yield is quantitative, as it does not account for excess sodium azide, which we found could contaminate the final product. We found that treating epoxyphen with sodium azide in aqueous conditions produced 5-Az-phen in 60% yield (Table 3). Interestingly, the efficiency of this reaction increased dramatically when the mole basis of the reaction was increased. An initial reaction performed on a 1 mmol basis with respect to epoxyphen produced 5-Az-phen at a 27% yield. When the reaction was scaled up to 10 mmol of epoxyphen, the yield increased to 60% (Table 3). A similar temperature dependence was observed, with room-temperature yields substantially lower than those at 70 °C.

**Table 3:** The variations of different reaction conditions for the preparation of 5-Az-phen^a^

Entry	Solvent	Temperature (°C)	NaN_3_ (mmol)	Yield (%)
1	H_2_O	70	1	27
2	H_2_O	70	10	60

a Full descriptions of these conditions are available within the SI.

The dmp derivative, 5-Az-dmp, was prepared similarly to previously discussed dmp derivatives. A 4 : 1 methanol-to-water mixture was used again to solubilize both the epoxydmp and sodium azide. A more traditional workup was utilized as flash column chromatography was required to obtain the final product, 5-Az-dmp. The yield of 5-Az-dmp was lower than that of 5-CN-dmp and 5-NO_2_-dmp; optimisation of solvent conditions would likely improve the conversion rate.

### Mechanistic proposal

The preparation of 5-CN-phen and 5-Az-phen were the only reported synthetic procedures for a one-pot route to a 5-substitution on phen. Attempts to generalize this procedure to other functionalities led to the formation of the dearomatized, hydroxylated, and nucleophile-substituted phenanthrolines. This compound is formed upon nucleophilic attack on the C–O bond, leading to ring opening of the epoxide. The proposed second mechanistic step is the elimination of the intermediate hydroxyl group, with hydroxide as the leaving group. In our experience, elimination was facile, and no additional strong bases were required given that the nucleophile was not an especially good electron donor.

**Scheme 4:**
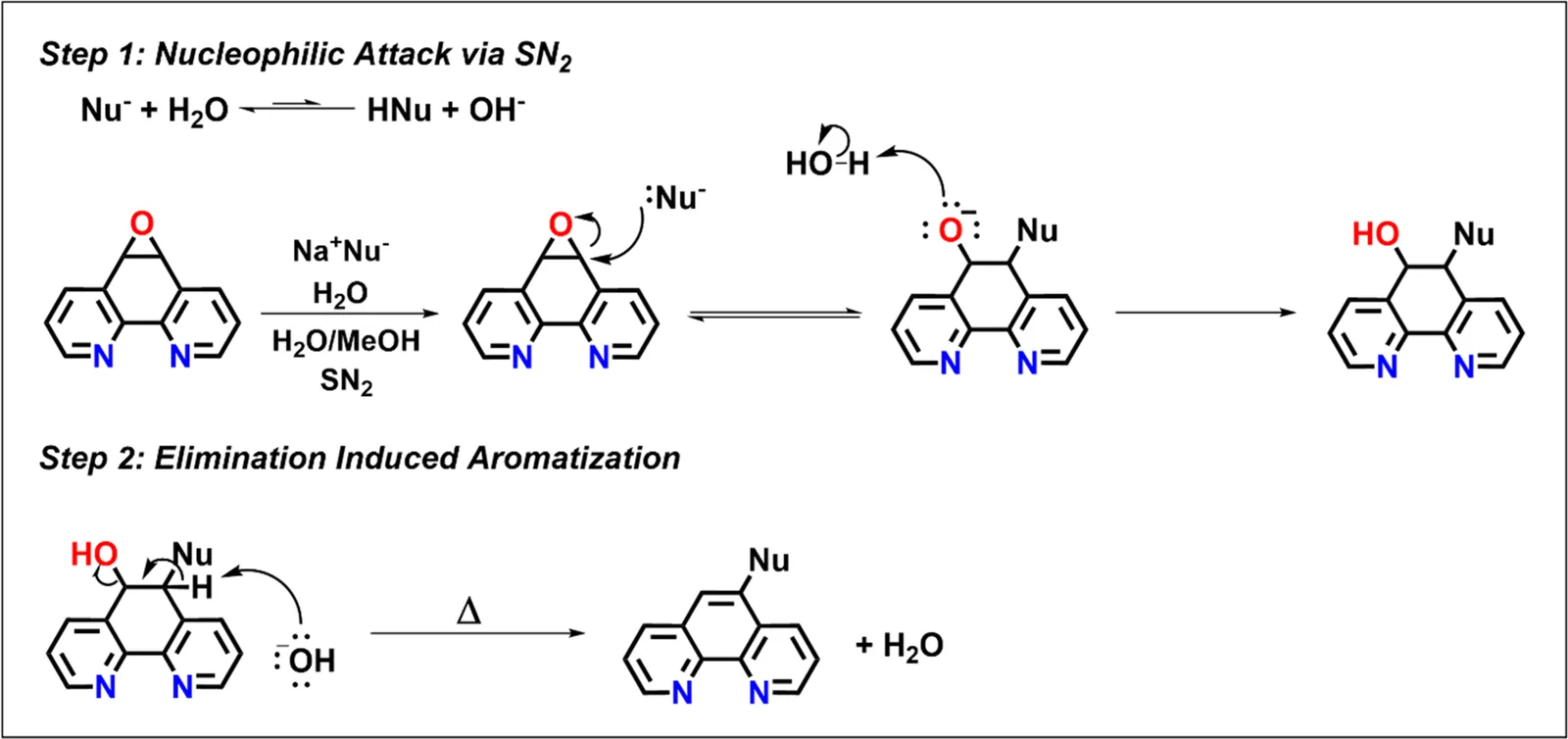
The proposed mechanism for the one-pot synthesis of 5-Nu-phen. Ring opening of epoxyphen occurs *via* an S_N_2-like mechanism upon interaction with Nu:^−^. The dissociation of NaNu facilitates the formation of OH^−^ in quantities proportional to the acid dissociation equilibrium constant (*K*_A_) of NaNu. Nucleophilicity dictates the efficiency of the ring opening. The efficiency of the second step depends on two factors: the concentration of hydroxide anions in solution and electron withdrawal by the now appended nucleophile. [OH^−^] can be modulated by varying salt concentration and/or temperature, leading to 5-substituted phenanthroline *via* an elimination mechanism.

Ring opening of arene oxides proceeds *via* an S_N_1- or an S_N_2 mechanism, depending on the solvent conditions (Scheme 4). The polar protic conditions used herein suggest that S_N_1 is the likely mechanism, given the increased stabilisation of the transient carbocation. However, CN^−^, NO_2_^−^, and N_3_^−^ are each the conjugate bases of weak acids. Additionally, prior reports have shown that the yields of the azidolysis of epoxycyclohexane are not meaningfully improved when using anhydrous acetonitrile.^82^ Hydrolysis of these conjugate bases within the aqueous solution results in the formation of the conjugate acid and a hydroxyl anion. This would suggest that the ring opening proceeds *via* an S_N_2-like mechanism to produce the corresponding 6-hydroxyphen or 6-hydroxy-dmp species (Scheme 4). There is a delicate balance required: the ring opening is highly dependent upon the synergy of the nucleophile being sufficient while being the conjugate base of a weak acid, such that small amounts of hydroxyl anions are produced. With this in mind, we conducted reactions with larger quantities of NaOH in solution. This, interestingly, led to the production of various byproducts. Other reports of the syntheses of 5-hydroxyl-6-Nu-phen utilize either a secondary amine or a thiol as nucleophiles in the presence of sodium methoxide. Moreover, based on our observations and previously reported basic conditions, we propose that epoxide ring opening occurs *via* an S_N_2-like mechanism. This process is thermodynamically favorable, driven by a loss of ring strain of ∼27 kcal mol^−1^.^83^

Once the 5-hydroxyl-6-Nu-phen species is formed, elimination of the hydroxyl group followed by aromatisation is observed (Scheme 4). This involves deprotonation of the Nu-substituted carbon, facilitating the efficient formation of the π-bond between carbons 5 and 6, which subsequently eliminates the hydroxyl group. During the synthesis of 5-Az-phen, it was noted that isolation of HON_3_-phen, followed by its resuspension and heating in water overnight, failed to yield 5-Az-phen. Small aliquots of dilute sulfuric acid were added to promote elimination by an E1 mechanism, but these also failed. Thus, it was concluded that the byproducts formed *in situ* from the initial nucleophilic attack or solvation of the salt in aqueous conditions were responsible for the elimination and aromatisation. As noted previously, small quantities of hydroxyl anions are formed through the equilibration of the intermediate alkoxide. The efficiency of the elimination step in these reactions appears to depend on p*K*_a_ (Table 4). For example, when using excess NaCN in the preparation of 5-CN-phen, a yield of 65% ± 5% was observed regardless of the temperature of the reaction. For NaNO_2_ and NaN_3_, their p*K*_a_ values are ∼3.4 and ∼4.7, respectively. Not only were the yields of the reactions with these nucleophiles lower than CN^−^, but additional thermal energy was observed to dramatically improve the efficiency of this second mechanistic step. It should be noted that elimination reactions are favored at higher temperatures. The modest change in the yield of 5-CN-phen and 5-CN-dmp formation suggests that large enough quantities of hydroxyl anions are formed *via* the hydrolysis of CN^−^ that an increase in the thermal energy of the system has a small impact on the overall [OH^−^]. Meanwhile, in the case of NO_2_^−^ and N_3_^−^, the formation of hydroxyl anions is sluggish enough that modest heating is sufficient to dramatically improve the rate of the elimination/aromatisation mechanistic step.

**Table 4:** The p*K*_a_ values of the conjugate acids of NaNO_2_, NaN_3_, and NaCN and yields of each reaction with epoxyphen

Salt	Conj. acid p*K*_a_ (25 °C)	Room temp. % yield	>70 °C % yield
NaNO_2_	3.2	16	54
NaN_3_	4.7	27	60
NaCN	9.3	63	69

To assess the veracity of this hypothesis, we initially used excess sodium nitrite and compared the room-temperature yields with those from the elevated-temperature reaction. Unsurprisingly, the reaction yields at elevated temperatures were observed to increase by a factor of three compared to those at room temperature. Varied concentrations of NaNO_2_ also supported this hypothesis, as Le Châtelier's principle and the van 't Hoff equation dictate that, if dissolution of a given system is exothermic, increasing the temperature will increase the dissociation constant. Additionally, an increase in reactant concentration shifts the reaction equilibrium toward more products, potentially explaining the increased yields as nucleophile concentration increased, since E2 reaction rates are proportional to the concentration of base in solution.

To increase our understanding of this one-pot synthesis of the 5-substituted phen and dmp derivatives, we expanded the scope of the nucleophiles we were considering – specifically to salts whose anions were the conjugate bases of strong acids. Given the near-unity *K*_A_ associated with these acids, we hypothesized that no hydroxide would form in solution and that limited reactivity would be observed. As anticipated, NaF, NaCl, NaBr, and NaI each yielded no measurable reaction. Interestingly, F^−^, while not the conjugate base of a strong acid, still showed no meaningful reactivity. This was attributed to F^−^ undergoing facile H-bonding with the polar, protic solvent, effectively forming a shell around the F^−^, hampering any possibility of reactivity.

An important consideration deserves acknowledgement within the context of this final elimination re-aromatisation reaction – the acidity of the proton found at the 6-position on the key 5-hydroxyl-6-Nu intermediate. Because OH^−^ is responsible for the deprotonation of this proton and the subsequent elimination of OH^−^ forming the final product, the p*K*_a_ of this proton directly impacts the rate at which elimination occurs. When the reaction to prepare 5-CN-phen was discovered by Shen and Sullivan, the electron withdrawing character of the cyanide group was likely not considered a crucial factor in the success of the reaction. Similarly, when Kellet and coworkers demonstrated the analogous reaction to produce 5-Az-phen, the electron withdrawing character of azide was likely overlooked as well. Similarly, we initially overlooked its importance, but upon closer examination of these three nucleophiles, one should notice that they each possess the propensity to withdraw electron density from the phenanthroline system and subsequently lower the p*K*_a_ of the proton at the 6-position of the 5-hydroxyl-6-Nu intermediates. Thus, we sought to more fully characterize this behaviour by expanding the scope of this reaction, including both electron withdrawing nucleophiles as well as some which we anticipated would fail due to excessive electron donation into the phenanthroline system. This insight underscores the importance of introducing strong bases into these reactions when nucleophiles of alkoxides or amides have ever been directly substituted onto epoxyphen. This reaction will naturally stop at the intermediate in the case of these electron donating nucleophiles necessitating the addition of bases like NaH to facilitate deprotonation and formation of the final 5-MeO-phen or 5-NMe_2_-phen derivatives.

### Expansion of nucleophile scope

**Scheme 5:**
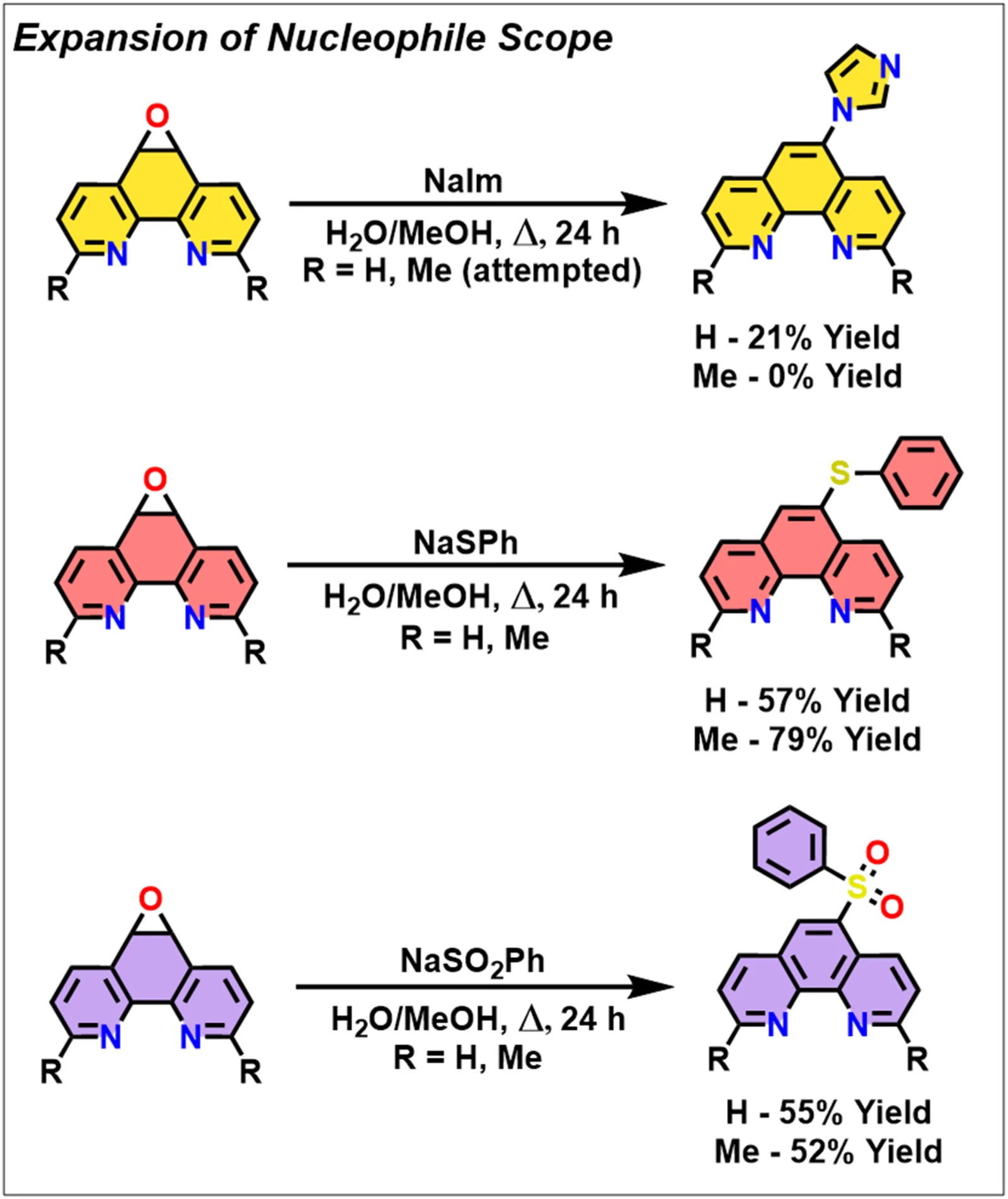
The scope of nucleophiles deployed to increase mechanistic understanding of the one-pot reaction.

**Scheme 6:**
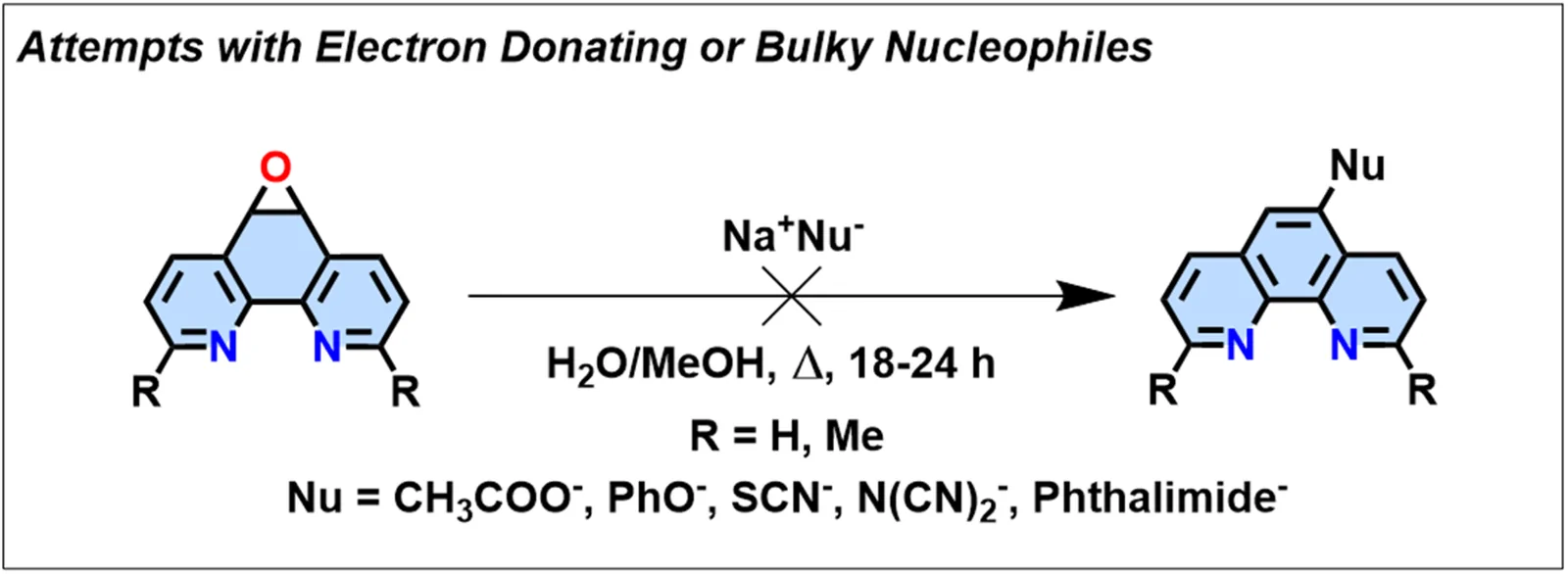
Unsuccessful nucleophilic substitutions which utilized the one-pot synthetic strategy.

To guide our expansion of the nucleophile scope, we consulted precedent literature reports which demonstrated similar reactions, albeit, with different parameters and methodologies. Prior reports have demonstrated the substitution of sulfanyl groups at the 5-position of phenanthroline utilizing NaOEt as a base additive. Therefore, we chose sodium thiophenylate (NaPhS) as it appeared amenable to our conditions (Scheme 5). To evaluate the importance basicity, we also considered sodium imidazolate (NaIm) (p*K*_a_ ∼14.3) within our expanded scope of nucleophiles (Scheme 5). We evaluated additional electron withdrawing groups such benzenesulfinate and dicyanamide to underscore the importance of electron withdrawing character to this one-pot method (Scheme 5 & 6). Analogous entries with electron donating groups were considered, however, these were less successful and are discussed later.

The preparation of 5-(1*H*-imidazole-1-yl)-1,10-phenanthroline (5-Im-phen) was performed in accordance with all other functionalisations of epoxyphen discussed herein. Following an overnight reaction, the reaction mixture was removed from heat, at which point a white precipitate formed, which was collected and identified as 5-Im-phen by ^1^H NMR. The yield was lower than expected (∼21%), given that imidazolate has a p*K*_a_ of 14.3, making it similarly basic to dilute sodium hydroxide. This was rationalized by the bulkier nature of imidazolate compared to the previously studied azide, nitrile, and nitro groups. Steric bulk is known to hamper S_N_2 reaction rates, suggesting that the ring-opening step of the mechanism is substantially hindered by bulkier substituents. Bolstering this hypothesis was the inability to isolate the hydroxyl intermediate, suggesting that the second elimination step occurs more rapidly due to the basicity of the imidazolate anion, forming large quantities of OH^−^*in situ*.

Meanwhile, 5-(1*H*-imidazole-1-yl)-2,9-dimethyl-1,10-phenanthroline was prepared according to the established procedure used for each of the aforementioned epoxydmp reactions. Solubility of the NaIm was somewhat limited in smaller volumes of water; the reaction volumes were scaled by a factor of 1.5 to compensate. This reaction was less successful than the other dmp reactions previously reported. Epoxydmp was not recovered from the reaction, and the ^1^H NMR spectrum of the recovered material was indecipherable, suggesting a significant propensity for epoxydmp to undergo deleterious side reactions with NaIm.

Functionalisation with thiolates was investigated with sodium thiophenylate (NaSPh) as a representative example. NaSPh was found to be poorly soluble in water to the extent that it never fully dissolved during the reaction with epoxyphen. Despite this poor water solubility, a heavier-than-water oil formed within the reaction mixture. This oil solidified upon cooling to room temperature. The oil was dissolved in dichloromethane, washed with water, and purified by flash column chromatography, yielding 5-(phenylthio)-1,10-phenanthroline (5-PhS-phen). Full purification of 5-PhS-phen was challenging, as PhS^−^ is a reasonable leaving group, resulting in the recovery of 1,10-phenanthroline as a minor product. Fortunately, 5-PhS-phen represented the bulk of the material obtained without the addition of base. To obtain a fully pure product, the reaction temperature was lowered (*ca.* 40 °C) to reduce *k*_b_*T* within the system. The analogous reaction was carried out with epoxydmp and full conversion to the product, 2,9-dimethyl-5-(phenylthiol)-1,10-phenanthroline (5-PhS-dmp) was observed in good yield (79%). Additionally, formation of dmp as a byproduct was observed at a substantially lower rate than the analogous unsubstituted phen derivative.

The last moiety we demonstrated was addition of arylsulfinates with us selecting benzenesulfinate as the substrate of interest. Following the previously established method for the other nucleophiles, through treatment of 1 equivalent of epoxyphen with 10 eq. of sodium benzenesulfinate (NaPhSO_2_), we observed formation of solid 5-PhSO_2_-phen which was subsequently collected as pure material by vacuum filtration in moderate yields (55% yield). The analogous reaction with epoxydmp was also successful. Following a similar purification process to that which we describe for the other 5-Nu-dmp derivatives, 5-PhSO_2_-dmp was isolated in a 52% yield. Unlike in the PhS derivatives, PhSO_2_ is a worse leaving group and isolation of phen or dmp was not observed.

### Unsuccessful nucleophiles

To enrich our understanding of proper use cases for this methodology, we needed to examine boundary conditions under which performance of the reaction is marginal or even fails completely. As discussed previously, hydrolysis was a crucial consideration as salts such as NaBr or NaCl showed no reactivity under these conditions. Additionally, because of the proposed S_N_2-like ring-opening of epoxyphen or epoxydmp, sufficiently strong nucleophiles were necessary. As such, treatment of either epoxyphen or epoxydmp with sodium acetate (NaCH_3_COO) was insufficient to form the corresponding 5-CH_3_COO derivatives. Seemingly, no meaningful reaction was observed to occur as ^1^H NMR revealed primarily epoxyphen or epoxydmp signals.

We attempted these reactions with sodium phenoxide (NaOPh), the oxo-derivative of NaSPh. Because PhO^−^ is a better nucleophile than PhS^−^ while being similarly sterically bulky, facile conversion to the 5-OPh derivatives seemed probable. However, while the nucleophilic substitution occurred readily, elimination of the hydroxyl group from 5-hydroxyl-6-OPh-phen and 5-hydroxyl-6-OPh-dmp would not proceed. This reveals an interesting periodic trend as NaSPh worked reasonably well; likely since it was able to readily stabilize the negative charge on C6 when deprotonation occurs. The PhO^−^ group is sufficiently electron donating that it reduces the acidity of this proton which must be removed during the elimination step. Prior reports describing the formation of alkoxide and amide derivatives at the 5-position of phen each have utilized a stepwise approach wherein the elimination is facilitated by addition of NaH or other very strong bases in polar, aprotic solvents. Thus, we can suggest that our one-pot approach is amenable to strong nucleophiles which are electron withdrawing or very weakly donating. We further confirmed this through attempted reactions with sodium dicyanamide (NaN(CN)_2_). Dicyanamide is a poorer nucleophile, less electron withdrawing, and bulkier than sodium cyanide; these properties in conjunction serve to dramatically reduce the rate of both the S_N_2-like and elimination steps of this reaction. As such, we observed no conversion of any kind and merely recovered starting epoxyphen or epoxydmp.

A final consideration is the impact of steric hindrance on the performance of this reaction. In the cases of many of the nucleophiles we chose to examine initially, these groups were physically small or had an isolated nucleophilic site poised to form a chemical bond (in the case of PhS^−^ and PhSO_2_^−^). In the case of imidazolate however, we observed reduced conversion as compared to the other nucleophiles on epoxyphen and no conversion on epoxydmp. A clearer understanding of the poorer performance of imidazolate was not obtained until we examined potassium phthalimide within the conditions of this reaction. The phthalimide anion is structurally similar to the imidazolate anion in that each feature a nitrogen heteroatom embedded within a 5-membered ring – this nitrogen acts as the reactive site for the nucleophilic attack. Phthalimide also possesses carbonyl carbons α to the nitrogen heteroatom, each of which serve to further congest the region surrounding the nitrogen. As such, phthalimide serves as a more sterically hindered analogue to imidazolate and, rather unsurprisingly, fails to meaningfully react under these conditions with either epoxyphen or epoxydmp. Moreover, we hypothesize that substantially sterically hindered nucleophiles will struggle to react efficiently with epoxyphen or epoxydmp. The reduction in reaction rate as a function of the steric bulk of the nucleophile also verifies that this nucleophilic substitution does proceed *via* an S_N_2-like mechanism as S_N_1 reaction rates are not significantly impacted by nucleophile steric considerations.

### Preparation of downstream derivatives

**Scheme 7:**
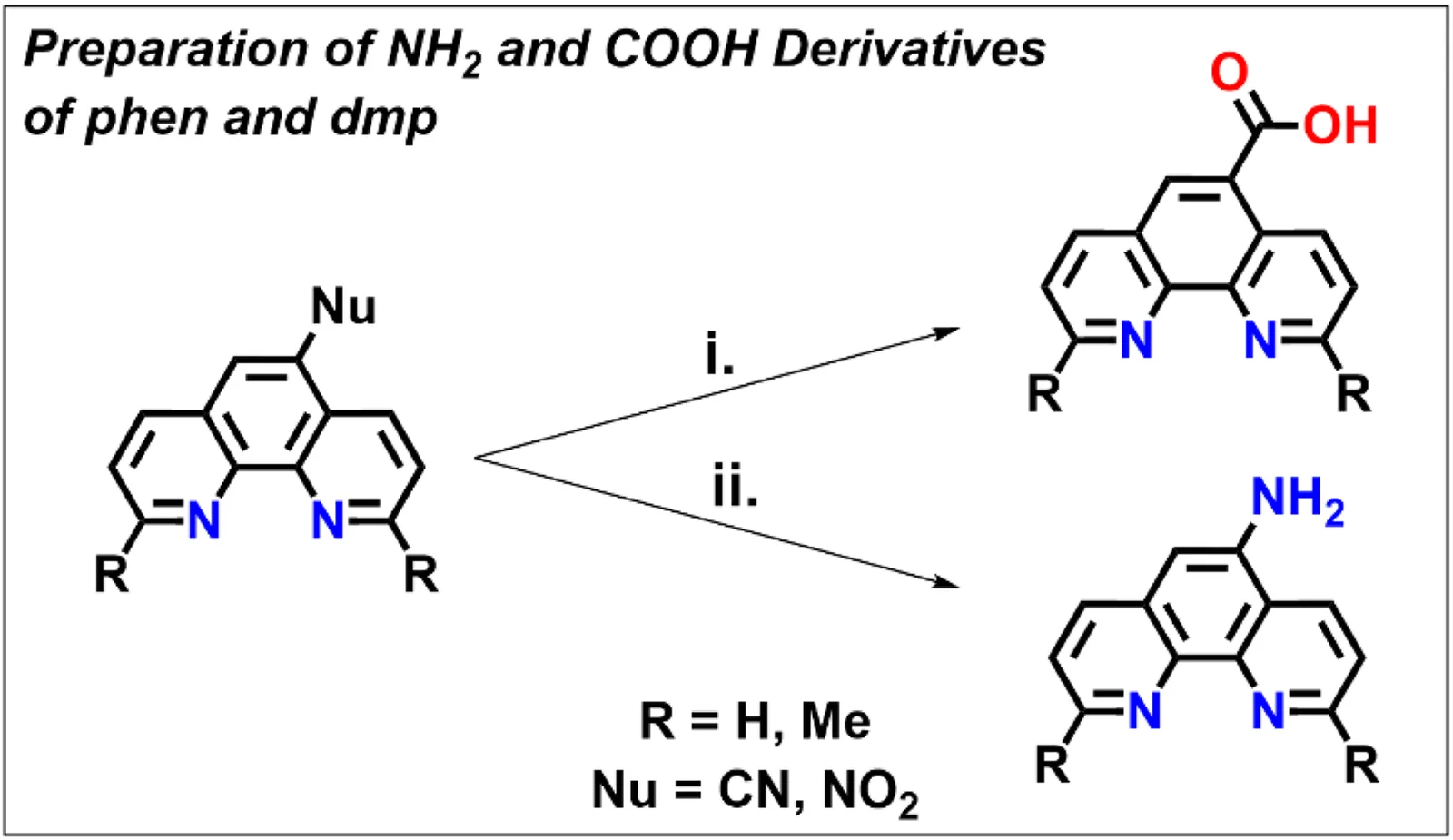
The preparation of carboxylic acid. ((i) NaOH (aq.), MeOH, HCl (for phen) or TFA and DCM (for dmp)) and amine (ii. Hydrazine hydrate, Pd/C, EtOH, 90 °C, 18 h) derivatives of phen and dmp from the nitro and carbonitrile derivatives prepared previously.

Each of the previously prepared materials represents a synthetically interesting intermediate that may be used to form useful derivatives. We demonstrated this by producing the 5-amino and 5-carboxylic acid derivatives of both phen and dmp (Scheme 7). Both 5-amino-1,10-phenanthroline (5-NH_2_-phen) and 1,10-phenanthroline-5-carboxylic acid (5-COOH-phen) have been prepared, with 5-COOH-phen produced from 5-CN-phen. 5-NH_2_-phen was produced from 5-NO_2_-phen *via* catalytic transfer hydrogenation with hydrazine monohydrate and palladium on carbon (Scheme 7). The corresponding compounds on dmp have never been reported to our knowledge. Preparation of these derivatives was accomplished *via* similar reaction methodologies. In the case of 5-amino-2,9-dimethyl-1,10-phenanthroline (5-NH_2_-dmp), identical conditions to those utilized in the preparation of 5-NH_2_-phen were suitable for the successful synthesis and isolation of 5-NH_2_-dmp (47% yield).

2,9-dimethyl-1,10-phenanthroline-5-carboxylic acid 5-COOH-dmp was prepared according to a different method than 5-COOH-phen. The primary reason for this methodological difference was the poor water solubility of 5-CN-dmp, which necessitated a MeOH/H_2_O solvent mixture in place of H_2_O alone in the case of 5-CN-phen. Upon treatment with NaOH, the sodium carboxylate intermediate (5-COO^−^Na^+^-dmp) formed. This material precipitated from solution, was washed, and isolated. Direct conversion to the carboxylic acid was achieved *via* treatment with acid (TFA); sonication of the solution improved the reaction rate and conversion to 5-COOH-dmp. The carboxylic acid derivative of phen has been used as a key intermediate in the synthesis of *N*-hydroxysuccinimide esters.^84^ These reactive materials have subsequently been used to prepare biologically active transition-metal complexes that interact with DNA and in other biochemical settings. Even without further derivatisation, the 5-COOH-phen has been utilized in a variety of biologically driven applications, suggesting that expanding the phenanthroline substrate scope to which this modality is effective represents an important synthetic observation.^42,85–88^ Meanwhile, amine derivatisations represent a crucial intermediate material to access amides, which may themselves be subjected to a variety of different chemical transformations.^89–91^ All told, the facile preparation of these different phenanthroline derivatives with key reactive functionalities at the 5-position enables the preparation of a wide range of novel phenanthrolines with diverse applications in inorganic, organic, and biochemistry.

## Conclusions

Herein, we have demonstrated a one-pot synthetic strategy to prepare derivatives of phen and dmp featuring functionalisation of the 5-position of the phenanthroline ligand. These strategies leverage the epoxyphen and epoxydmp motifs; when treated with strong, electron withdrawing nucleophiles, which are the conjugate bases of weak acids and solvated in polar, protic solvents, hydroxyl anions formed by hydrolysis of these nucleophiles result in an S_N_2-like ring opening of the epoxide, forming the 5-hydroxyl-6-Nu-phen or 5-hydroxyl-6-Nu-dmp intermediates. Then, a subsequent elimination reaction is catalyzed by the hydroxyl anions formed through the initial hydrolysis, resulting in deprotonation of the carbon to which the nucleophile is bonded. Aromatisation of the phenanthroline backbone, followed by elimination of the hydroxyl group, yields 5-Nu-phen and 5-Nu-dmp. The p*K*_a_ of the conjugate acid of each nucleophile appears to have a significant impact on the efficiency of the elimination step in the mechanism. Previously, only CN derivatives of phen were facile to produce due to the p*K*_a_ of HCN favoring formation of hydroxyl anions, which play an active role in both the S_N_2-like and elimination steps of this mechanism. Heating and super-stoichiometric loading of nucleophiles promoted the formation of more hydroxide in solution and favored efficient elimination, providing a more general synthetic route to 5-functionalized phen.

Addition of methanol to the solvent system enhanced the generalisation of this method to include dmp. This improved solubility of epoxydmp in methanol permitted access to several dmp derivatives, which are themselves valuable synthons in just two steps from a commercially available phenanthroline derivative. These key intermediates are important steppingstones to –COOH and –NH_2_ derivatives, which serve as building blocks for a variety of more specific functionalisations. Additionally, the azide functionality enables click-chemistry derivatisations. The generalisation of this chemistry to dmp from phen supports the hypothesis that, under the right conditions, it may be extended to other phenanthroline derivatives, such as 3,4,7,8-tetramethyl-1,10-phenanthroline (tmp), yielding a variety of interesting new applications for these 5-functionalized phenanthrolines. This reaction represents a powerful synthetic tool because of both its potential for generalisation to other phenanthroline derivatives and for new functionalisations at the 5-position, permitting a broad library of new ligand choices for transition-metal complexes in photosensitisation, catalysis, and biochemical applications.

## Author contributions

This manuscript was written through the contributions of all authors, with each author approving the final draft.

## Conflicts of interest

The authors have no financial conflicts of interest to declare.

## Supplementary Material

RA-OLF-D6RA04592H-s001

## Data Availability

The supplementary information (SI) contains substantial detail, including all raw molecular characterisation data. Supplementary information is available. See DOI: https://doi.org/10.1039/d6ra04592h.
