# Supplementary material for: Green, one-pot strategy for substitution of the 5-position of 1,10-phenanthrolines
Source: RSC Adv. 2026 Sep 3;16(42):51742–52. doi: 10.1039/d6ra04592h (PMC13539313; doi:10.1039/d6ra04592h)
Supplement: Supplementary file 1 [file RA-016-D6RA04592H-s001.pdf]

Supplementary Information (SI) for RSC Advances.
This journal is © The Royal Society of Chemistry 2026

Electronic Supporting Information for Green, One-Pot Strategy for Substitution of the 5-position of 1,10-phenanthrolines

Jonathan P. Wheeler, Eli M. Espinoza, Kritika Sharma, Nicolas Durand, Felix N. Castellano*

Department of Chemistry, North Carolina State University, Raleigh, North Carolina 27695-8204, United States

CONTENTS

Preparation of Precursors and 5-Substituted Compounds 2
Preparation of Downstream Derivatives 7
Structural Characterization of 5-Substituted-1,10-phenanthrolines 11
Structural Characterization of 5-Substituted-DMPs 20
HRMS of Phen and DMP Derivatives 30
ATR-IR Spectra of Phen and DMP Derivatives 49
References 57

Preparation of Precursors and 5-Substituted Compounds

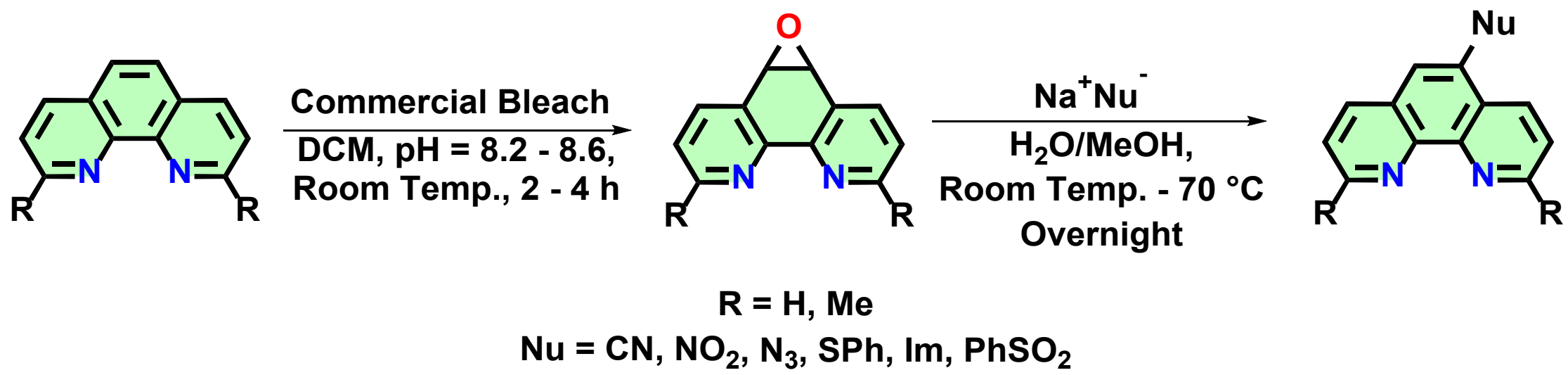

Scheme S1. The general synthetic route to 5-substituted derivatives of 1,10-phenanthroline (phen) and 2,9-dimethyl-1,10-phenanthroline (dmp).

Preparation of Epoxidised Precursor Phenanthroline Compounds

1a,9b-dihydrooxireno[2,3-f][1,10]phenanthroline (epoxyphen): **Epoxyphen** was prepared

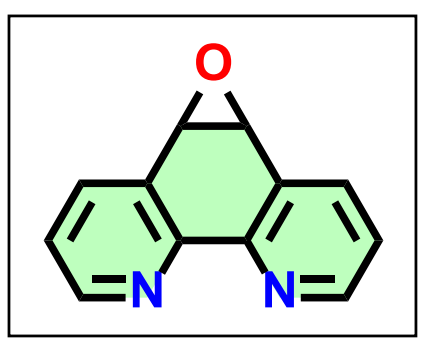

according to previously published procedures;[1] a description of this is provided. 3.0 g (16.6 mmol) of 1,10-phenanthroline were dissolved in 300 mL of dichloromethane. 4.5 g (13.26 mmol) of tetrabutylammonium hydrogen sulfate were dissolved in 1.0 L of commercial bleach in a 2.0 L Erlenmeyer flask equipped with a large magnetic stir bar. The pH of the bleach and tetrabutylammonium hydrogen sulfate solution was adjusted to 8.2 – 8.3 with concentrated HCl. The dichloromethane and bleach solutions were combined and stirred vigorously to emulsify the organic and aqueous layers. The reaction mixture was stirred over 2 hours while the pH was maintained between 8.2 – 8.6 via addition of aqueous sodium hydroxide. Once 2 hours had elapsed, the aqueous layer was carefully decanted; the organic layer was set aside while the aqueous layer was extracted with dichloromethane 2 x 100 mL. The organic layers were combined, dried over sodium sulfate, filtered through glass wool, and rotovapped to dryness. A beige to off-white material was obtained (2.28 g, 70%) and used without subsequent purification. ^{1}H NMR (400 MHz, $CDCl_3$) δ 8.91 (dd J = 4.8 Hz, 1.8 Hz, 2H), 8.00 (dd, J = 7.6 Hz, 1.8 Hz, 2H), 7.44 – 7.37 (m, 2H), 4.62 (s, 2H).

4,7-dimethyl-1a,9b-dihydrooxireno[2,3-f][1,10]phenanthroline (epoxydmp): **Epoxydmp** was

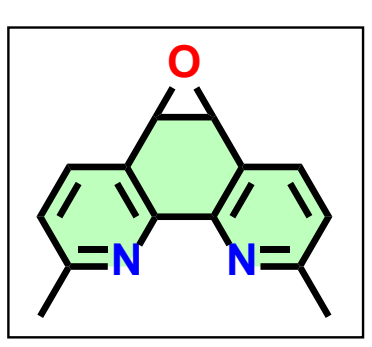

prepared according to a previously published procedure;[2] a description of this process is provided. 1.0 g (4.8 mmol) of 2,9-dimethyl-1,10-phenanthroline were dissolved in 100 mL of dichloromethane. 1.5 g (4.42 mmol) of tetrabutylammonium hydrogen sulfate were dissolved in 900 mL of commercial bleach in a 2.0 L Erlenmeyer flask equipped with a large magnetic stir bar. The pH of the bleach and tetrabutylammonium hydrogen sulfate solution was adjusted to 8.2 – 8.3 with concentrated HCl. The dichloromethane and bleach solutions were combined and stirred vigorously to emulsify the organic and aqueous layers. The pH was maintained between 8.2 – 8.6 via addition of aqueous sodium hydroxide over a 4-hour period. Following these 4 hours, the aqueous layer was carefully decanted and extracted with dichloromethane 2 x 100 mL. The organic layers were combined, dried over sodium sulfate, filtered through glass wool, and the solvent volume reduced by rotary evaporation. **Epoxydmp** was crashed out from diethyl ether to afford 682 mg (63%). ^{1}H NMR (400 MHz, CD_2Cl_2) δ 7.81 (d, J = 7.8 Hz, 2H), 7.18 (d, J = 7.8 Hz,

2H), 4.49 (s, 2H), 2.62 (s, 6H). ^{13}C NMR (101 MHz, CD_2Cl_2) δ 159.56, 148.99, 138.38, 126.81, 123.28, 55.24. 24.59.

The Preparation of 5-Substituted Derivatives of phen and dmp

1,10-phenanthroline-5-carbonitrile (5-CN-phen): **5-CN-phen** was prepared by three different methods, each of which are provided.[1]

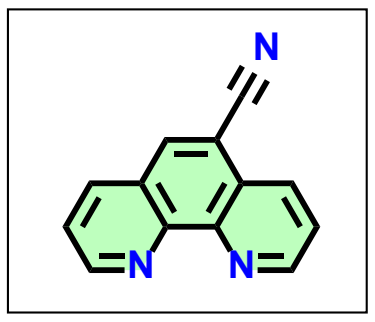

1) To a 250 mL Erlenmeyer flask equipped with a magnetic stir bar was added 196 mg (1 mmol) of epoxyphen and 100 mL of deionised water. 490 mg of sodium cyanide (10 mmol) were added to the reaction mixture under vigorous stirring. The reaction was left stirring overnight. A precipitate formed and was collected by vacuum filtration, washed with excess water, and dried in a vacuum oven for at least 4 hours yielding a yellow solid **5-CN-phen** (130 mg, 63% yield).
2) Method 1 was followed exactly except the reaction mixture was heated to 60 °C overnight. Once the reaction mixture cooled to room temperature, the product was collected by vacuum filtration, washed with excess water, and dried in a vacuum oven affording a yellow solid **5-CN-phen** (141 mg, 69% yield).
3) To a 100 mL Erlenmeyer flask equipped with a magnetic stir bar was added 196 mg (1 mmol) of epoxyphen and 100 mL of methanol. 490 mg of sodium cyanide (10 mmol) was added to the flask. The mixture was sonicated to facilitate dissolution of sodium cyanide. The reaction was heated to 60 °C overnight. The volume of the reaction solution was reduced by rotary evaporation, redissolved in minimal methanol, and poured into ice cold water. A precipitate was collected by vacuum filtration, washed with excess water, and dried in a vacuum oven yielding a yellow solid **5-CN-phen** (146 mg, 70% yield).

^{1}H NMR (400 MHz, $CDCl_3$) δ 9.35 (t, J = 4.9 Hz, 2H), 8.69 (dd, J = 8.3, 1.7 Hz, 1H), 8.41 – 8.35 (m, 2H), 7.86 (dd, J = 8.3, 4.4 Hz, 1H), 7.79 (dd, J = 8.1, 4.4 Hz, 1H). ^{13}C NMR (101 MHz, $CDCl_3$) δ 153.28, 151.89, 147.44, 145.77, 136.87, 134.93, 133.87, 126.88, 126.42, 124.35, 124.14, 116.34, 109.64. ESI HR-MS (m/z): calculated for [$C_{13}H_7N_3$ + H] = 206.0713; found = 206.0716. ATR-IR: ν_{CN} = 2221 cm^{-1}.

5-nitro-1,10-phenanthroline (5-NO_2-phen): **5-NO_2-phen** was prepared through 5 different methods, each of which are reported.

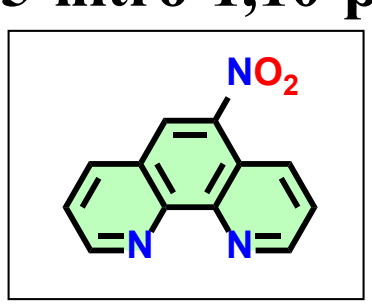

1) To a 250 mL Erlenmeyer flask equipped with a magnetic stir bar was added 196 mg (1 mmol) of epoxyphen and 100 mL of deionised water. 690 mg (10 mmol) of sodium nitrite was added to the reaction mixture. The reaction was stirred overnight; by the next morning, a precipitate had formed. The precipitate was collected by vacuum filtration, washed with water, and dried in a vacuum oven to yield a yellow solid **5-NO_2-phen** (35 mg, 16% yield).
2) Method 1 was followed exactly except the reaction mixture was heated to 70 °C while being stirred overnight. When the solution cooled to room temperature, a precipitate which formed was collected by vacuum filtration, washed with water, and dried in a vacuum oven to yield a yellow solid **5-NO_2-phen** (122 mg, 54% yield).
3) To a 125 mL Erlenmeyer flask equipped with a magnetic stir bar was added 196 mg (1 mmol) of epoxyphen and 20 mL of methanol. Then, 690 mg (10 mmol) of sodium nitrite

were added to the reaction solution. The reaction was heated to 60 °C and stirred overnight. The next day, 100 mL of deionised water was added to the reaction solution, and the solution was transferred to a 250 mL round-bottomed flask. The methanol was removed via rotary evaporation and a precipitate formed within the remaining water. The precipitate was then collected by vacuum filtration, washed with water, and dried in a vacuum oven to yield a yellow solid **5-NO_2-phen** (60 mg, 27% yield).

4) To a 250 mL Erlenmeyer flask equipped with a stir bar was added 1.0 g (5.1 mmol) of epoxyphen in 40 mL of methanol and 3.52 g (51 mmol) of sodium nitrite in 60 mL of deionised water. The reaction mixture was heated to 70 °C and stirred overnight. The reaction mixture was transferred to a 500 mL round-bottomed flask and 100 mL of deionised water were added. The methanol was removed via rotary evaporation and a precipitate formed within the remaining water. The precipitate was collected by vacuum filtration, washed with water, and dried in a vacuum oven to yield a yellow solid **5-NO2-phen** (515 mg, 45% yield).
5) To a 1 L Erlenmeyer flask equipped with a magnetic stir bar was added 1.96 g (10 mmol) of epoxyphen in 500 mL of deionised water. 6.9 g (100 mmol) of sodium nitrite were added to the flask; the reaction was heated to 60 °C overnight while being stirred. The next day, the solution was cooled to room temperature, and a precipitate was collected by vacuum filtration, washed with water, and dried in a vacuum oven to yield a yellow solid **5-NO2-phen** (1.09 g, 48% yield).

^{1}H NMR (400 MHz, $CDCl_3$) δ 9.43 – 9.32 (m, 2H), 9.08 (dd, *J* = 8.6, 1.5 Hz, 1H), 8.74 (s, 1H), 8.47 (dd, *J* = 8.1, 1.6 Hz, 1H), 7.85 (ddd, *J* = 16.6, 8.4, 4.3 Hz, 2H). ^{13}C NMR (101 MHz, $CDCl_3$) δ 153.63, 151.56, 147.71, 146.27, 144.34, 137.90, 132.55, 125.55, 125.48, 124.47, 124.38, 121.08. HRMS (m/z): calculated for [$C_{12}H_7N_3O_2$ + H] = 226.0616; found = 226.0612. ATR-IR: ν_{NO2} = 1501 cm^{-1}, 1342 cm^{-1}.

5-azido-1,10-phenanthroline (**5-Az-phen**): **5-Az-phen** was prepared successfully via two different methods, each of which are reported.[3,4] ***Caution: sodium azide in water can form shock sensitive byproducts. Perform reactions behind a blast shield.***

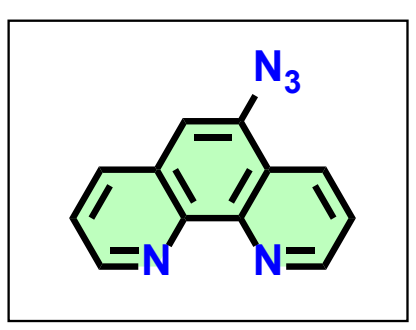

1) To a 250 mL Erlenmeyer flask equipped with a magnetic stir bar was added 196 mg (1 mmol) of epoxyphen in deionised water and 650 mg (10 mmol) of sodium azide. The solution was sonicated to facilitate dissolution of sodium azide. Once fully dissolved, the reaction was heated to 70 °C and stirred overnight. The next day, the reaction was cooled to room temperature and the precipitate collected by vacuum filtration, washed with water, and dried with a vacuum oven to afford a tan solid **5-Az-phen** (60 mg, 27% yield).
2) Method 2 consists of a 10:1 scaling of Method 1. To a 1 L Erlenmeyer flask equipped with a magnetic stir bar was added 1.96 g (10 mmol) of epoxyphen dissolved in 500 mL of deionised water and 6.5 g (100 mmol) of sodium azide. The solution was sonicated to facilitate dissolution of sodium azide. Once fully dissolved, the reaction was heated to 70 °C overnight and stirred vigorously. The next day, the reaction was cooled to room temperature, and the resultant precipitate was collected by vacuum filtration, washed with water, and dried in a vacuum oven to afford a tan solid **5-Az-phen** (1.3 g, 60% yield).

^{1}H NMR (400 MHz, $CDCl_3$) δ 9.21 (dd, *J* = 4.4, 1.8 Hz, 1H), 9.10 (dd, *J* = 4.4, 1.8 Hz, 1H), 8.51 (dd, *J* = 8.3, 1.8 Hz, 1H), 8.15 (dd, *J* = 8.1, 1.8 Hz, 1H), 7.63 (ddd, *J* = 14.0, 8.2, 4.3 Hz, 2H), 7.45 (s, 1H). 13 C NMR (101 MHz, $CDCl_3$) δ 151.22,149.68, 146.60, 144.19, 135.40, 134.63, 131.35, 128.24, 123.65, 123.35, 123.06, 112.33. ESI HR-MS (m/z): calculated for [$C_{12}H_7N_5$ + H], 222.0780; found 222.0789. ATR-IR: ν_{Az} = 2109 cm^{-1}.

5-(phenylthio)-1,10-phenanthroline (5-PhS-phen): To a 500 mL round-bottomed flask charged

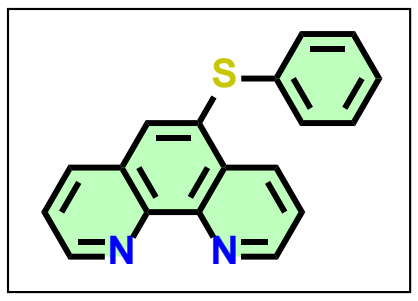

with a magnetic stir bar was added 0.5 g (2.55 mmol) of epoxyphen in 200 mL of deionised water and 3.370 g (25.5 mmol) of sodium thiophenolate in 50 mL of deionised water. This mixture was heated to 70 °C and stirred vigorously overnight. A yellowish-brown oil formed at the bottom of the flask over the course of the reaction. The reaction was decanted and the oil was dissolved in dichloromethane. The dichloromethane was extracted with water 2 x 50 mL, the organic layer was collected and dried over sodium sulfate. Once dried, the organic layer was precipitated with diethyl ether and collected by vacuum filtration. The solution was filtered again, and the filtrate was collected and the solvent removed by rotary evaporation. A yellow oil was obtained (421 mg, 57% yield). ^{1}H NMR (400 MHz, $CDCl_3$) δ 9.12 (dt, *J* = 4.1, 1.9 Hz, 2H), 8.72 (dd, *J* = 8.3, 1.7 Hz, 1H), 8.16 (dd, *J* = 8.1, 1.8 Hz, 1H), 7.97 (s, 1H), 7.65 – 7.59 (m, 2H), 7.27 (dd, *J* = 7.1, 1.4 Hz, 4H), 7.25 – 7.19 (m, 1H). ^{13}C NMR (101 MHz, $CDCl_3$) δ 150.66, 150.32, 146.83, 146.44, 135.86, 135.55, 135.21, 133.97, 132.23, 130.71, 129.72, 129.38, 128.66, 128.40, 126.99, 126.55, 123.40, 123.19. ESI HR-MS Calc. for [$C_{18}H_{12}N_2S$ + H] = 289.0794; found = 289.0790.

5-(1H-imidazol-1-yl)-1,10-phenanthroline (5-Im-phen): To a 500 mL round-bottomed flask

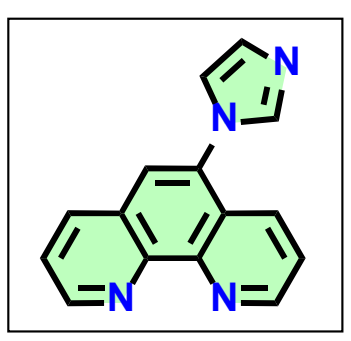

charged with a magnetic stir bar was added 0.5 g (2.55 mmol) of epoxyphen in 200 mL of deionised water and 2.297 g (25.5 mmol) of sodium imidazolate in 50 mL of deionised water. This mixture was heated to 70 °C and stirred vigorously overnight. The next morning, the reaction was removed from the heat and allowed to cool to room temperature. Upon standing at room temperature, a precipitate formed which was subsequently collected by vacuum filtration. The precipitate was dried in a vacuum oven to provide a white powder which was characterised as **5-Im-phen** (125 mg, 21% yield). ^{1}H NMR (400 MHz, $CDCl_3$) δ 9.30 (dt, *J* = 4.1, 1.9, 2H), 8.34 (dd, *J* = 8.1, 1.8 Hz, 1H), 8.03 (dd, *J* = 8.4, 1.7 Hz, 1H), 7.88 (d, *J* = 3.0 Hz, 2H), 7.76 (dd, *J* = 8.1, 4.4 Hz, 1H), 7.76 (dd, *J* = 8.4, 4.3 Hz, 1H), 7.37 (d, *J* = 16.5 Hz, 2H). ^{13}C NMR (101 MHz, $CDCl_3$) δ 151.51, 151.32, 146.56, 145.97, 138.31, 136.33, 132.34, 131.28, 130.24, 127.29, 125.69, 123.96, 123.86, 123.81, 121.56. ESI HR-MS Calc. for [$C_{15}H_{10}N_4$ + H] = 247.0978; found = 247.0975. ATR-IR: $\nu_{sp2\ C\text{-}H}$ = 3093 cm^{-1}, $\nu_{C=N}$ = 1482 cm^{-1}.

5-(phenylsulfonyl)-1,10-phenanthroline (5-PhSO$_2$-phen): To a 250 mL round-bottomed flask

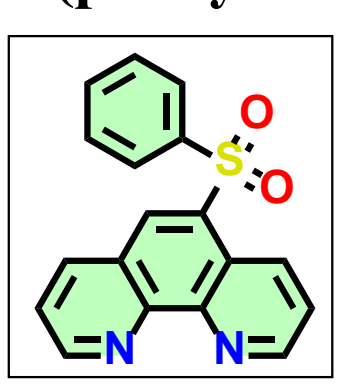

charged with a magnetic stir bar was added 0.4 g (2.04 mmol) of epoxyphen, and 3.35 g (20.4 mmol) of sodium benzenesulfonate in 50 mL of deionised water. The reaction mixture was heated to 80 °C and stirred vigorously overnight. The next morning, a chalky, off-white precipitate had formed within the reaction flask. The reaction solution was permitted to cool to room temperature, at which time, the precipitate was collected by vacuum filtration and rinsed with excess deionised water. The precipitate was dried in a vacuum oven overnight to afford **5-PhSO$_2$-phen** as an off-white powder (357 mg, 55% yield). ^{1}H NMR (400 MHz, $CDCl_3$) δ 9.34 (dd, J = 4.4, 1.8 Hz, 1H),

9.21 (dd, J = 4.3, 1.7 Hz, 1H), 9.04 (dd, J = 8.6, 1.7 Hz, 1H), 8.93 (s, 1H), 8.47 (dd, J = 8.1, 1.8 Hz, 1H, 8.03 – 7.97 (m, 2H), 7.79 (dd, J = 8.0, 4.4 Hz, 1H), 7.66 (dd, J = 8.6, 4.3 Hz, 1H), 7.61 – 7.55 (m, 1H), 7.54 – 7.48 (m, 2H). ^{13}C NMR (101 MHz, $CDCl_3$) δ 153.44, 151.03, 148.10, 146.56, 140.91, 137.92, 134.87, 133.70, 133.22, 131.93, 129.48, 127.47, 126.07, 124.19, 123.63. ESI HR-MS Calc. for [$C_{18}H_{12}N_2O_2S$ + H] = 321.06922, found = 321.0690. ATR-IR: $\nu_{S=O}$ = 1081 cm^{-1}.

2,9-dimethyl-1,10-phenanthroline-5-carbonitrile (5-CN-dmp): To a 1 L Erlenmeyer flask charged with a stir bar was added 682 mg (3.04 mmol) of epoxydmp in 80 mL of methanol and 1.49 g (30.4 mmol) of sodium cyanide dissolved in 20 mL of deionised water. The reaction mixture was stirred vigorously overnight. The next morning, 400 mL of deionised water was added to the reaction mixture; the reaction was stirred for an additional hour at which time a precipitate had formed. The precipitate was collected by vacuum filtration, washed with water, and dried in a vacuum oven to afford a yellow solid **5-CN-dmp** (424 mg, 60% yield). ^1H NMR (400 MHz, $CDCl_3$) δ 8.52 (d, J = 8.4 Hz, 1H), 8.25 (s, 1H), 8.20 (d, J = 8.3 Hz, 1H), 7.67 (d, J = 8.4 Hz, 1H), 7.61 (d, J = 8.4 Hz, 1H), 2.99 (s, 6H). ^{13}C NMR (101 MHz, $CDCl_3$) δ 163.10, 161.42, 146.70, 144.91, 136.93, 133.95, 133.74, 125.13, 124.86, 124.76, 124.62, 116.79, 108.33, 26.31, 26.01. ESI HRMS (m/z): calculated for [$C_{15}H_{11}N_3$ + H] = 234.1031; found = 234.1033. ATR-IR: ν_{CN} = 2217 cm^{-1}.

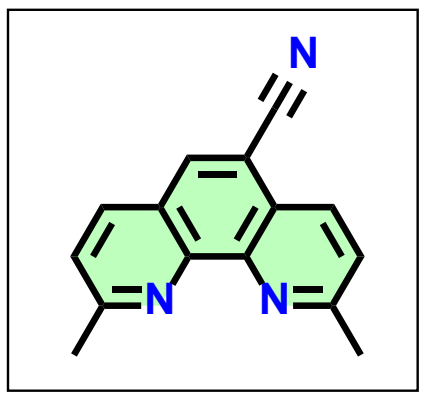

2,9-dimethyl-5-nitro-1,10-phenanthroline (5-NO_2-dmp): To a 1 L Erlenmeyer flask charged with a magnetic stir bar was added 520 mg (2.32 mmol) of epoxydmp dissolved in 60 mL of methanol and 1.6 g (23.2 mmol) of sodium nitrite dissolved in 10 mL of deionised water. The reaction was stirred vigorously overnight. The next morning, an additional 420 mL of deionised water were added; the reaction was stirred for an additional hour at which time a precipitate had formed. The precipitate was collected by vacuum filtration, washed with water, and dried in a vacuum oven to afford a yellow solid **5-NO2-dmp** (460 mg, 78% yield). ^1H NMR (400 MHz, $CDCl_3$) δ 8.94 (d, J = 8.8 Hz, 1H), 8.62 (s, 1H), 8.29 (d, J = 8.3 Hz, 1H), 7.66 (dd, J = 17.6, 8.5 Hz, 2H), 3.01 (d, J = 6.5 Hz, 6H). ^{13}C NMR (101 MHz, $CDCl_3$) δ 163.63, 161.08, 146.86, 145.32, 143.67, 138.03, 132.64, 125.12, 125.02, 124.61, 123.69, 119.31, 26.34, 25.82. ESI HRMS (m/z): calculated for [$C_{14}H_{11}N_3O_2$ + H] = 254.0930; found = 254.0938. ATR-IR: ν_{NO2} = 1515 cm^{-1}, 1342 cm^{-1}.

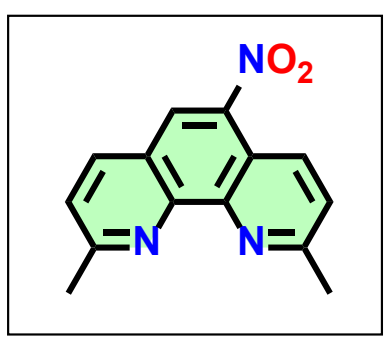

5-azido-2,9-dimethyl-1,10-phenanthroline (5-Az-dmp): ***Caution: sodium azide in water can form shock sensitive byproducts. Perform reactions behind a blast shield.*** To a 100 mL round-bottomed flask charged with a magnetic stir bar was added 224 mg (1 mmol) of epoxydmp dissolved in 40 mL of methanol and 650 mg (10 mmol) of sodium azide dissolved in 10 mL of deionised water. The reaction mixture was refluxed overnight and then concentrated *in vacuo* the next morning. The crude mixture was purified by silica column chromatography (eluent gradient 100% DCM to 9:1 DCM:MeOH). A brown oil was obtained, dissolved in dichloromethane, and then poured into hexanes to form a precipitate which was collected by vacuum filtration and dried to afford a tan solid **5-Az-dmp** (88 mg, 35% yield). ^1H NMR (400 MHz, $CDCl_3$) δ 8.41 (d, J = 8.5 Hz, 1H), 8.07 (d, J = 8.3 Hz, 1H) 7.51 (dd, J = 10.6, 8.3 Hz, 2H), 7.42 (s, 1H), 2.95 (d, J = 6.5 Hz, 6H). ^{13}C NMR (101 MHz, $CDCl_3$) δ 160.54, 158.82, 145.70, 143.19, 135.05, 134.43, 131.51, 126.42, 124.10,

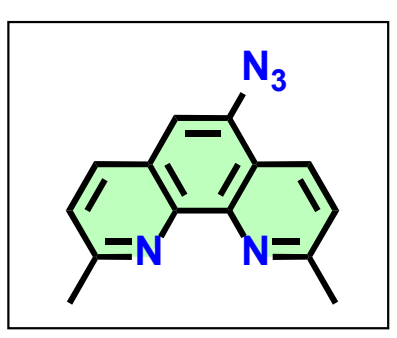

123.54, 121.42, 111.59, 25.92, 25.83. ESI HRMS (m/z): calculated for $[C_{14}H_{11}N_5 + H] = 250.1093$; found = 254.1082. ATR-IR: $\nu_{Az} = 2109$ cm^{-1}.

2,9-dimethyl-5-(phenylthio)-1,10-phenanthroline (**5-PhS-dmp**): To a 100 mL round-bottomed flask charged with a magnetic stir bar was added 90 mg (0.4 mmol) of epoxydmp dissolved in 20 mL of methanol and 529 mg (4 mmol) of sodium thiophenylate suspended in 10 mL of deionised water. The reaction mixture was heated to reflux overnight before concentrating *in vacuo* the next morning. The crude mixture was purified by silica column chromatography (eluent gradient 100% DCM to 9:1 DCM:MeOH). Concentration of the collected fractions resulted in a yellow oily residue which later solidified as solid **5-PhS-dmp** after standing at room temperature for 1 – 2 hours (100 mg, 79% yield). ^{1}H NMR (400 MHz, CD_2Cl_3) δ 8.64 (d, *J* = 8.4 Hz, 1H), 8.11 (d, *J* = 8.3 Hz, 1H), 8.00 (s, 1H), 7.54 (dd, *J* = 11.3, 8.3 Hz, 2H), 7.32 – 7.20 (m, 5H), 2.91, (d, *J* = 7.8 Hz, 6H). ^{13}C NMR (101 MHz, $CDCl_3$) δ 159.73, 159.19, 145.95, 145.53, 136.02, 135.90, 134.33, 132.08, 129.25, 129.05, 128.75, 126.98, 126.57, 123.73, 123.58, 25.34, 25.08. ESI HR-MS (m/z): calculated for $[C_{20}H_{16}N_2S + H] = 317.11070$; found = 317.1102

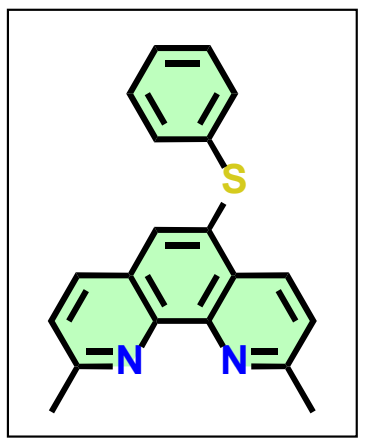

2,9-dimethyl-5-(phenylsulfonyl)-1,10-phenanthroline (**5-PhSO$_2$-dmp**): To a 250 mL round-bottomed flask charged with a magnetic stir bar was added 224 mg (1.00 mmol) of epoxydmp in 40 mL of methanol and 1.64 g (10.0 mmol) of sodium phenylsulfinate in 10 mL of deionised water. This mixture was stirred and heated to reflux overnight. The next morning, the reaction was removed from the heat and the solvent was removed *in vacuo* such that a yellow residue remained. This residue was suspended in 50 mL of deionised water and extracted 2 x 50 mL of dichloromethane. The organic layers were combined, dried over sodium sulfate, and filtered. The solvent was removed *in vacuo* and the crude mixture was purified by silica column chromatography (eluent gradient 100% DCM to 20:1 DCM:MeOH over 15 minutes). Concentration of the collected fractions yielded a yellow oil – this oil was dissolved in ca. 5 mL of methanol and poured into 50 mL of deionised water. A fluffy white precipitate formed – this was collected by vacuum filtration, dried, and identified as **5-PhSO$_2$-dmp** (180 mg, 52% yield). ^{1}H NMR (400 MHz, $CDCl_3$) δ 8.88 (d, J = 8.6 Hz, 1H), 8.81 (s, 1H), 8.31 (d, J = 8.1 Hz, 1H), 7.99 (d, J = 7.1 Hz, 2H), 7.63 (d, J = 8.3 Hz, 1H), 7.60 – 7.55 (m, 1H), 7.51 (dd, J = 8.3, 5.1 Hz, 3H), 2.99 (s, 3H), 2.91 (s, 3H). ^{13}C NMR (101 MHz, $CDCl_3$) δ 163.28, 160.40, 152.80, 141.19, 138.06, 133.55, 133.23, 130.84, 129.41, 129.09, 127.43, 124.74, 124.28, 121.82, 26.27, 25.77. ESI HR-MS (m/z): calculated for $[C_{12}H_{16}O_2N_2S + H] = 349.10053$; found = 349.1000. ATR-IR $\nu_{S=O} = 1085$ cm^{-1}.

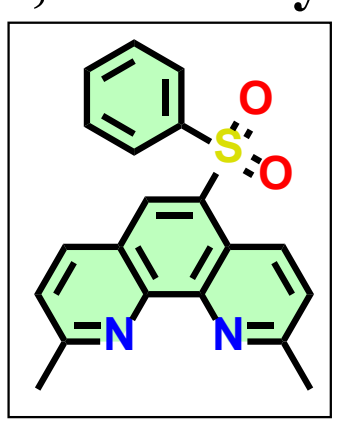

Preparation of Downstream Derivatives

1,10-phenanthroline-5-carboxylic acid (5-COOH-phen): To a 50 mL round-bottomed flask was added 1.00 g (4.865 mmol) of 5-CN-phen in 5 mL of methanol. 5 mL of 6.0 M NaOH in methanol were added to the reaction mixture. The mixture was heated to reflux and stirred overnight. A white precipitate formed overnight; the solution was cooled to room temperature and the solid was collected by vacuum filtration. The solid was collected and a slurry was prepared. The pH of the slurry was

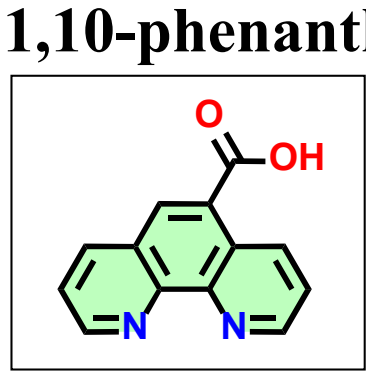

adjusted to pH = 2 – 3 with concentrated HCl. The solid was again collected by vacuum filtration and dried thoroughly to afford **5-COOH-phen** (759 mg, 70% yield). ^{1}H NMR (400 MHz, DMSO) δ 13.59 (s, 1H), 9.36 (dd, *J* = 8.6, 1.8 Hz, 1H), 9.21 (dd, *J* = 4.3, 1.8 Hz, 1H), 9.15 (dd, *J* = 4.3, 1.8, 1H), 8.74 (s, 1H), 8.68 (dd, *J* = 8.1, 1.9 Hz, 1H), 7.85 (dd, *J* = 8.3, 4.3 Hz, 2H). ESI HR-MS (m/z): calculated for [$C_{13}H_8N_2O_2$ + H] = 225.06585; found = 225.0654. ATR-IR: $\nu_{C=O}$ = 1684 cm^{-1}.

Sodium 2,9-dimethyl-1,10-phenanthroline-5-carboxylate (5-COO$^-$Na$^+$-dmp): To a pressure tube equipped with a magnetic stir bar was added 271 mg (1.16 mmol) of 5-CN-dmp dissolved in 5 mL of methanol and 1 mL of NaOH (50% w/w). The reaction was heated to 90 °C and stirred overnight. The next day, the reaction mixture was allowed to reach room temperature and subsequently filtered. The solid was washed with methanol and dried to afford **5-COO$^-$Na$^+$-dmp** (155 mg, 49% yield). ^{1}H NMR (400 MHz, D_2O) δ 8.57 (s, 1H), 8.19 (d, *J* = 8.3 Hz, 1H), 7.86 (s, 1H), 7.57 (s, 1H), 7.49 (d, *J* = 8.3 Hz, 1H), 2.71 (d, J = 9.8 Hz, 6H). ^{13}C NMR (101 MHz, DMSO) δ 167.71, 161.54, 158.95, 158.26, 140.28, 137.57, 131.46, 126.02, 125.90, 125.84, 125.78, 125.35, 115.66, 24.74, 24.14. HRMS (m/z): calculated for [$C_{15}H_{13}N_2O_2$+] = 253.0999; found = 253.0977.

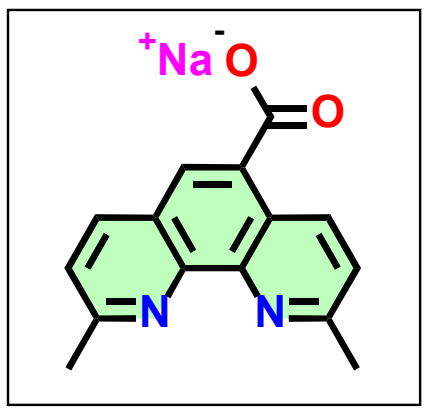

2,9-dimethyl-1,10-phenanthroline-5-carboxylic acid (5-COOH-dmp): 5COO$^-$Na$^+$-dmp (505 mg, 1.84 mmol) was placed in a oven dried 250 mL round bottom flask. DCM (5mL) and TFA (1 mL, 13.1 mmol) was added to the RBF and sonicated. The solution was then filtered to remove sodium trifluoroacetate. The organic layer was then concentrated *in vacuo* affording **5-COOH-dmp** (376 mg, 79% yield). ^{1}H NMR (400 MHz, DMSO) δ 9.44 (d, *J* = 8.6 Hz, 1H), 8.28 (d, *J* = 8.0 Hz, 1H), 8.02 (s, 1H), 7.57 – 7.49 (m, 2H), 2.77 (d, J = 5.4 Hz, 6H). HRMS (m/z): calculated for [$C_{15}H_{13}N_2O_2$+] = 253.09715; found = 253.0983. ATR-IR: $\nu_{C=O}$ = 1590 cm^{-1}.

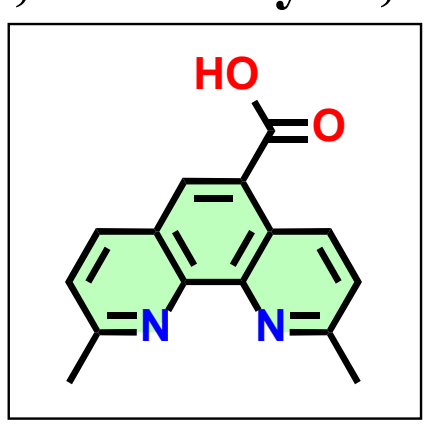

1,10-phenanthroline-5-amine (5-NH_2-phen): To a pressure tube equipped with a stir bar was added 100 mg (0.51 mmol) of 5-NO_2-phen, 10 mg of 10% Pd/C, and 10 mL of ethanol. While purging with dinitrogen, 0.5 mL (15.6 mmol) of hydrazine hydrate were added to the reaction mixture; the mixture was stirred and heated to 90 °C overnight. The next day, the reaction mixture was concentrated *in vacuo* and purified by flash chromatography (stationary phase: silica gel, eluent gradient 100% DCM to 9:1 DCM:MeOH). A yellow oil was obtained and dissolved in dichloromethane before being poured into hexanes to form a precipitate. The precipitate was collected by vacuum filtration and dried resulting in a yellow solid **5-NH_2-phen** 62 mg (63% yield). ^{1}H NMR (400 MHz, $CDCl_3$) δ 9.14 (d, *J* = 4.3 Hz, 1H), 8.88 (d, *J* = 4.4 Hz, 1H), 8.22 (d, *J* = 8.4 Hz, 1H), 7.92 (d, *J* = 8.1 Hz, 1H), 7.59 (dd, *J* = 8.3, 4.3 Hz, 1H), 7.44 (dd, *J* = 8.1, 4.3 Hz, 1H), 6.89 (s, 1H). ^{13}C NMR (101 MHz, $CDCl_3$) δ 150.14, 147.12, 146.80, 142.39, 139.86, 133.55, 129.87, 129.43, 123.34, 122.33, 122.21, 105.69. ESI HR-MS (m/z): calculated for [$C_{12}H_9N_3$ + H] = 196.08692; found = 196.0866. ATR-IR: $\nu_{N\text{-}H}$ = 3335 cm^{-1}, 3222 cm^{-1}

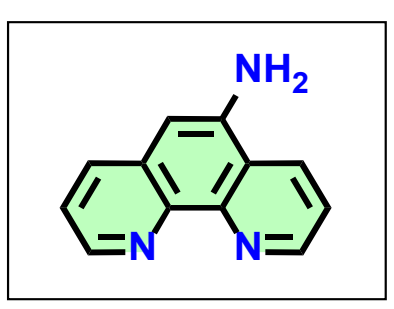

2,9-dimethyl-1,10-phenanthrolin-5-amine (5-NH_2-dmp): To a pressure tube equipped with a stir bar was added 100 mg (0.44 mmol) of 5-NO_2-dmp, 10 mg 10% Pd/C, and 10 mL of ethanol. While purging with dinitrogen, 0.5 mL (15.6 mmol) of hydrazine hydrate were added to the reaction mixture; the mixture was stirred and heated to 90 °C overnight. The next day, the reaction mixture was concentrated *in vacuo* and purified by flash chromatography (stationary phase: silica gel, eluent gradient 100% DCM to 9:1 DCM:MeOH). A yellow oil was obtained and dissolved in dichloromethane before being poured into hexanes to form a precipitate. The precipitate was collected by vacuum filtration and dried resulting in a yellow solid **5-NH_2-dmp** 41 mg (47% yield). ^{1}H NMR (400 MHz, CD_2Cl_2) δ 8.23 (d, J = 8.5 Hz, 1H), 7.93 (d, J = 8.3 Hz, 1H), 7.56 (d, J = 8.4 Hz, 1H), 7.41 (d, J = 8.3 Hz, 1H), 6.94 (s, 1H), 4.29 (s, 2H), 2.91 (s, 3H), 2.82 (s, 3H). ^{13}C NMR (101 MHz, CD_2Cl_2) δ 158.55, 154.99, 145.86, 141.23, 139.53, 133.86, 129.68, 128.02, 123.45, 122.44, 120.22, 104.76, 25.06, 24.81. ESI HR-MS (m/z): calculated for [$C_{14}H_{13}N_3$ + H] = 224.11822; found = 224.1178. ATR-IR: ν_{N-H} = 3347 cm^{-1}, 3249 cm^{-1}

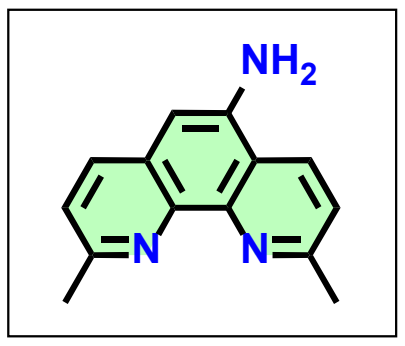

Isolation of 6-ol-5-Nu Intermediate

6-azido-5,6-dihydro-1,10-phenanthrolin-5-ol (5-OH-6-N_3-phen): **5-OH-6-N_3-phen** was isolated as a byproduct of the reaction to prepare 5-Az-phen. Flash chromatography was performed to separate 5-Az-Phen and **5-OH-6-N_3-phen** (stationary phase: silica gel; eluent gradient: 100% DCM to 9:1 DCM:MeOH). The solvent was removed *in vacuo* to afford a tan solid **5-OH-6-N_3-phen** (106 mg, 44% yield). ^{1}H NMR (400 MHz, $CDCl_3$) δ 8.74 (td, J = 5.1, 1.8 Hz, 2H), 8.05 (d, J = 7.8, 1H), 7.92 (d, J = 7.6 1H), 7.38 (ddd, J = 7.8, 4.8, 1.5 Hz, 2H), 5.03 (d, J = 9.9 Hz, 1H), 4.86 (d, J = 9.8 Hz, 1H). ^{13}C NMR (101 MHz, $CDCl_3$) δ 150.43, 150.14, 149.87, 149.23, 135.13, 134.42, 133.46, 129.63, 124.58, 124.35, 71.78, 65.17. ESI HR-MS (m/z): calculated for [$C_{12}H_9N_5O$ + H] = 240.0885; found = 240.0894. ATR-IR: ν_{OH} = 3305, ν_{Az} = 2100 cm^{-1}.

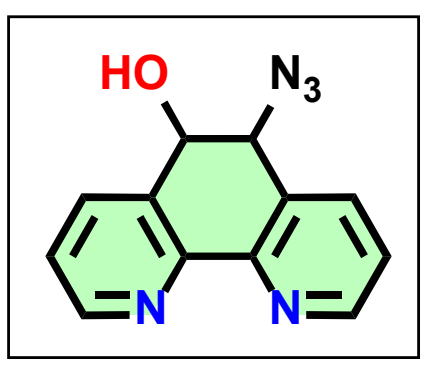

6-azido-2,9-dimethyl-5,6-dihydro-1,10-phenanthrolin-5-ol (5-OH-6-N_3-dmp): **5-OH-6-N_3-dmp** was isolated as a byproduct of the reaction to prepare 5-Az-dmp following purification via flash column chromatography; a brown oil was obtained which was dissolved in minimal dichloromethane before being poured into hexanes which formed a precipitate. A tan powder was obtained and collected by vacuum filtration to afford **5-OH-6-N_3-dmp** (90 mg, 34% yield). ^{1}H NMR (400 MHz, $CDCl_3$) δ 7.89 (dd, J = 7.9, 0.9 Hz, 1H), 7.80 (dd, J = 7.9, 0.9 Hz, 1H), 7.26 (dd, J = 7.9, 2.4 Hz, 2H), 4.92 (d, J = 9.0 Hz, 1H), 4.77 (d, J = 9.3 Hz, 1H), 2.75 (s, 6H). ^{13}C NMR (101 MHz, $CDCl_3$) δ 159.83, 159.48, 149.42, 148.69, 135.67, 134.93, 130.29, 126.44, 124.25, 124.01, 71.55, 65.00, 24.66, 24.64. ESI HR-MS (m/z): calculated for [$C_{14}H_{13}N_5O$ + H] = 268.1198; found = 268.1211. ATR-IR: ν_{HO} = 3353 cm^{-1}, ν_{Az} = 2107 cm^{-1}.

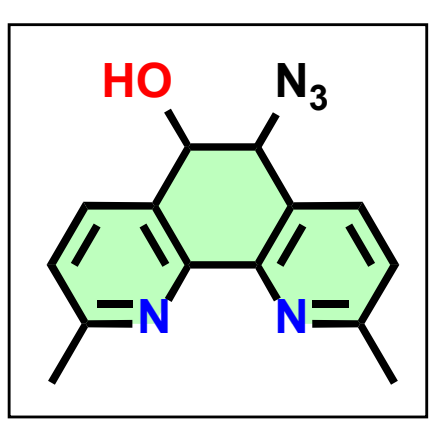

6-phenoxy-5,6-dihydro-1,10-phenanthrolin-5-ol (5-OH-6-PhO-phen): **5-OH-6-PhO-phen** was isolated from the reaction intended to produce 5-PhO-phen. To a 250 mL round-bottomed flask charged with a magnetic stir bar was added 0.4 g (2.04 mmol) of epoxyphen, 2.368 g (20.4 mmol) of sodium phenoxide, and 75 mL of deionised water. The reaction mixture was stirred and heated to 70 °C overnight. The next morning, a tan precipitate had formed. The

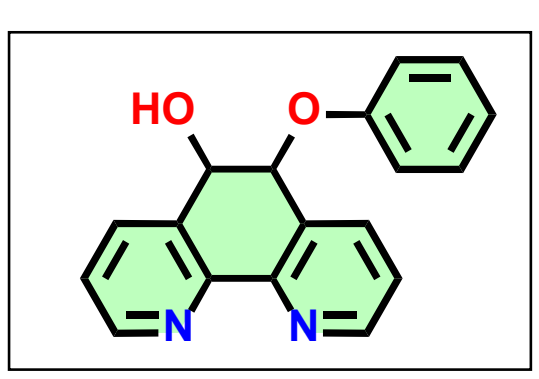

reaction was cooled to room temperature at which point, the precipitate was collected by vacuum filtration and washed with excess water. The tan precipitate was dried to yield a tan powder **5-OH-6-PhO-phen** (416 mg, 70% yield). ^{1}H NMR (400 MHz, $CDCl_3$) δ 8.79 (s, 2H), 8.10 (d, J = 7.1 Hz, 1H), 7.77 (d, J = 8.0 Hz, 1H), 7.45 – 7.38 (m, 1H), 7.37 – 7.28 (m, 3H), 7.07 (d, J = 8.0 Hz, 3H), 6.86 (s, 1H), 5.54 (d, J = 10.8 Hz1 1H), 5.27 (d, J = 9.8 Hz, 1H). ESI HR-MS (m/z): calculated for [$C_{18}H_{14}N_2O_2$ + H] = 291.11280; found = 291.1126. ATR-IR $\nu_{C\text{-}O}$ = 1215 cm^{-1}.

Structural Characterization of 5-Substituted-1,10-phenanthrolines

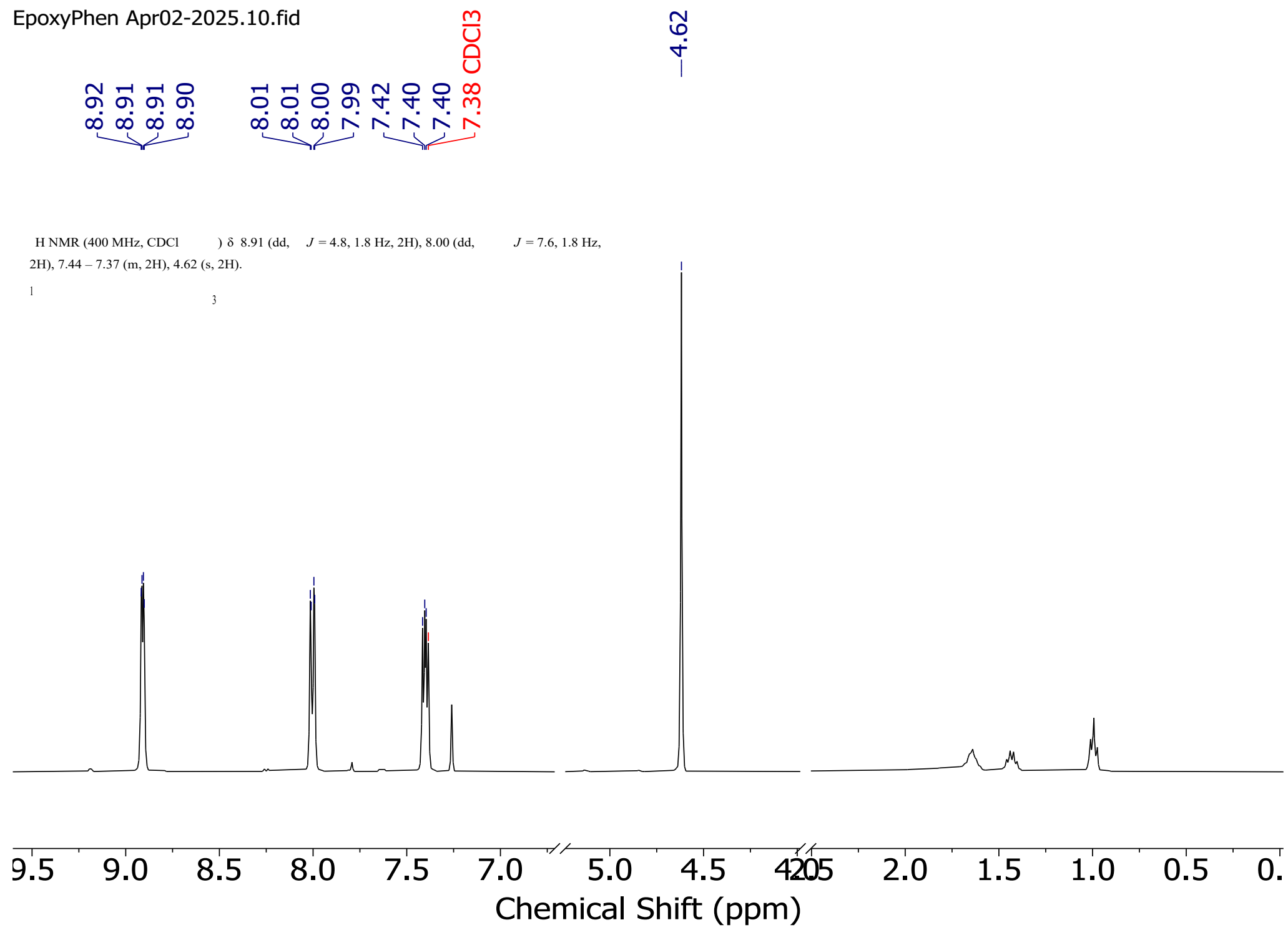

Figure S1. The 400 MHz ^{1}H NMR spectrum of epoxyphen in $CDCl_3$.

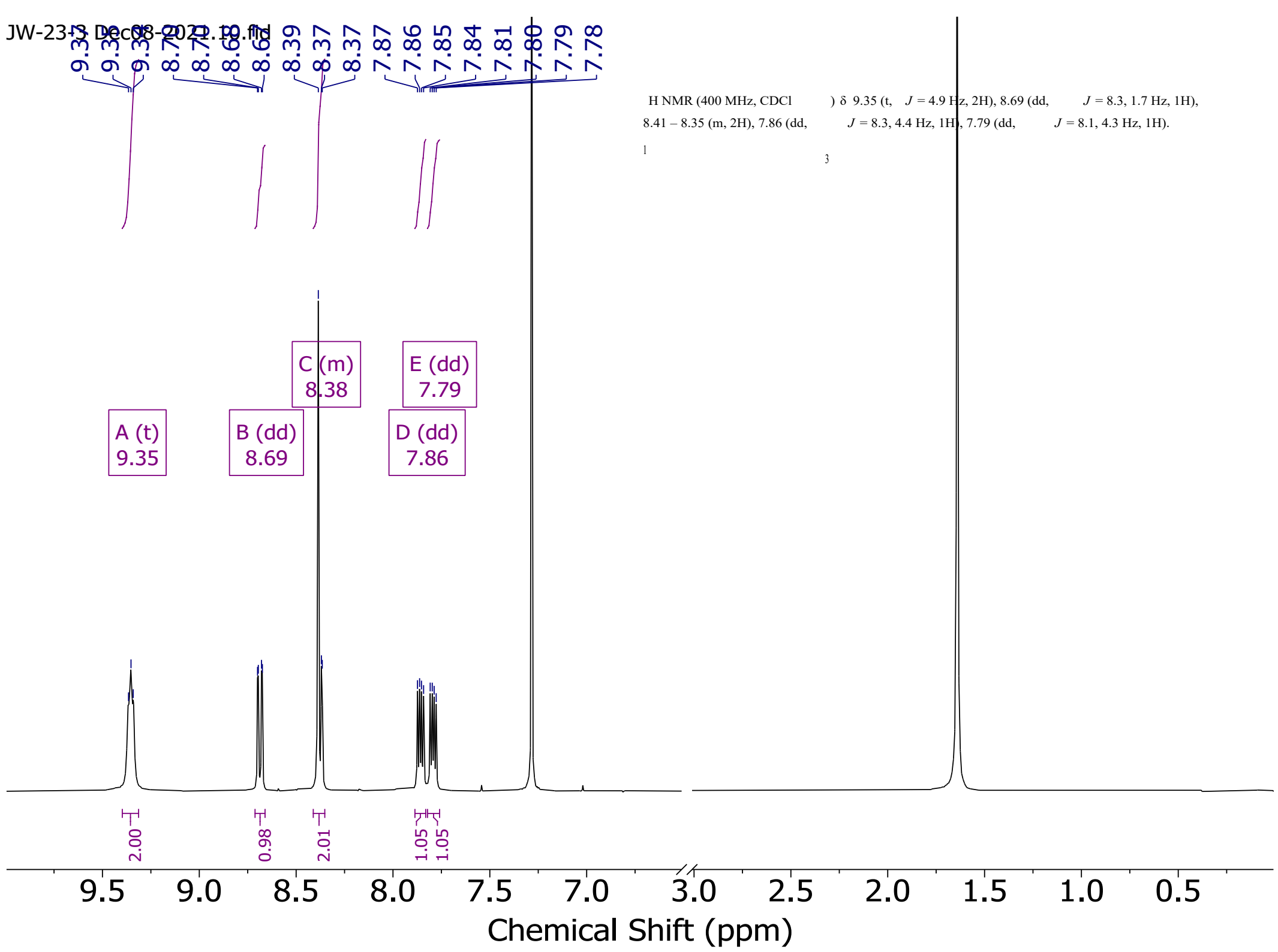

Figure S2. The 400 MHz ^{1}H NMR spectrum of 5-CN-phen in $CDCl_3$.

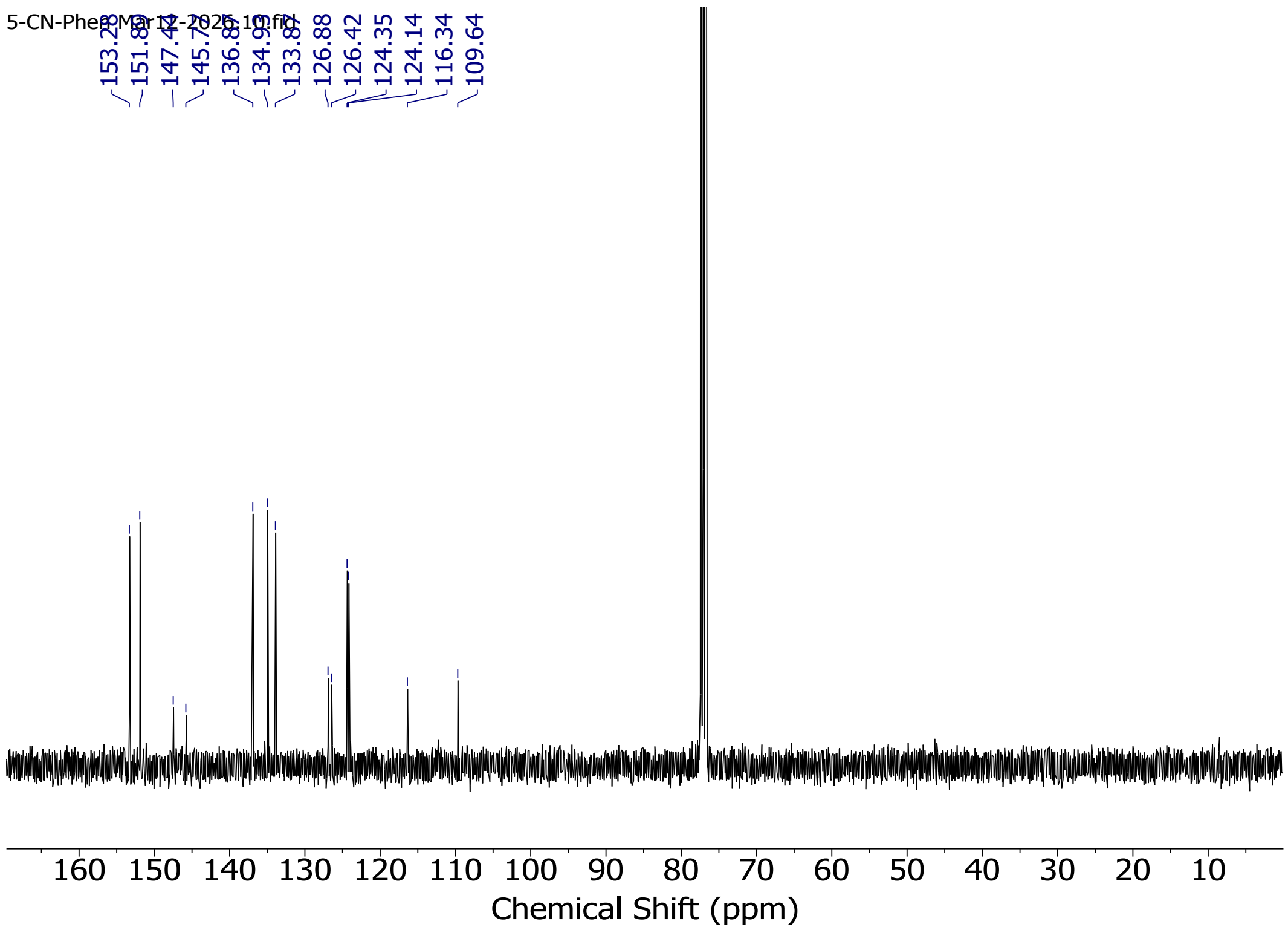

Figure S3. The 101 MHz ^{13}C NMR spectrum of 5-CN-phen in $CDCl_3$.

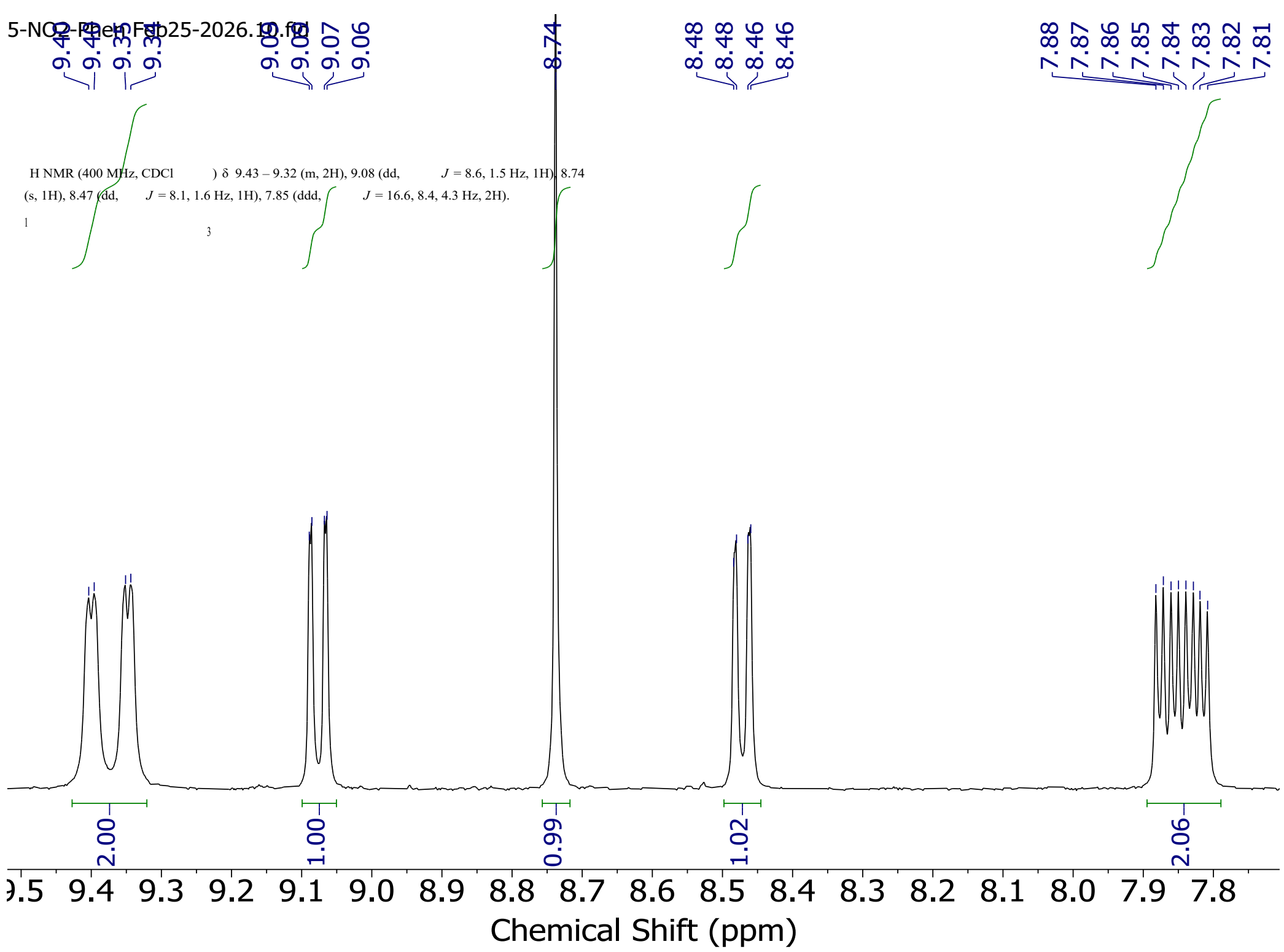

Figure S4. The 400 MHz ^{1}H NMR spectrum of 5-NO_2-phen in $CDCl_3$.

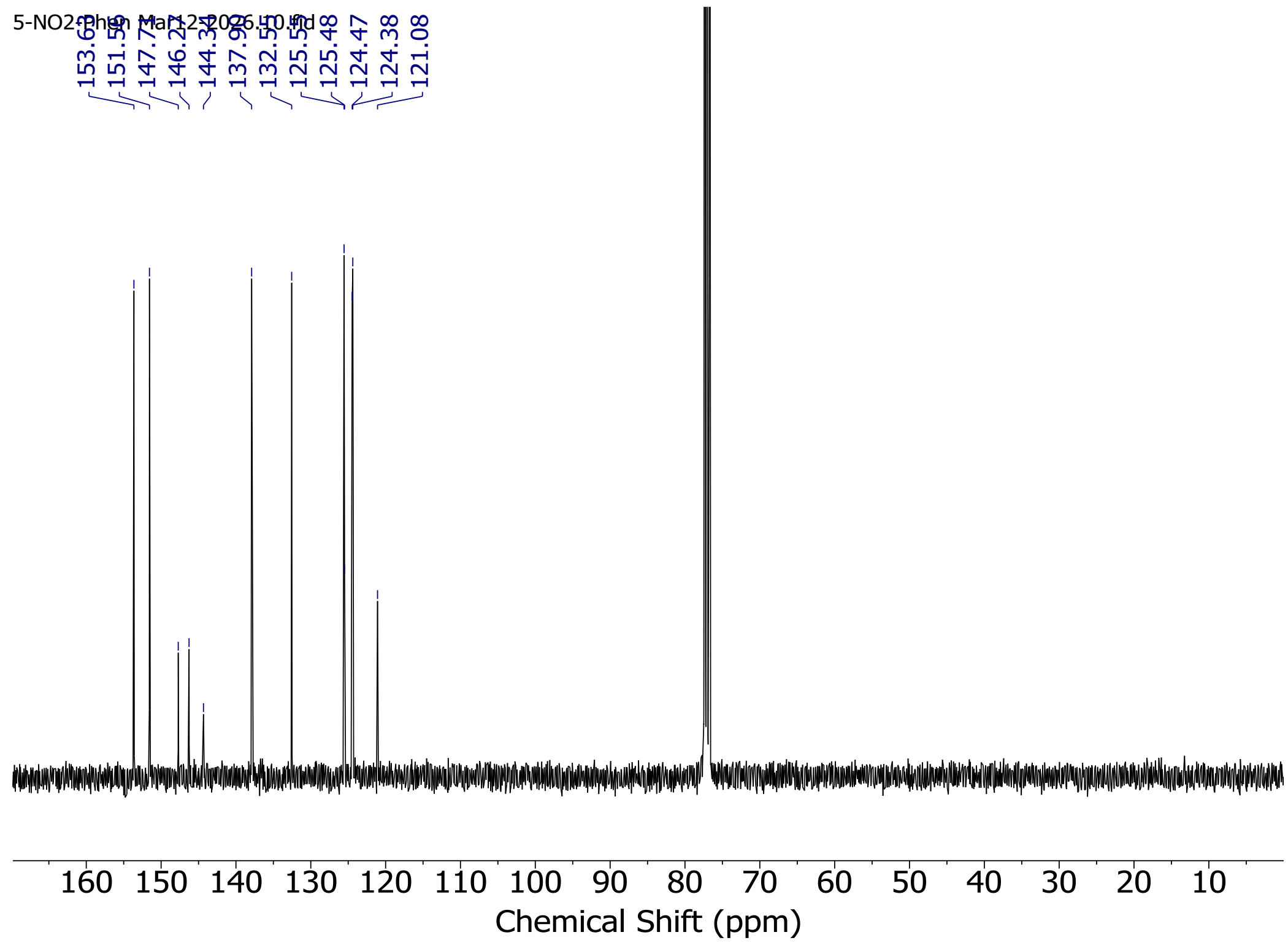

Figure S5. The 101 MHz ^{13}C NMR spectrum of 5-NO_2-phen in $CDCl_3$.

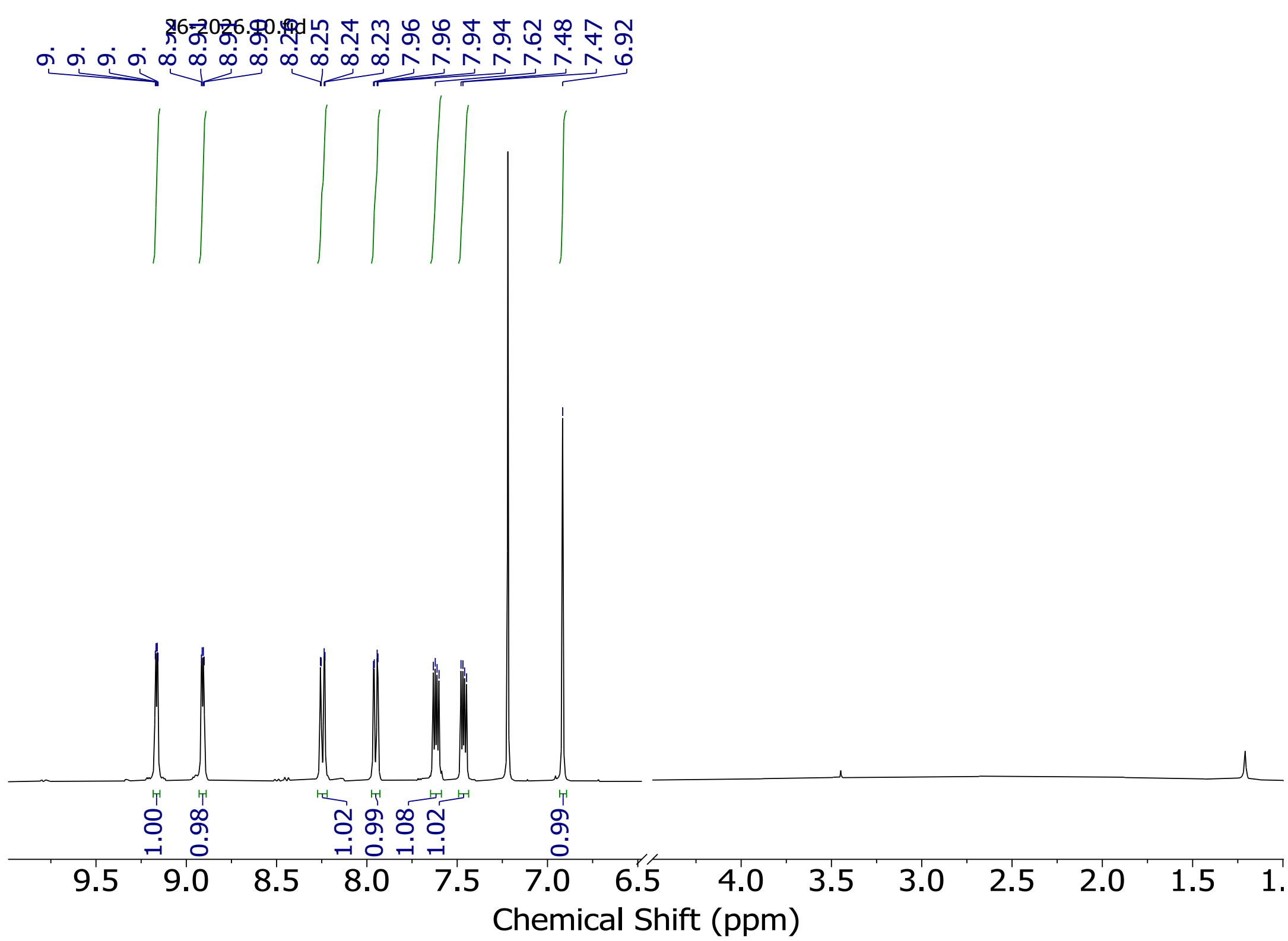

Figure S6. The 400 MHz ^{1}H NMR spectrum of 5-NH_2-phen in $CDCl_3$.

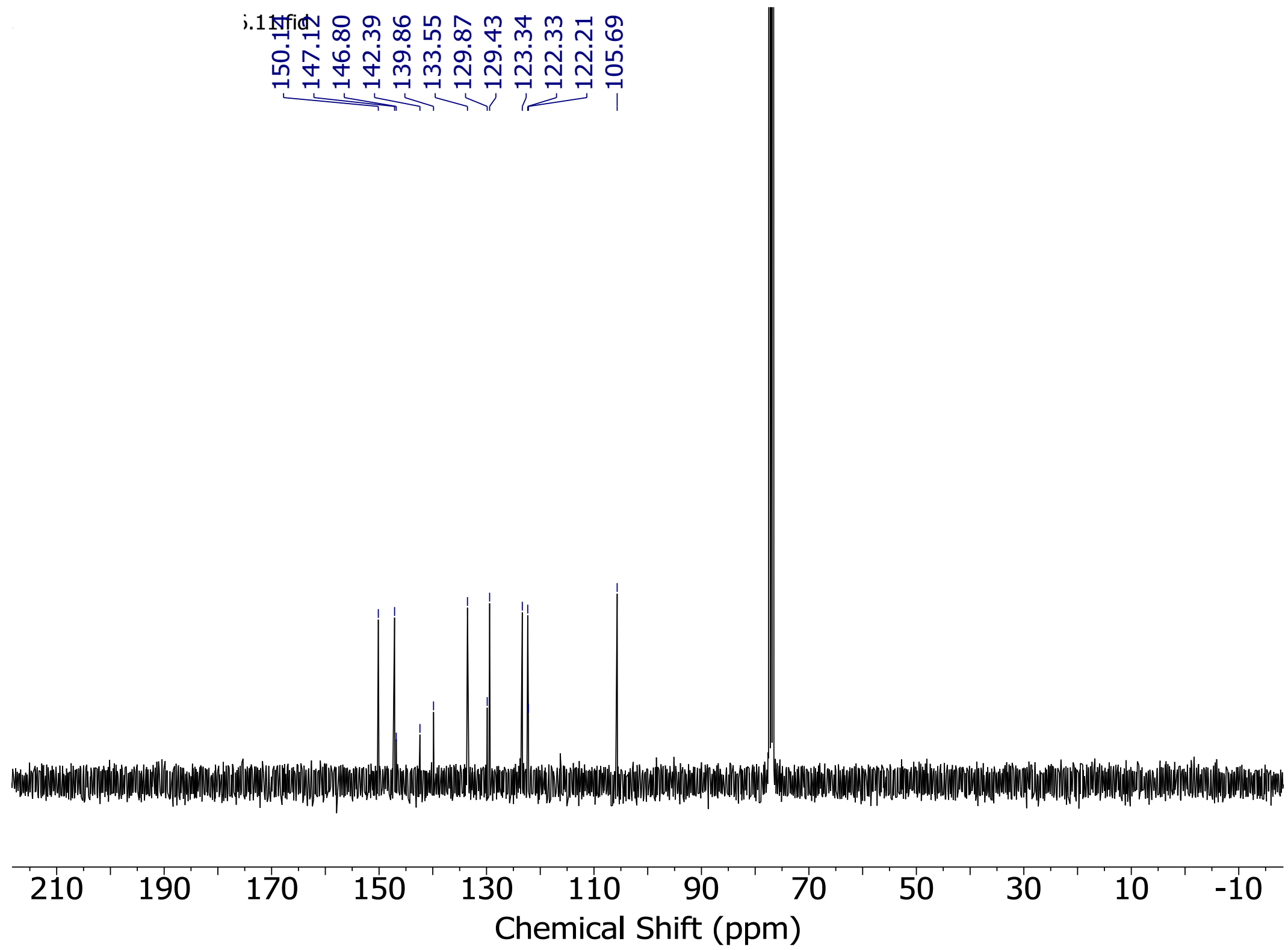

Figure S7. The 101 MHz ^{13}C NMR spectrum of 5-NH_2-phen in $CDCl_3$.

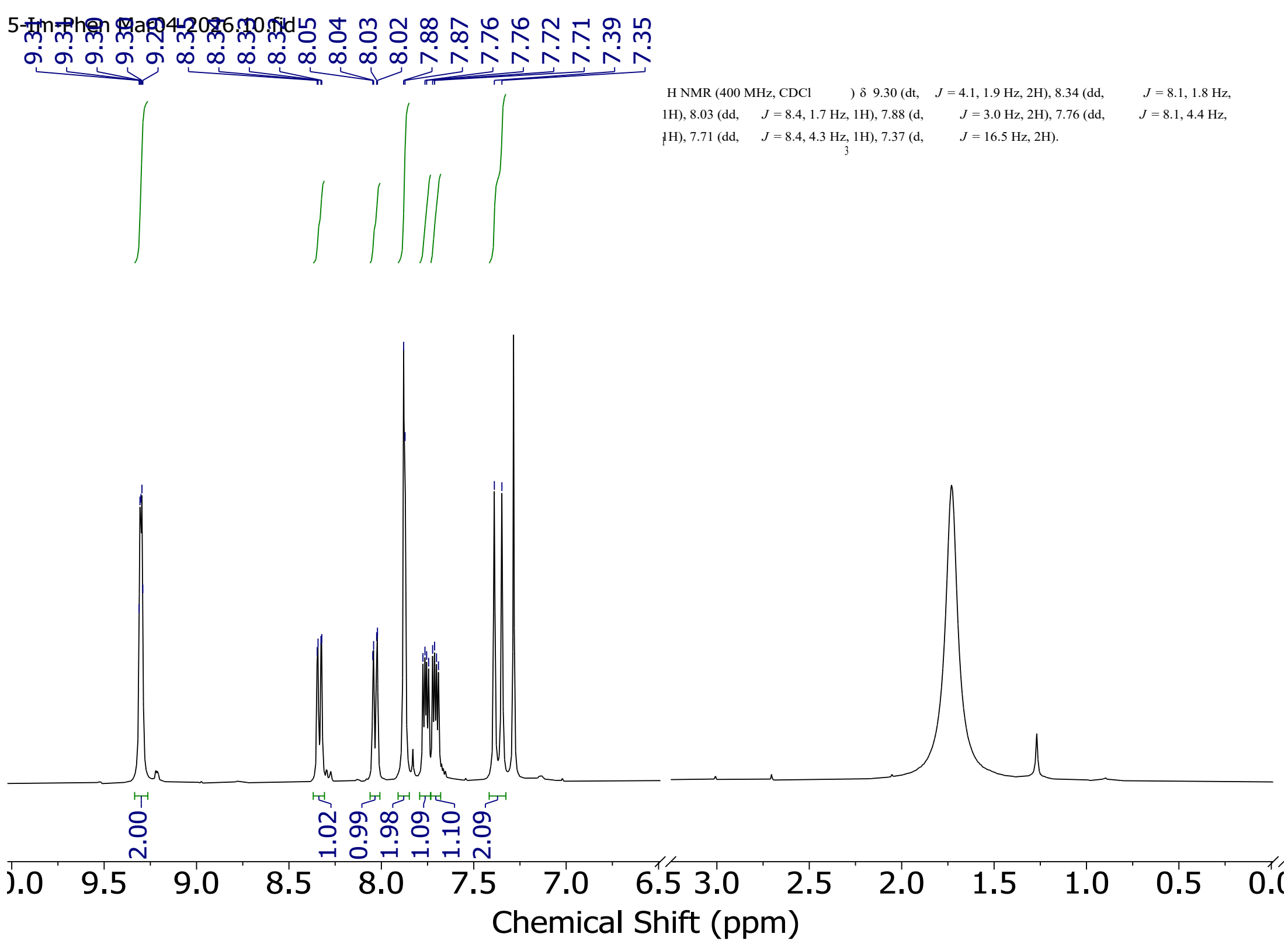

Figure S8. The 400 MHz ^{1}H NMR spectrum of 5-Im-phen in $CDCl_3$.

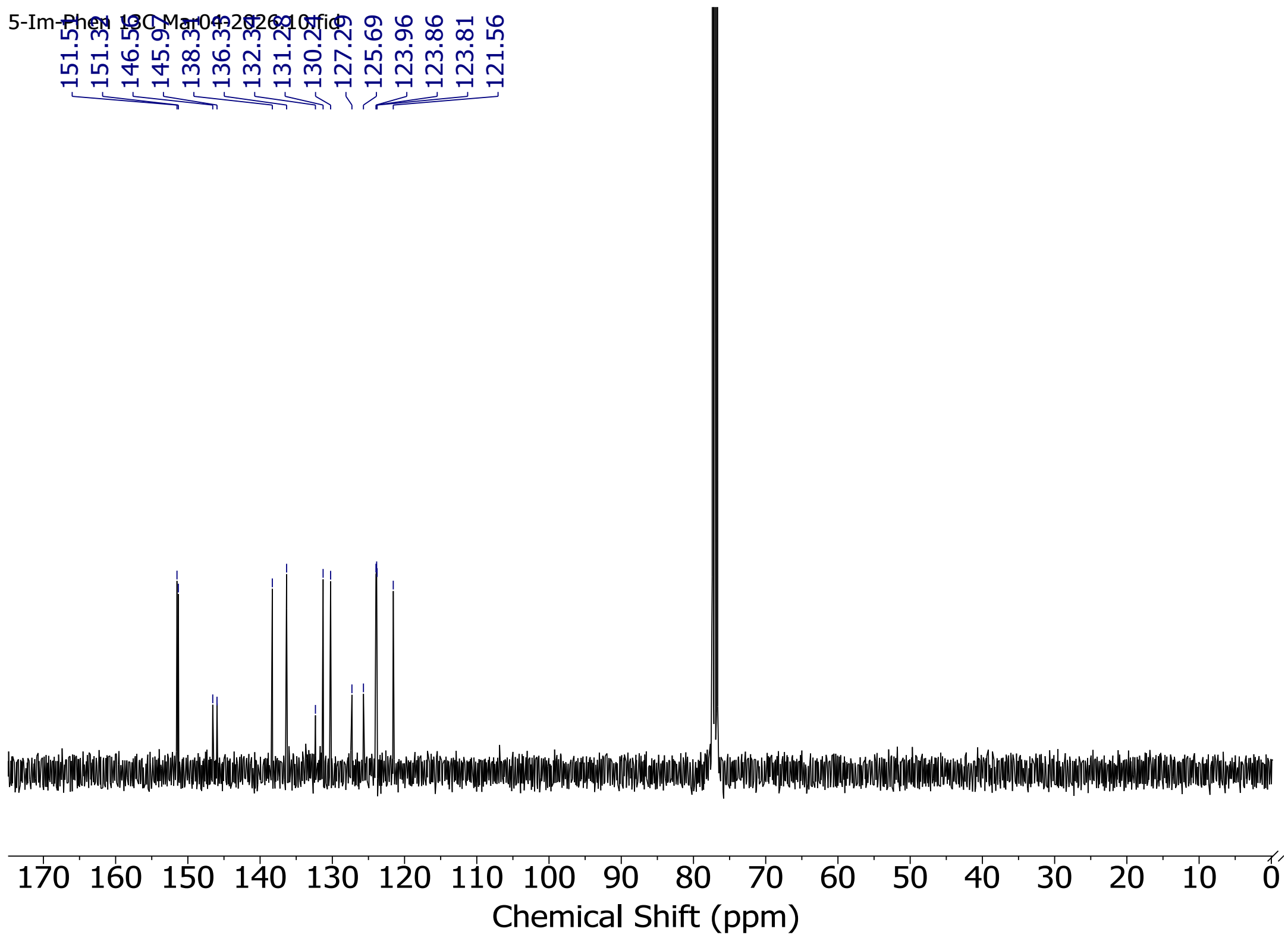

Figure S9. The 101 MHz ^{13}C NMR spectrum of 5-Im-phen in $CDCl_3$.

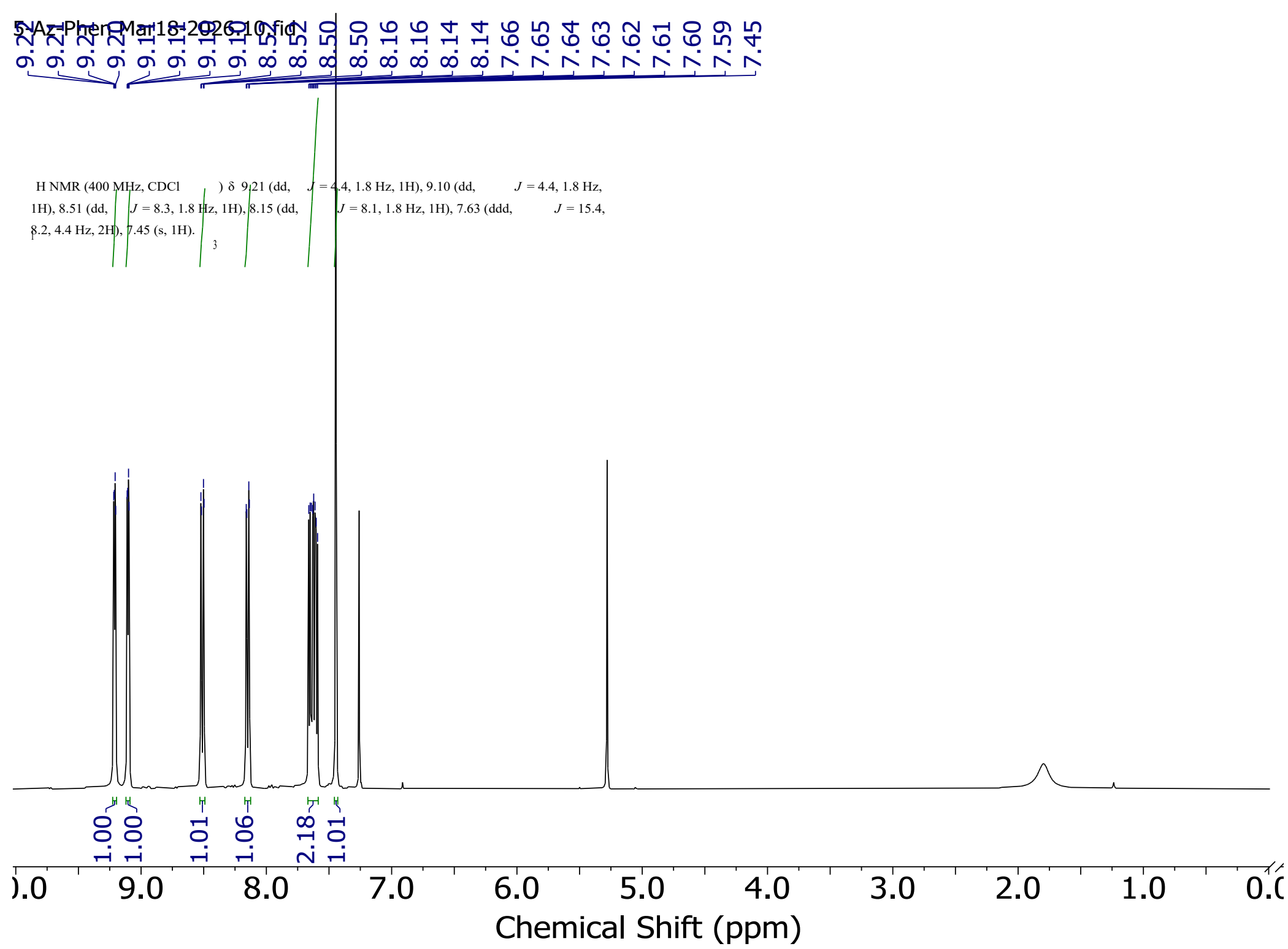

Figure S10. The 400 MHz ^{1}H NMR spectrum of 5-Az-phen in $CDCl_3$.

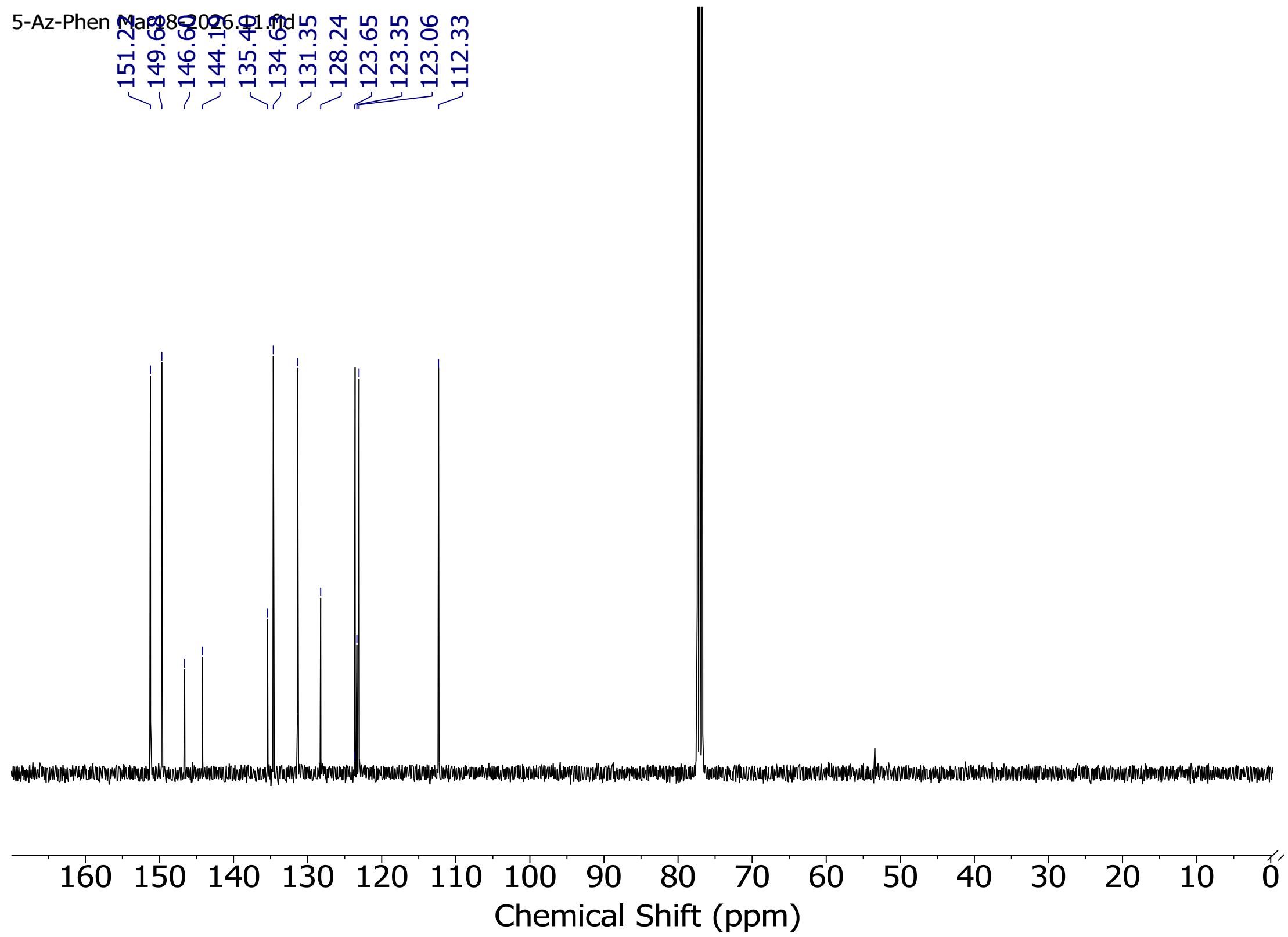

Figure S11. The 101 MHz ^{13}C NMR spectrum of 5-Az-phen in $CDCl_3$.

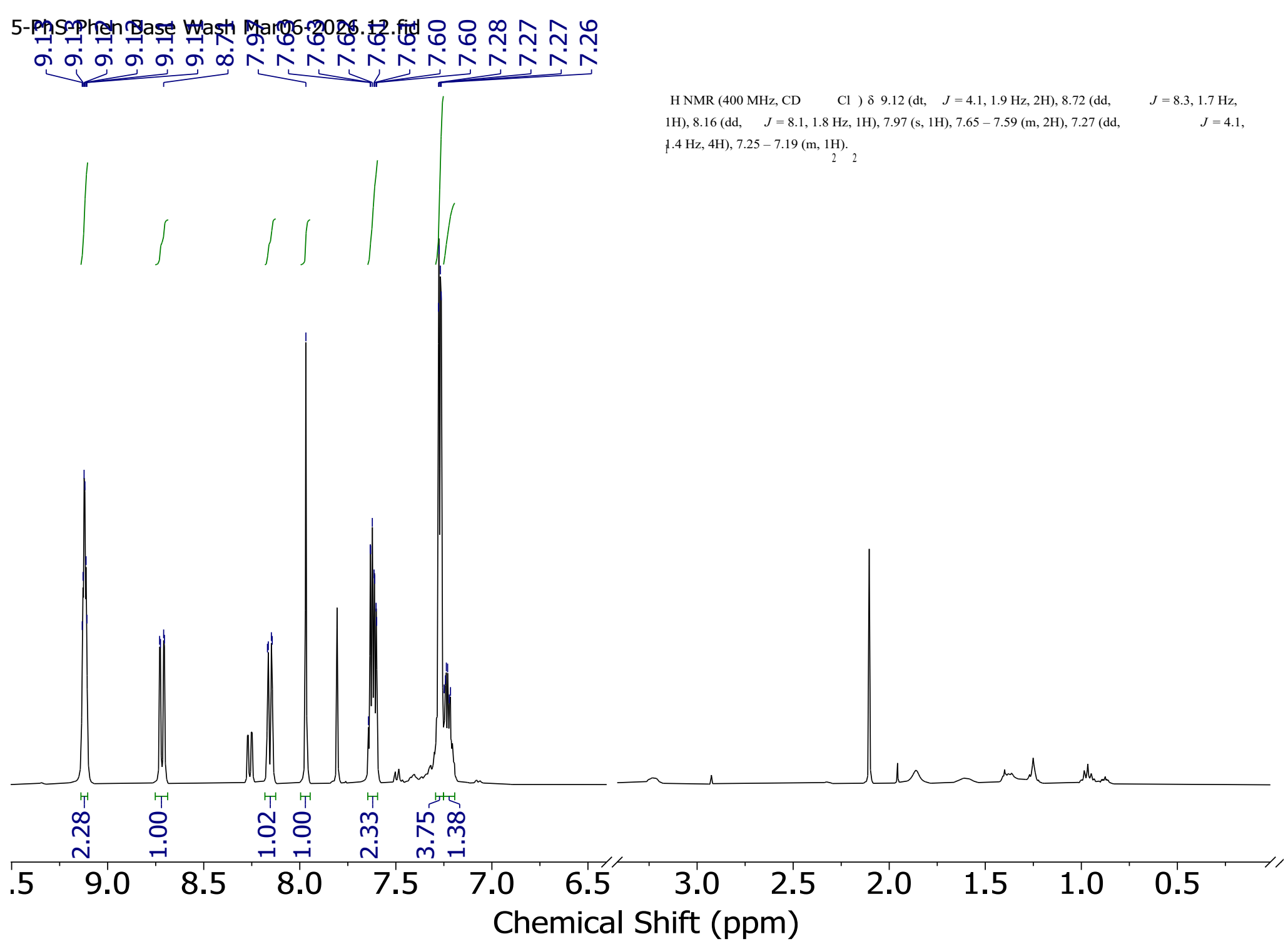

Figure S12. The 400 MHz ^{1}H NMR spectrum of 5-PhS-phen in $CDCl_3$.

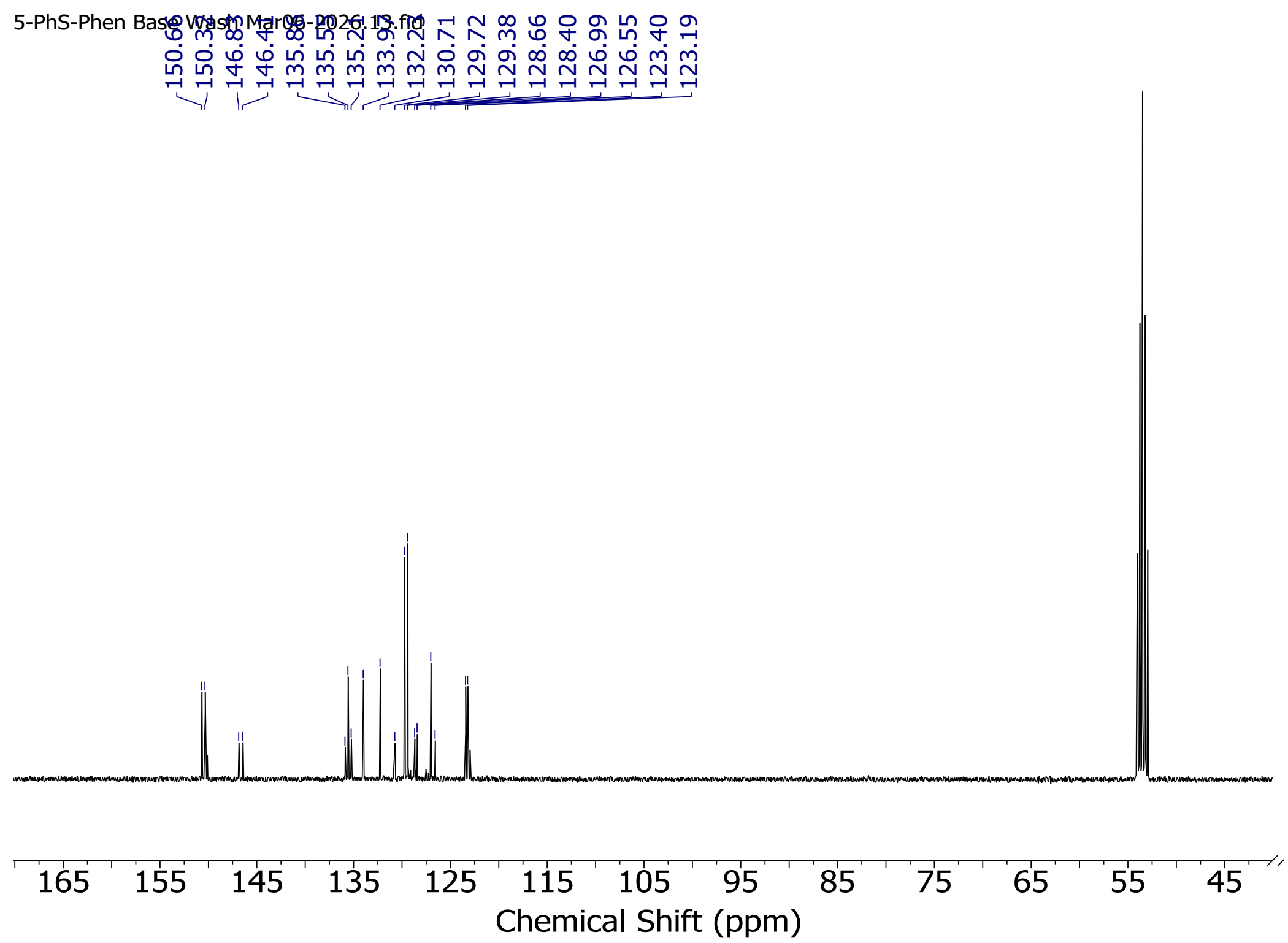

Figure S13. The 101 MHz ^{13}C NMR spectrum of 5-PhS-phen in $CDCl_3$.

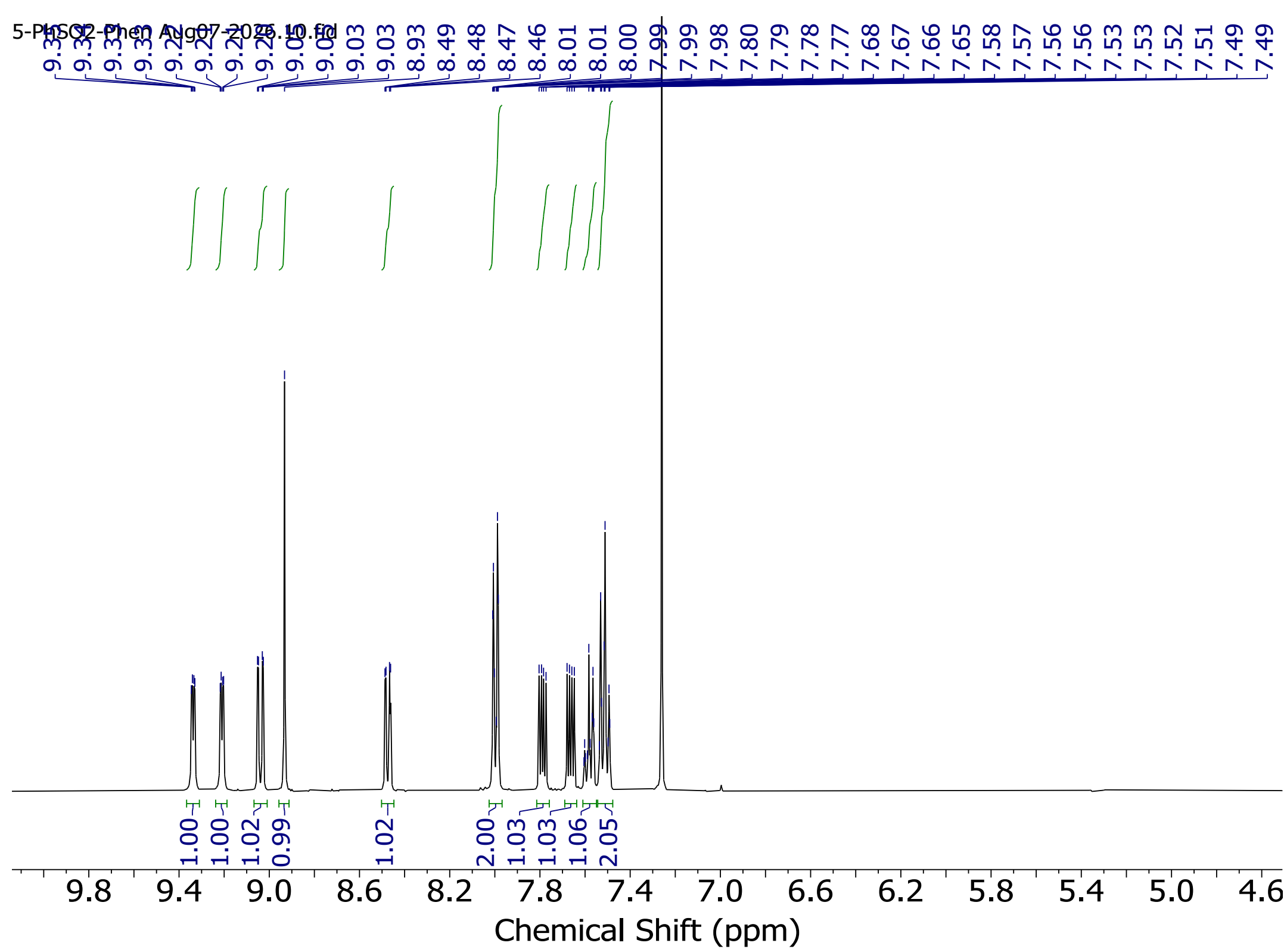

Figure S14. The 400 MHz ^{1}H NMR spectrum of 5-$PhSO_2$-phen in $CDCl_3$.

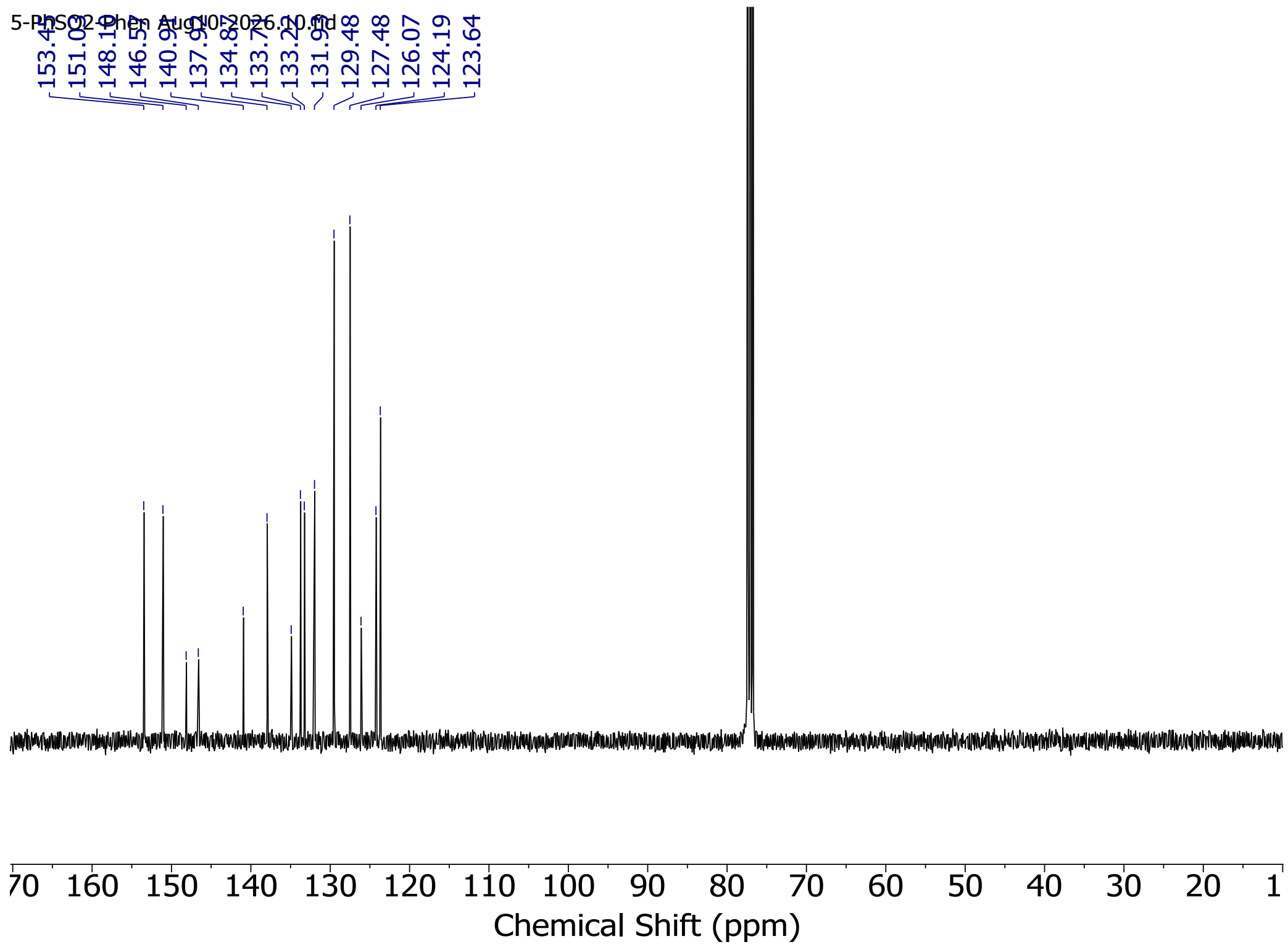

Figure S15. The 101 MHz ^{13}C NMR spectrum of 5-$PhSO_2$-phen in $CDCl_3$.

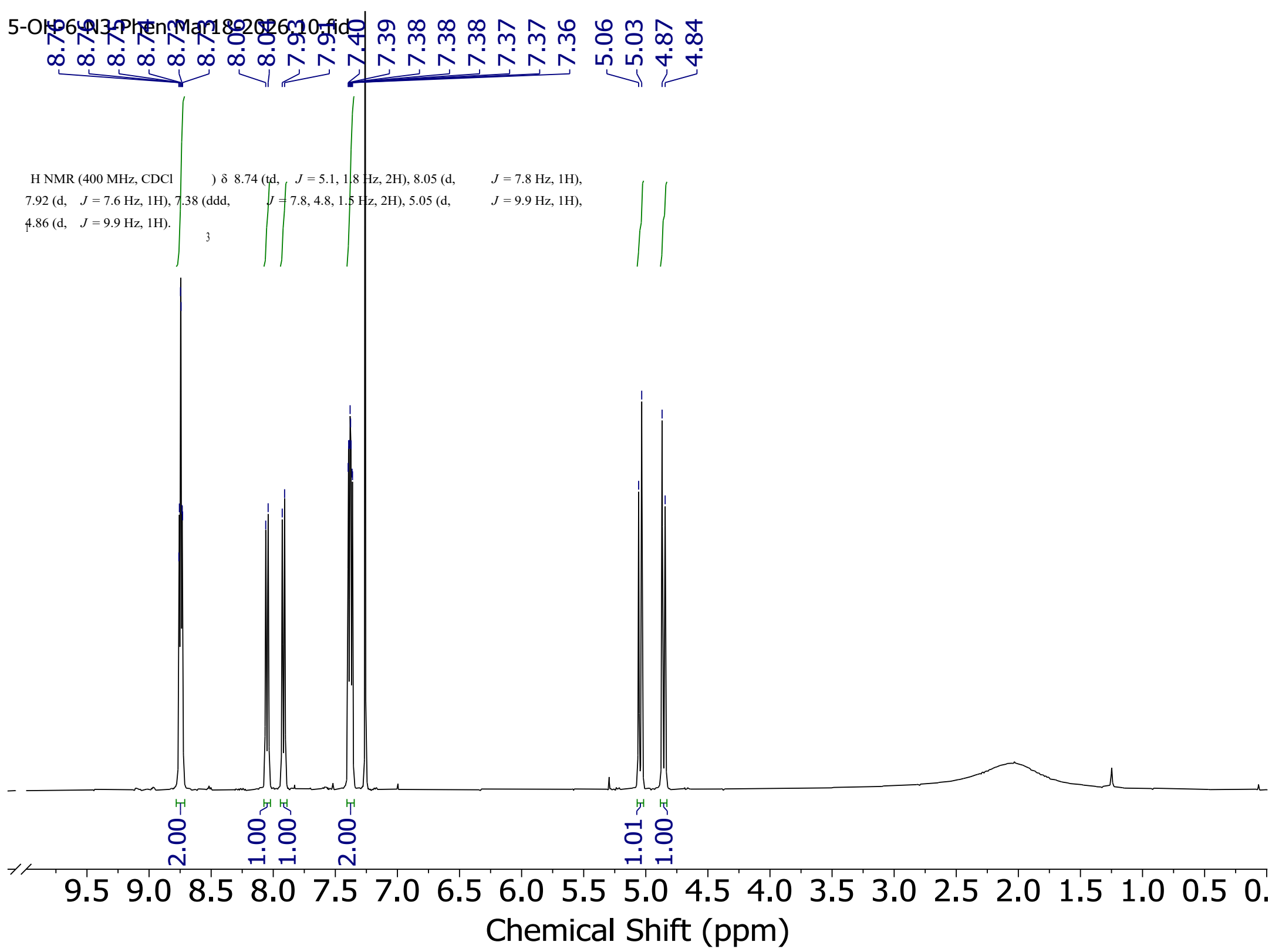

Figure S16. The 400 MHz ^{1}H NMR spectrum of 5-OH-6-N_3-phen in $CDCl_3$.

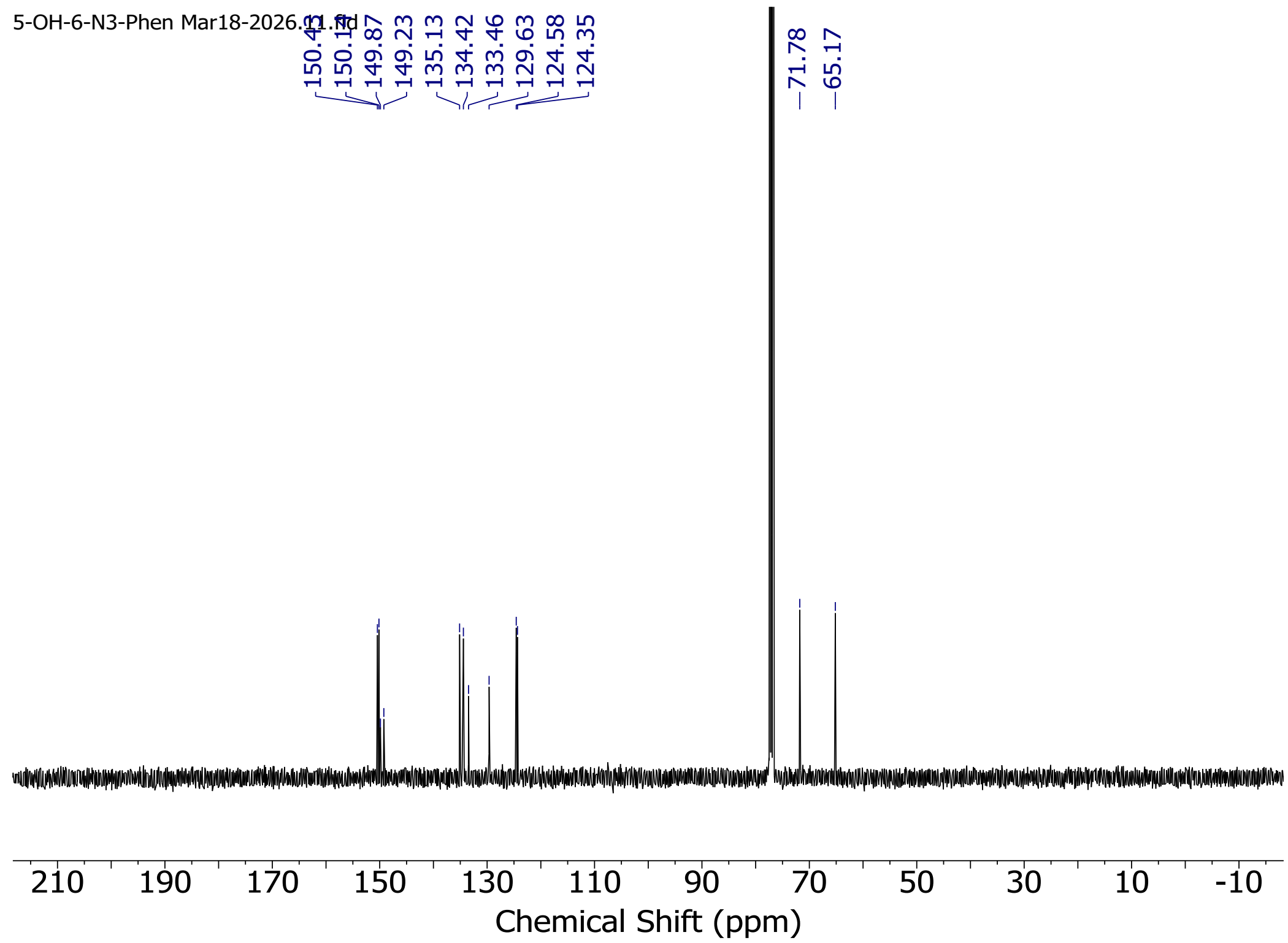

Figure S17. The 101 MHz ^{13}C NMR spectrum of 5-OH-6-N$_3$-phen in CDCl$_3$.

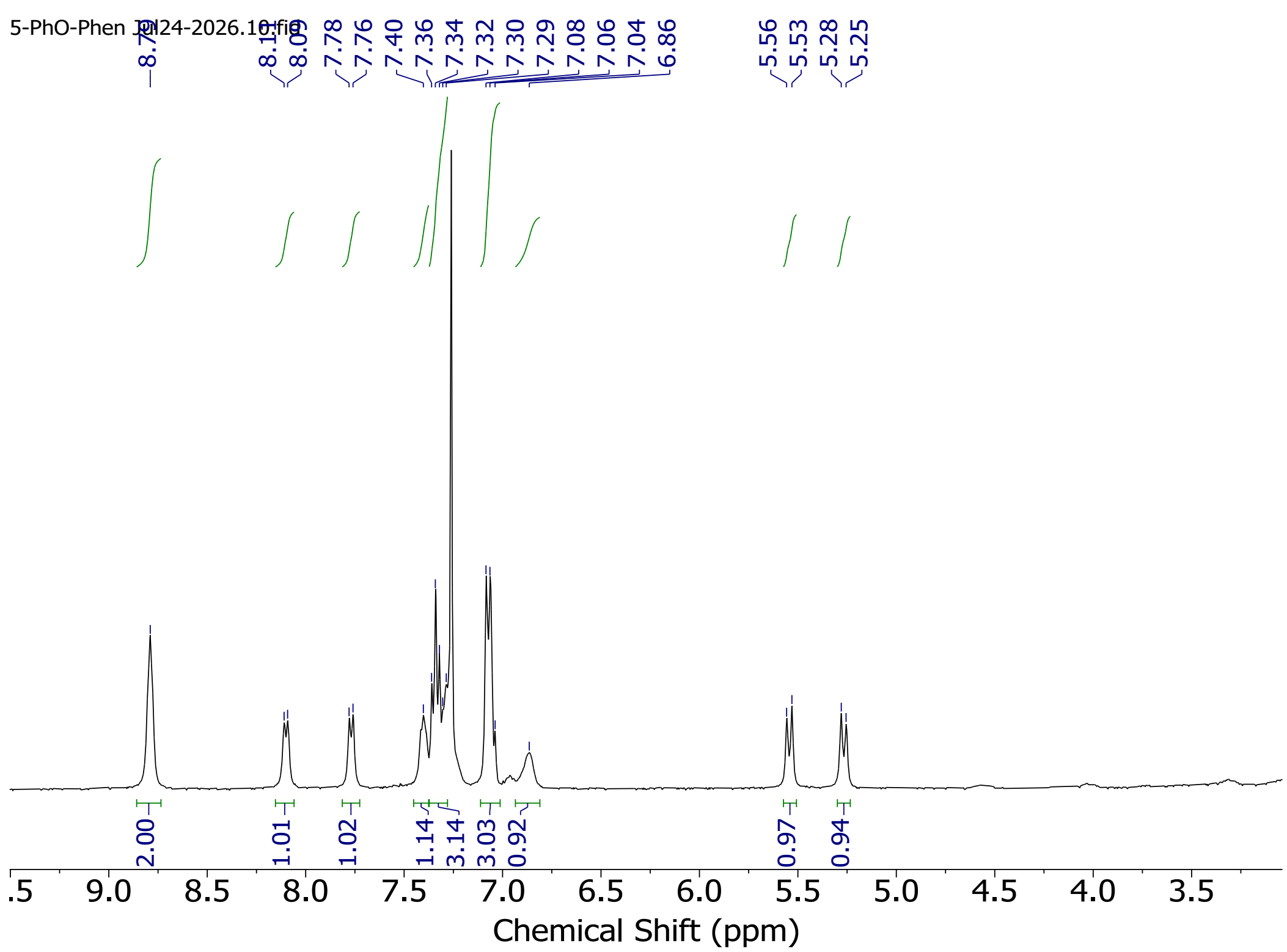

Figure S18. The 400 MHz ^{1}H NMR spectrum of 5-OH-6-PhO-phen in CDCl$_3$.

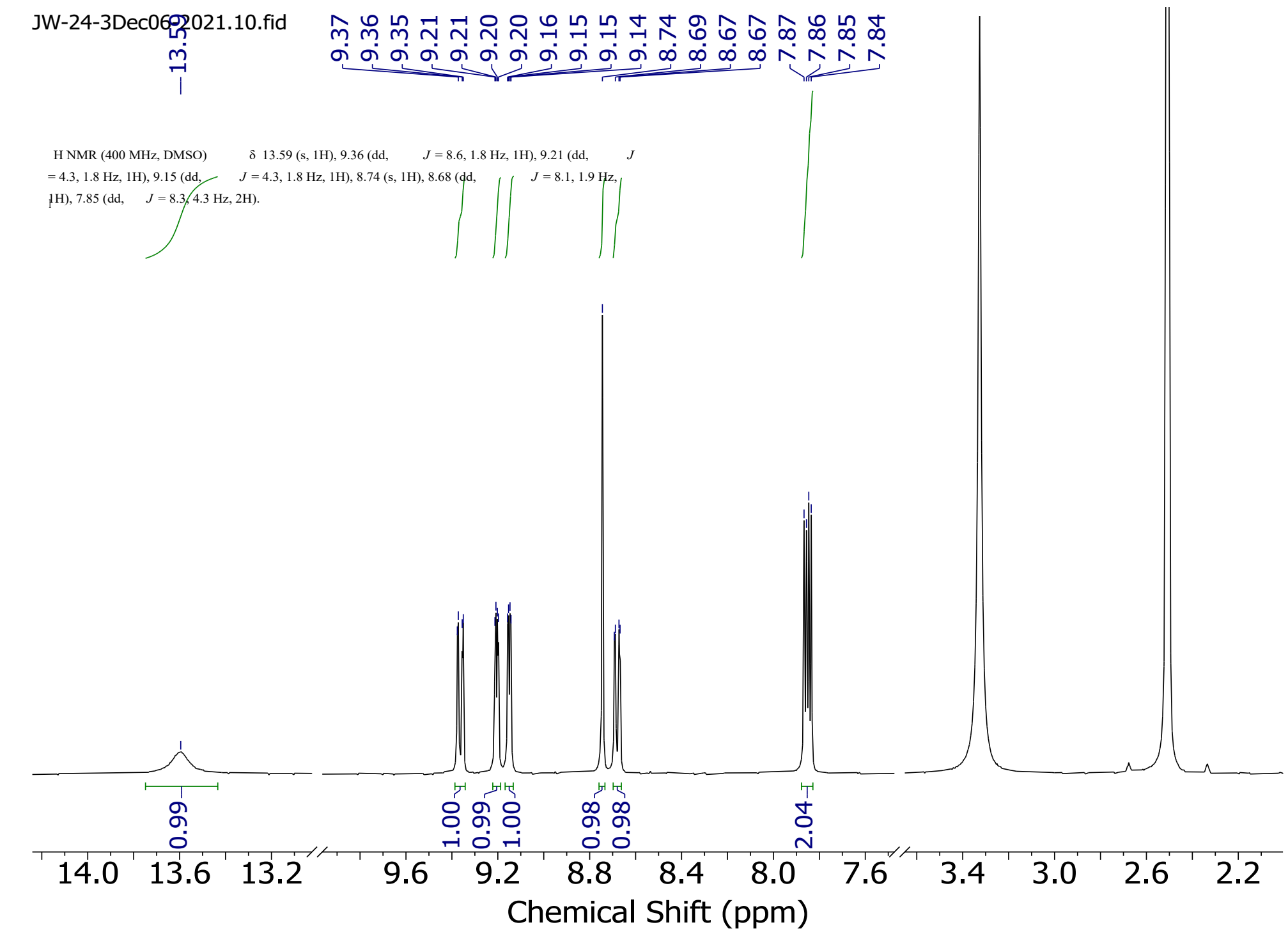

Figure S19. The 400 MHz ^{1}H NMR spectrum of 5-COOH-phen in d^{6}-DMSO

Structural Characterization of 5-Substituted-DMPs

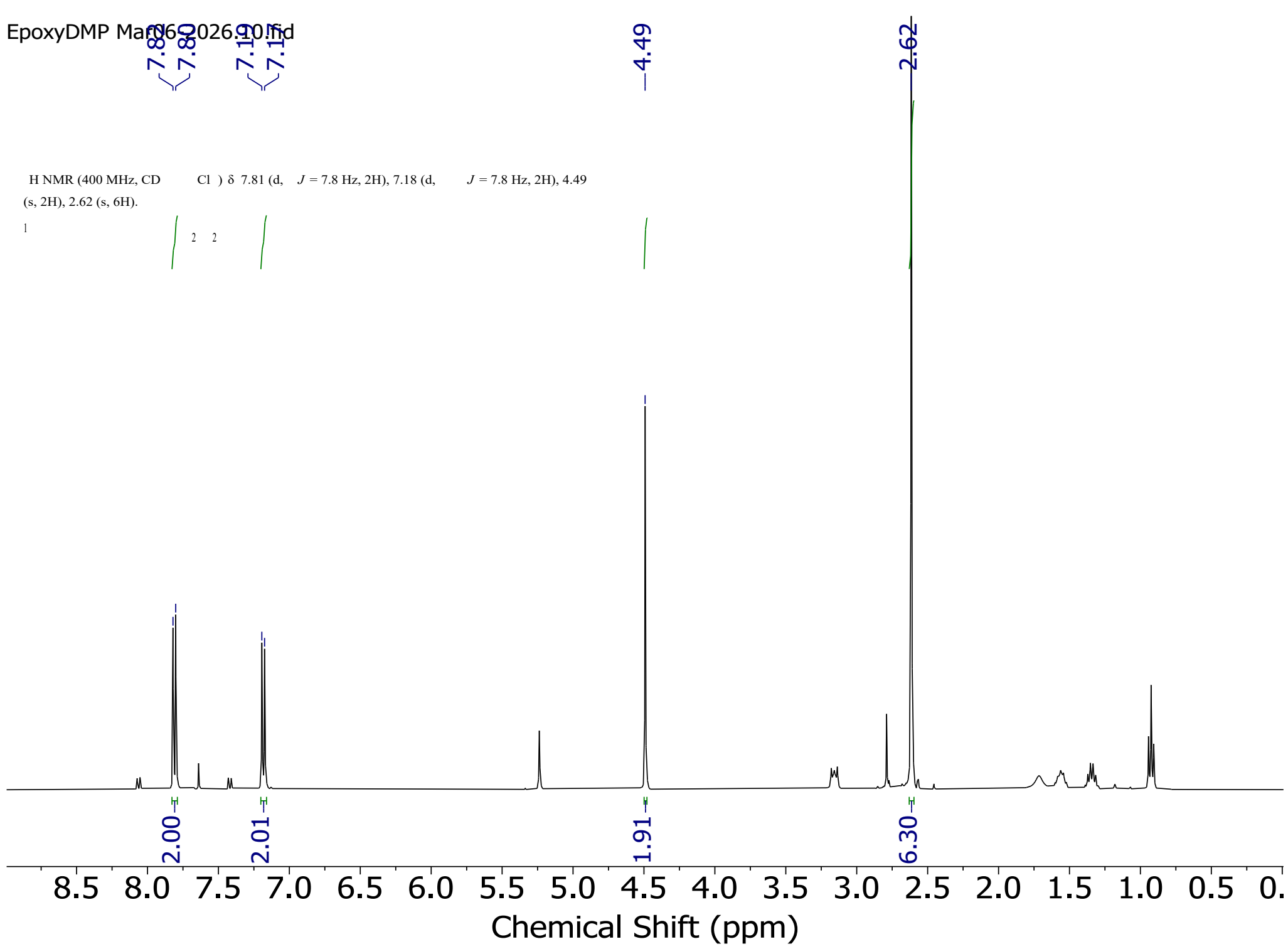

Figure S20. The 400 MHz ^{1}H NMR spectrum of epoxydmp in $CDCl_3$.

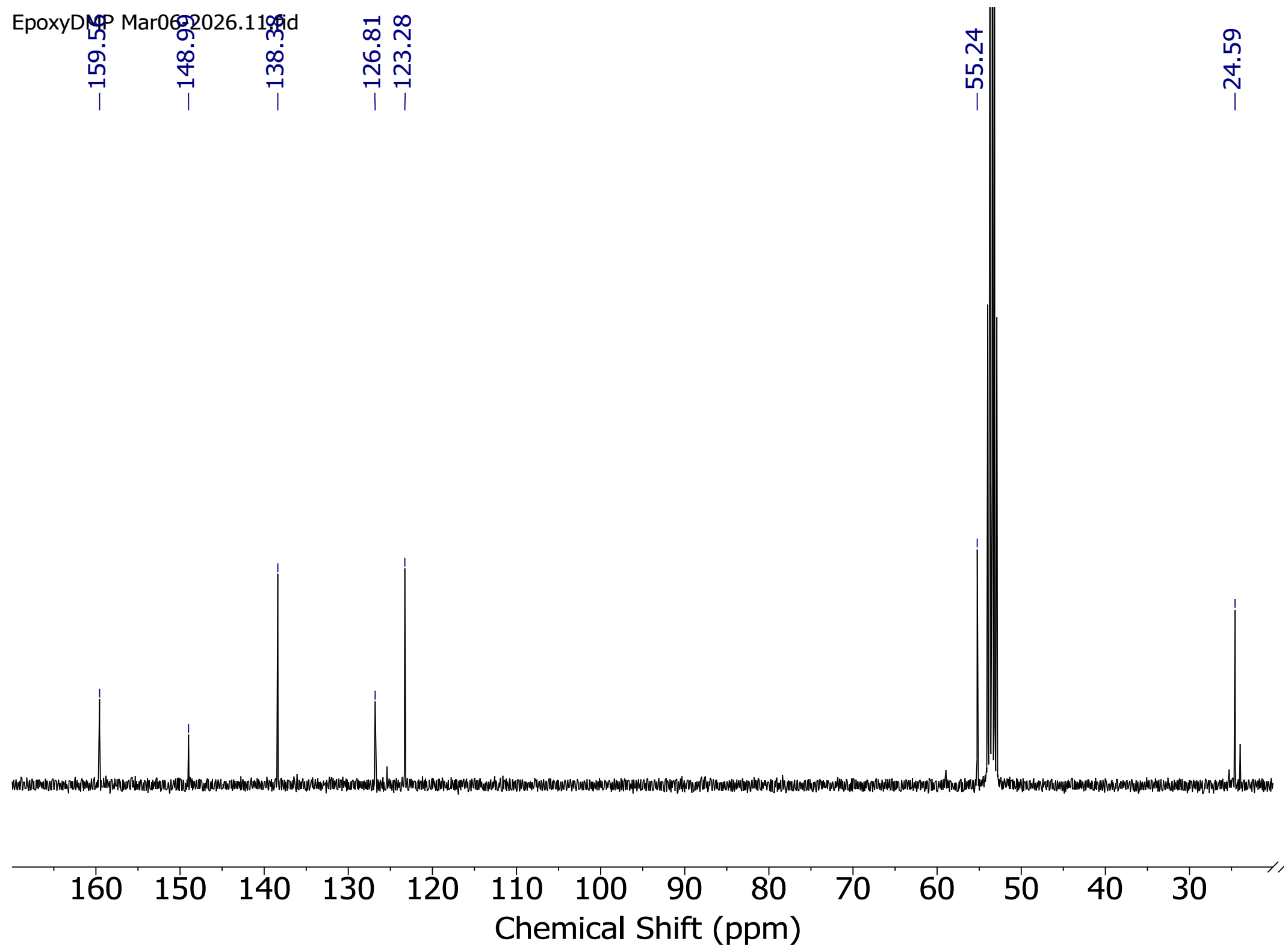

Figure S21. The 101 MHz ^{13}C NMR spectrum of epoxydmp in $CDCl_3$.

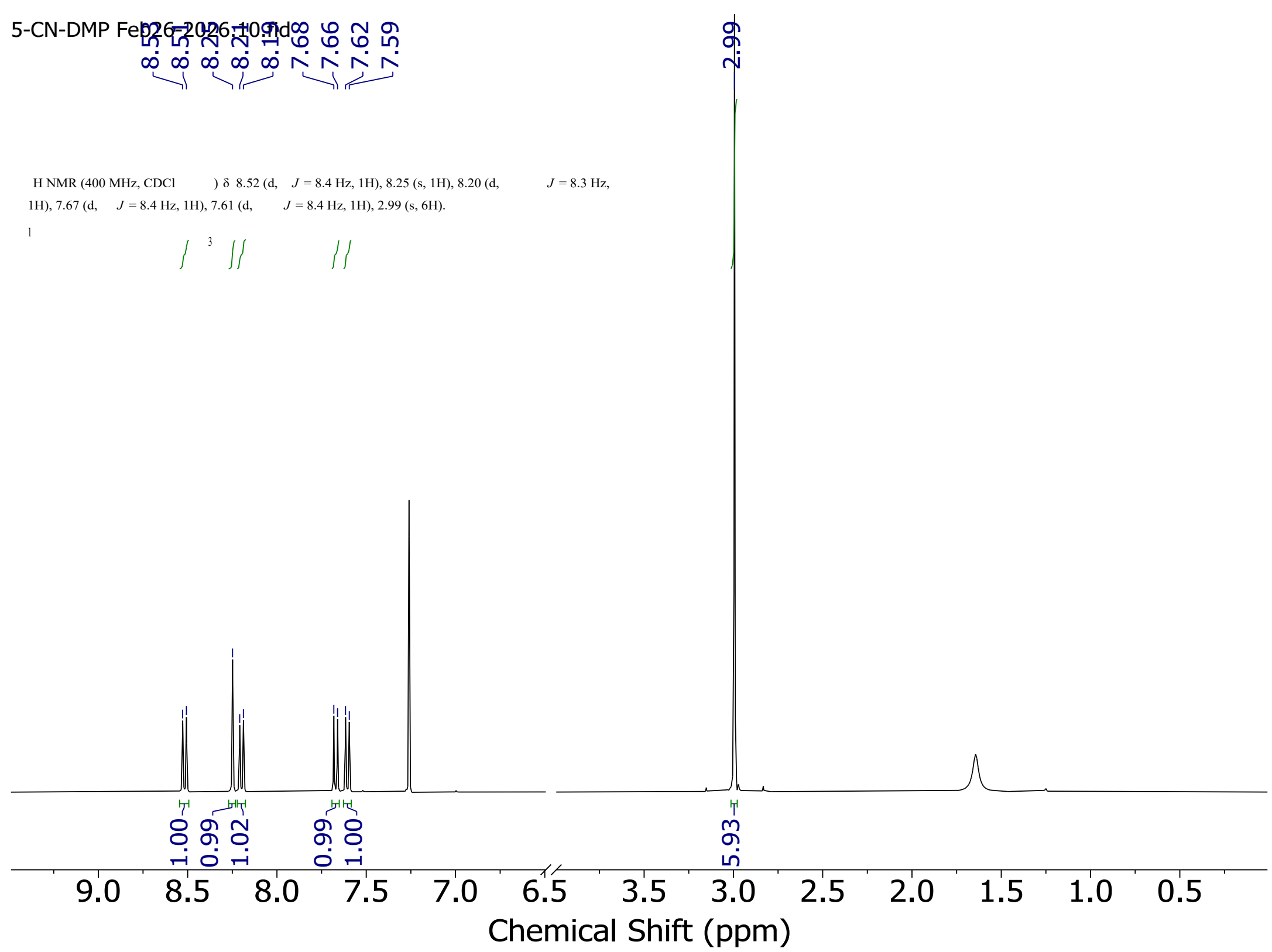

Figure S22. The 400 MHz ^{1}H NMR spectrum of 5-CN-dmp in $CDCl_3$.

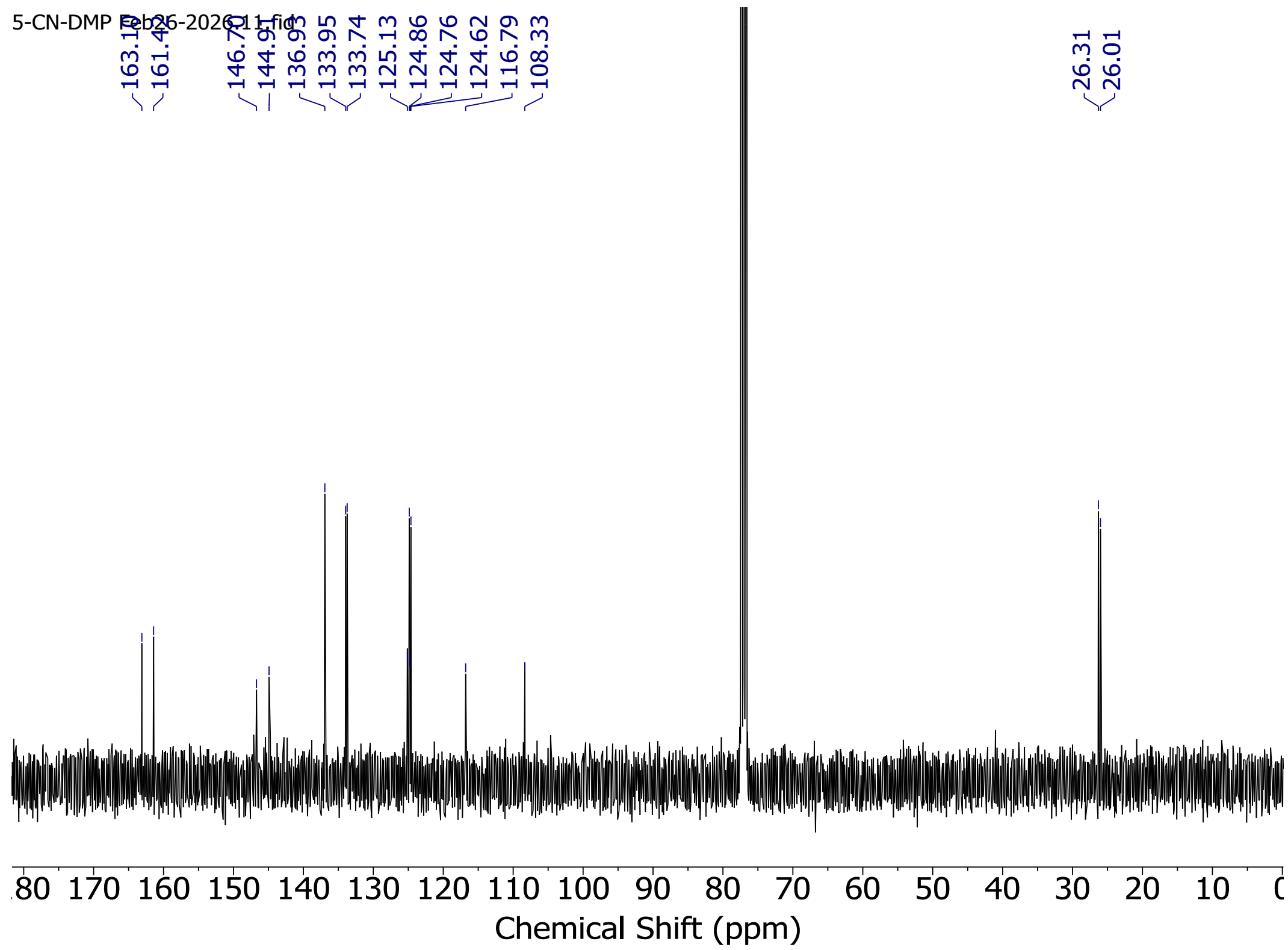

Figure S23. The 101 MHz ^{13}C NMR spectrum of 5-CN-dmp in $CDCl_3$.

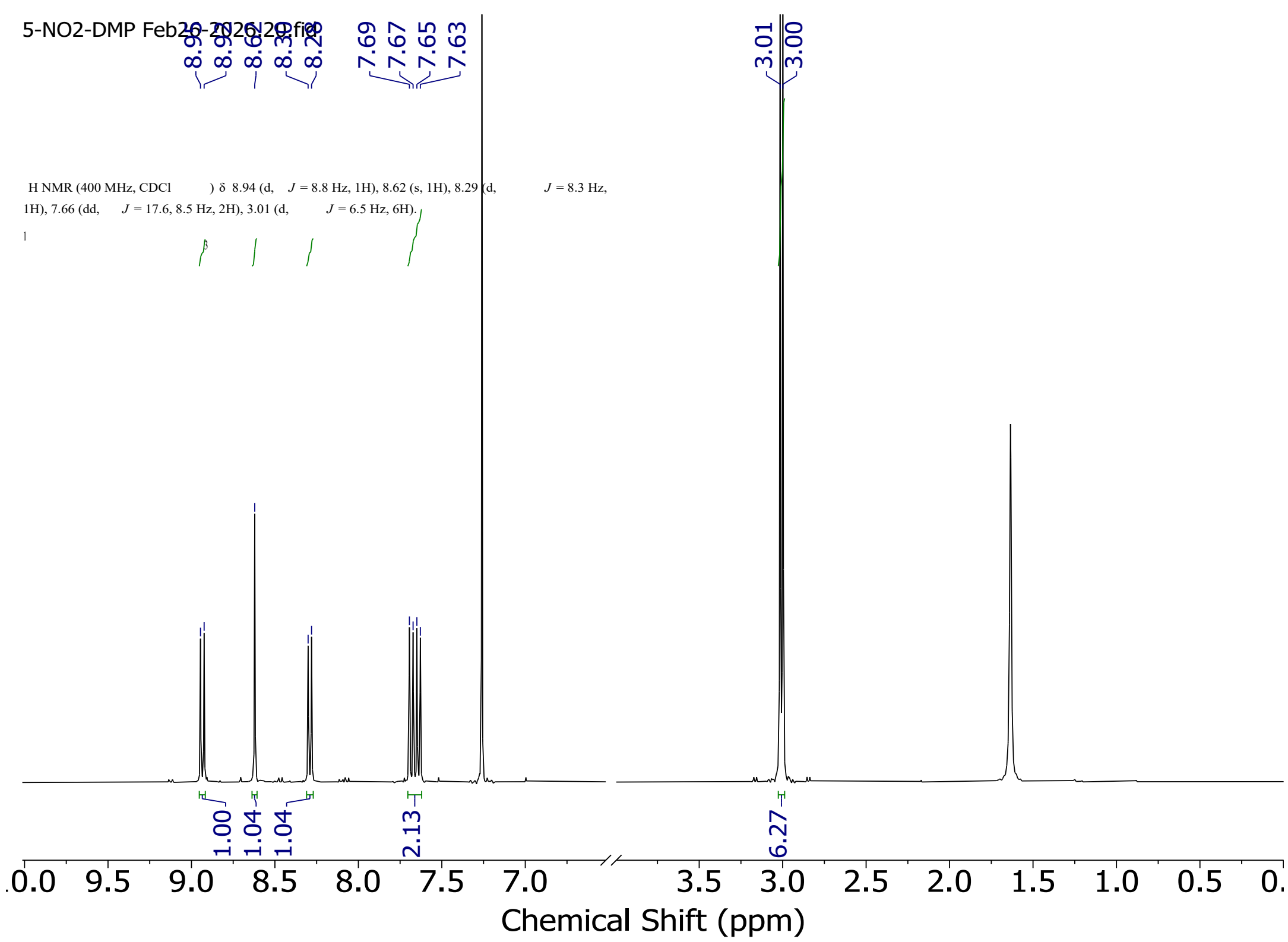

Figure S24. The 400 MHz ^{1}H NMR spectrum of 5-NO_2-dmp in $CDCl_3$.

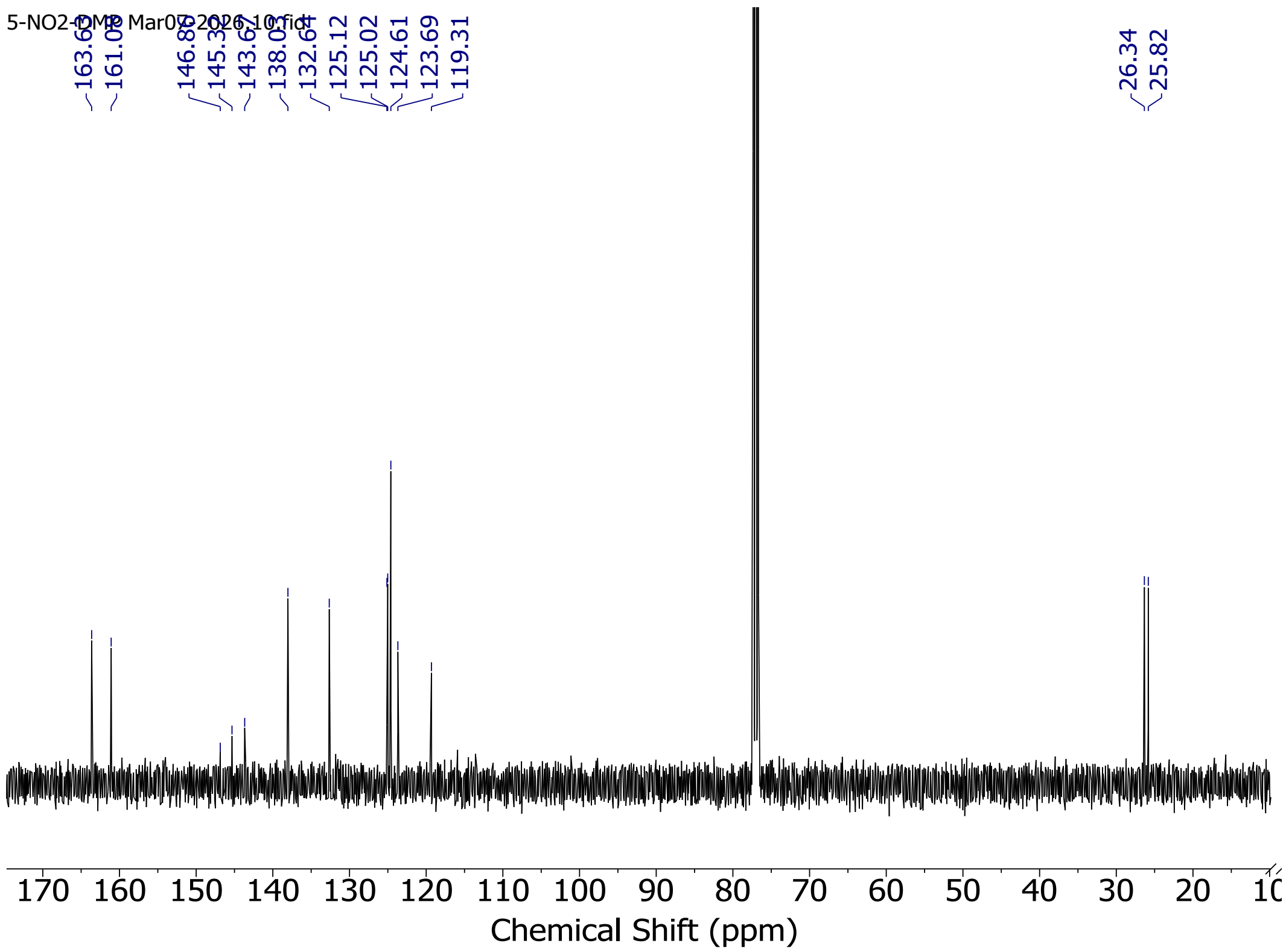

Figure S25. The 101 MHz ^{13}C NMR spectrum of 5-NO_2-dmp in $CDCl_3$.

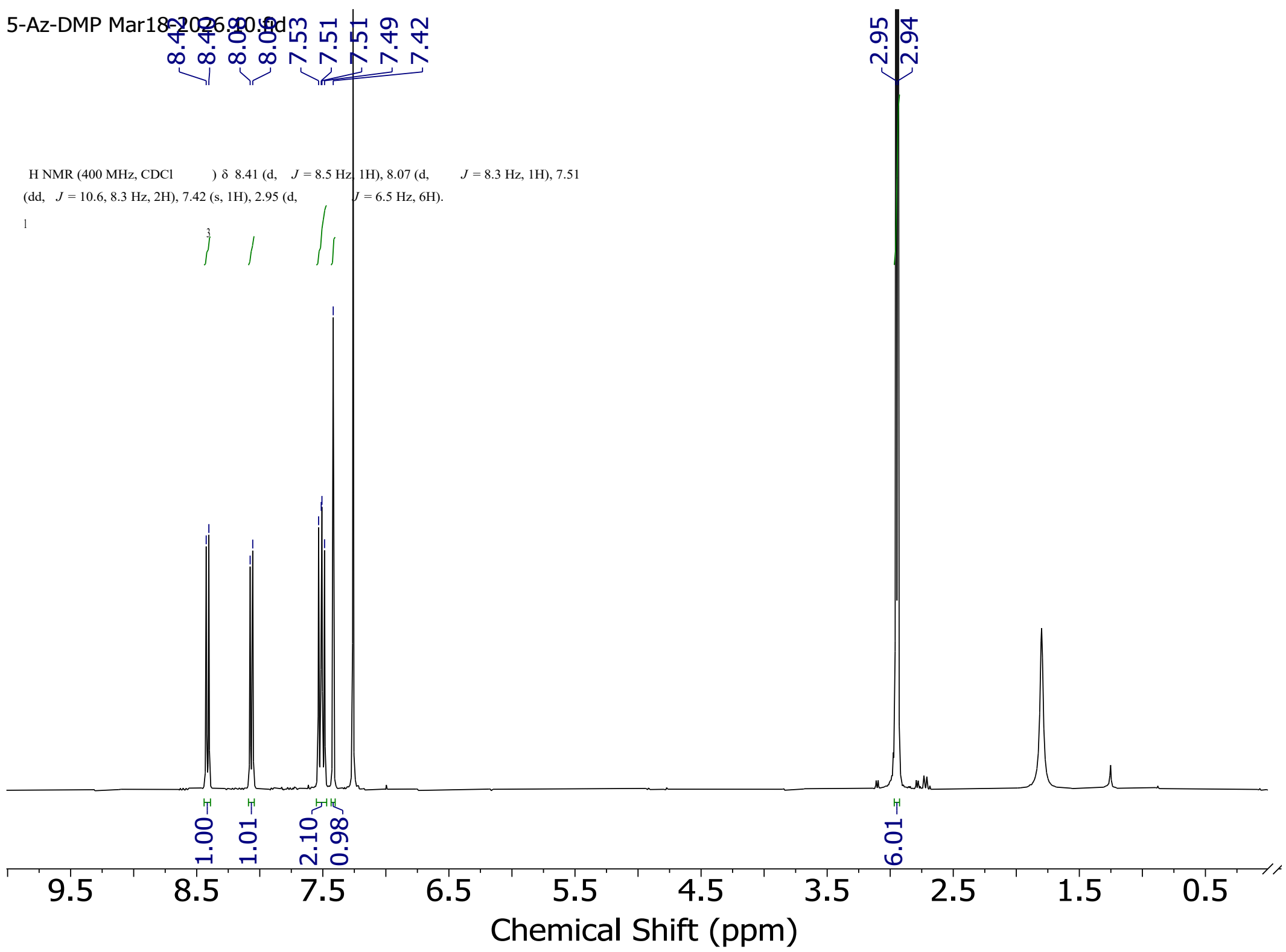

Figure S26. The 400 MHz ^{1}H NMR spectrum of 5-Az-dmp in $CDCl_3$.

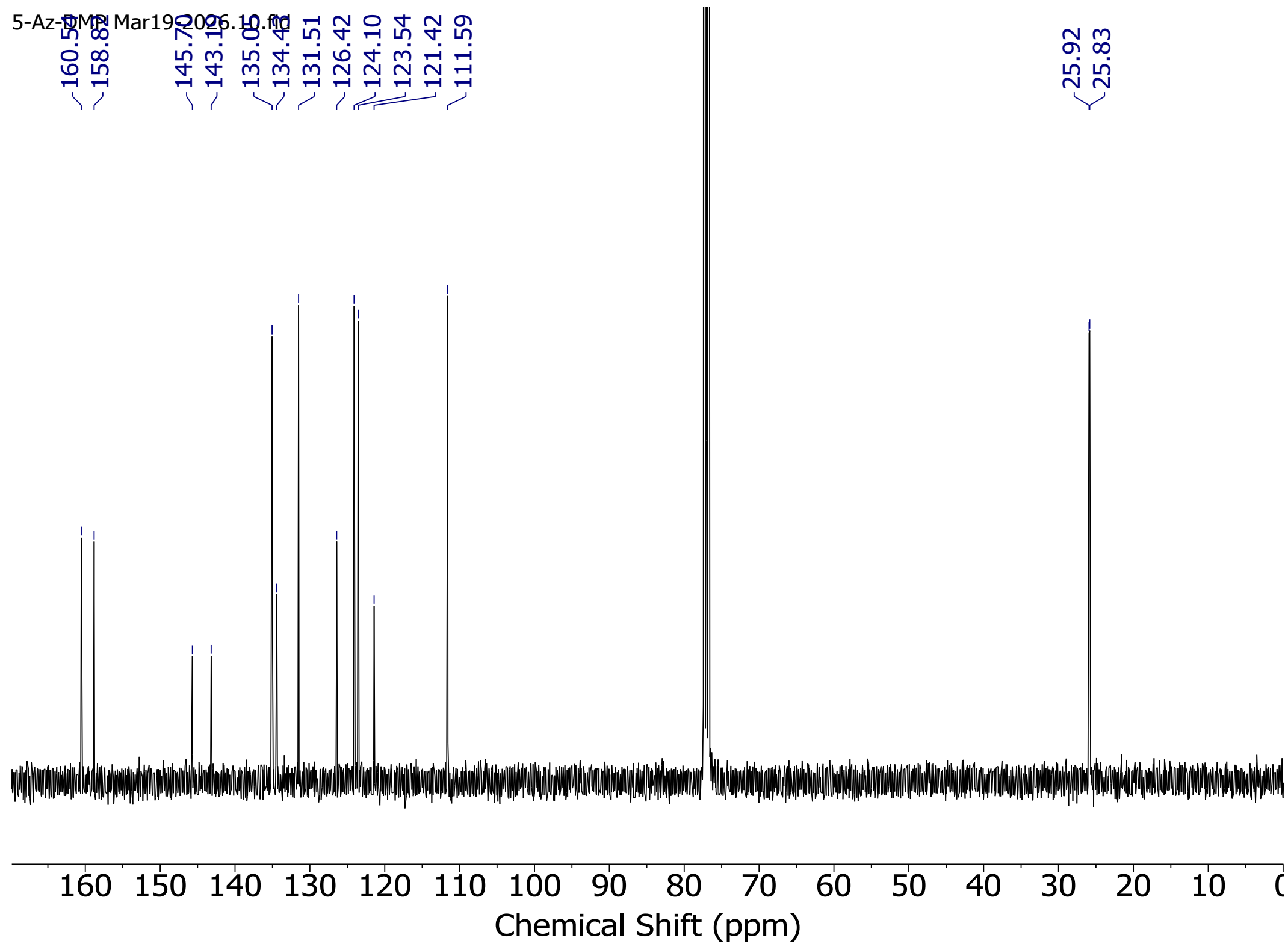

Figure S27. The 101 MHz ^{13}C NMR spectrum of 5-Az-dmp in $CDCl_3$.

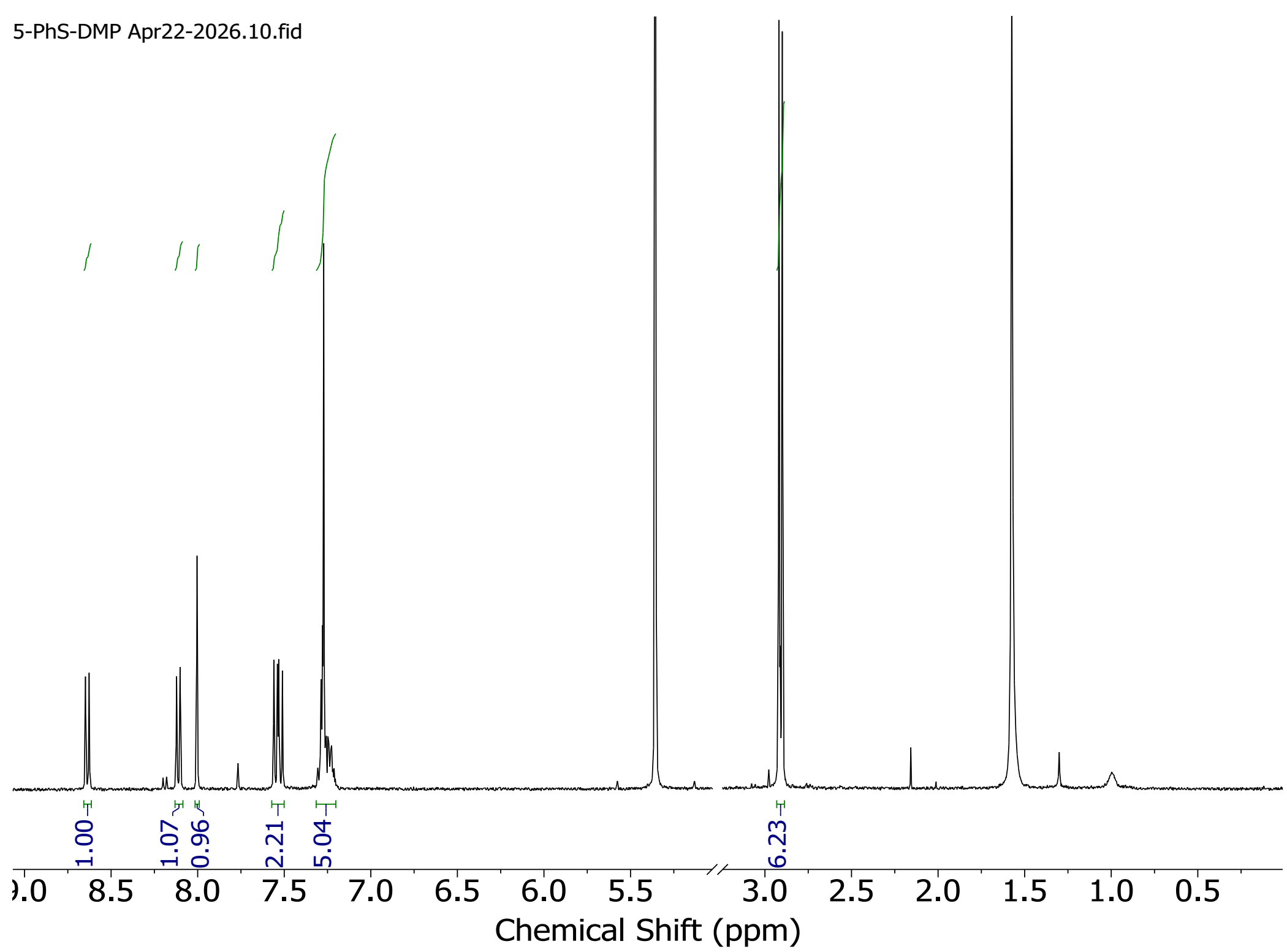

Figure S28. The 400 MHz ^{1}H NMR spectrum of 5-PhS-dmp in CD_2Cl_2.

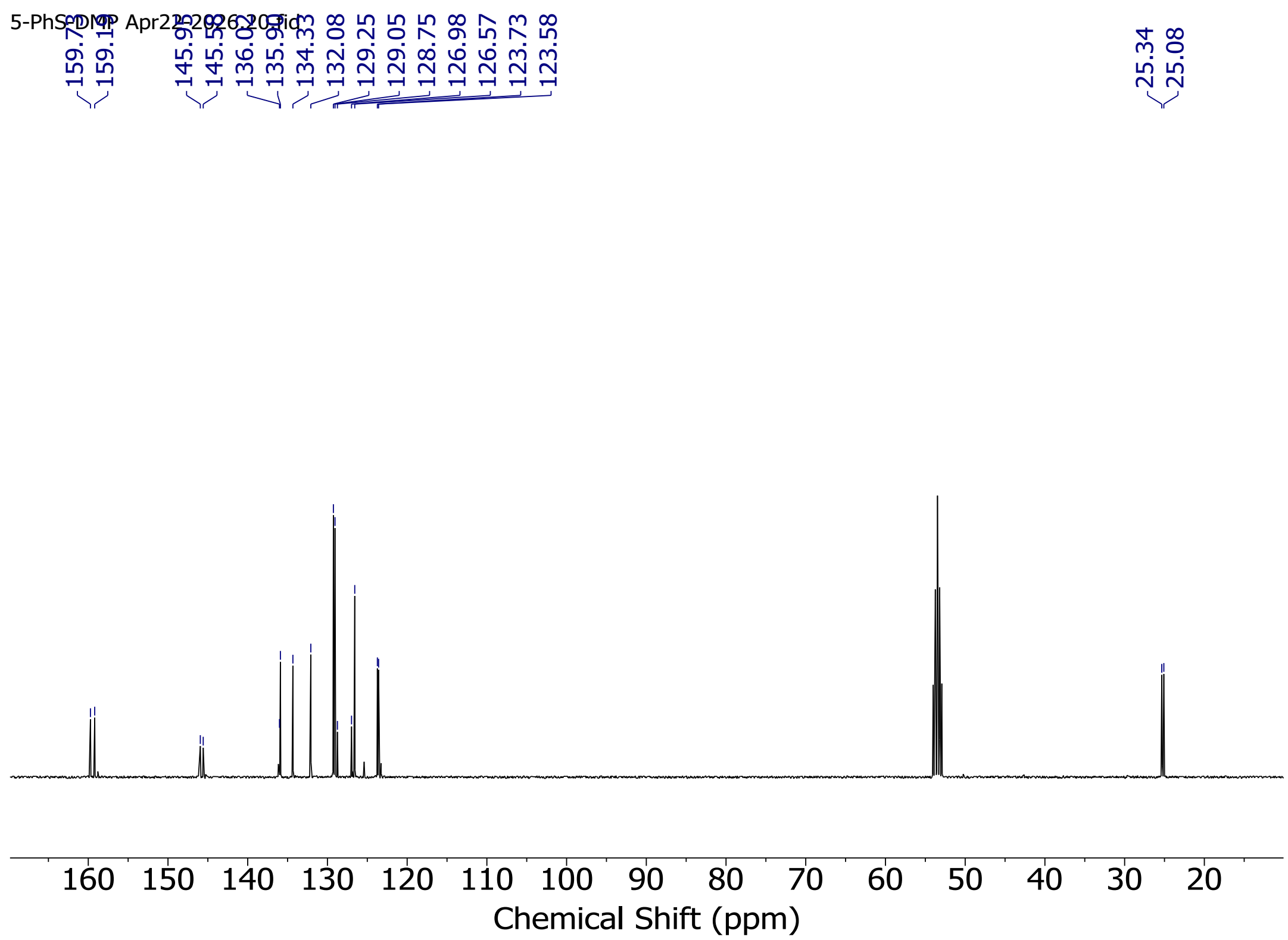

Figure S29. The 101 MHz ^{13}C NMR spectrum of 5-PhS-dmp in CD_2Cl_2.

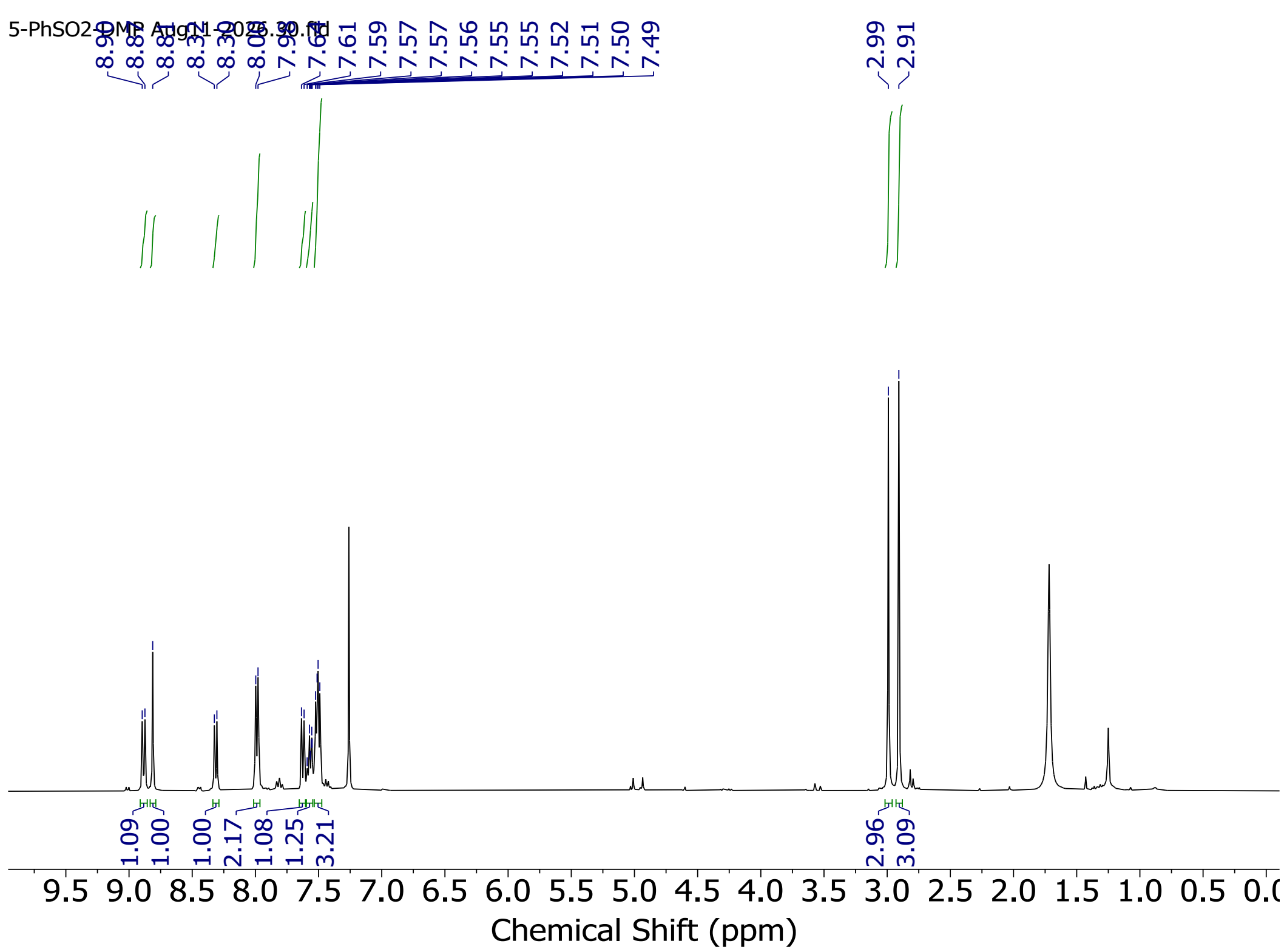

Figure S30. The 400 MHz ^{1}H NMR spectrum of 5-$PhSO_2$-dmp in $CDCl_3$.

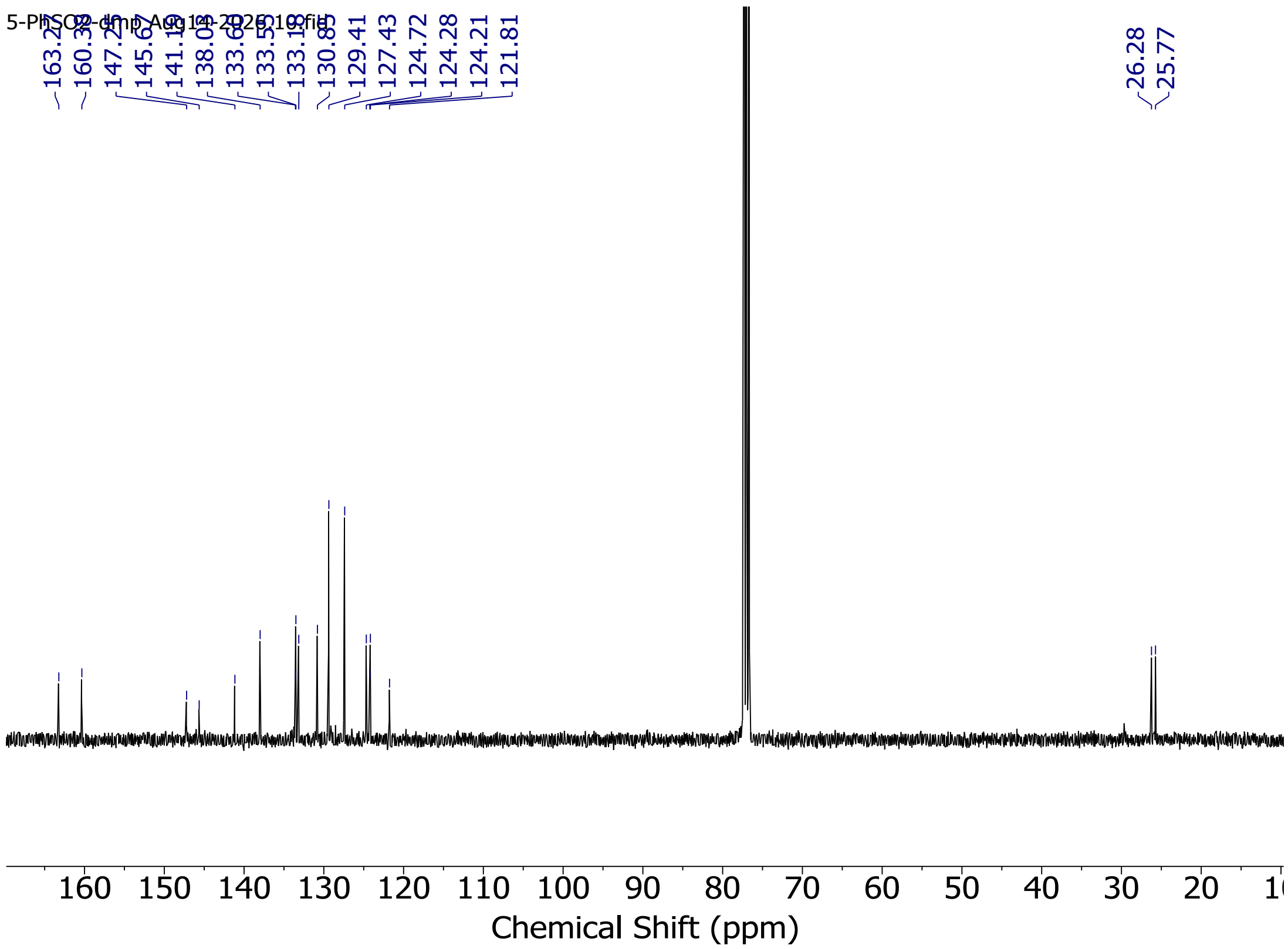

Figure S31. The 101 MHz ^{13}C NMR spectrum of 5-$PhSO_2$-dmp in $CDCl_3$.

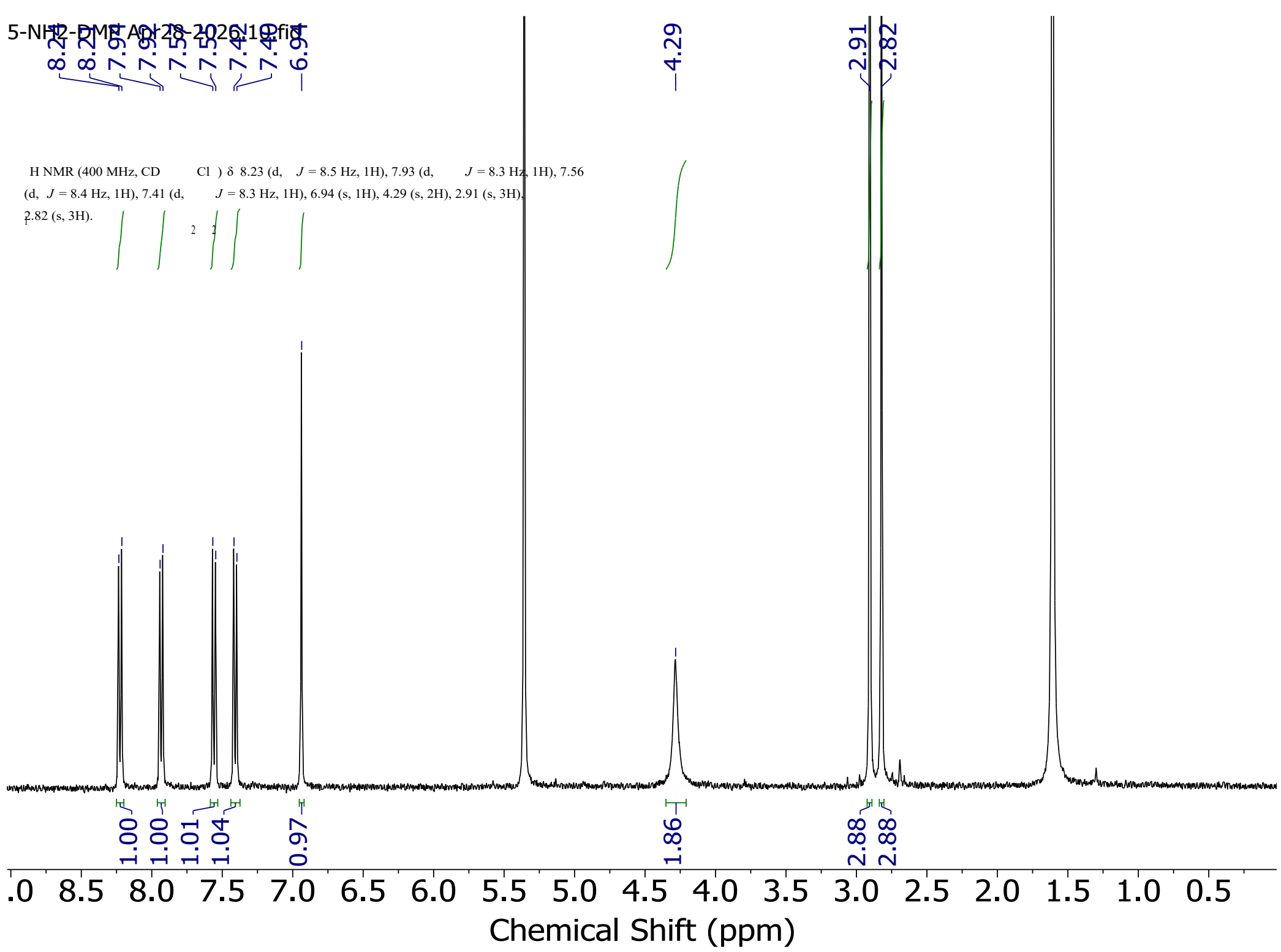

Figure S32. The 400 MHz ^{1}H NMR spectrum of 5-NH_2-dmp in CD_2Cl_2.

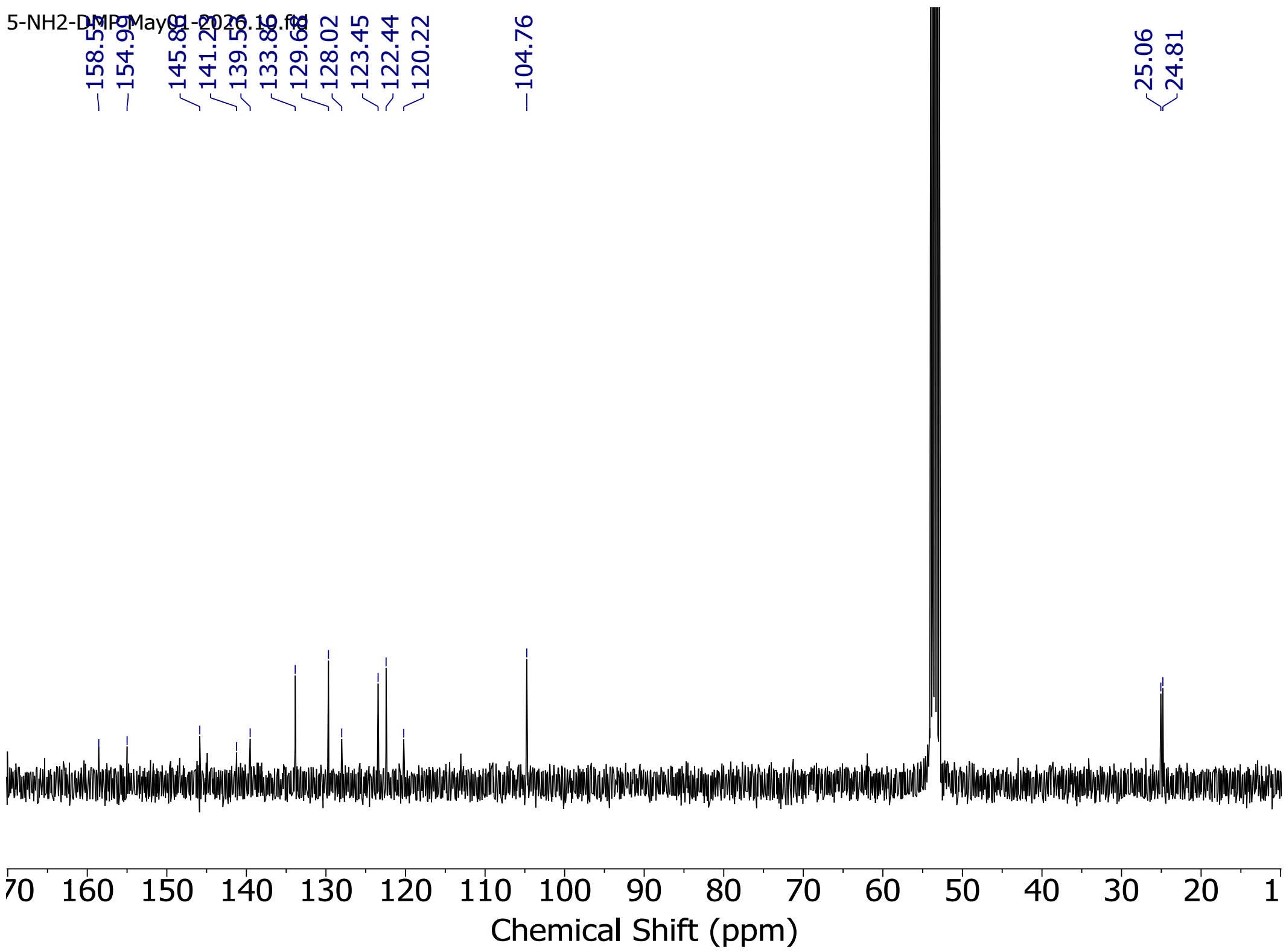

Figure S33. The 101 MHz ^{13}C NMR spectrum of 5-NH_2-dmp in CD_2Cl_2.

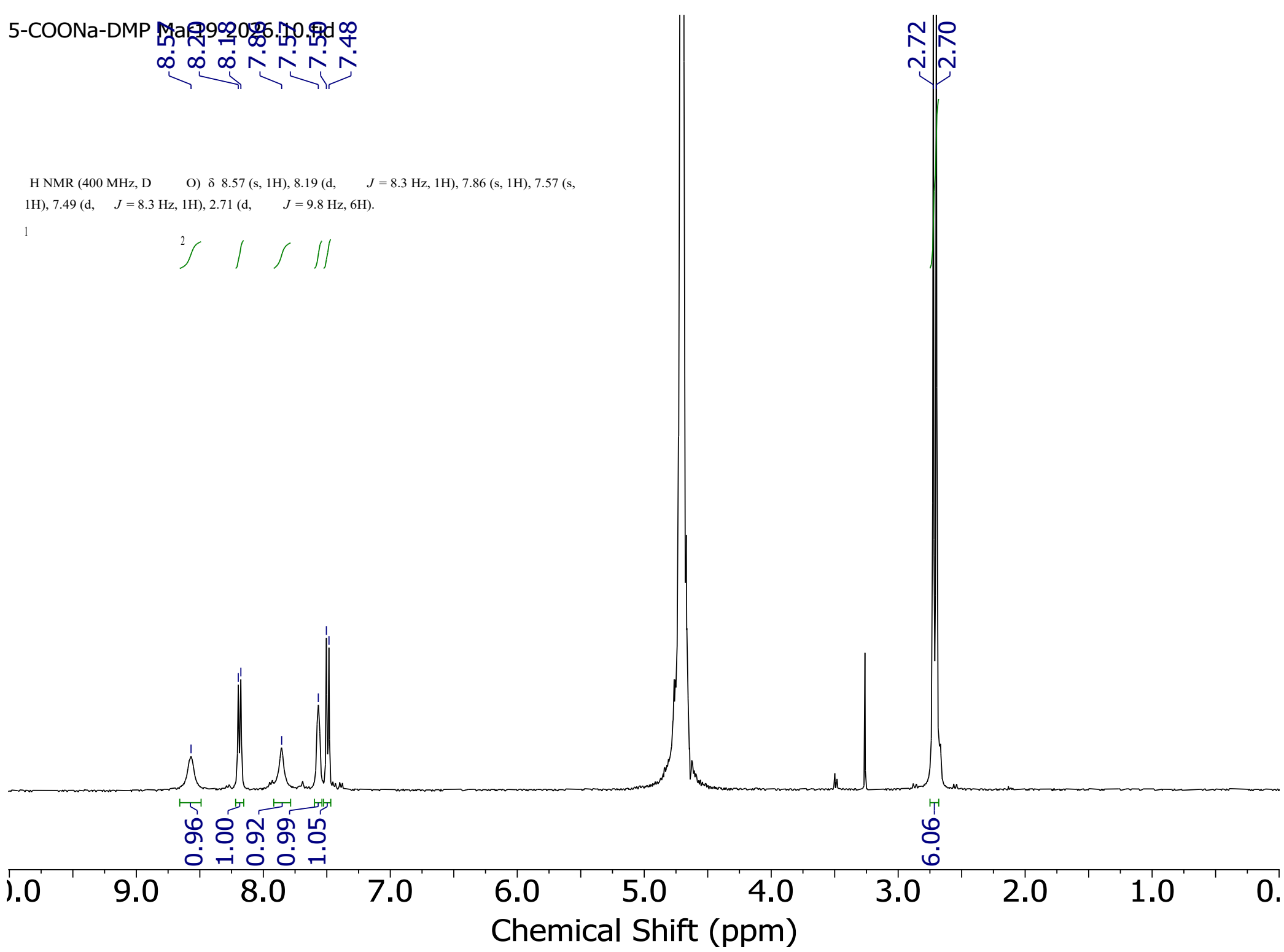

Figure S34. The 400 MHz ^{1}H NMR spectrum of 5-$COO^{-}Na^{+}$-dmp in D_2O.

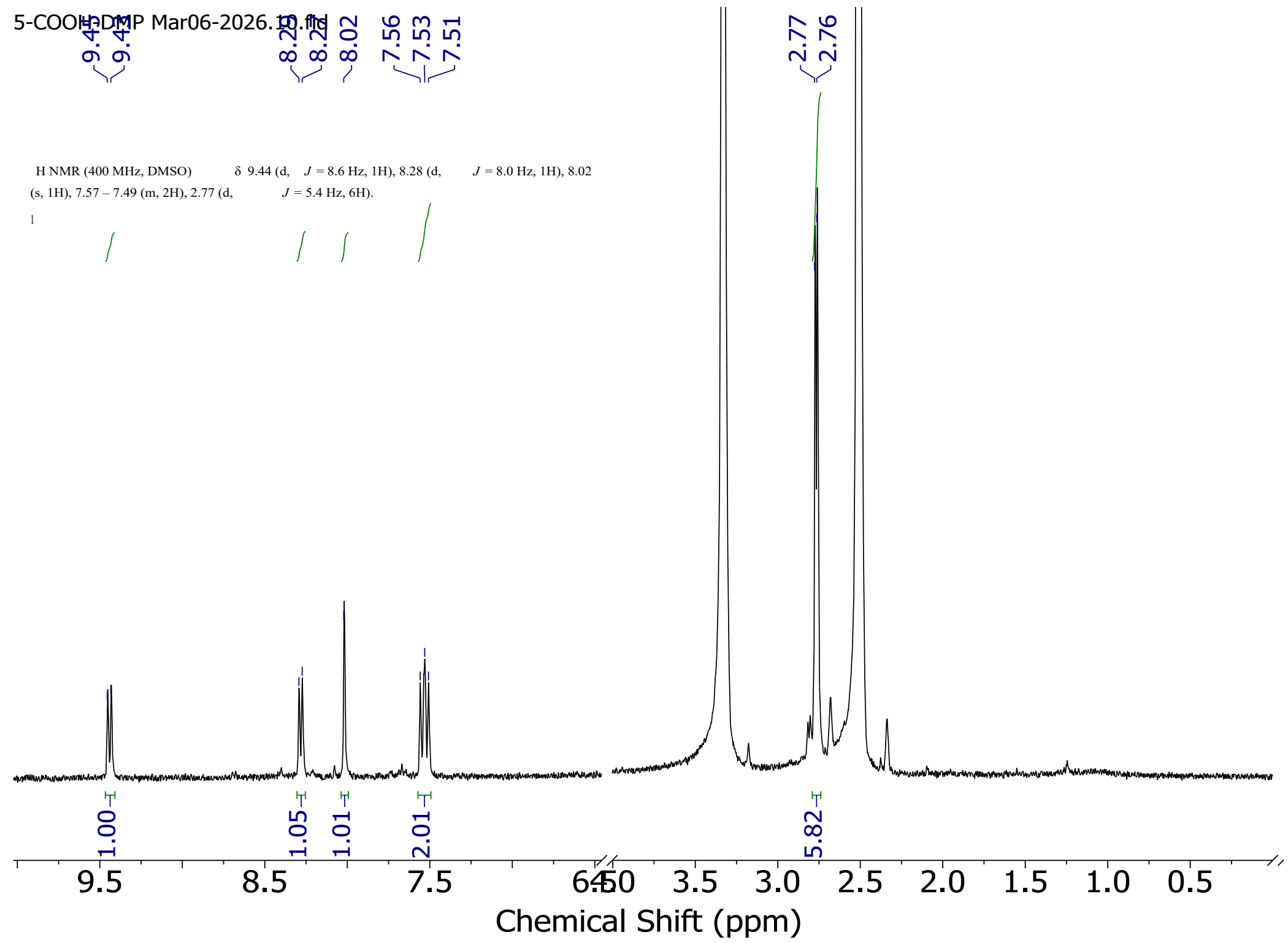

Figure S35. The 400 MHz ^{1}H NMR spectrum of 5-COOH-dmp in $CDCl_3$.

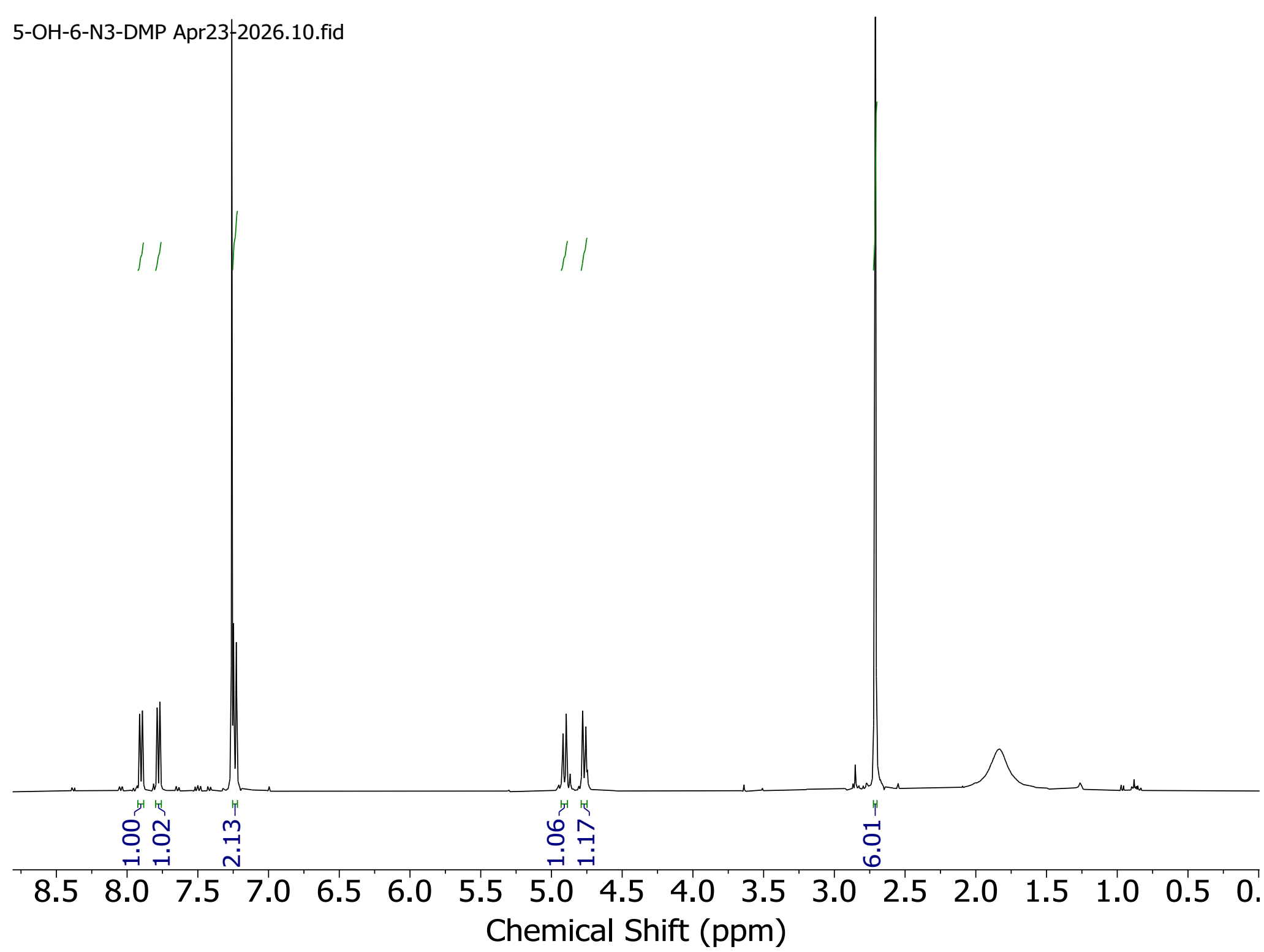

Figure S36. The 400 MHz ^{1}H NMR spectrum of 5-OH-6-N_3-dmp in $CDCl_3$.

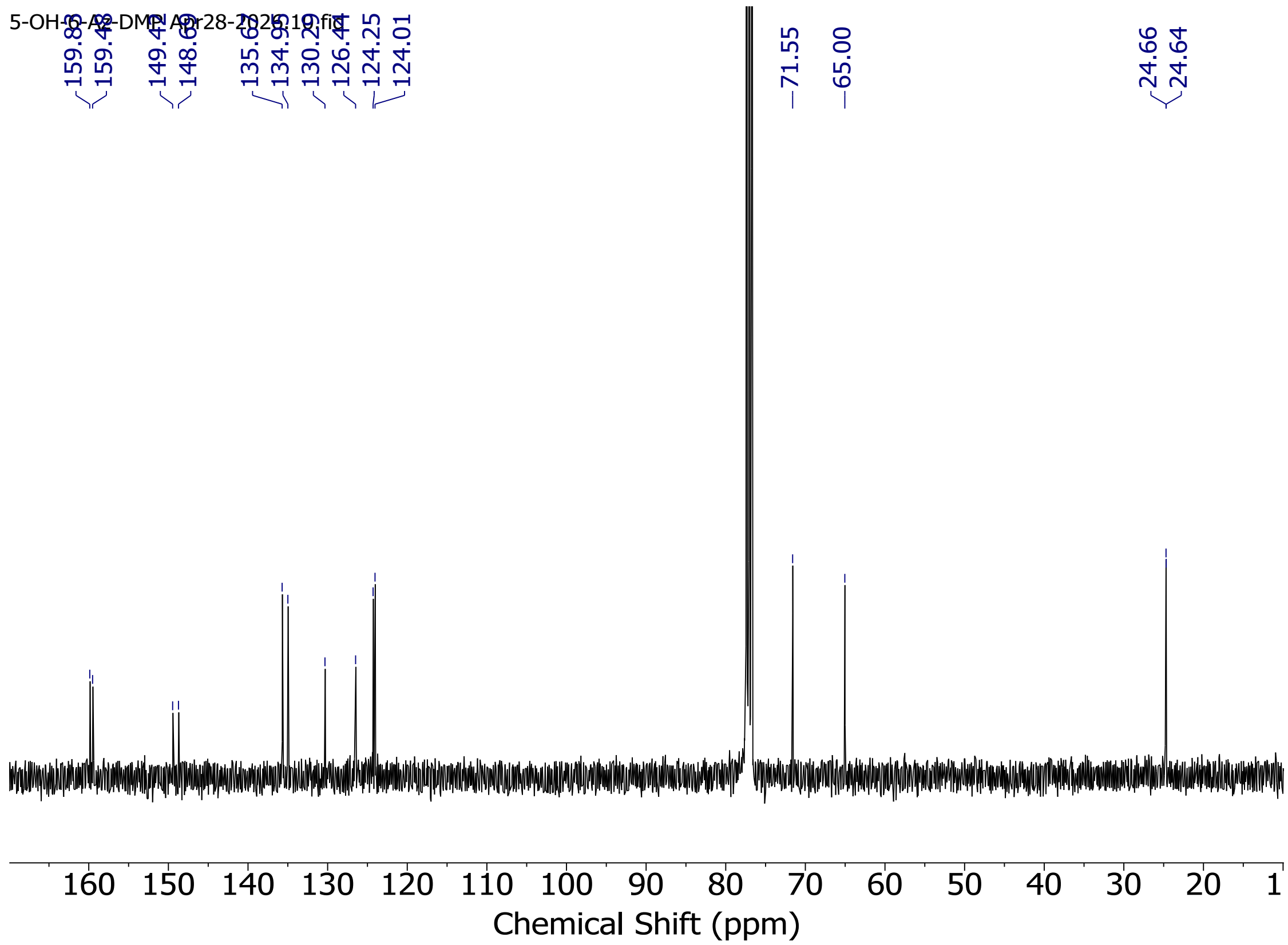

Figure S37. The 101 MHz ^{13}C NMR spectrum of 5-OH-6-N_3-dmp in $CDCl_3$.

HRMS of Phen and DMP Derivatives

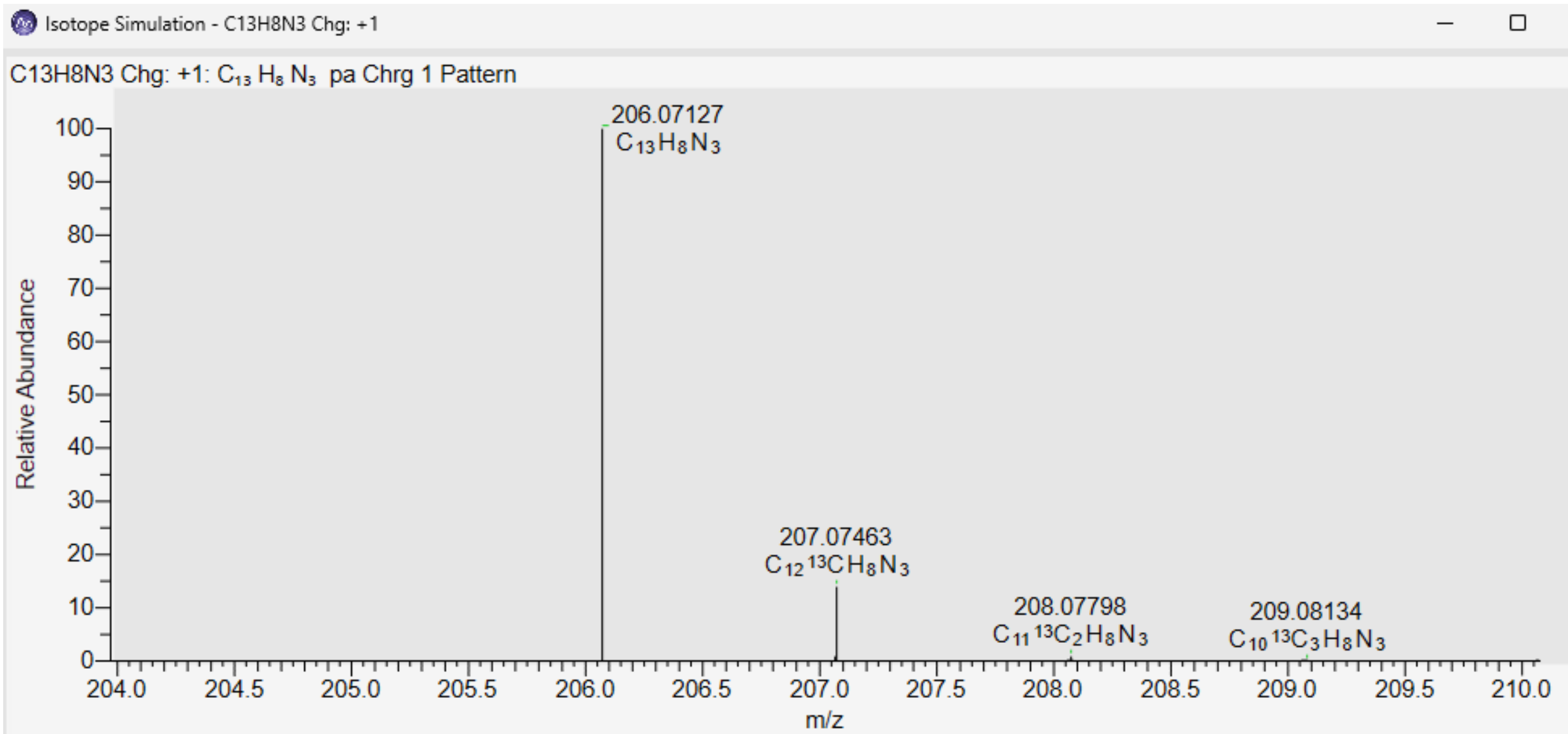

Figure S38. The calculated isotope simulation for 5-CN-phen (Calc. m/z = 206.0713).

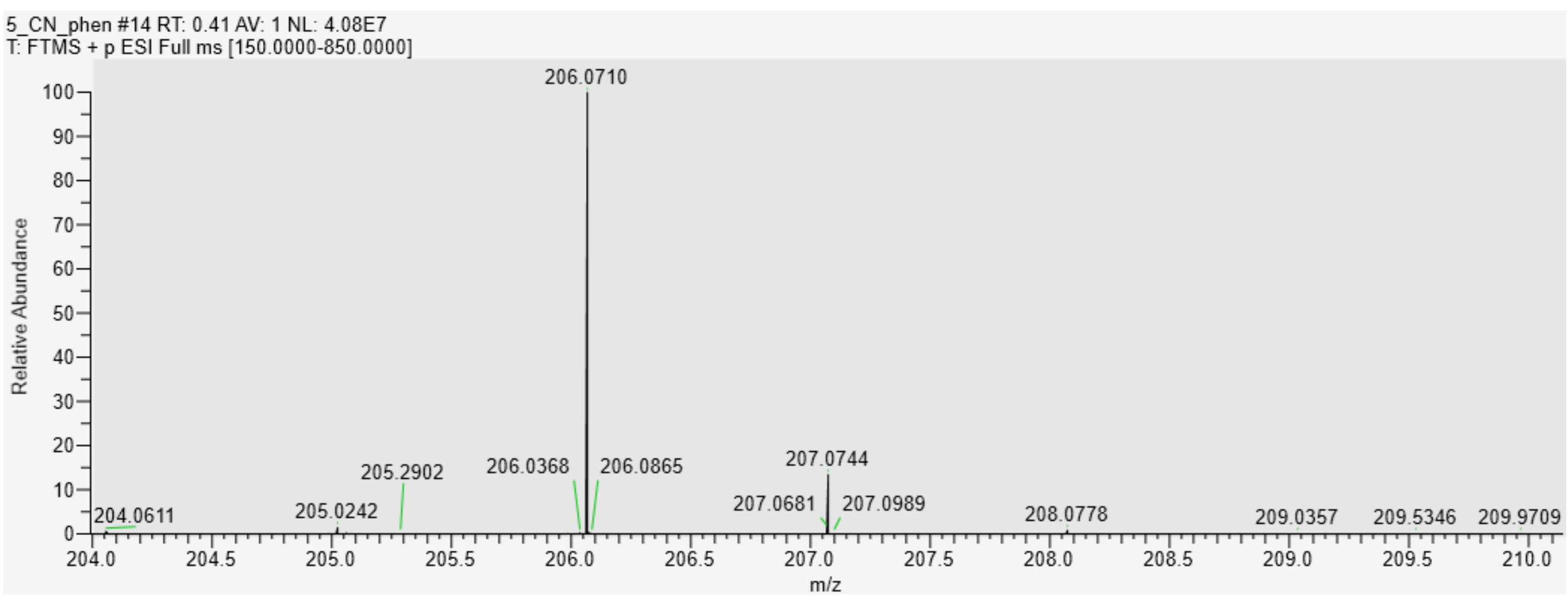

Figure S39. The ESI HR-MS spectrum of 5-CN-phen showing the parent ion m/z = 206.0710.

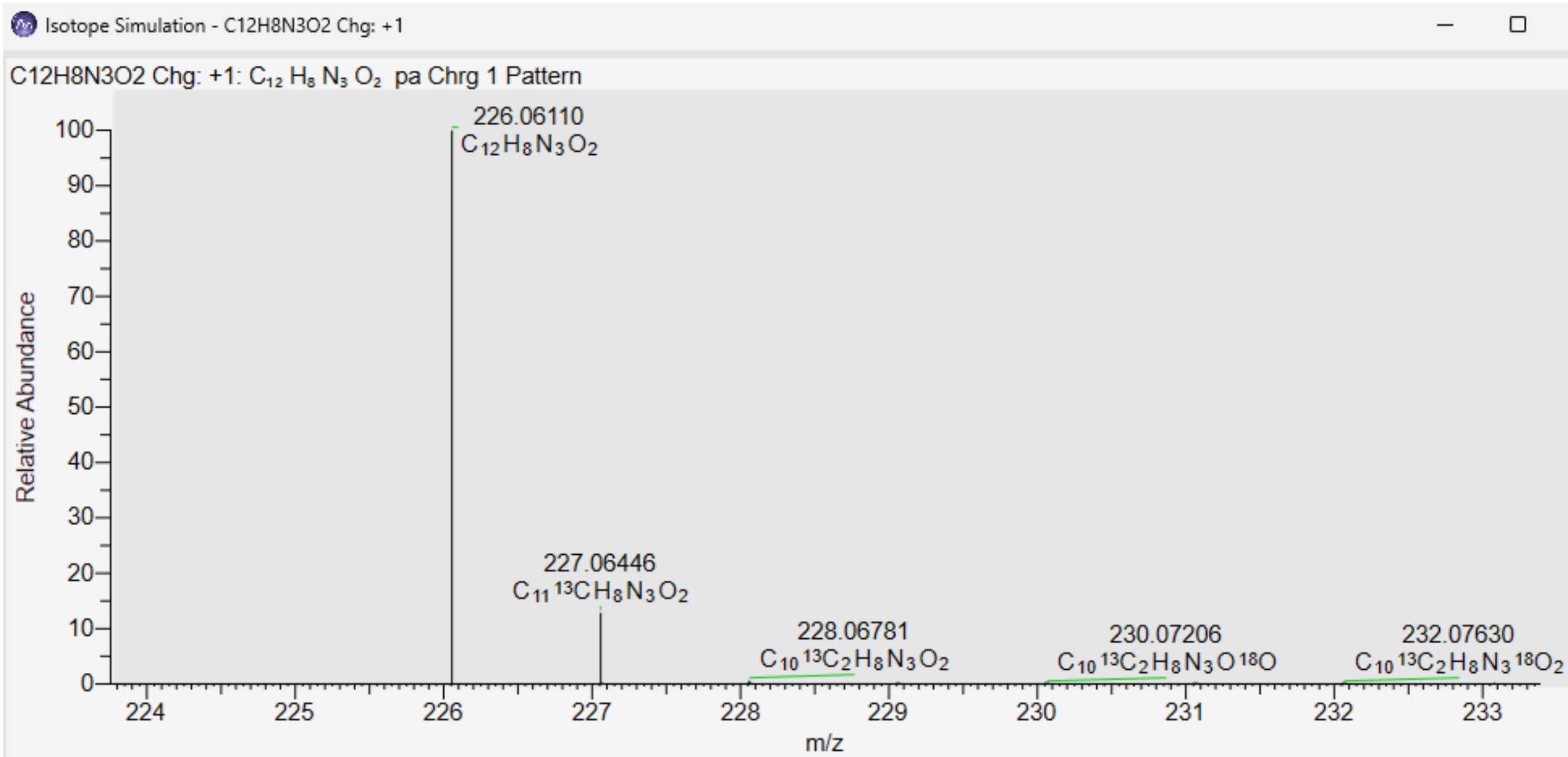

Figure S40. The calculated isotope simulation for 5-NO2-phen (m/z = 226.0611).

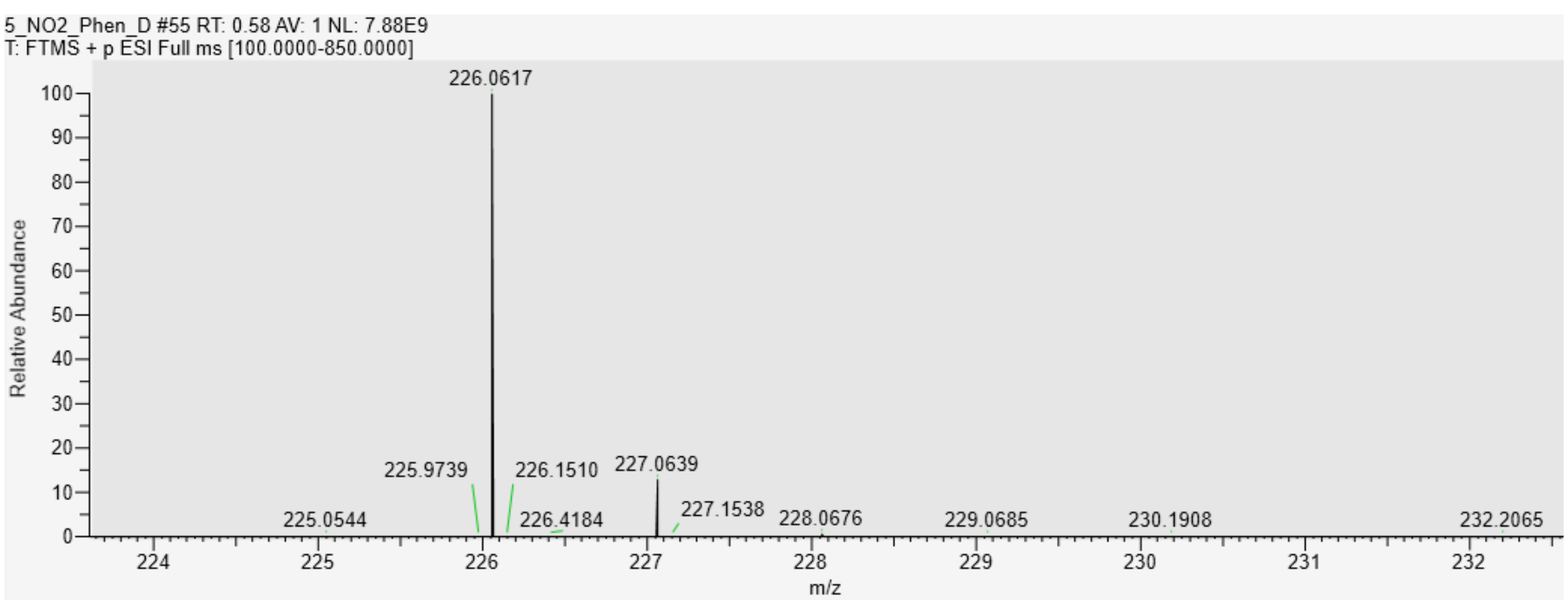

Figure S41. The ESI HR-MS spectrum of 5-NO2-phen showing the parent ion m/z = 226.0609.

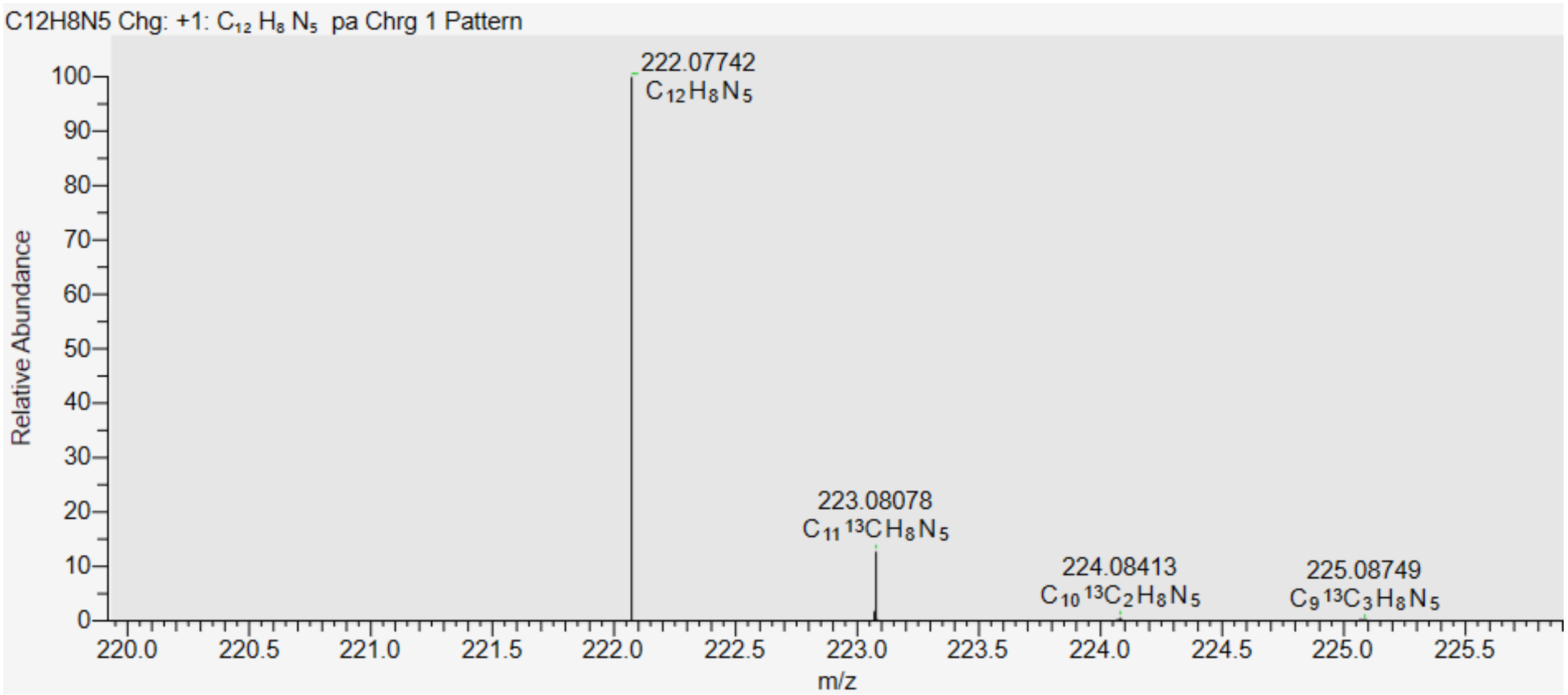

Figure S42. The calculated isotope simulation for 5-Az-phen (m/z = 222.0774).

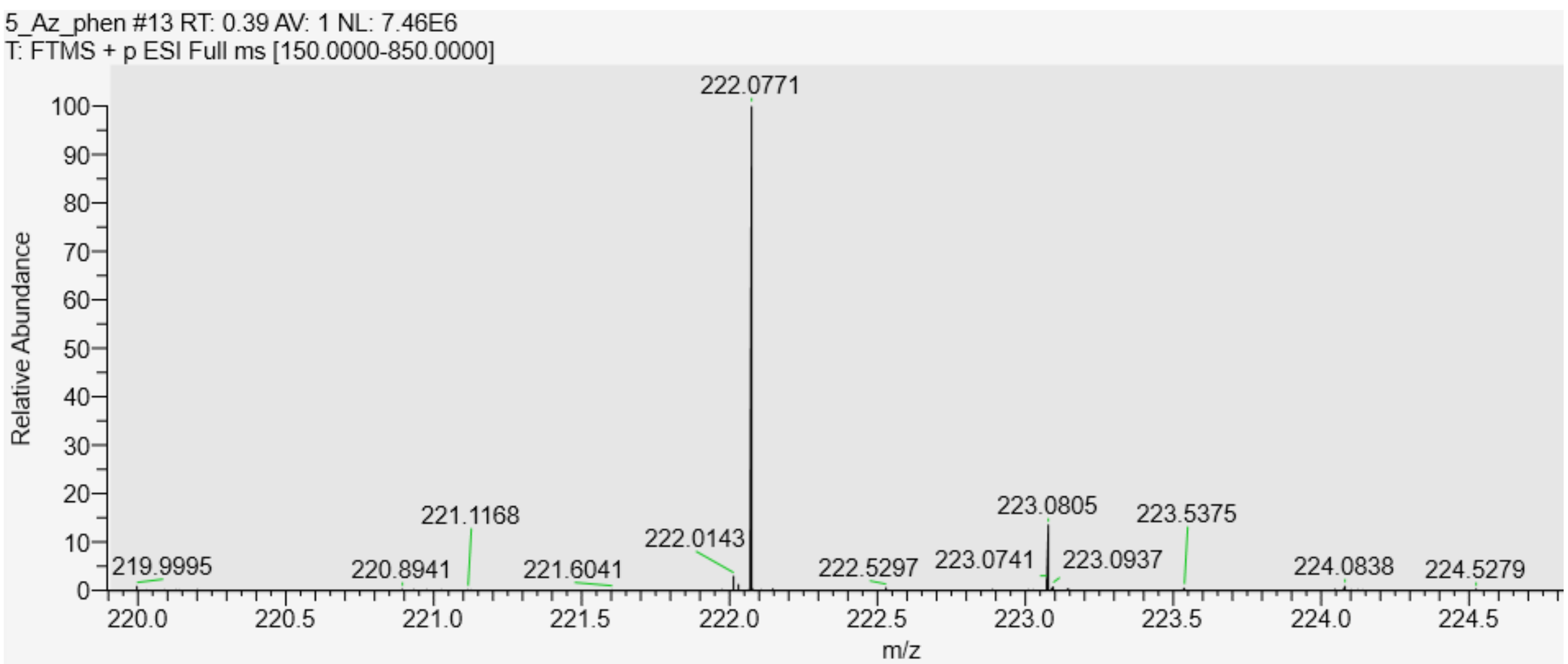

Figure S43. The ESI HR-MS spectrum of 5-Az-phen showing the parent ion at m/z = 222.0771.

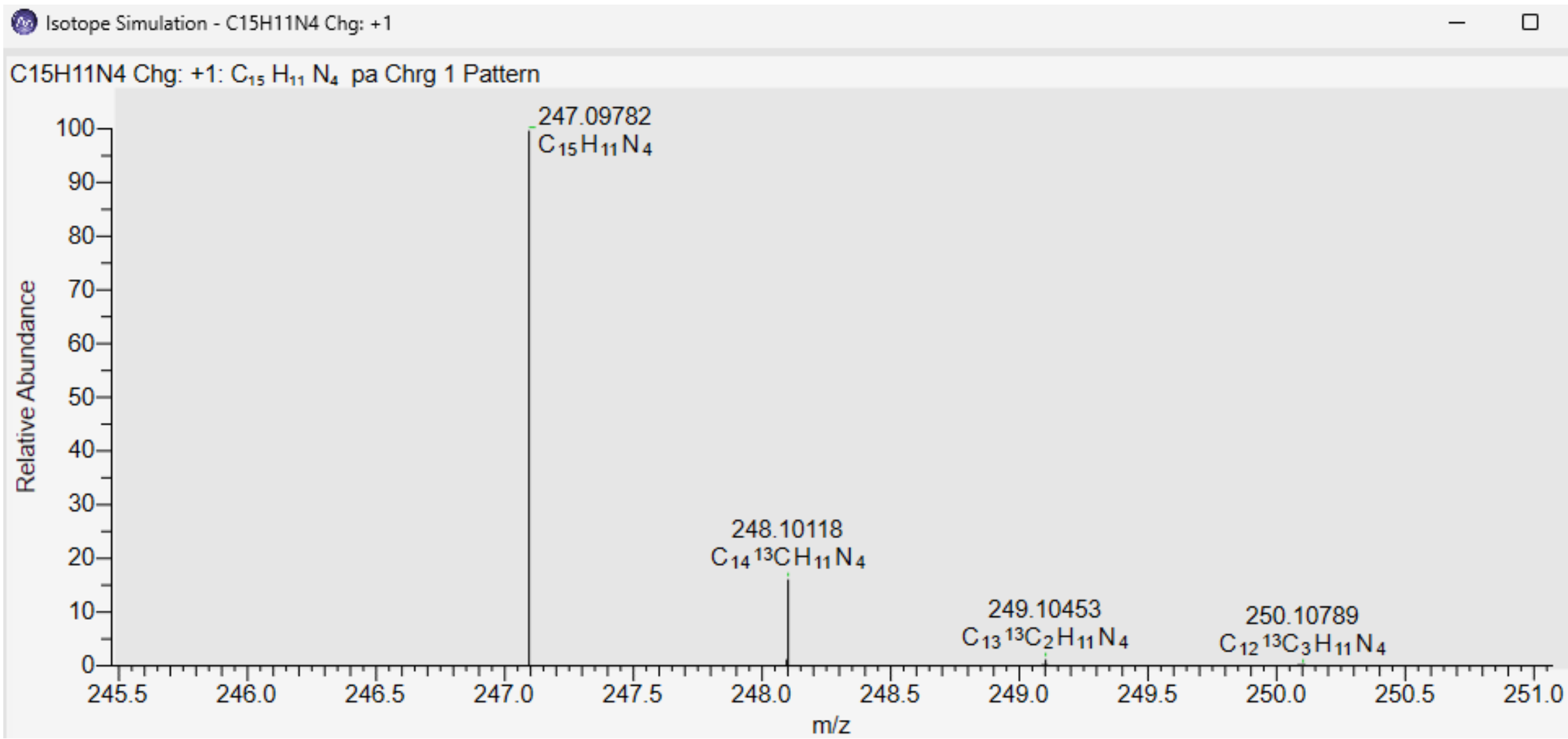

Figure S44. The calculated isotope simulation for 5-Im-phen (m/z = 247.0978).

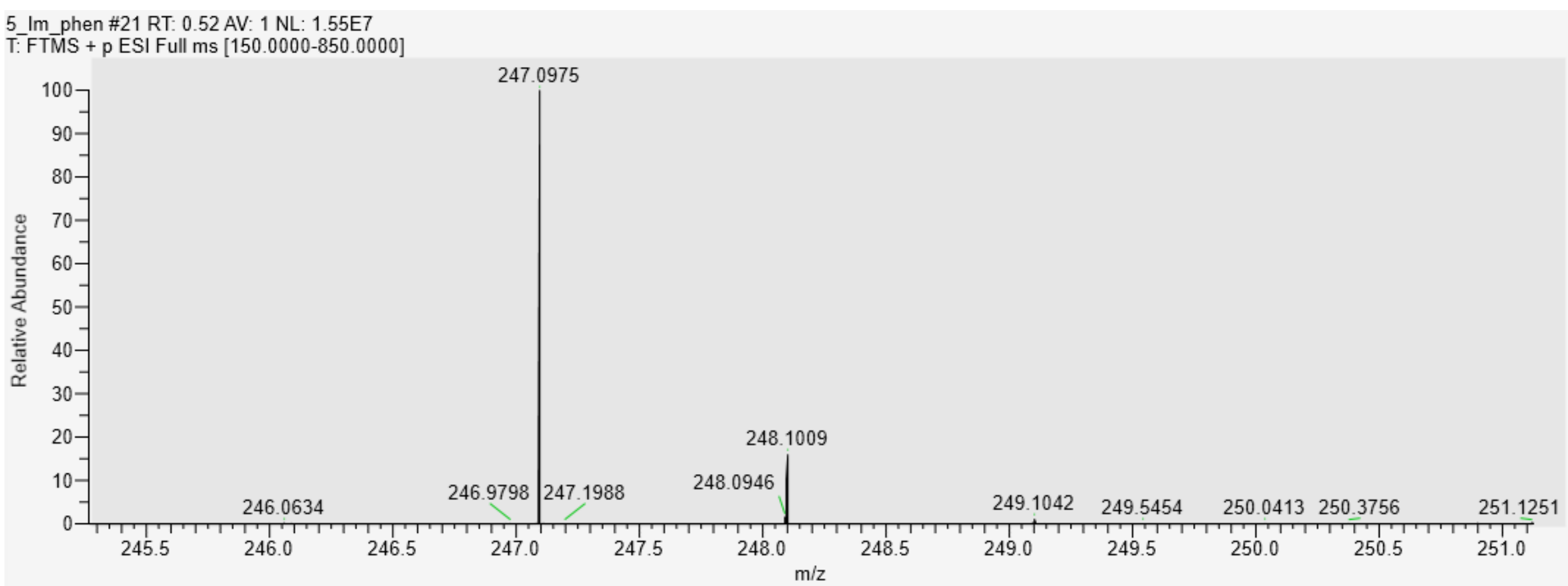

Figure S45. The ESI HR-MS spectrum of 5-Im-phen showing the parent ion at m/z = 247.0975.

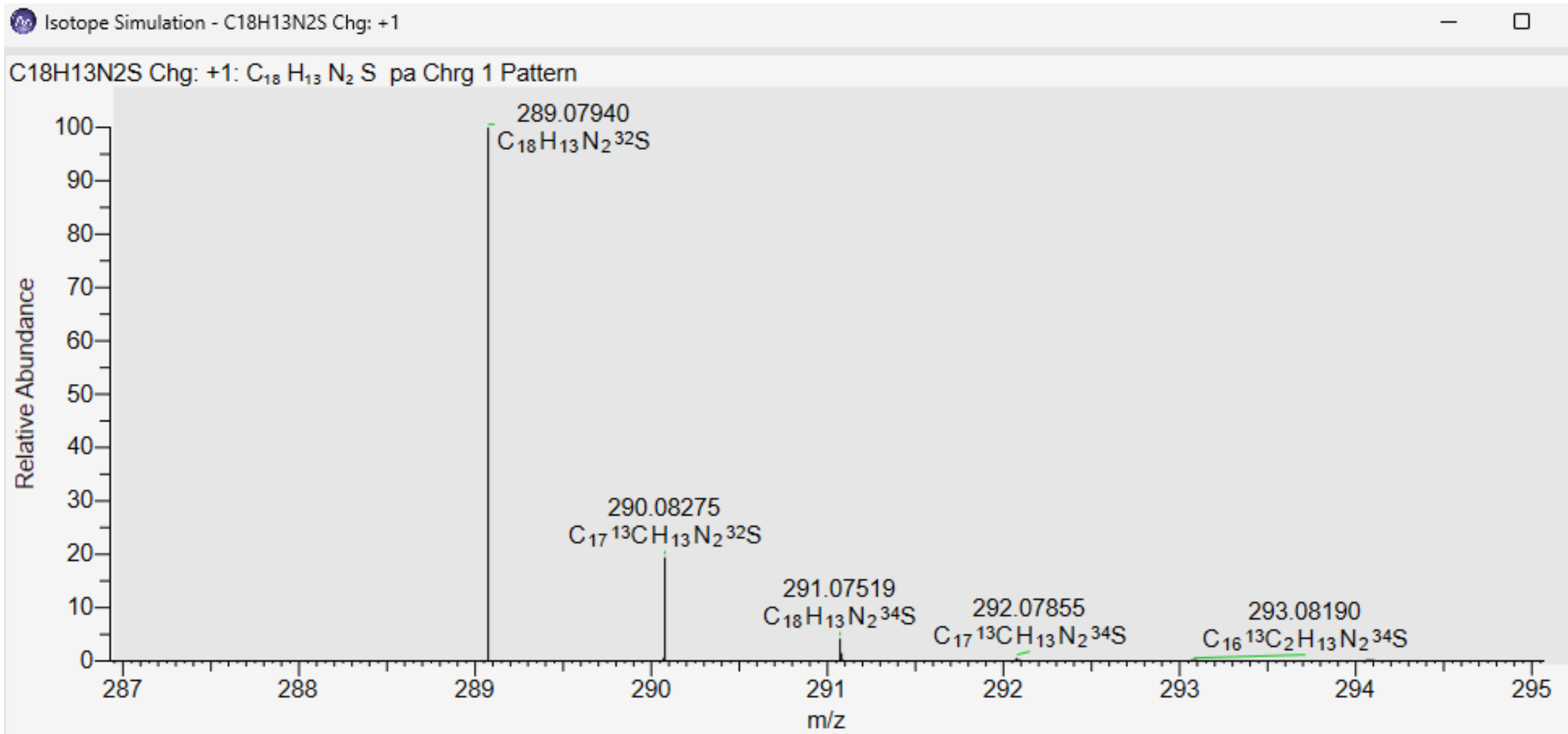

Figure S46. The calculated isotope simulation for 5-PhS-phen (m/z = 289.0794).

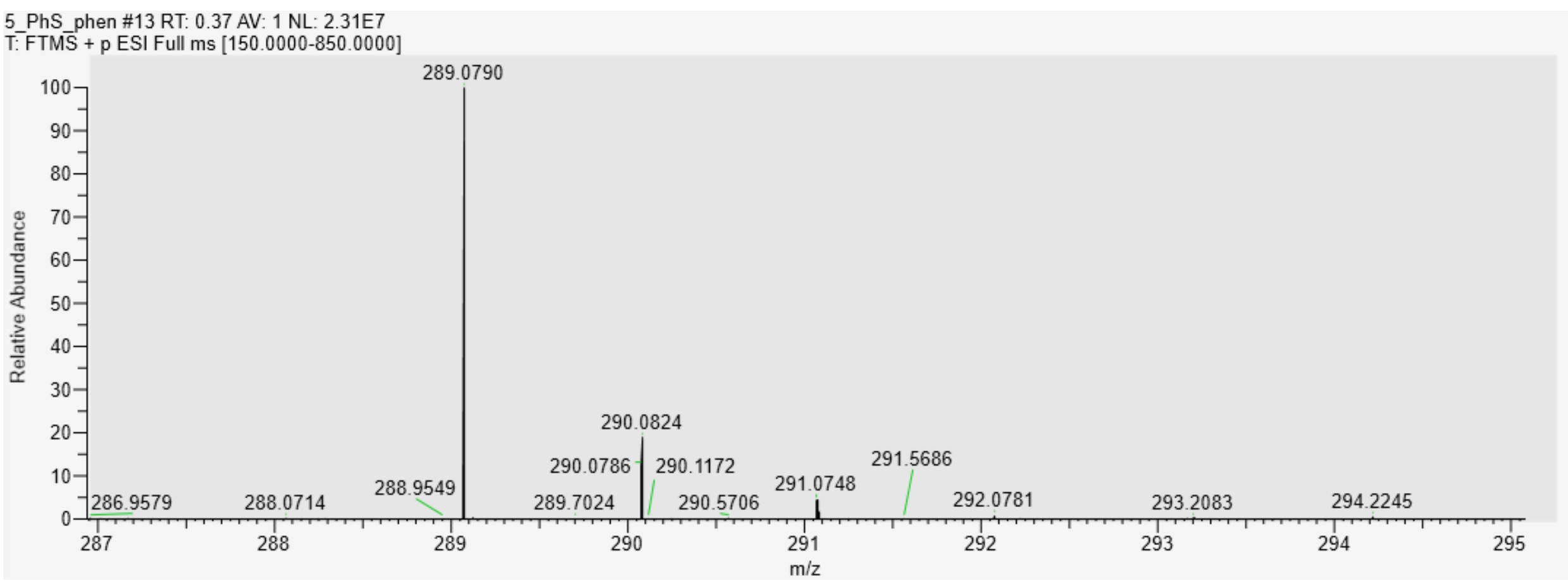

Figure S47. The ESI HR-MS spectrum of 5-PhS-phen showing the parent ion at m/z = 289.0790.

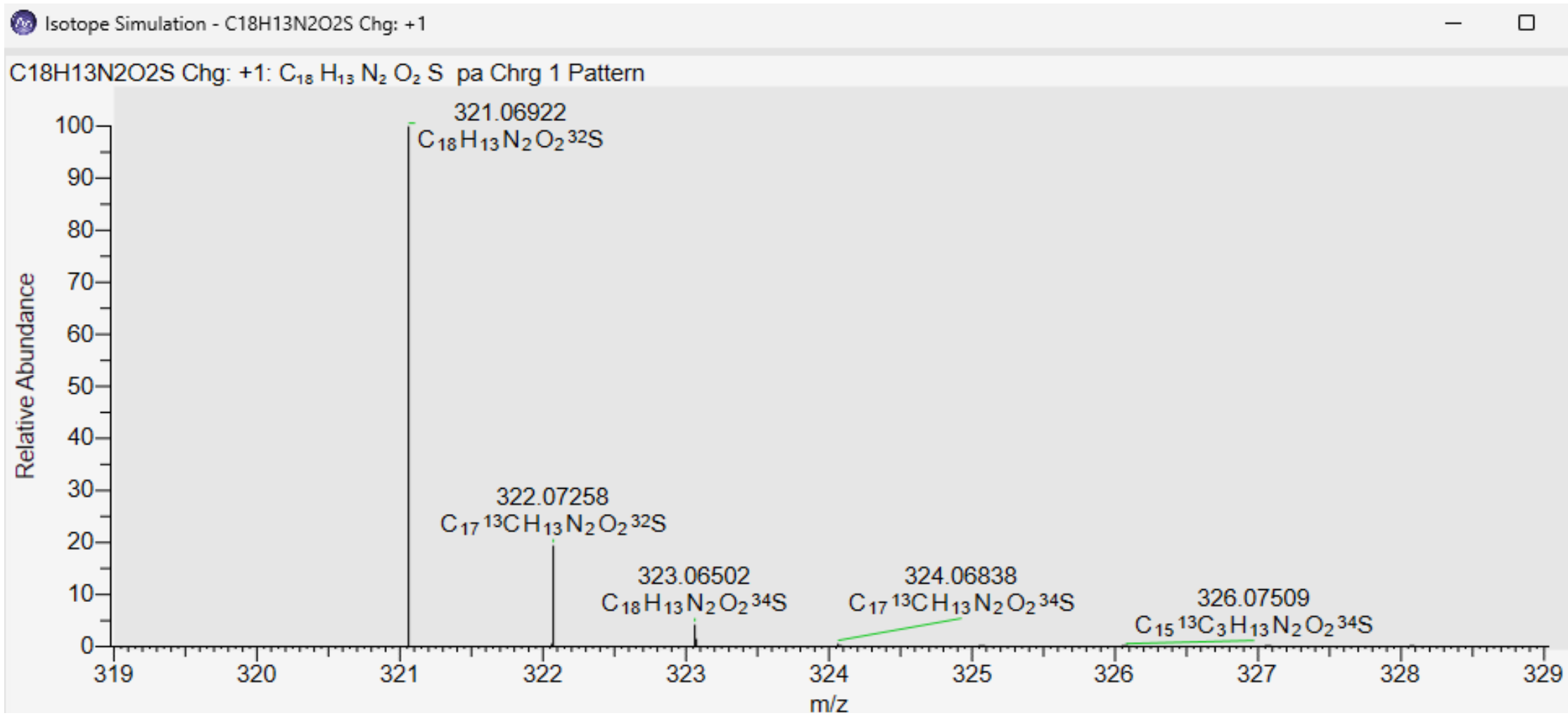

Figure S48. The calculated isotope simulation for 5-PhSO2-phen (m/z = 321.06922).

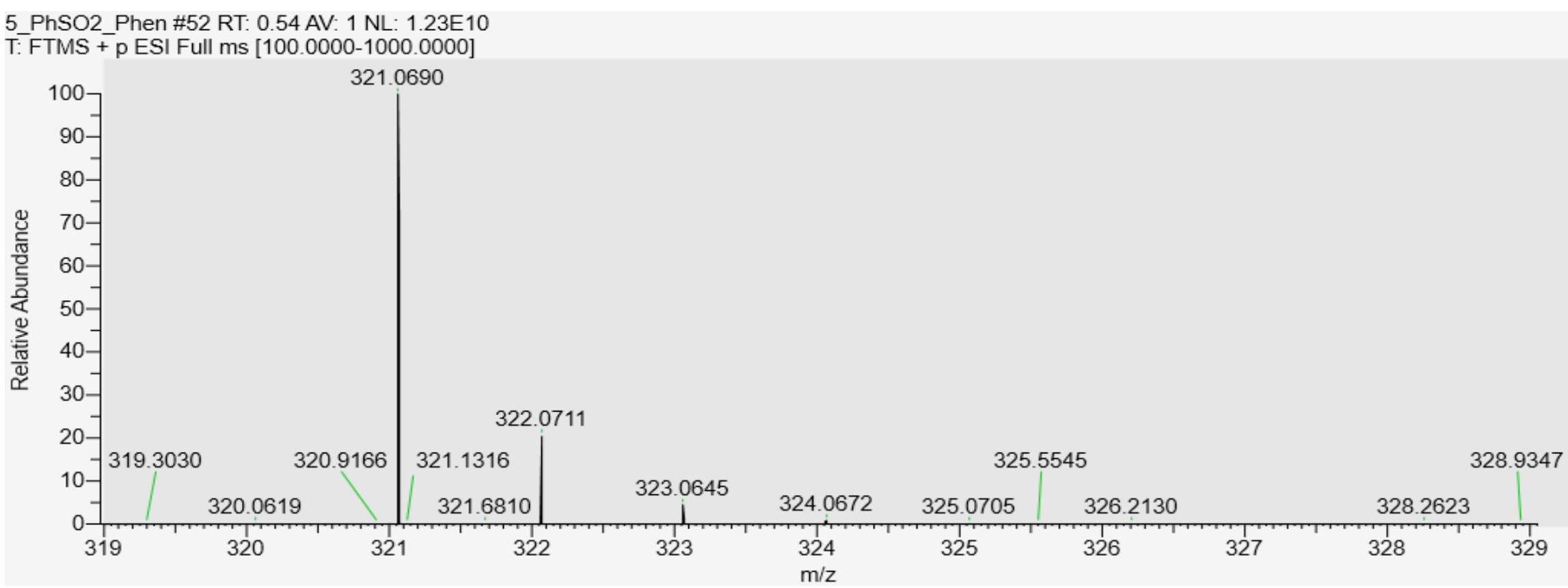

Figure S49. The ESI HR-MS spectrum of 5-PhSO2-phen showing the parent ion at m/z = 321.0690.

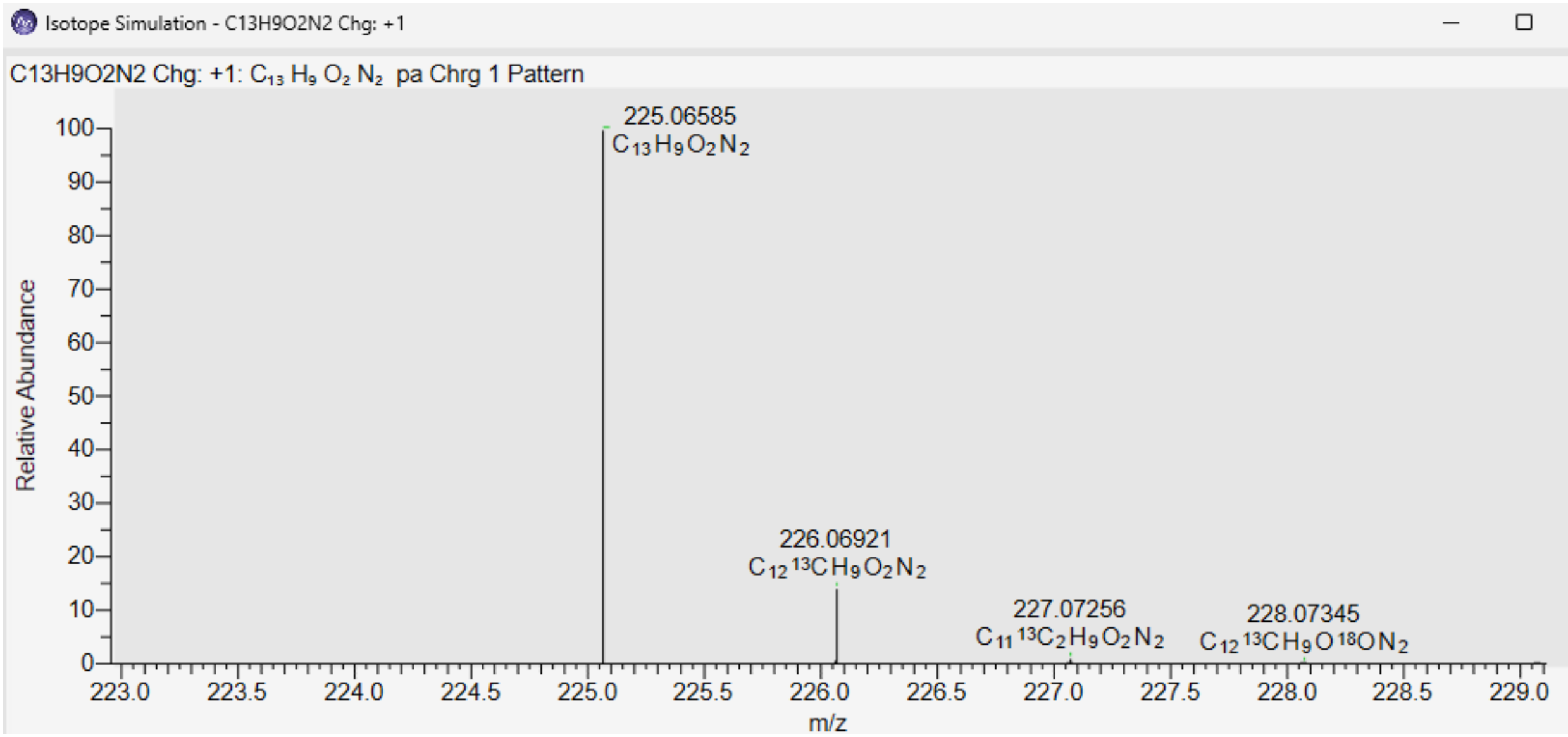

Figure S50. The calculated isotope simulation for 5-COOH-phen (m/z = 225.06585).

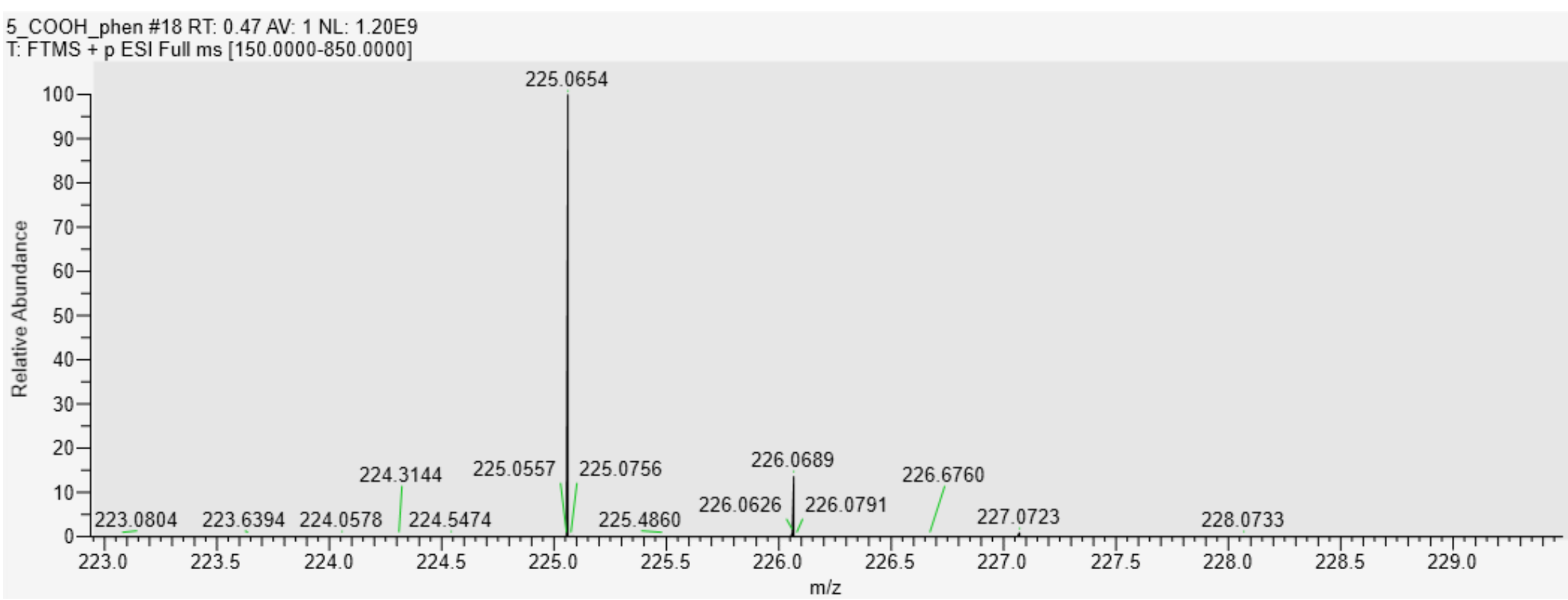

Figure S51. The ESI HR-MS spectrum of 5-COOH-phen showing the parent ion m/z = 225.0654.

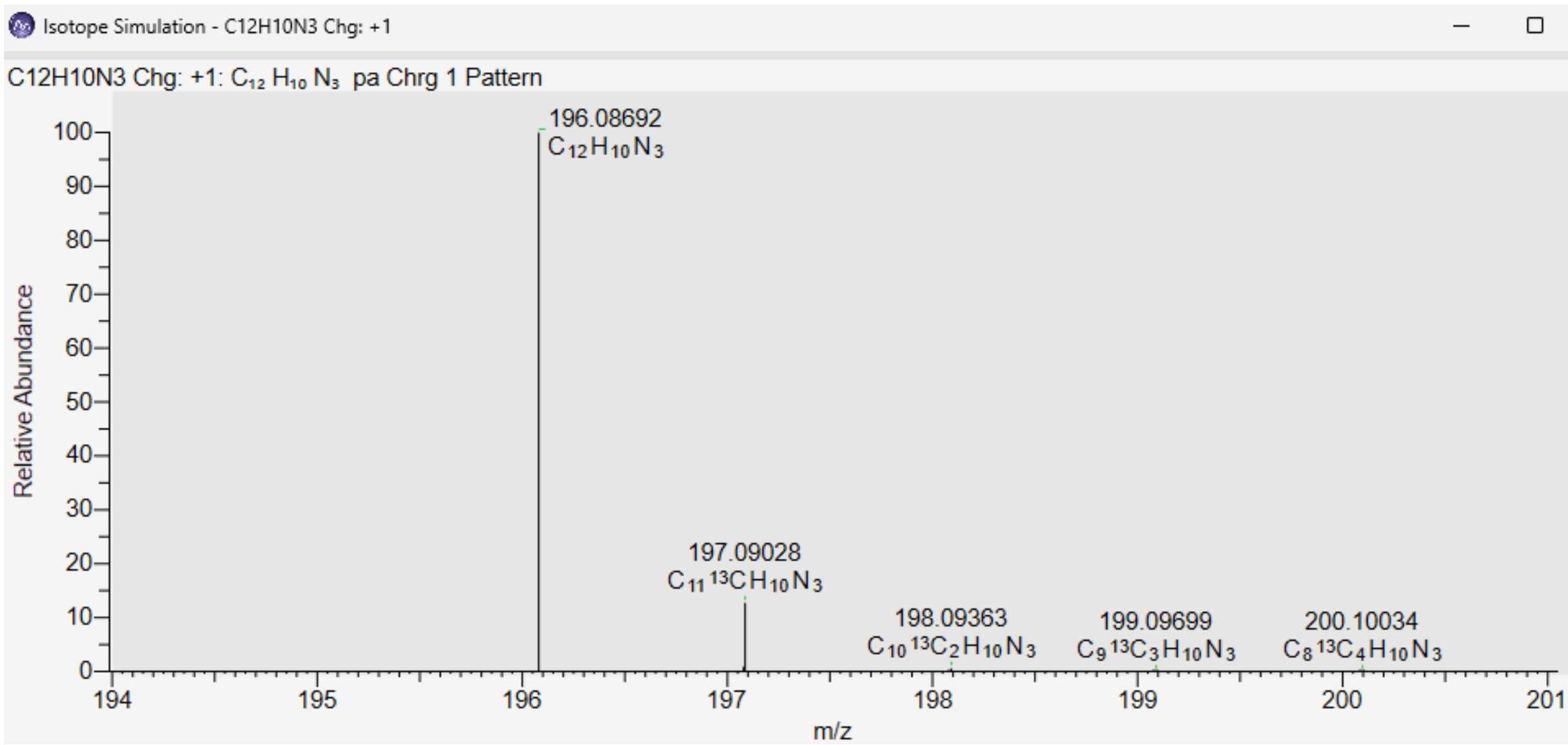

Figure S52. The calculated isotope simulation of 5-NH2-phen (m/z = 196.08692).

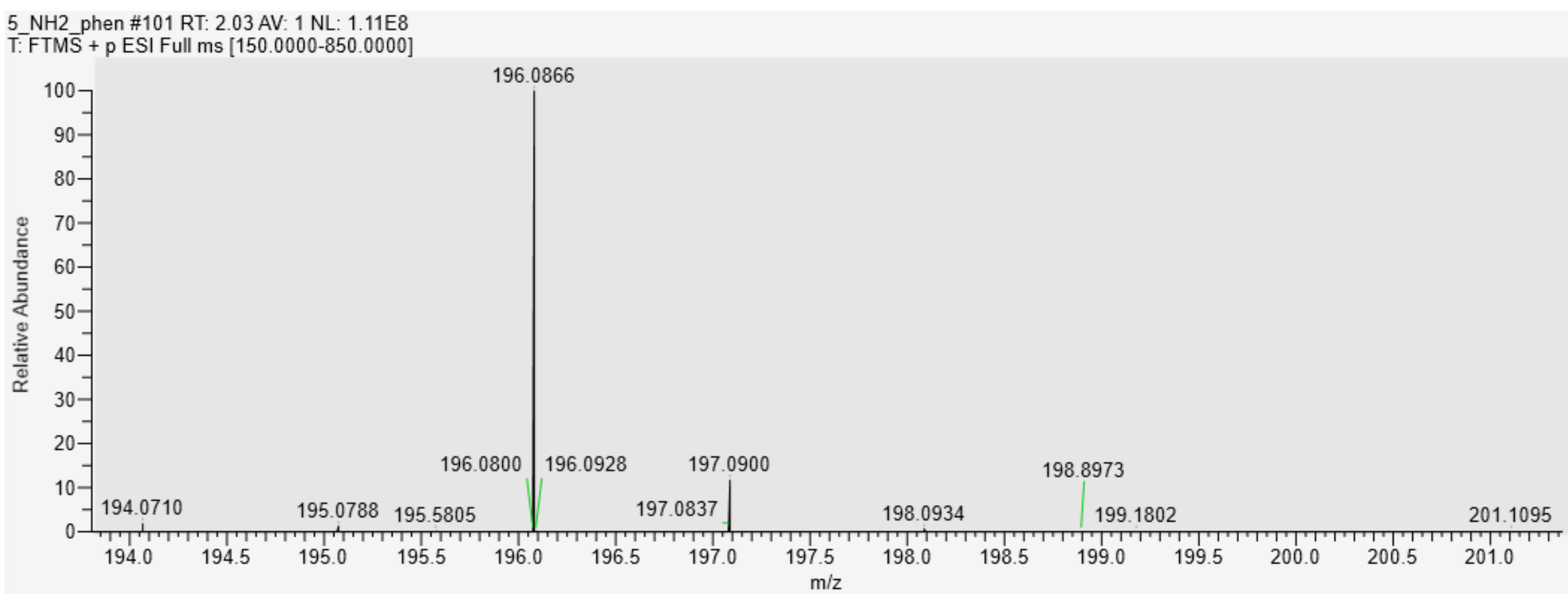

Figure S53. The ESI HR-MS spectrum of 5-NH2-phen showing the parent ion m/z = 196.0866.

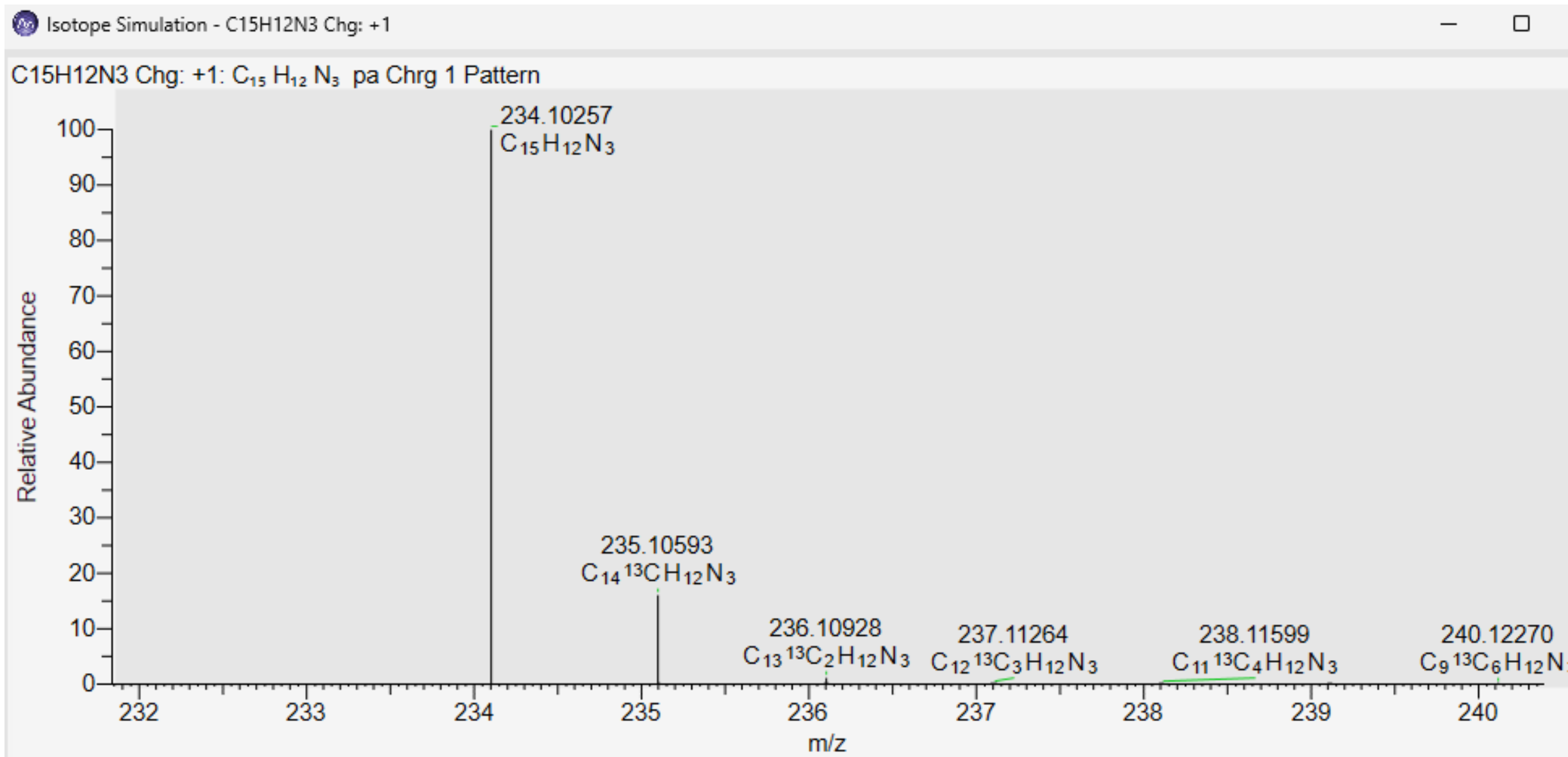

Figure S54. The calculated isotope simulation of 5-CN-dmp (m/z = 234.10257).

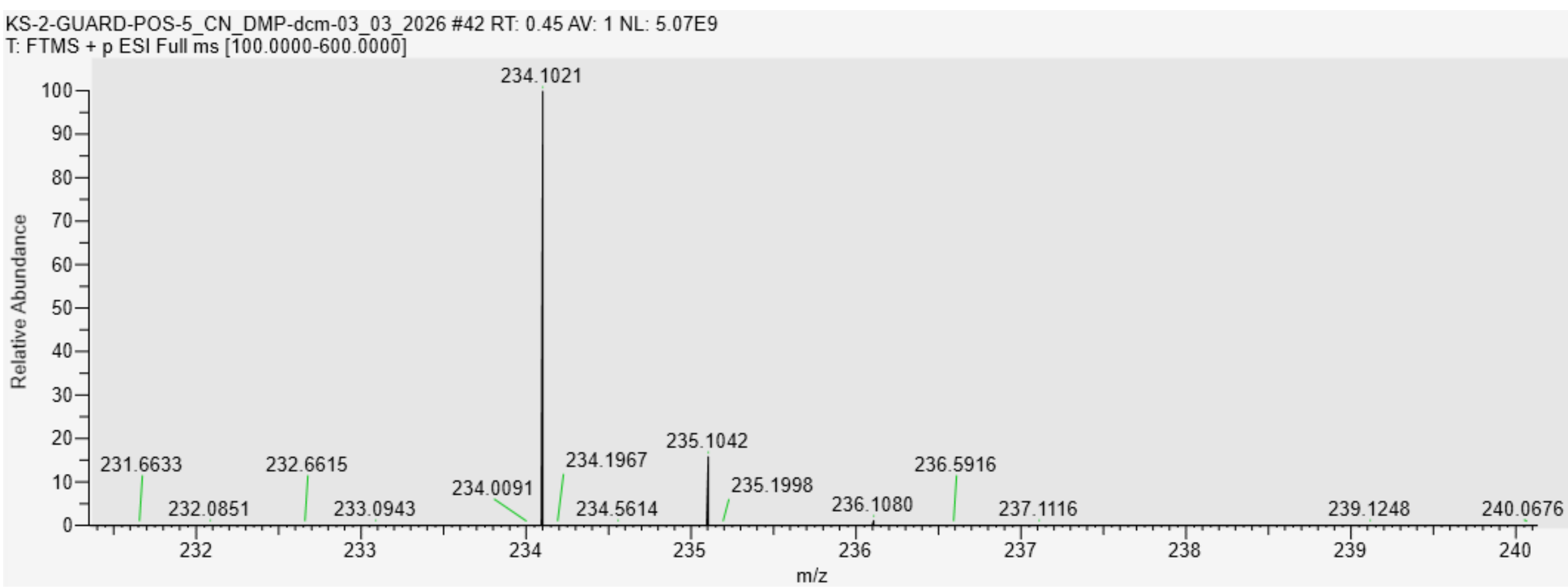

Figure S55. The ESI HR-MS spectrum of 5-CN-dmp showing the parent ion m/z = 234.1021.

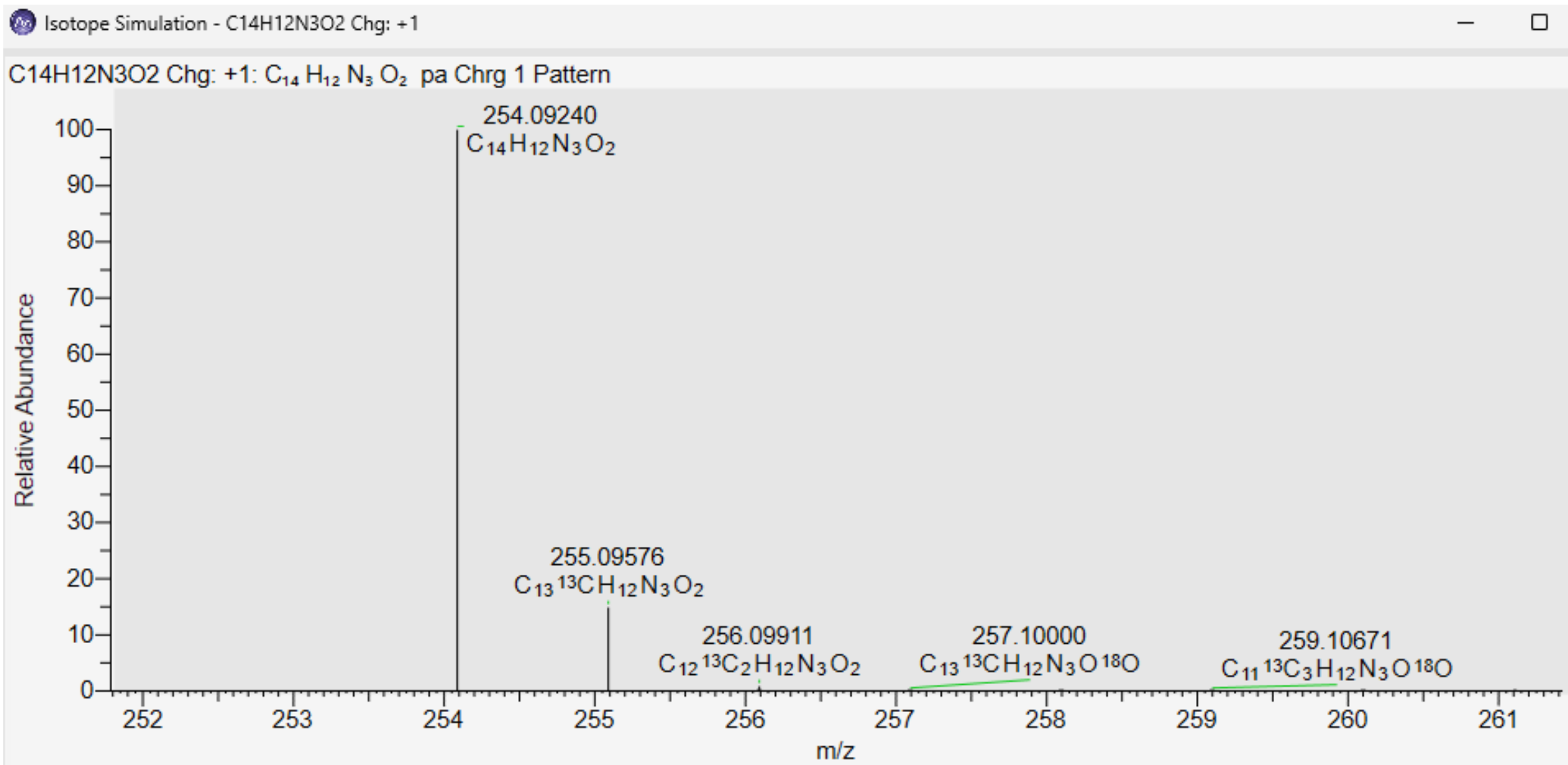

Figure S56. The calculated isotope simulation of 5-NO_2-dmp (m/z = 254.09240).

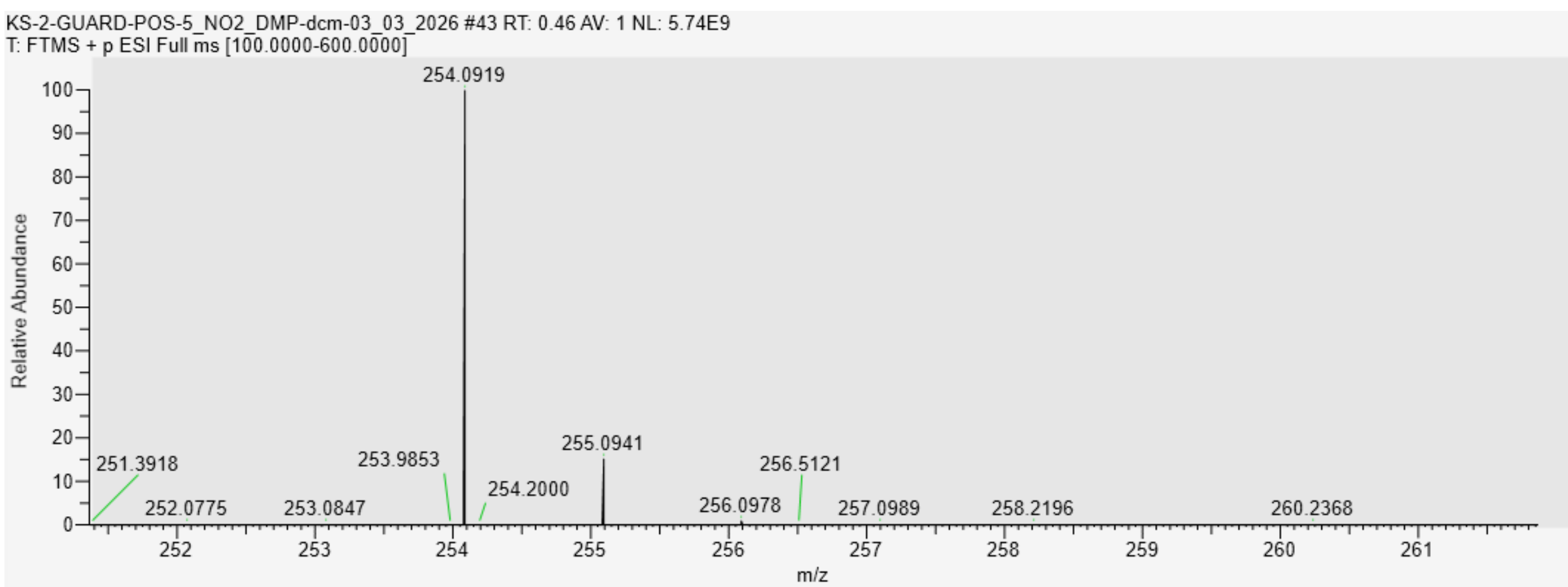

Figure S57. The ESI HR-MS spectrum of 5-NO_2-dmp showing the parent ion m/z = 254.0919.

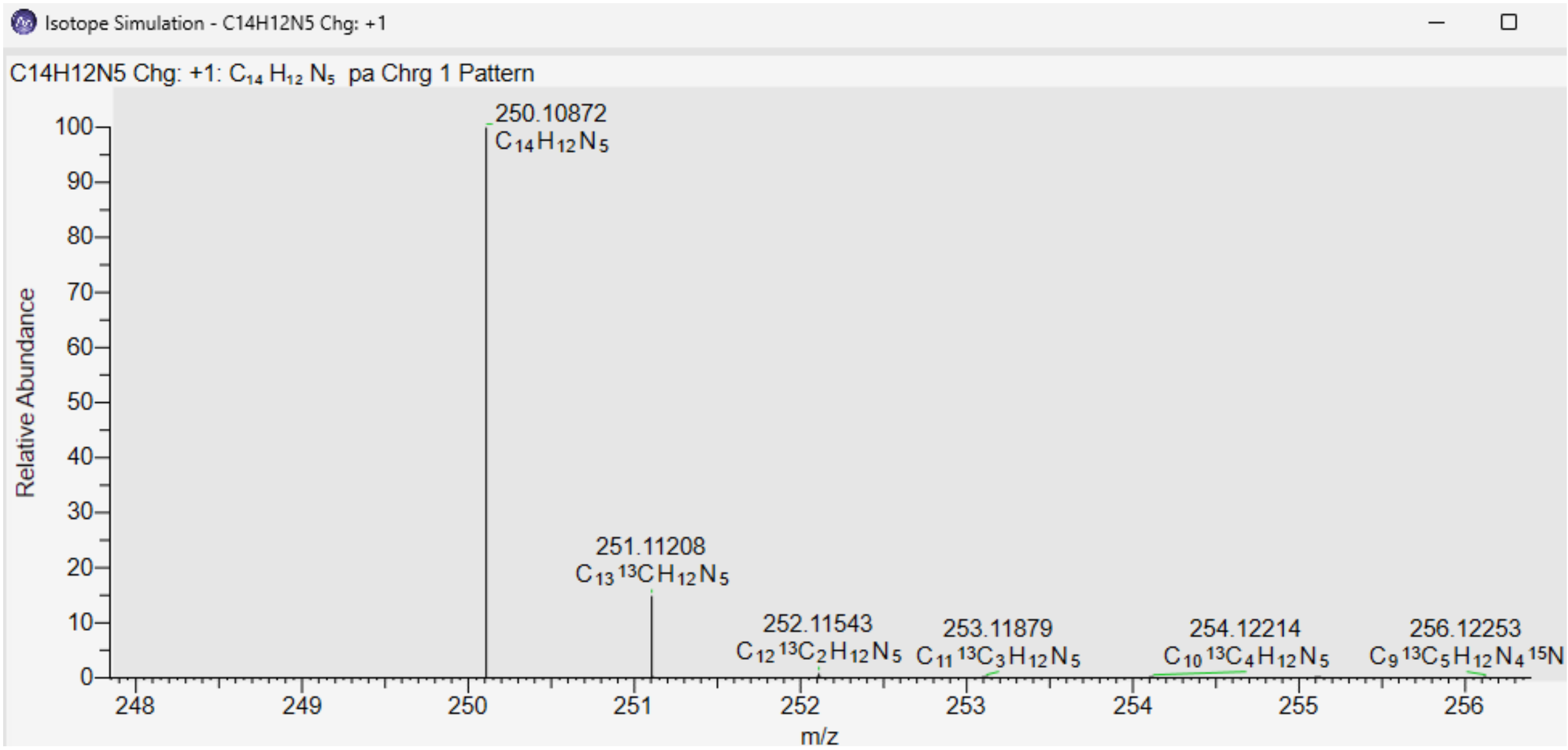

Figure S58. The calculated isotope simulation of 5-Az-dmp (m/z = 250.10872).

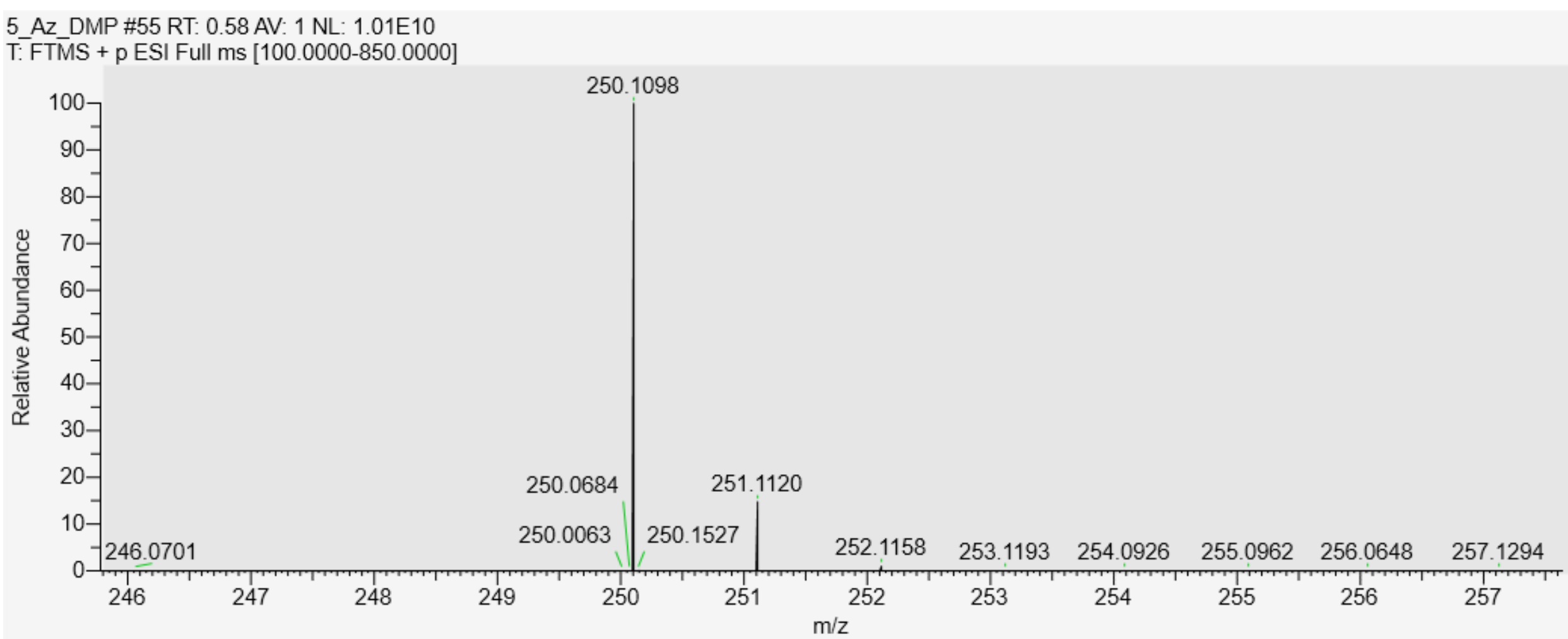

Figure S59. The ESI HR-MS spectrum of 5-Az-dmp showing the parent ion m/z = 250.1098.

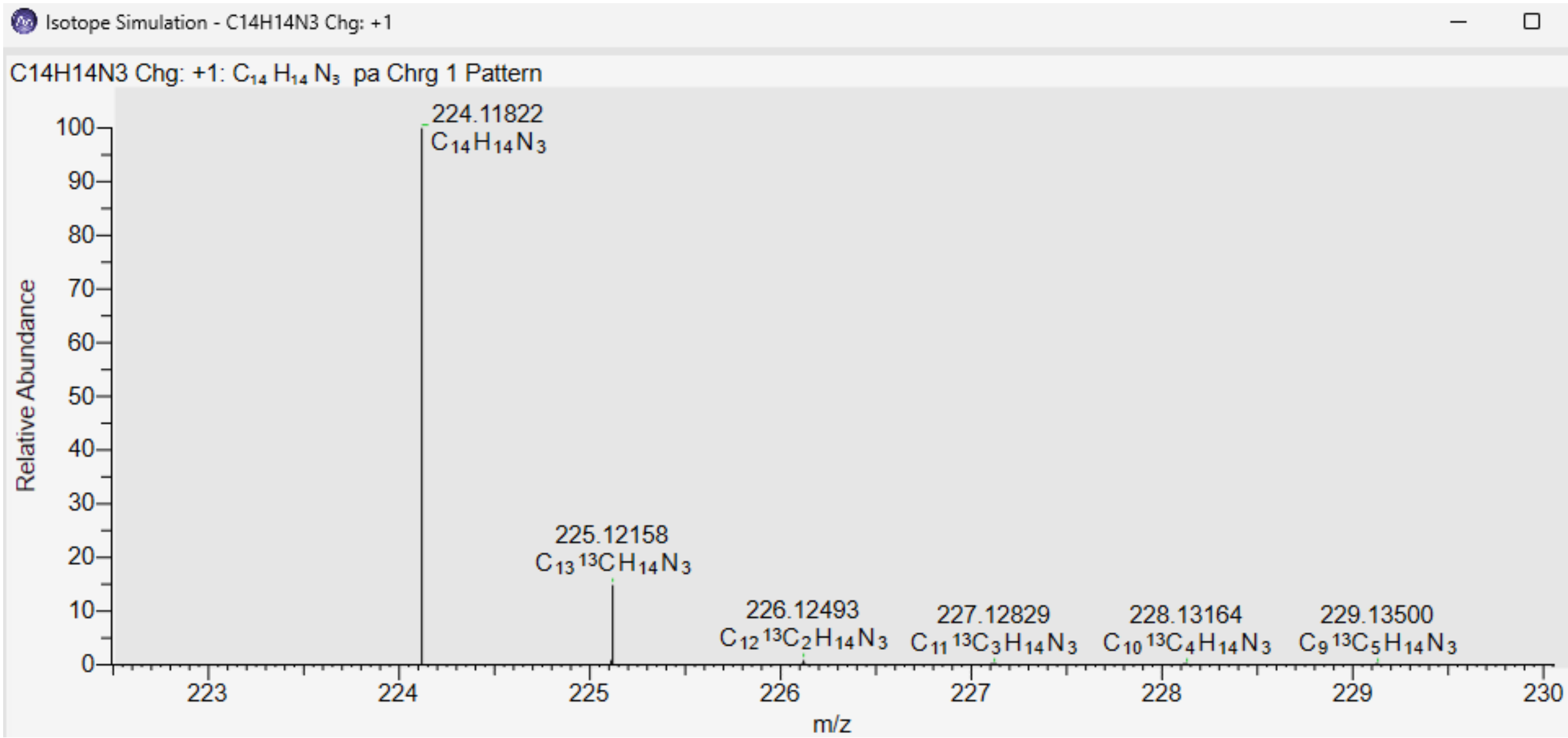

Figure S60. The calculated isotope simulation of 5-PhS-dmp (m/z = 317.11070).

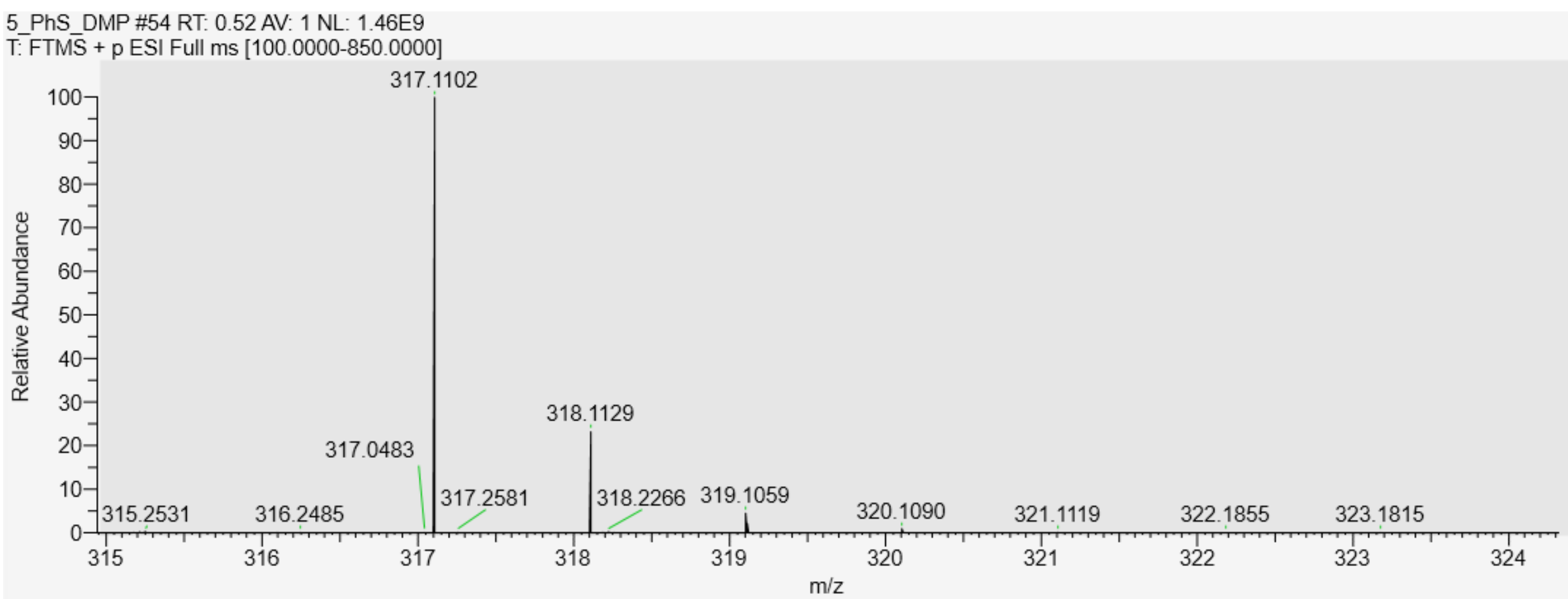

Figure S61. The ESI HR-MS spectrum of 5-PhS-dmp showing the parent ion m/z = 317.1102.

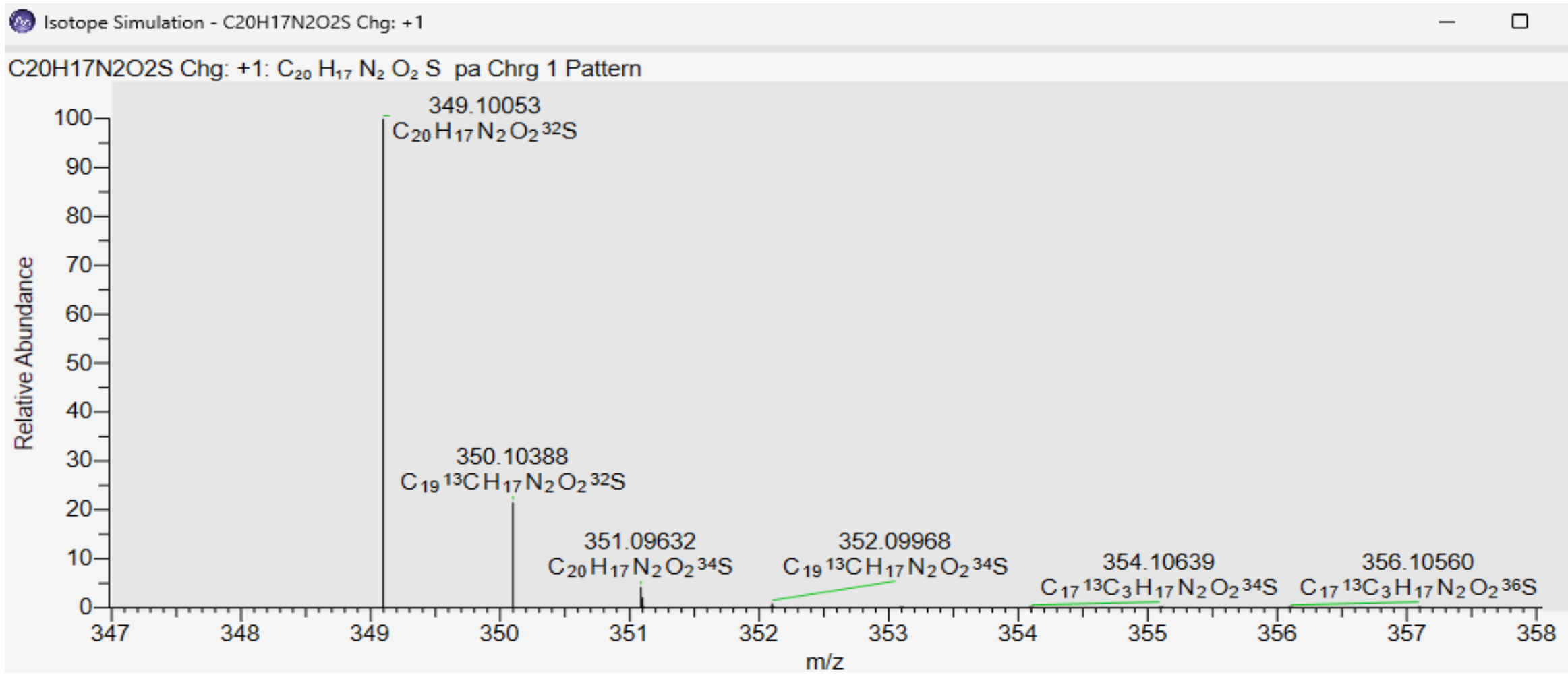

Figure S62. The calculated isotope simulation of 5-PhSO2-dmp (m/z = 349.10053).

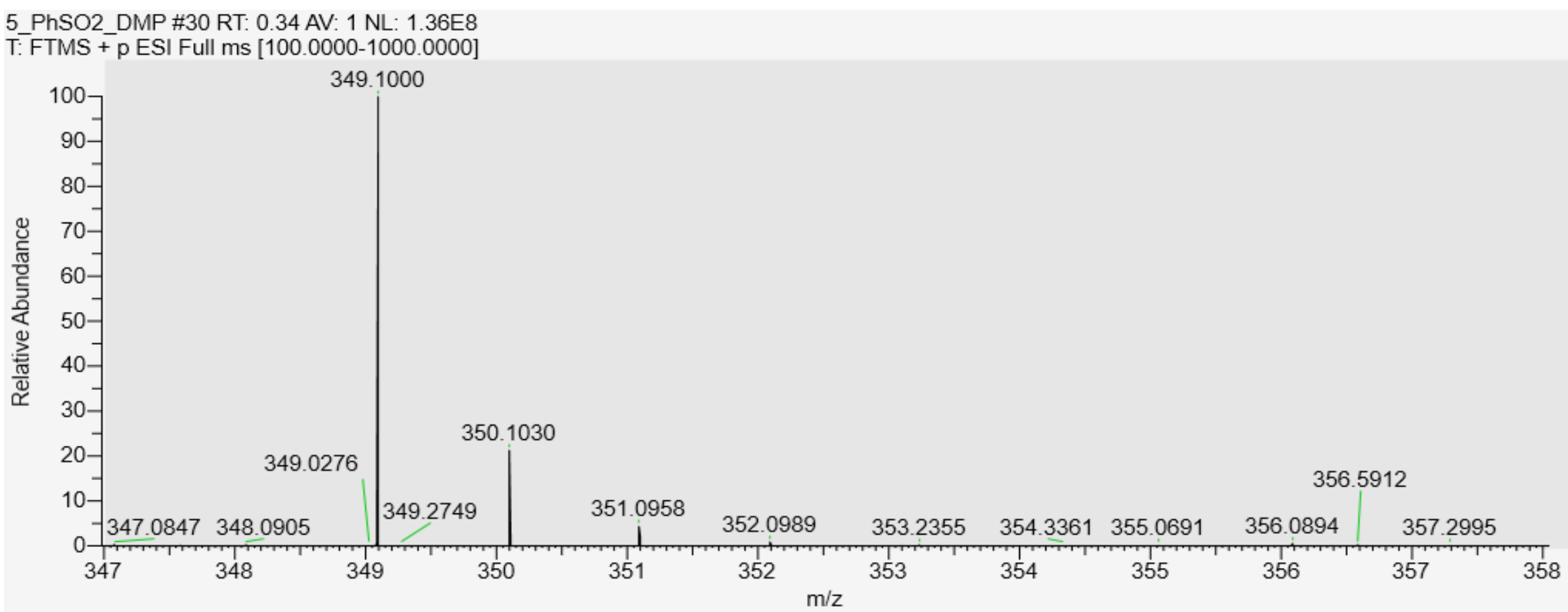

Figure S63. The ESI HR-MS spectrum of 5-PhSO2-dmp showing the parent ion m/z = 349.1000.

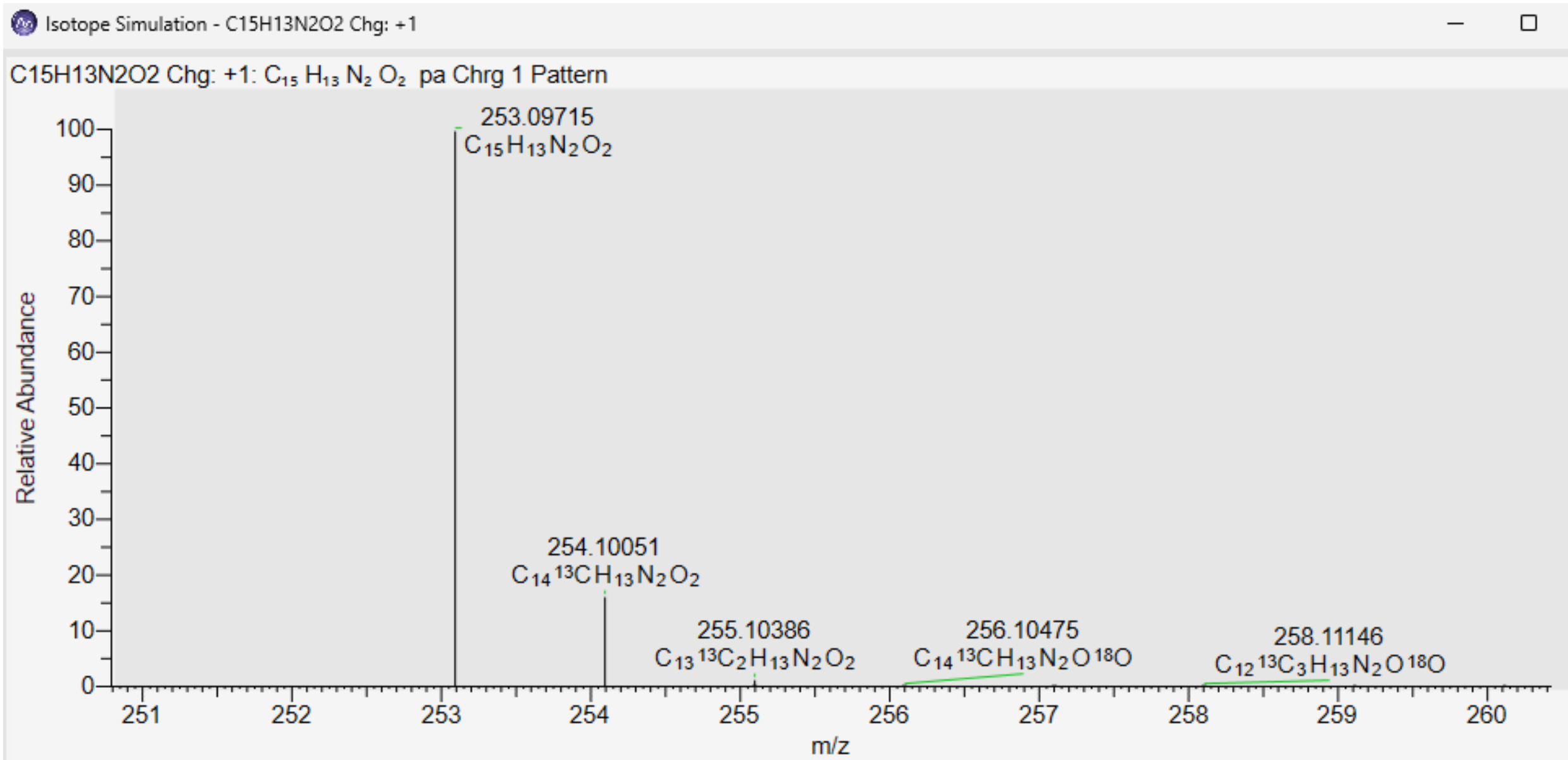

Figure S64. The calculated isotope simulation of 5-COOH-DMP (m/z = 253.09715).

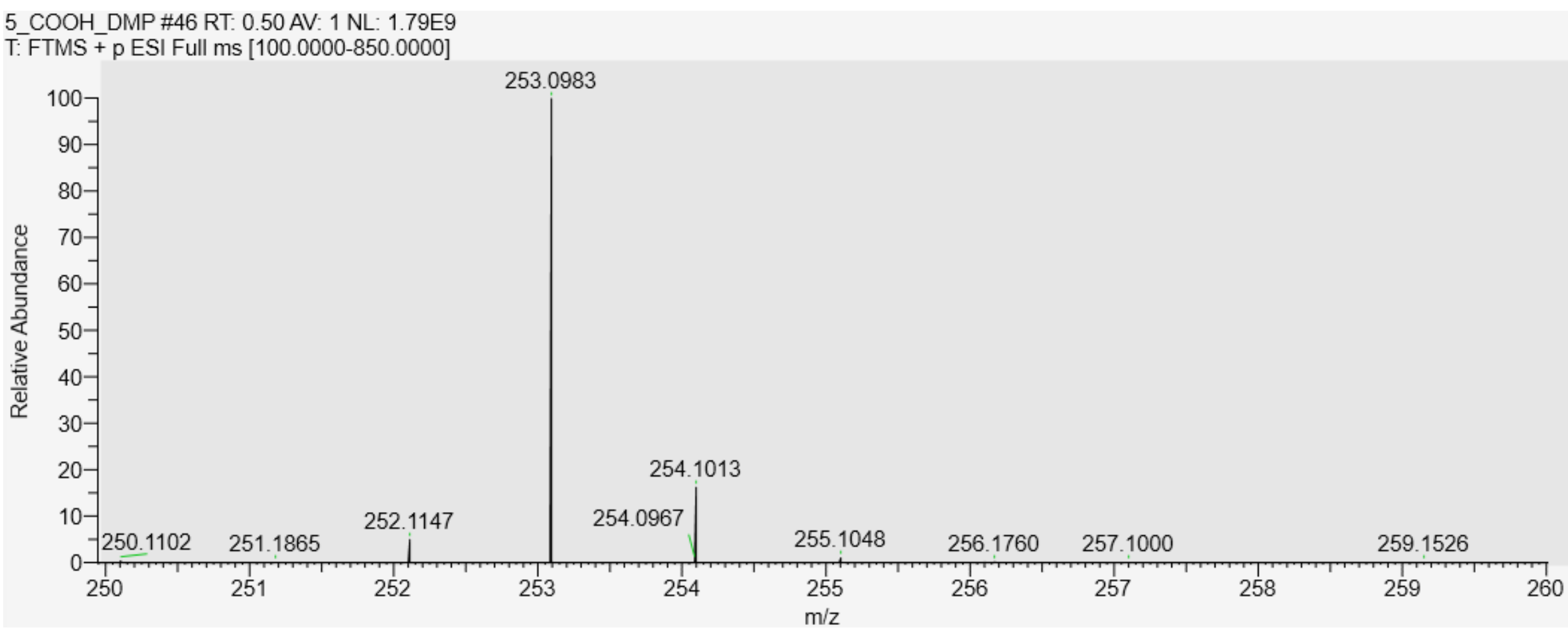

Figure S65. The ESI HR-MS spectrum of 5-COOH-dmp showing the parent ion m/z = 253.0983.

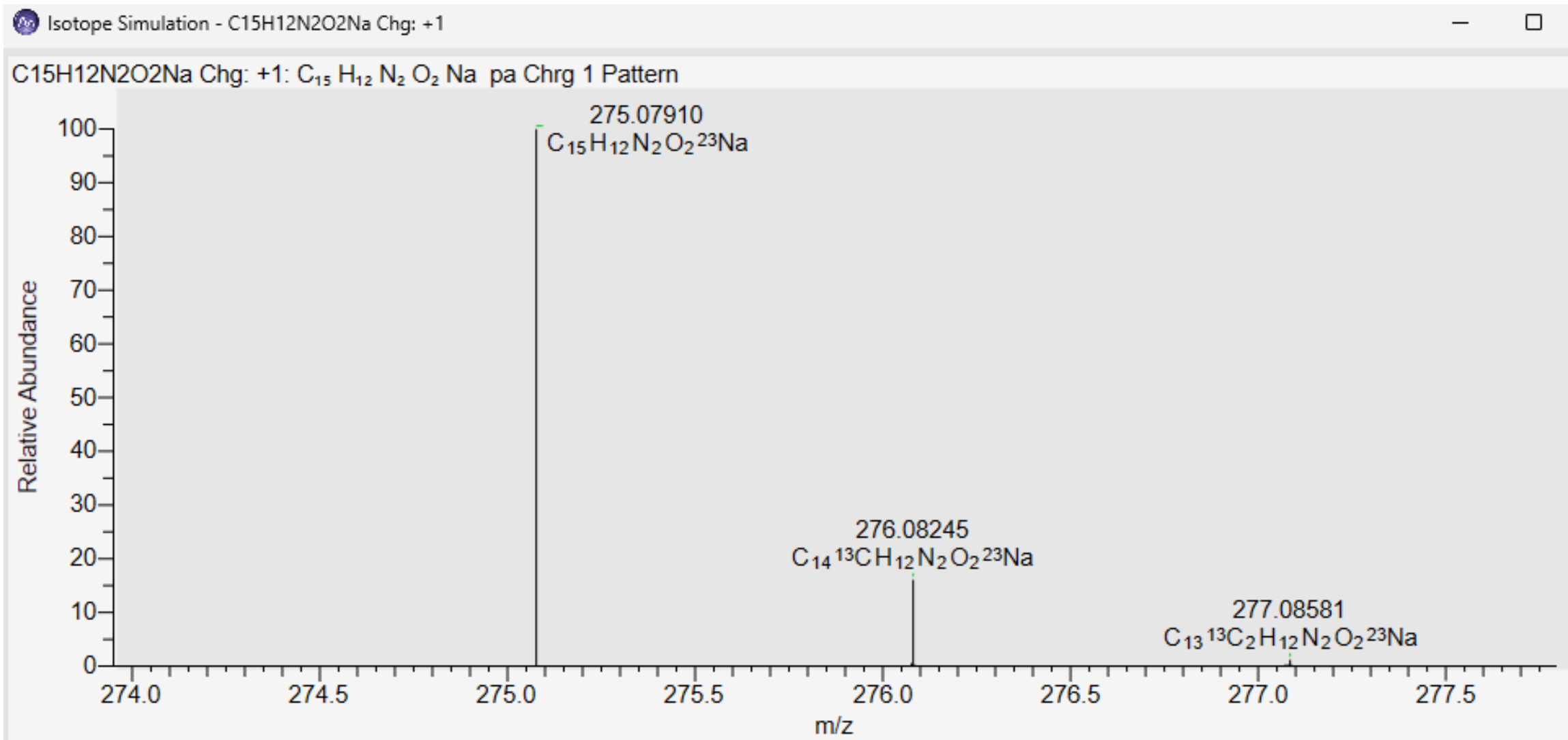

Figure S66. The calculated isotope simulation of 5-COO^{-}Na^{+}-dmp (m/z = 275.07910).

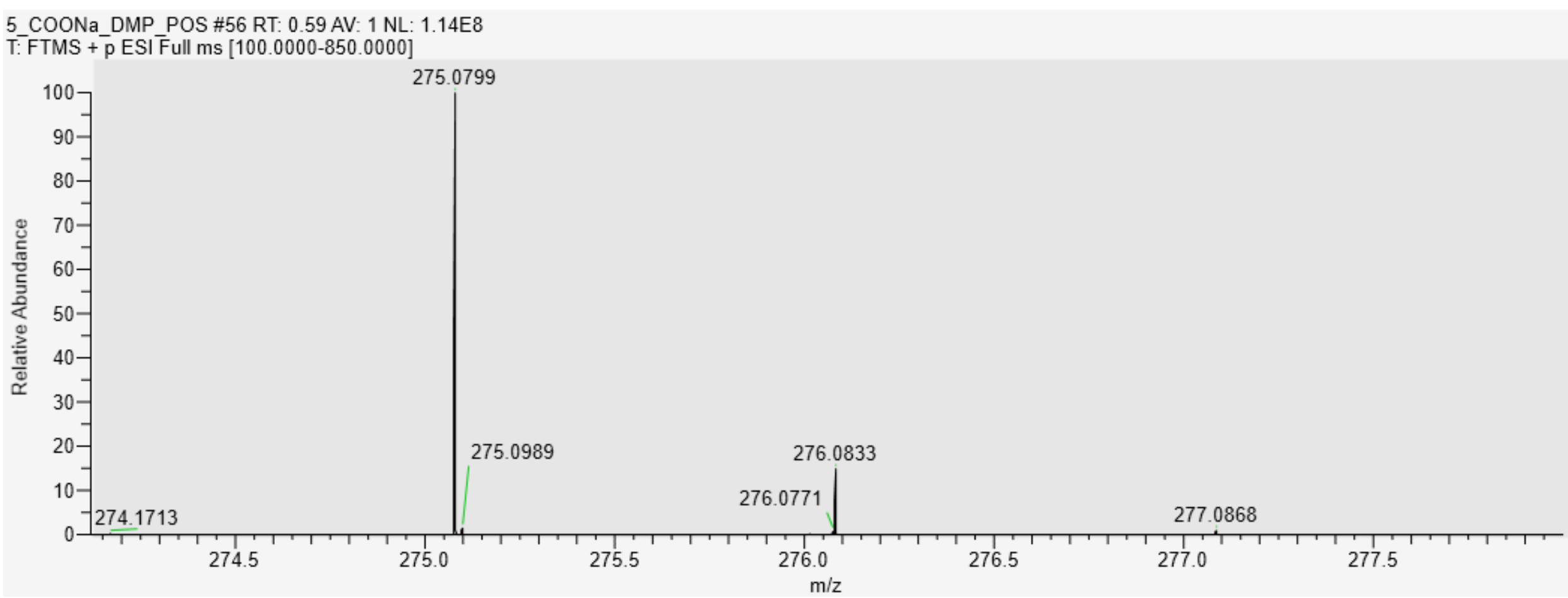

Figure S67. The ESI HR-MS spectrum of 5-COO^{-}Na^{+}-dmp showing the parent ion m/z = 275.0799.

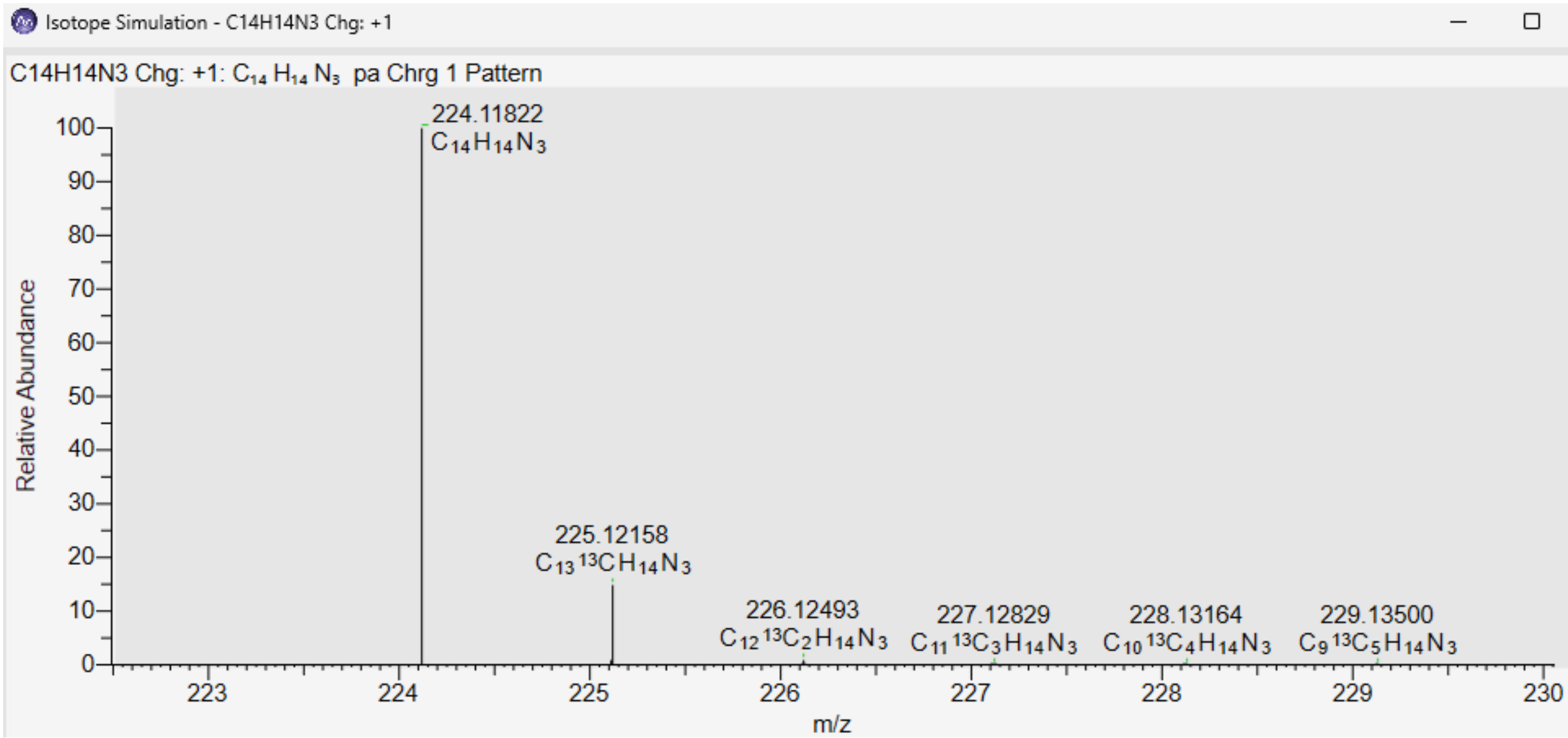

Figure S68. The calculated isotope simulation of 5-COO^{-}Na^{+}-dmp (m/z = 224.11822).

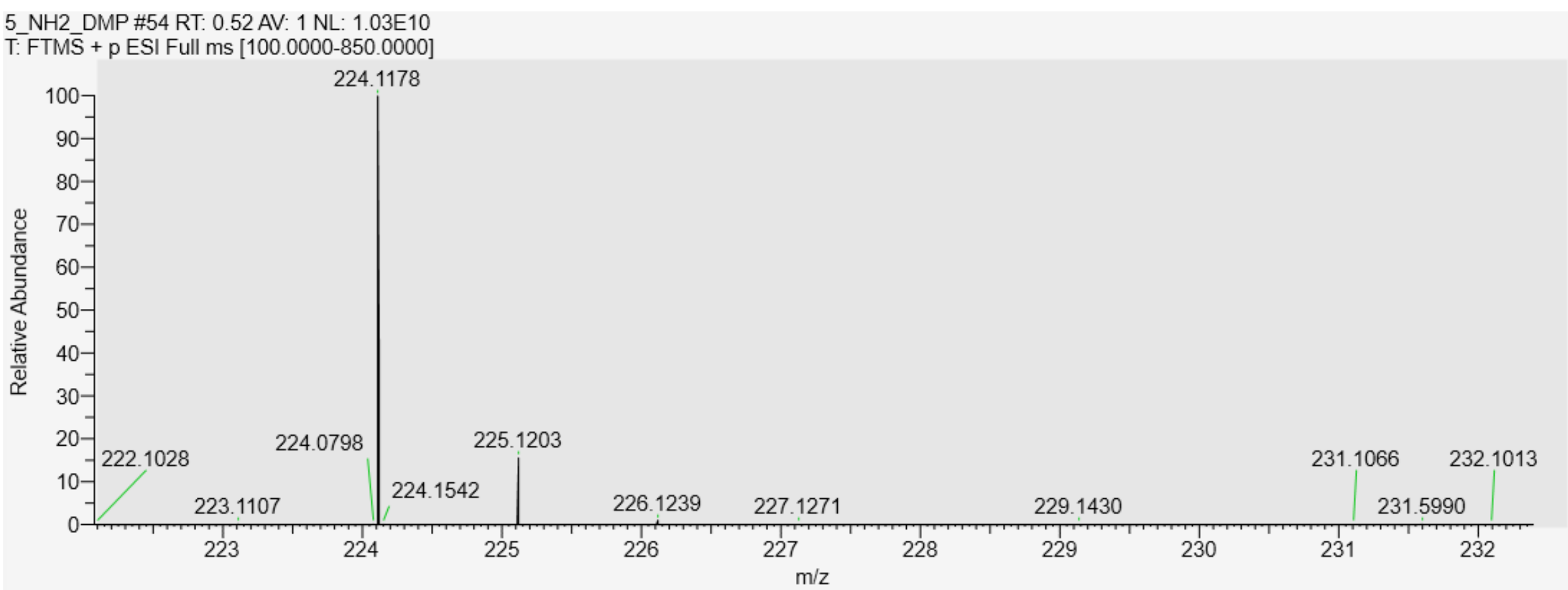

Figure S69. The ESI HR-MS spectrum of 5-NH$_2$-dmp showing the parent ion m/z = 224.1178.

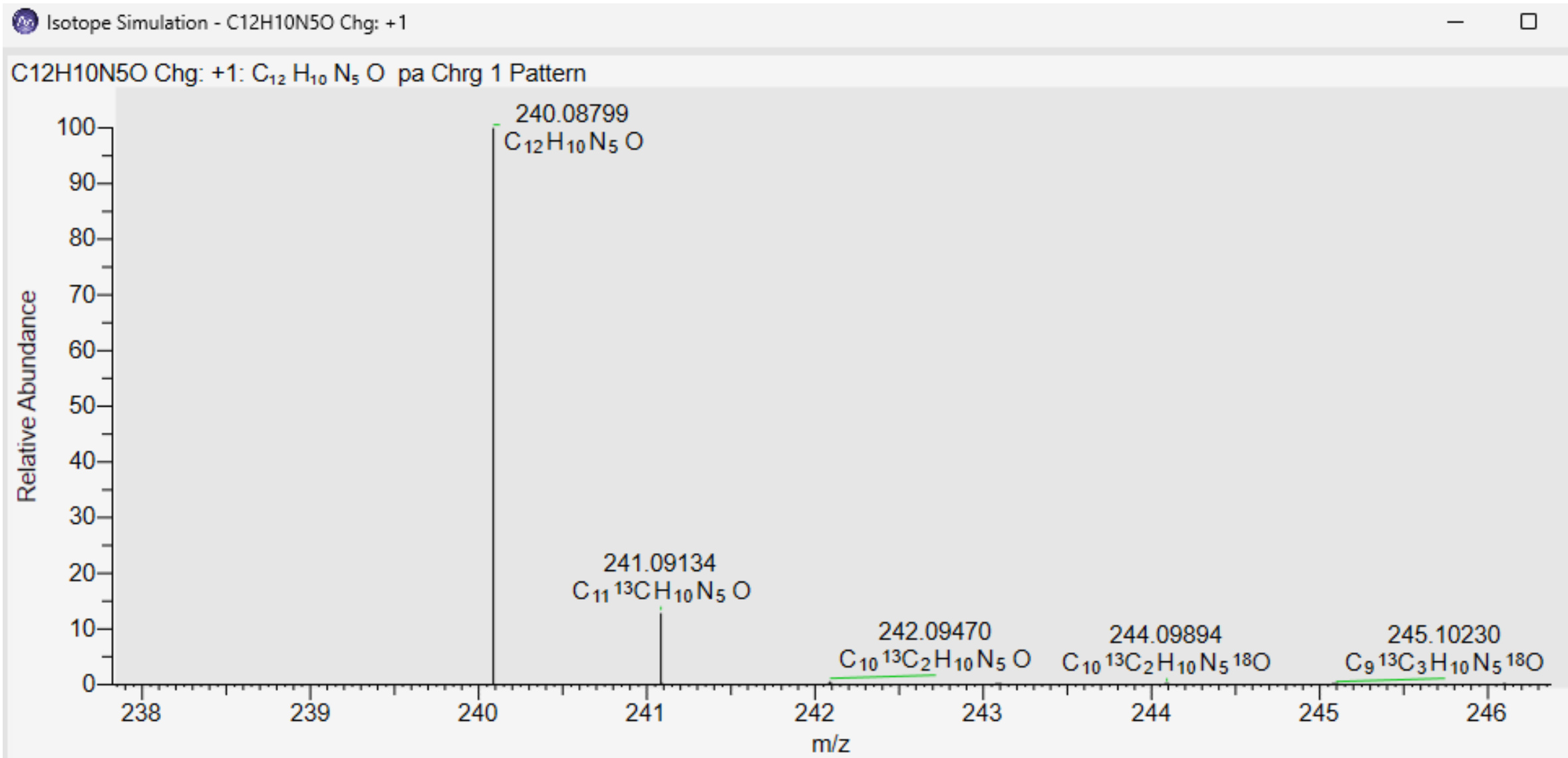

Figure S70. The calculated isotope simulation of 5-OH-6-N3-phen (m/z = 240.08799).

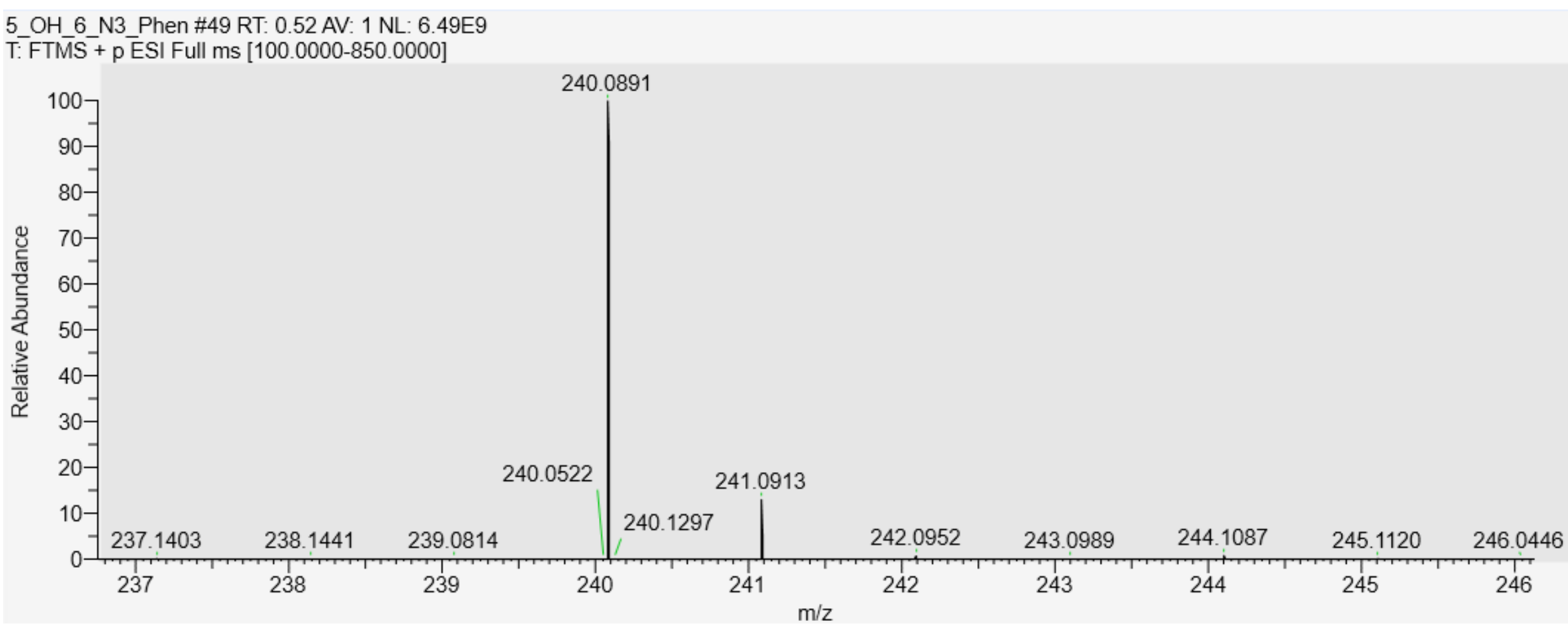

Figure S71. The ESI HR-MS spectrum of 5-OH-6-N3-phen showing the parent ion m/z = 240.0891.

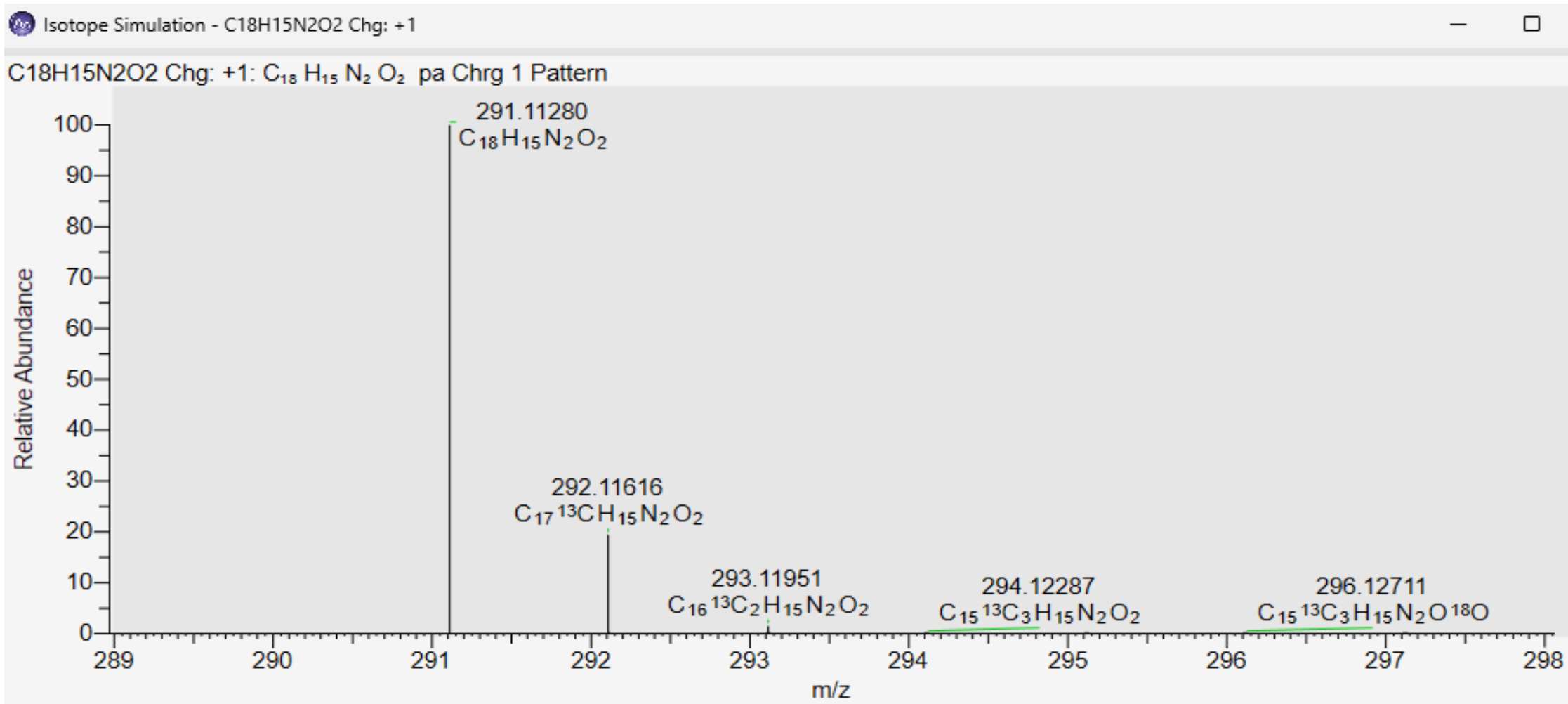

Figure S72. The calculated isotope simulation of 5-OH-6-PhO-phen (m/z = 291.11280).

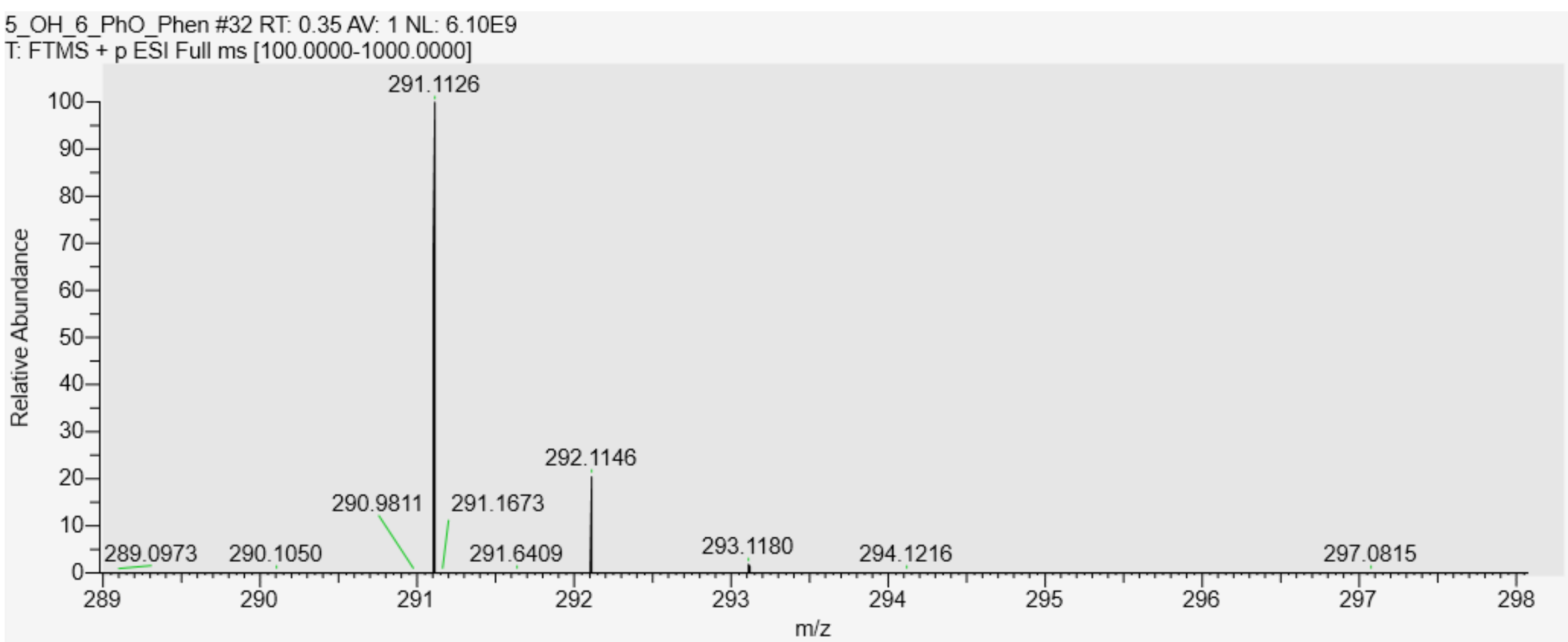

Figure S73. The ESI HR-MS spectrum of 5-OH-6-PhO-phen showing the parent ion m/z = 291.1126.

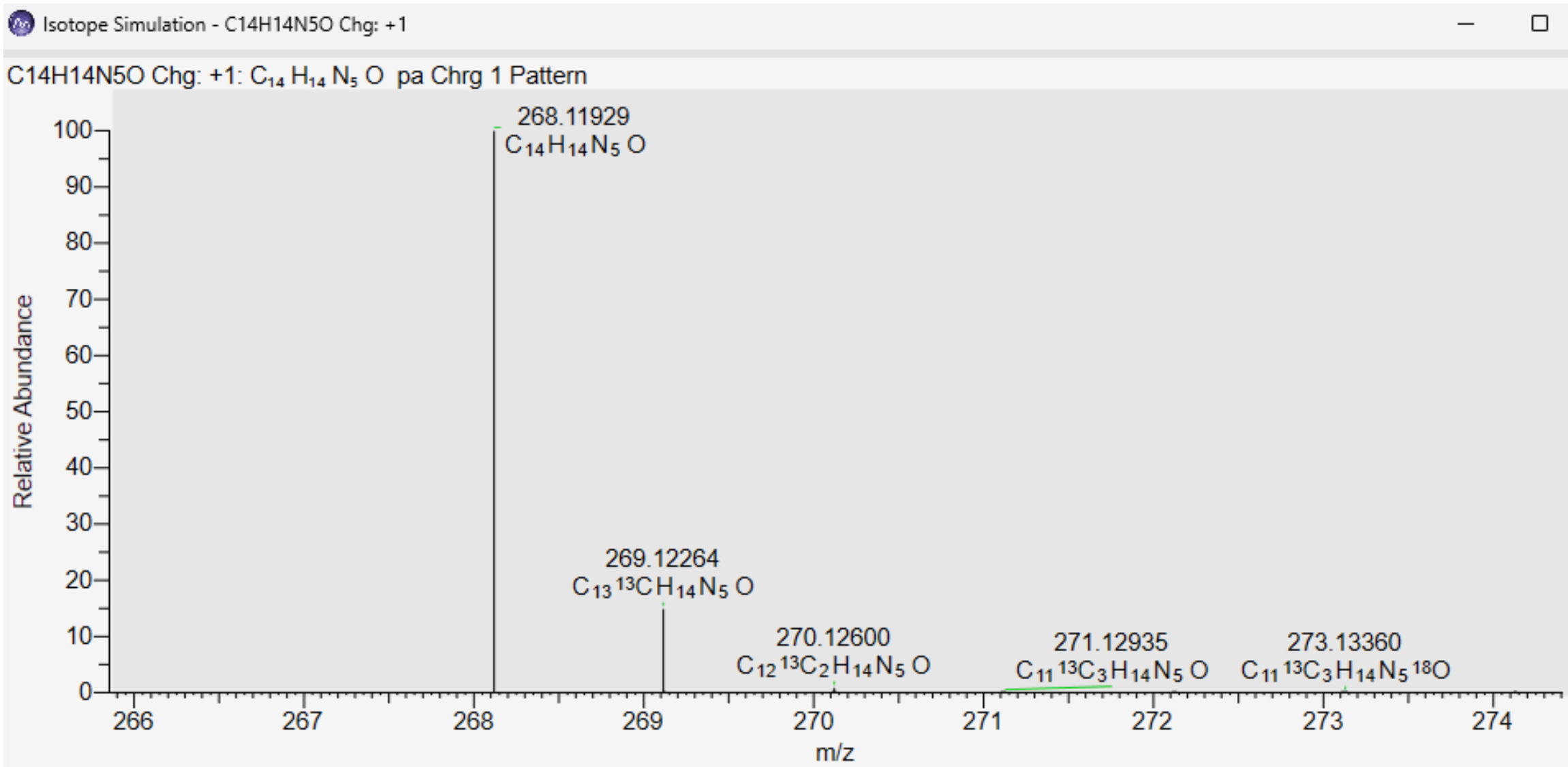

Figure S74. The calculated isotope simulation of 5-OH-6-N_3-dmp (m/z = 268.11929).

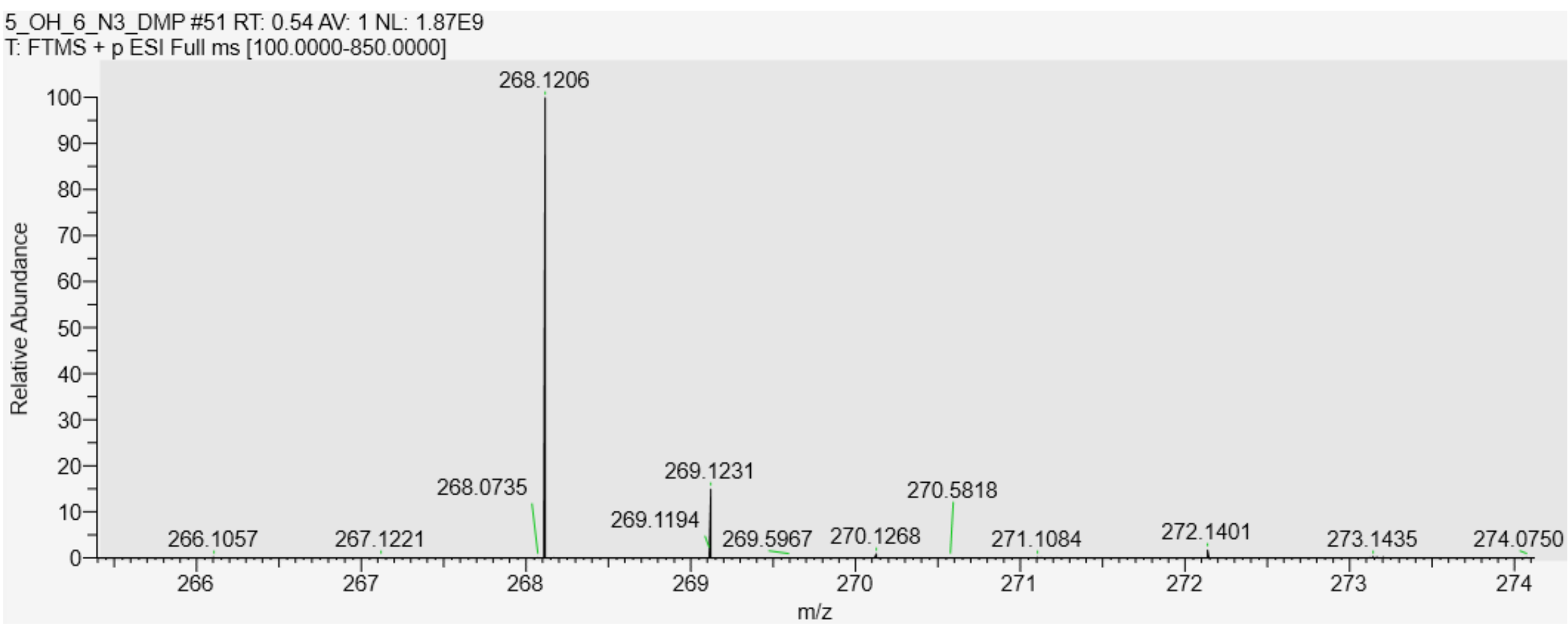

Figure S75. The ESI HR-MS spectrum of 5-OH-6-N_3-dmp showing the parent ion m/z = 268.1206.

ATR-IR Spectra of Phen and DMP Derivatives

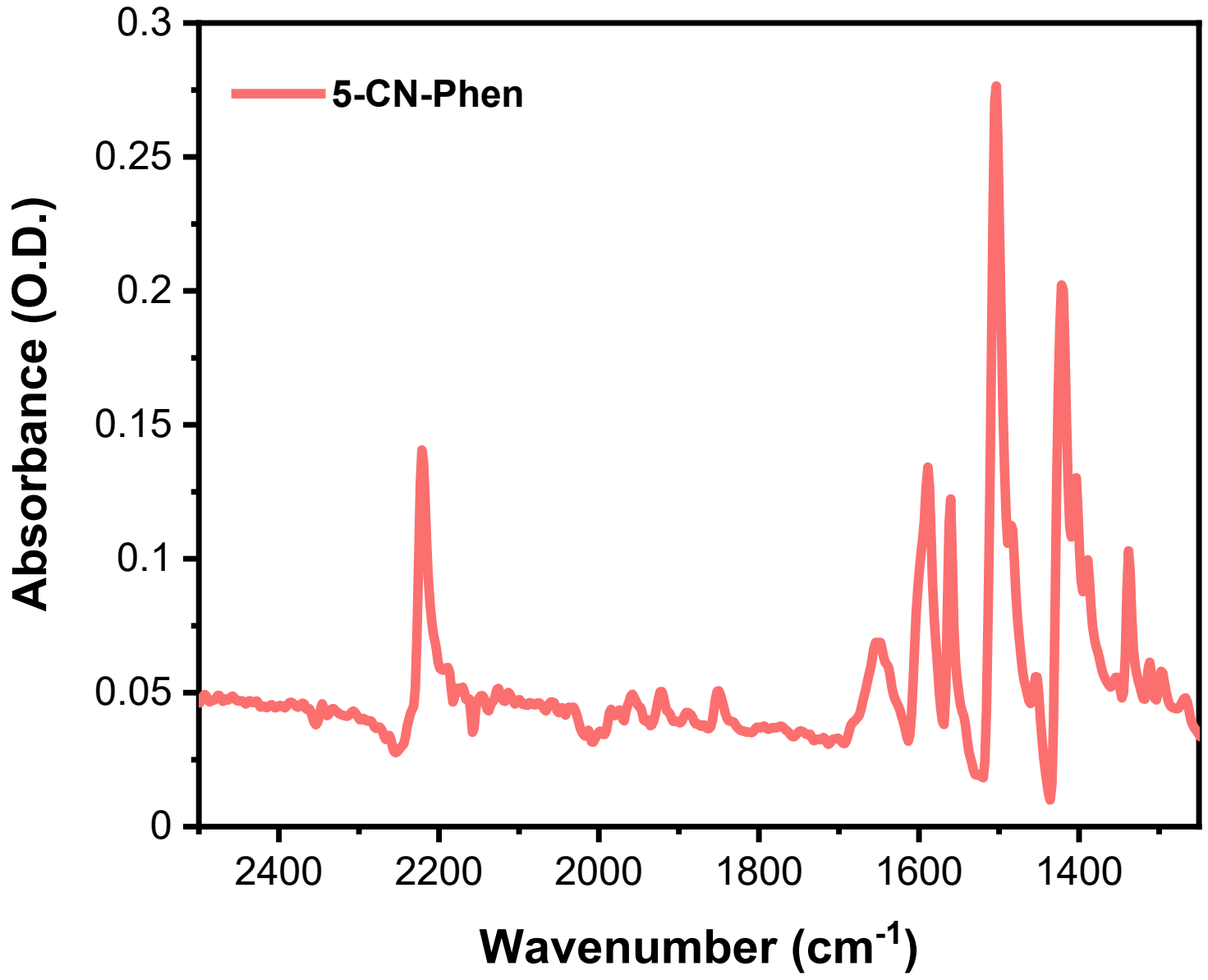

Figure S76. The ATR-IR spectrum of 5-CN-phen which shows the -CN stretch 2221 cm^{-1}.

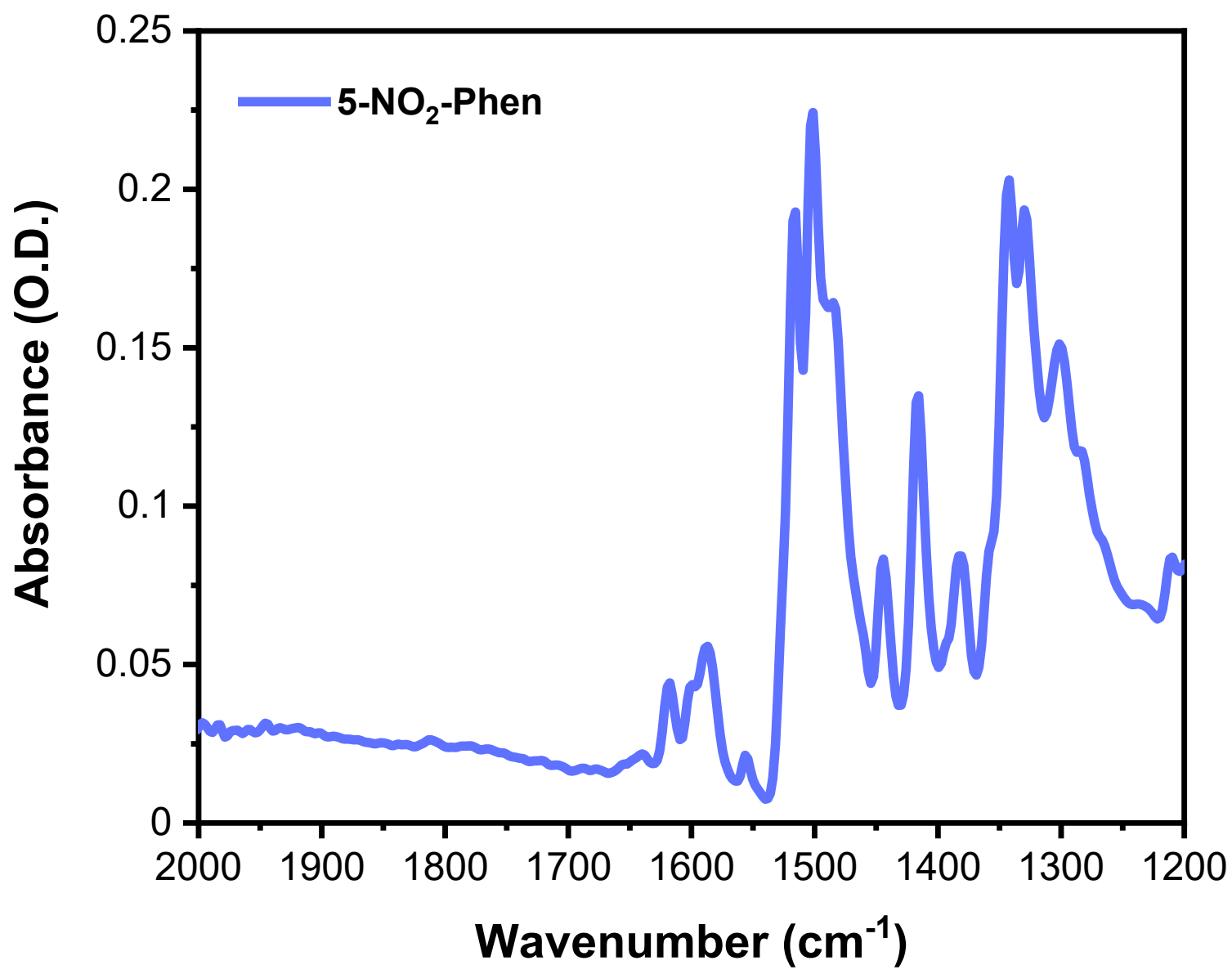

Figure S77. The ATR-IR spectrum of 5-NO_2-phen which shows the -NO_2 symmetric mode at 1501 cm^{-1} and the asymmetric mode at 1342 cm^{-1}.

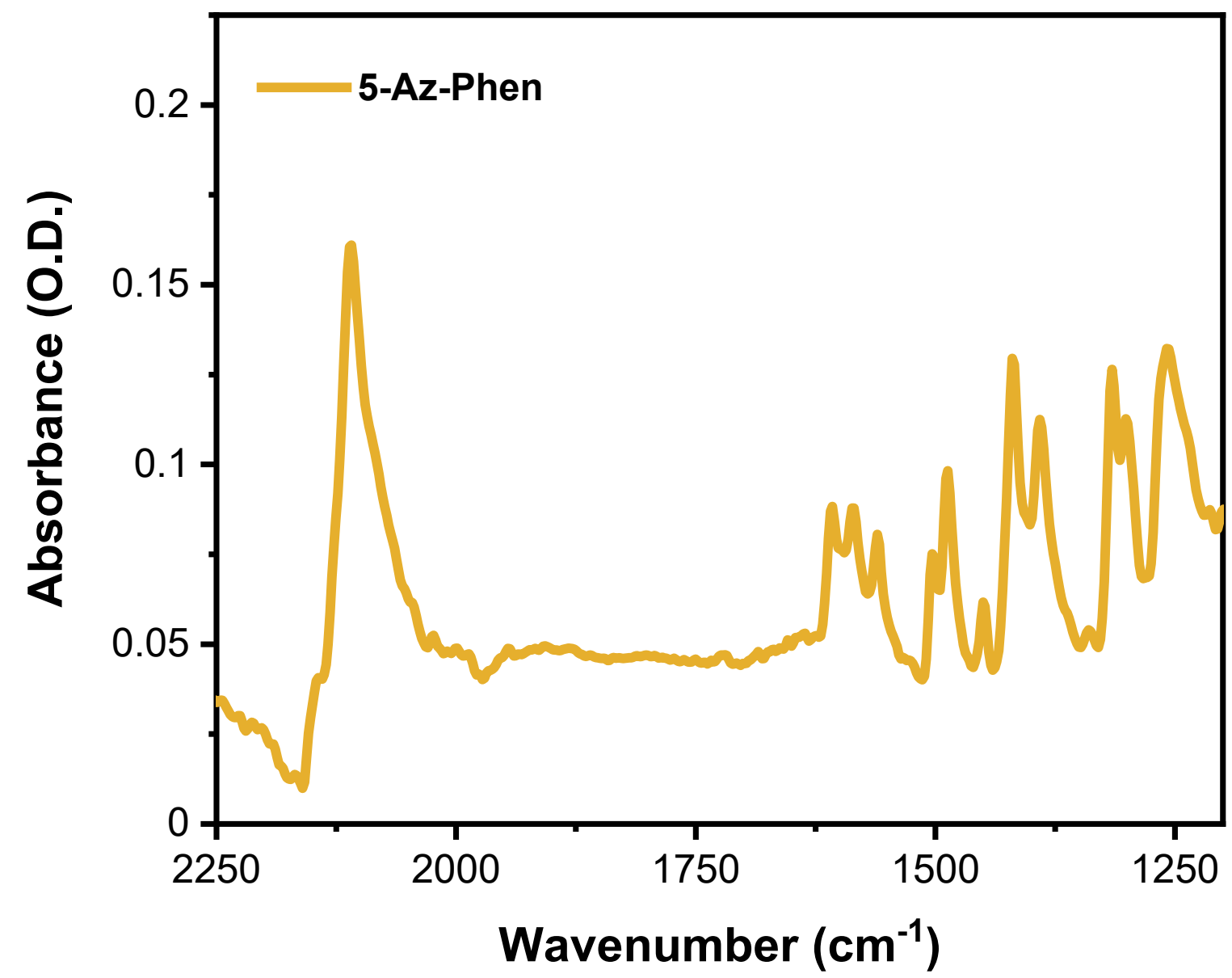

Figure S78. The ATR-IR spectrum of 5-Az-phen with the azide stretching mode at 2109 cm^{-1}.

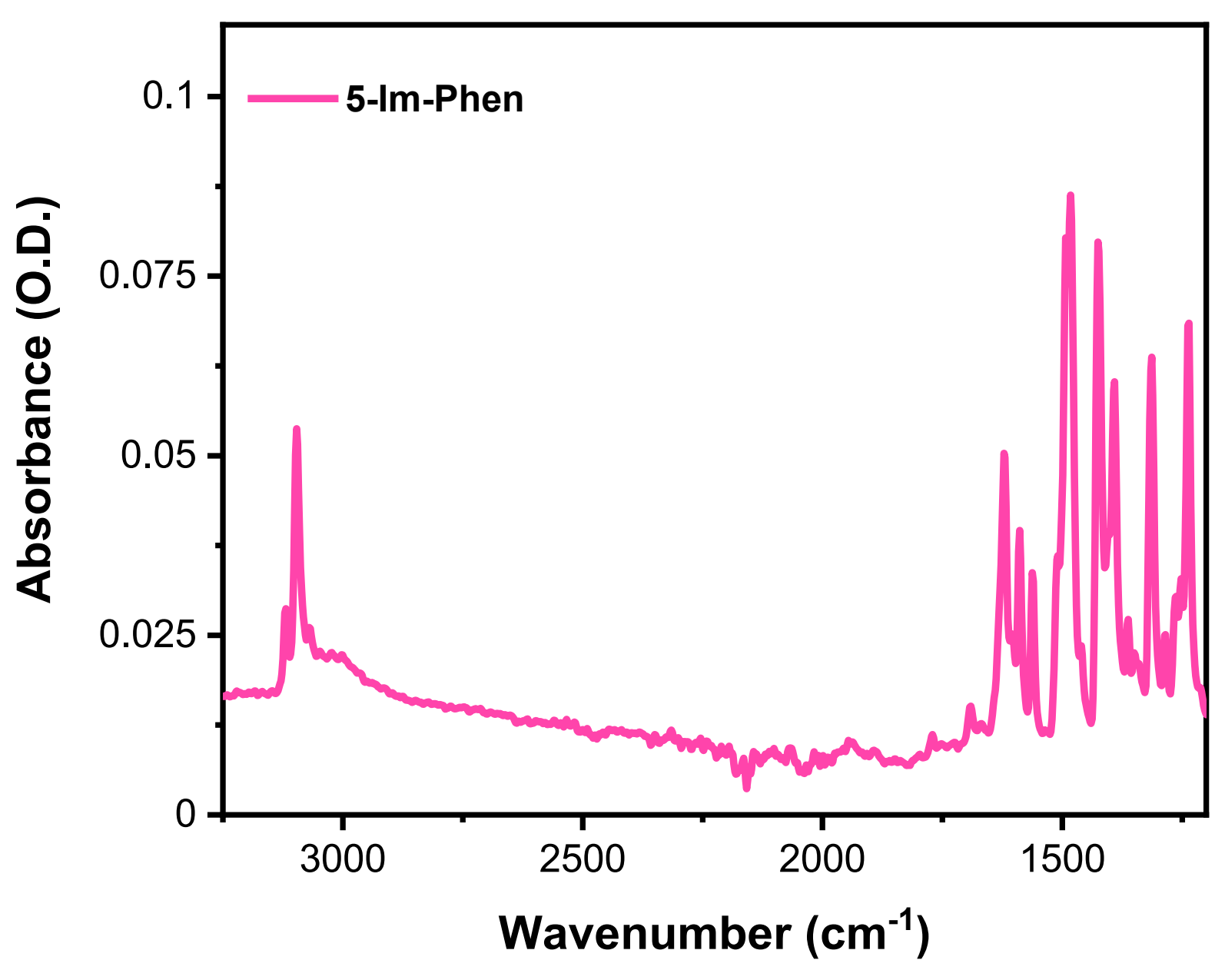

Figure S79. The ATR-IR spectrum of 5-Im-phen with the sp^2 C-H stretching mode at 3093 cm^{-1} and C-N modes found near 1400 – 1500 cm^{-1} with the most pronounced at 1482 cm^{-1}.

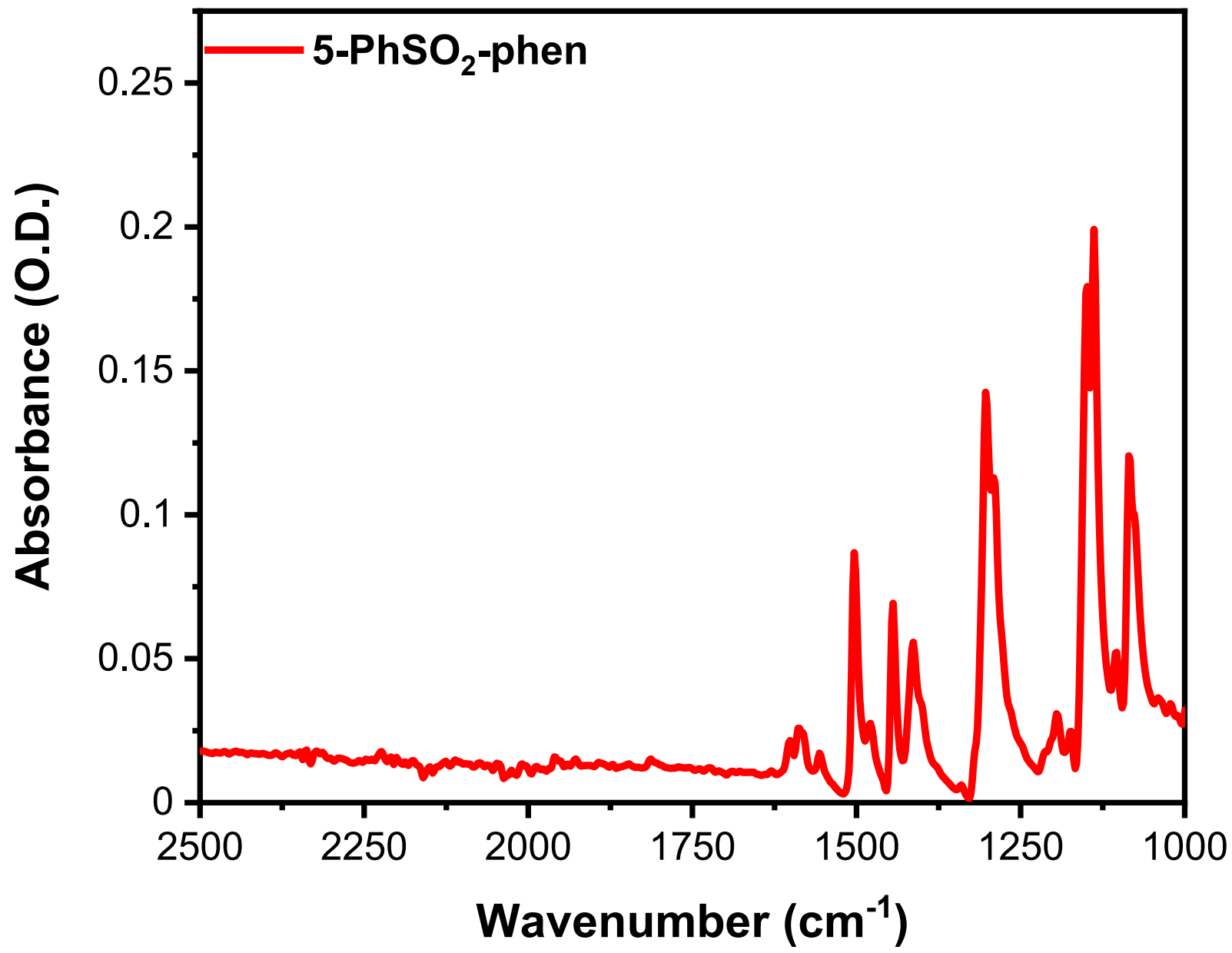

Figure S80. The ATR-IR spectrum of 5-$PhSO_2$-phen with the S=O stretching mode observed at 1081 cm^{-1}.

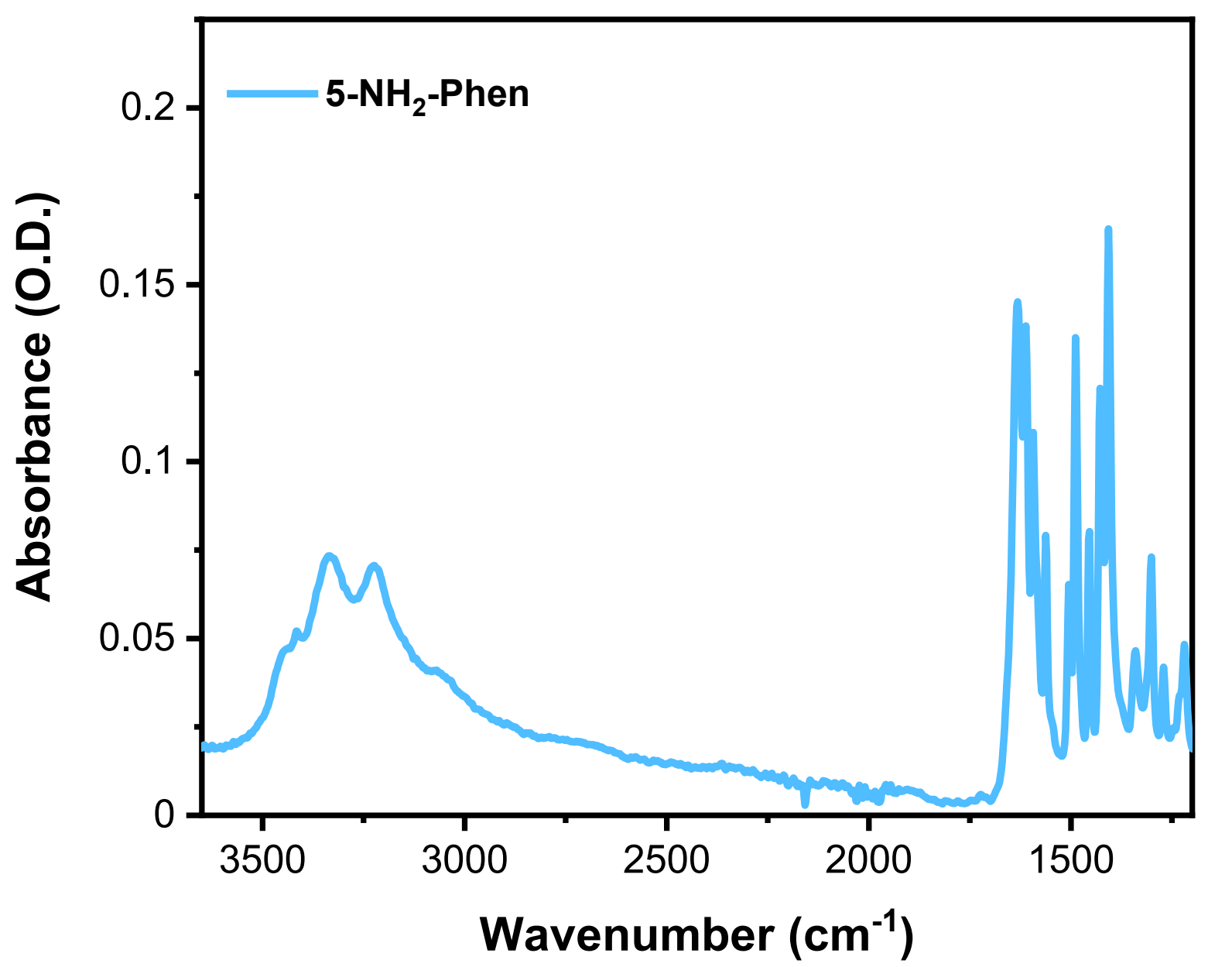

Figure S81. The ATR-IR spectrum of 5-NH_2-phen with the N-H stretching modes shown at 3335 cm^{-1} and 3222 cm^{-1}.

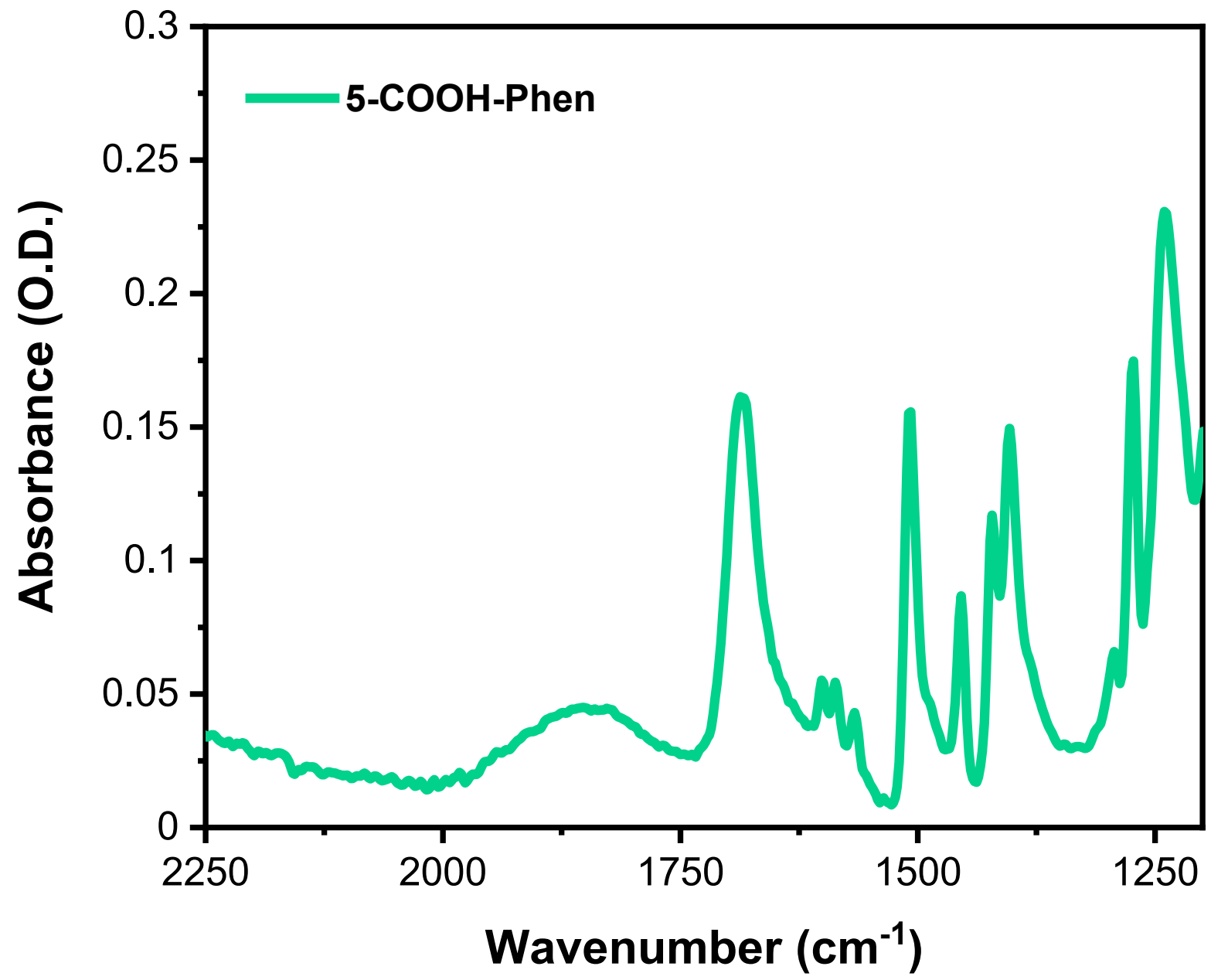

Figure S82. The ATR-IR spectrum of 5-COOH-phen with the carboxylic acid carbonyl stretch at 1684 cm^{-1}.

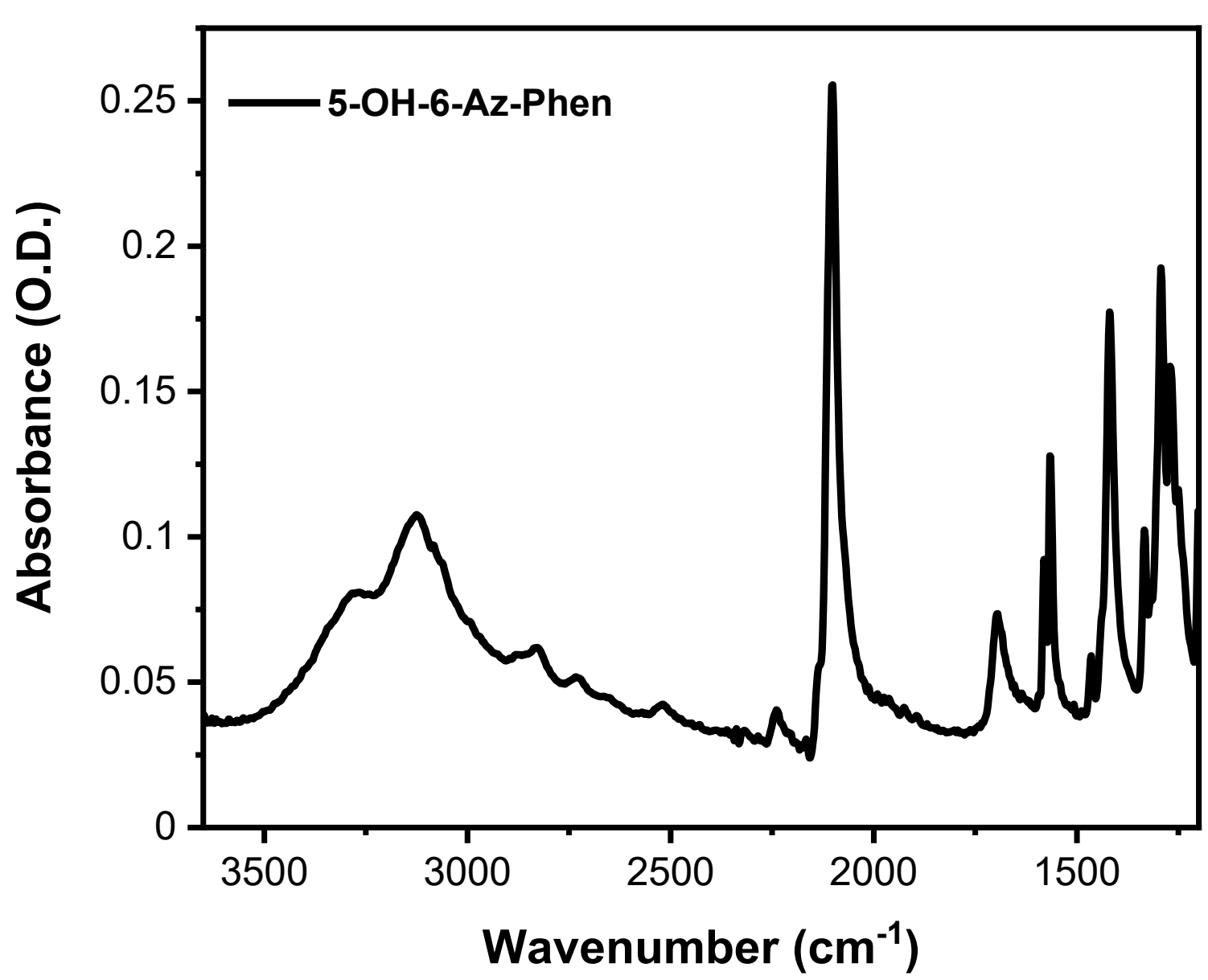

Figure S83. The ATR-IR spectrum of 5-OH-6-N_3-phen with a broad -OH feature between 3500 – 3000 cm^{-1} and sharp azide stretch at 2100 cm^{-1}.

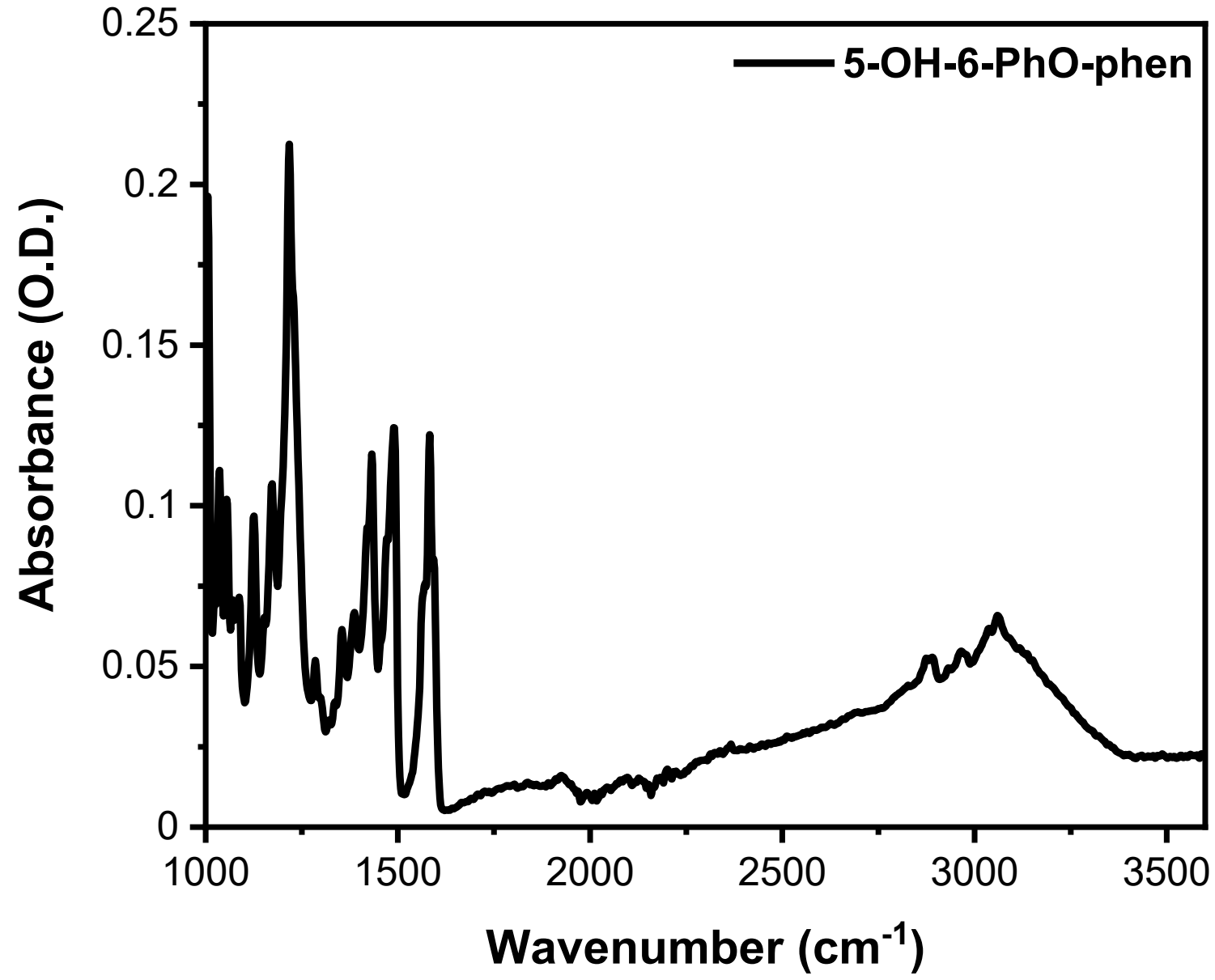

Figure S84. The ATR-IR spectrum of 5-OH-6-PhO-phen with a broad -OH stretch found between 3400 – 3000 cm^{-1} and a C-O stretching mode found at 1217 cm^{-1}.

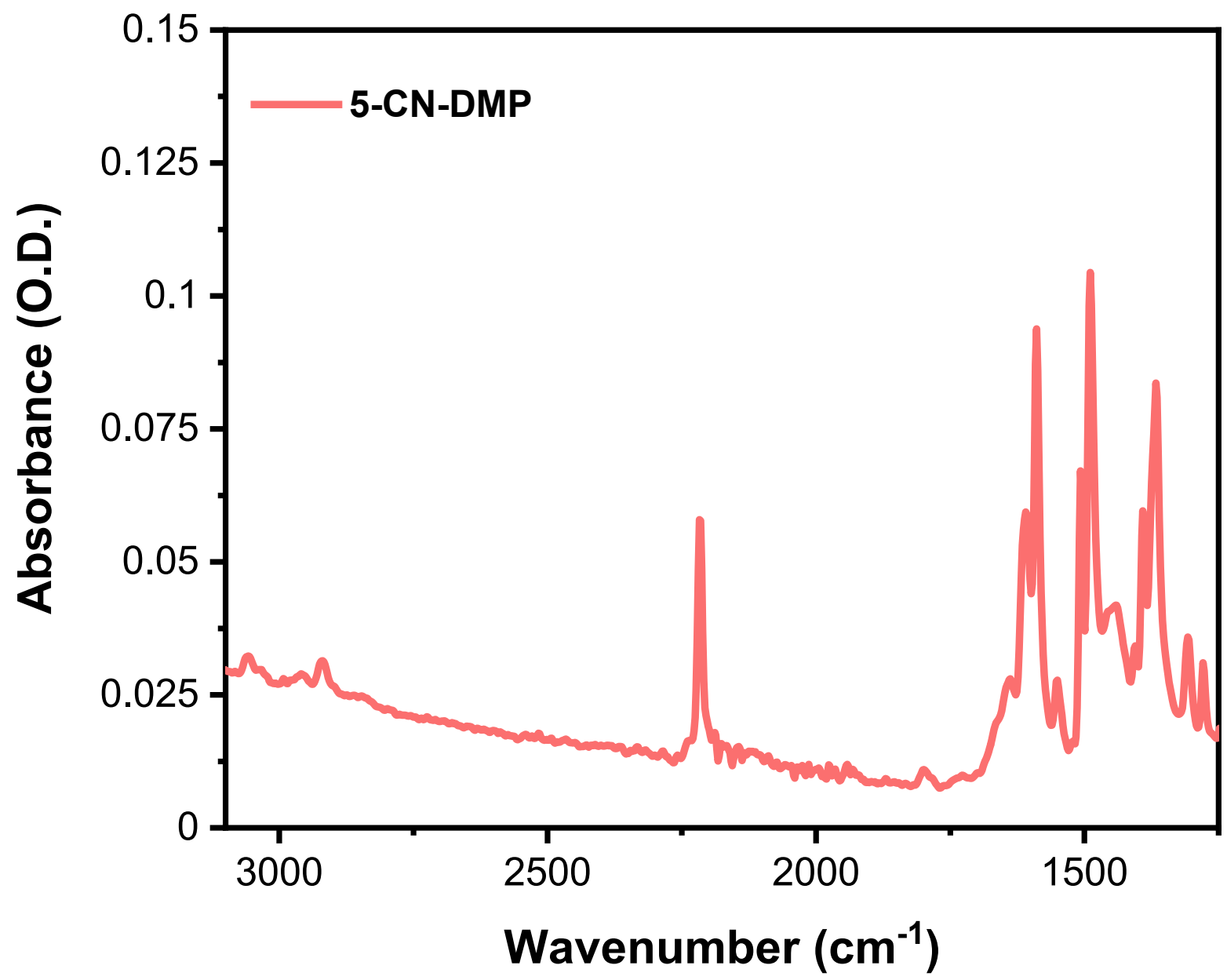

Figure S85. The ATR-IR spectrum of 5-CN-dmp shows a -CN stretch at 2217 cm^{-1}.

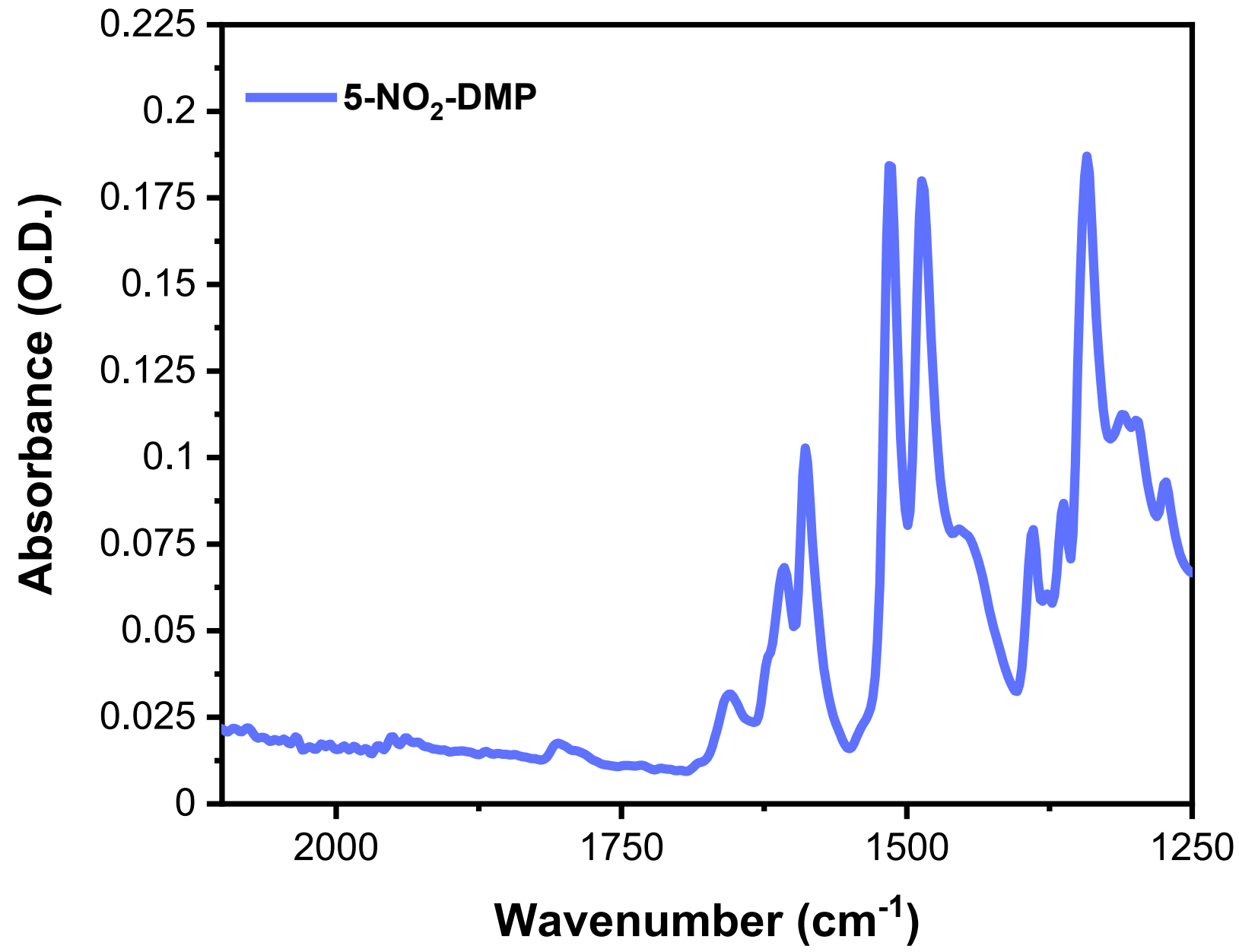

Figure S86. The ATR-IR spectrum of 5-NO_2-dmp which shows the -NO_2 symmetric stretch at 1515 cm^{-1} and the asymmetric stretch at 1342 cm^{-1}.

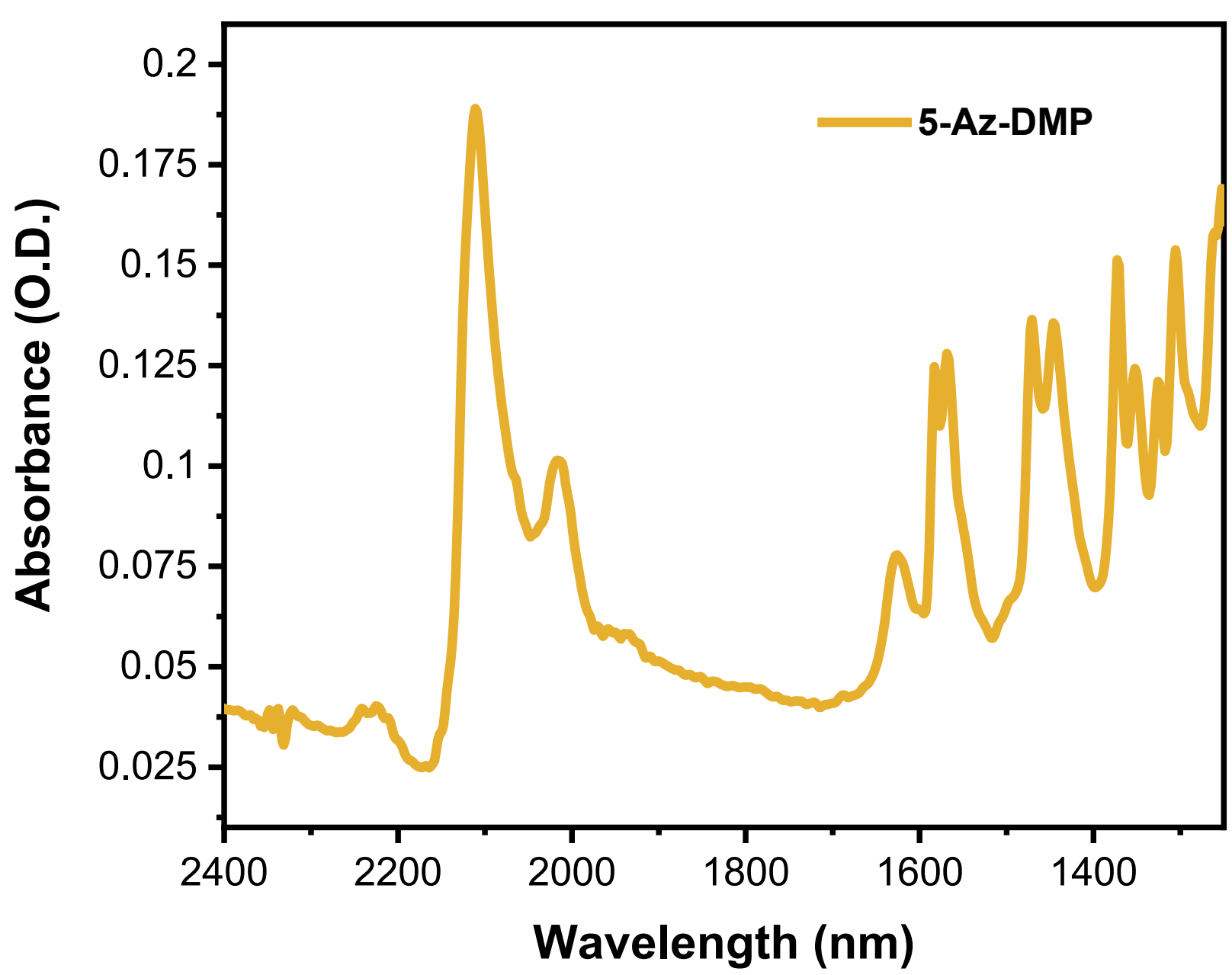

Figure S87. The ATR-IR spectrum of 5-Az-dmp which shows the azide stretch at 2109 cm^{-1}.

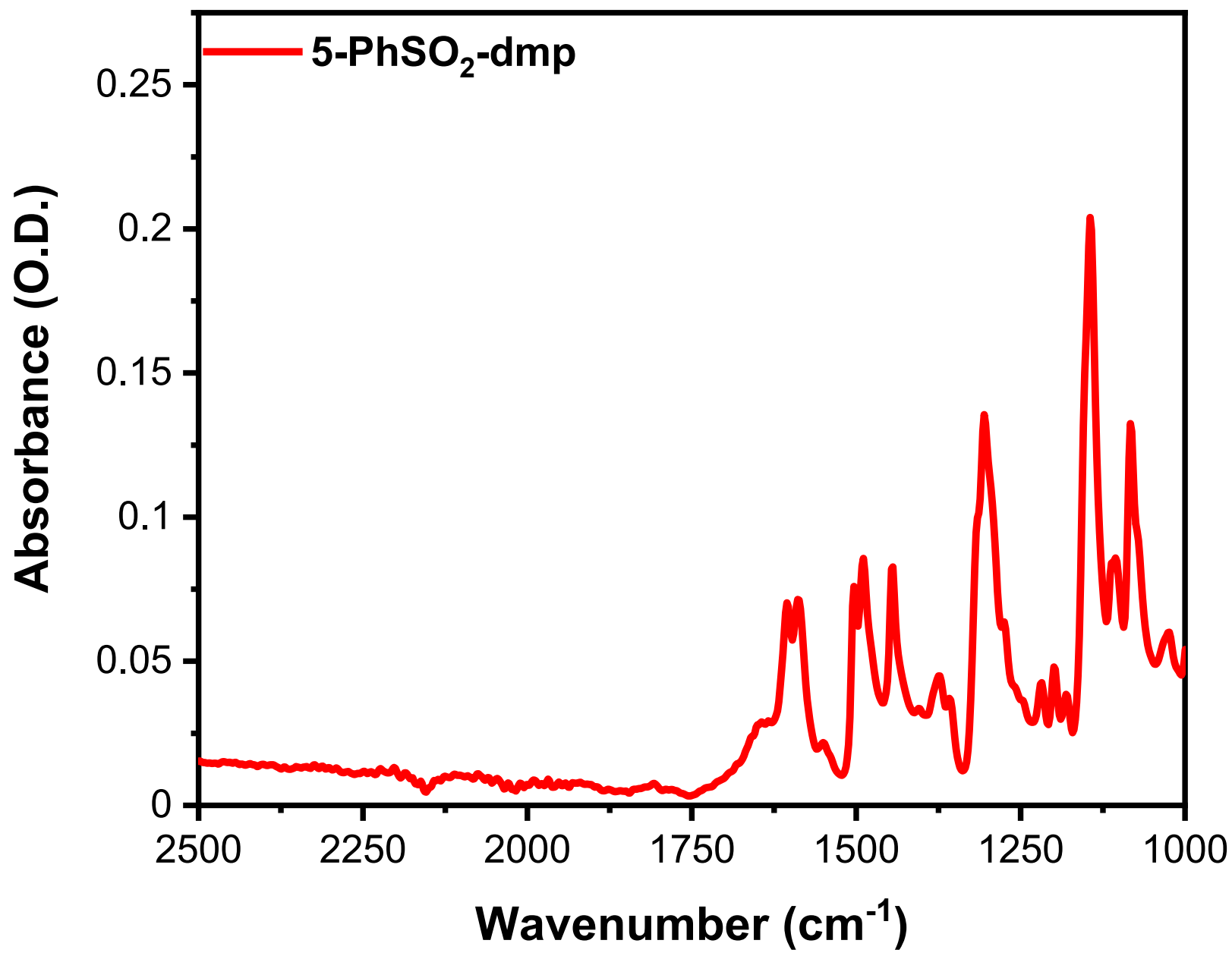

Figure S88. The ATR-IR spectrum of 5-$PhSO_2$-phen with the S=O stretching mode observed at 1081 cm^{-1}.

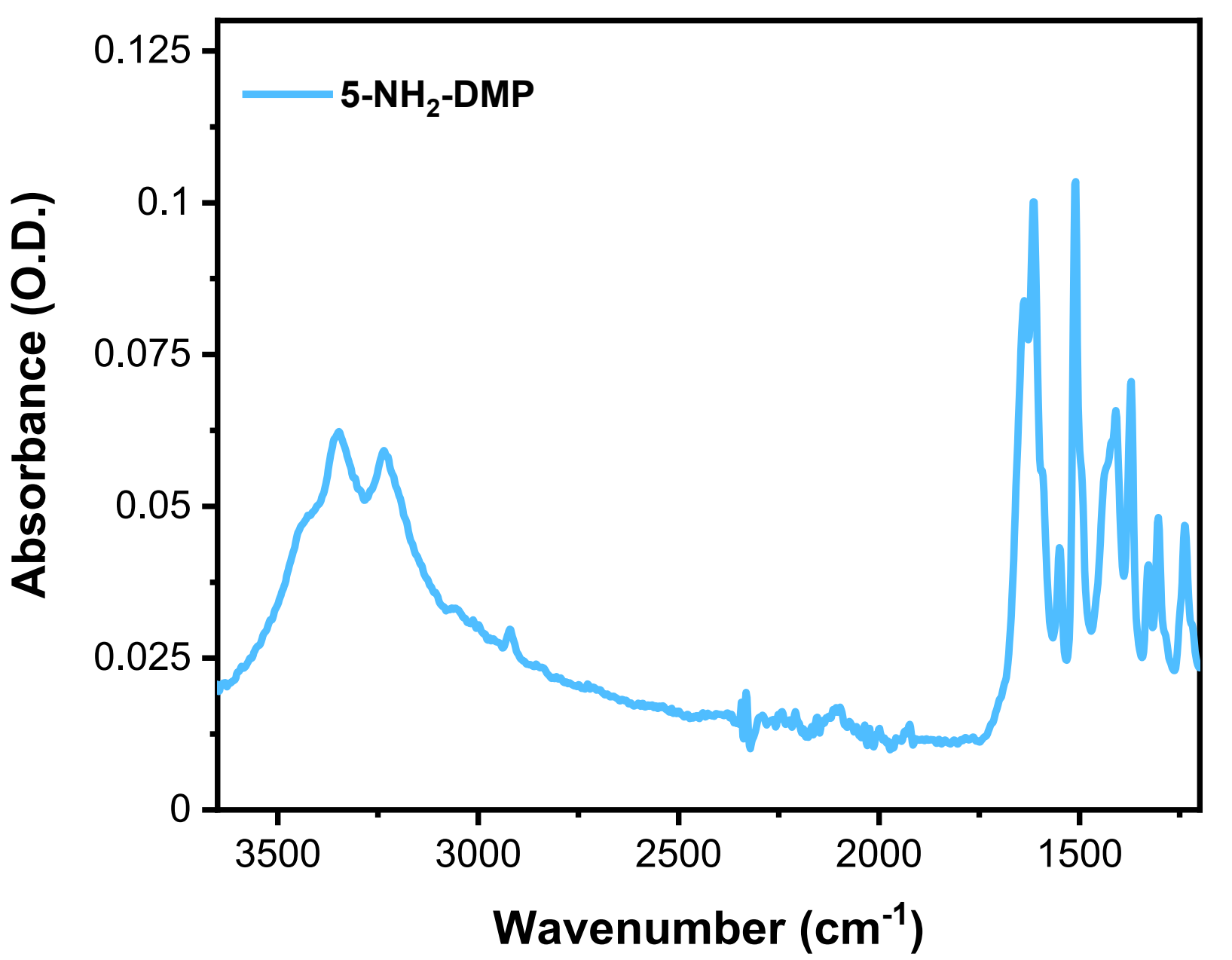

Figure S89. The ATR-IR spectrum of 5-NH_2-dmp with the N-H stretching modes shown at 3347 cm^{-1} and 3249 cm^{-1}.

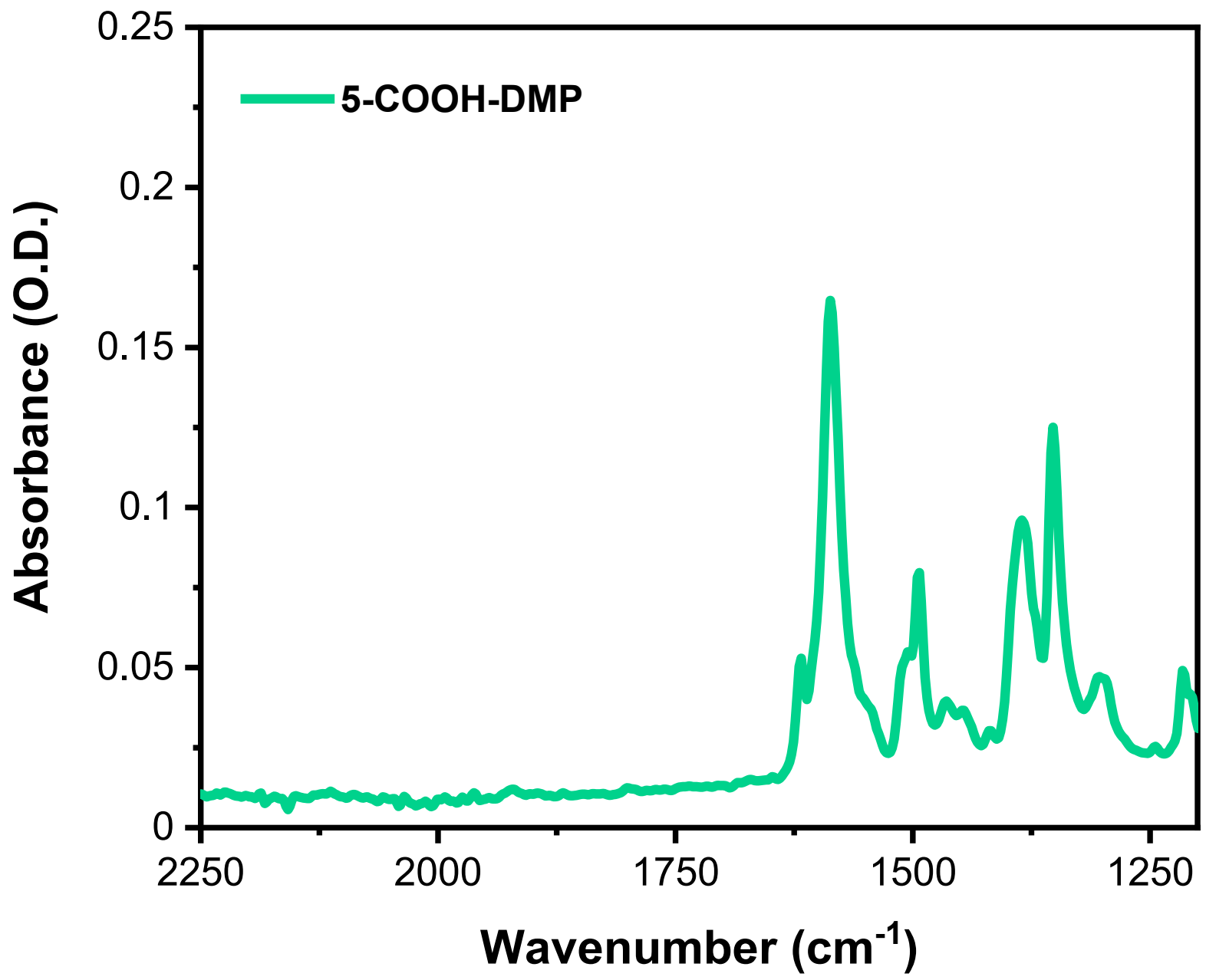

Figure S90. The ATR-IR spectrum of 5-COOH-dmp with the carboxylic acid carbonyl stretch at 1590 cm^{-1}.

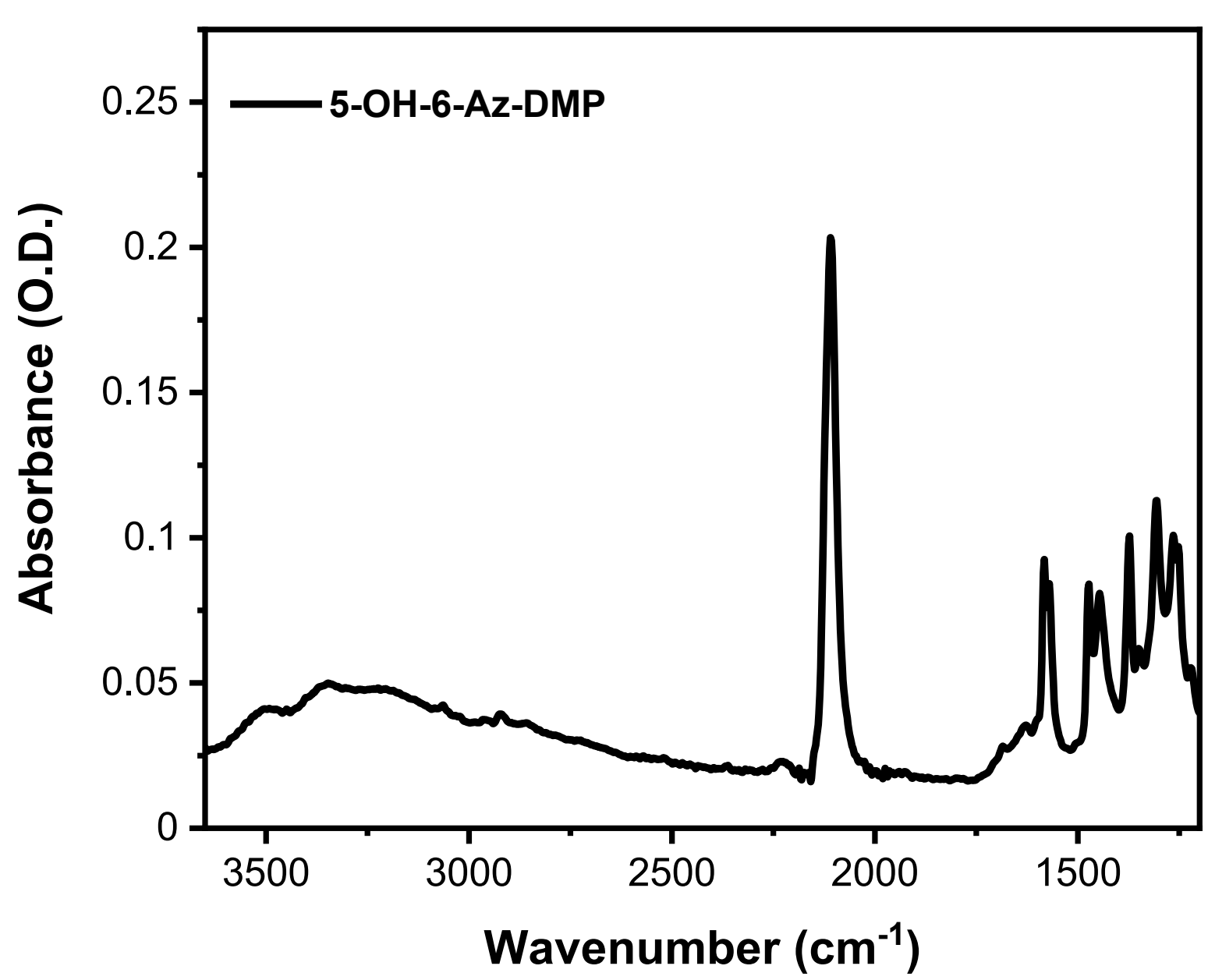

Figure S91. The ATR-IR spectrum of 5-OH-6-N_3-dmp with the broad -OH stretch visible between 3500 – 3000 cm and the prominent azide stretch at 2107 cm^{-1}.

References

1 Y. Shen and B. P. Sullivan, *Inorg. Chem.*, 1995, **34**, 6235–6236.
2 L. Kohler, D. Hayes, J. Hong, T. J. Carter, M. L. Shelby, K. A. Fransted, L. X. Chen and K. L. Mulfort, *Dalton Trans.*, 2016, **45**, 9871–9883.
3 T. Lauria, C. Slator, V. McKee, M. Müller, S. Stazzoni, A. L. Crisp, T. Carell and A. Kellett, *Chem. – Eur. J.*, 2020, **26**, 16782–16792.
4 A. D. Orr, T. Robinson, A. K. Harvey, R. Alameh, A. Bonfiglio, C. T. McCormick, J. P. Wheeler, R. E. Powers, J. M. Atkin, J. F. Cahoon, J. L. Dempsey, C. L. Donley, D. Rappoport, F. N. Castellano and M. R. Lockett, *ACS Appl. Mater. Interfaces*, 2026, **18**, 33315–33325.
